# Research on the perspectives of people affected by dementia with Lewy bodies: a scoping review

**DOI:** 10.1186/s13195-025-01760-4

**Published:** 2025-05-26

**Authors:** Paula Sinead Donnelly, Aoife Sweeney, Anthony P. Passmore, Noleen K. McCorry, Joseph P. M. Kane

**Affiliations:** Centre for Public Health, Queen’s University Belfast, Institute of Clinical Science B, Royal Victoria Hospital, Grosvenor Road, Belfast, BT12 6BA UK

**Keywords:** Dementia with Lewy bodies, Scoping review, Lewy body dementia, Perspectives, Dementia

## Abstract

**Background:**

Dementia with Lewy bodies (DLB) is associated with specific challenges, including heterogeneity in clinical presentation and a less favourable prognosis relative to other dementia subtypes. These challenges necessitate person-centred care informed by the perspectives of those affected by DLB. This scoping review aimed to map the extent, type, and nature of research focusing on the perspectives of individuals with DLB and their care partners.

**Methods and results:**

We searched six databases and two grey literature sources to identify all types of work providing information on the perspectives of individuals with DLB and/or their care partners. Two reviewers independently applied study selection criteria. Data from eligible articles were extracted, charted, and summarised using descriptive numerical analysis and basic qualitative content analysis. The review included 140 sources, of which 89.3% were research articles. Excluding non-structured reflections and commentary articles (*n* = 4), 68.4% of sources were quantitative and 65.4% were cross-sectional. The most common method of collecting perspective data was standardised measures assessing multidimensional concepts, such as caregiver burden. In total, 13 topics were identified, with ‘emotional and psychological well-being’ (*n* = 64) being the most widely investigated. There was a significant gap before the next most common topic: ‘perspectives related to the symptom and illness experience’ (*n* = 34).

**Conclusion:**

While a range of methods was identified in this review, the evidence base is characterised by a predominance of standardised measures, with comparatively less use of qualitative approaches or non-standardised tools incorporating bespoke questions tailored to the study population. There was a disproportionate focus on specific topics, leading to research gaps. We recommend exploring novel methods to systematically capture perspectives in DLB cohorts, particularly on topics of highest priority to those affected.

**Supplementary Information:**

The online version contains supplementary material available at 10.1186/s13195-025-01760-4.

## Introduction

Lewy body dementia (LBD) encompasses two entities: dementia with Lewy bodies (DLB) and Parkinson’s disease dementia (PDD) [1]. DLB is characterised by four core clinical features: visual hallucinations, rapid eye movement sleep behaviour disorder, cognitive fluctuations, and spontaneous motor features of parkinsonism [2]. While DLB shares characteristics with other forms of dementia, it also presents specific challenges, including heterogeneity in clinical presentation and trajectory [3], and higher rates of both care partner (CP) stress [4] and hospitalisation [5]. Management is complicated by the fact that treatment of one symptom can exacerbate another [6], such as the treatment of parkinsonism precipitating psychosis [7]. This necessitates person-centred care strategies informed by the perspectives of those affected by DLB, as stakeholder-informed decision-making shapes healthcare systems that are more responsive to patients and CPs [8].

Studies of all-cause dementia cohorts have explored the perspectives of those affected across a range of topics including experiences, needs, quality of life (QoL), and CP burden [9–13]. However, the specific perspectives of those affected by DLB may be overlooked in aggregated cohorts. Similarly, given their clinical overlap, DLB and PDD are often conflated. A recent review examined lived experiences in LBD, focusing on day-to-day experiences and healthcare interactions [14]. Although offering valuable insights, aggregating LBD samples risks assuming homogeneity of experience. For instance, the temporal onset of dementia and parkinsonism differs between the two diagnoses [2]; some individuals with DLB may never develop parkinsonism; cognitive decline profiles differ [15]; and individuals with DLB may respond less favourably to dopaminergic therapies [2, 16]. Such distinctions shape experiences and care needs, highlighting the need for research focused specifically on DLB cohorts.

Various methods exist for capturing perspectives, including surveys, interviews, patient-reported outcome measures, patient-reported experience measures, and stated preference methods [17–20]. These methods differ in their aims, scope, and the types of information captured. For example, standardised instruments offer structured, validated assessments; however, many rely on closed-response formats, limiting the depth and flexibility of responses. In contrast, qualitative methods aim to generate rich data through close engagement with respondents’ perspectives [21], though they may pose challenges in dementia populations [22]. Given the breadth of available methods, each with specific strengths and limitations, it is important to map their application in DLB to identify methodological gaps.

No work has yet examined and mapped the extent, type, and nature of research activity specifically on the perspectives of those affected by DLB. This review focuses on DLB and considers a broader range of topics beyond day-to-day experiences and healthcare interactions. It aims to systematically map the types of methods used to capture perspective data.

### Aims

Using the Food and Drug Administration’s (FDA) definition of ‘patient perspective’ [23], this scoping review aimed to explore published work on the perspectives of individuals with DLB and their CPs, focusing on data beyond the characterisation and measurement of clinical symptoms.

### Methodology

The scoping review approach was selected as it facilitates the identification and mapping of available evidence, the identification and analysis of knowledge gaps, and the examination of how research is conducted [24]. To compile this review, we utilised the five-stage framework by Arksey and O’Malley (identifying the research question; identifying relevant articles; screening and study selection; data charting; and collating, summarising, and reporting the data) [25], as well as the *Joanna Briggs Institute* methodology [26, 27]. An a priori protocol was registered on the Open Science Framework (https://doi.org/10.17605/OSF.IO/ZTJX2). Guidelines for the Preferred Reporting Items for Systematic Reviews and Meta-Analyses Extension for Scoping Reviews (PRISMA-ScR) were followed [28] (Additional file 1, Appendix 1).

### Identifying the research question

The research questions were:

1. Which methods have been used to elicit the perspectives of people with DLB and their CPs?
2. On which topics have the perspectives of patients and CPs been sought?

### Identifying relevant articles

We employed a three-step search strategy. Firstly, an initial search on MEDLINE and CINAHL identified key terms in consultation with a subject librarian. Secondly, we searched MEDLINE ALL, EMBASE, CINAHL Plus, PsycINFO, PubMed and Web of Science Core Collection from their inception, as well as grey literature sources (Google Scholar and The Networked Digital Library of Theses and Dissertations (NDLTD)). All searches were conducted on 26 and 27 September 2023 and were limited to English-language sources. For search strings, see Additional file 1, Appendix 2. The first 100 results from both grey literature sources were retrieved. Sources published after the final search were included only if provided by the author in response to requests for additional information. Thirdly, reference lists of included sources were manually screened. During full-text screening, reference lists of review articles were screened to identify primary evidence sources.

Where necessary, we contacted authors for additional information, excluding sources if there was no response. Theses and reports with multiple work packages were included but superseded by peer-reviewed articles when possible. If peer-reviewed articles covered all relevant data, the original source was excluded; otherwise, it was retained for any additional relevant data.

### Inclusion and exclusion criteria

To be eligible, sources had to satisfy all the following criteria:

#### Types of sources

All types of published work, including peer-reviewed and grey literature, were included provided they collected data beyond the characterisation and measurement of diagnostic symptoms, regardless of whether this was the source’s primary focus.

#### Participants

The focus was on individuals diagnosed with DLB ante-mortem (including those with mixed diagnosis; diagnosis could be self-reported or established using any published criteria) at any stage of the disease, and their formal and informal CPs. Formal CPs were defined as paid professionals, including community care providers, but excluding clinicians. There were no restrictions regarding age, gender, or race.

We excluded sources focused exclusively on PDD, Parkinson’s disease (PD), or mild cognitive impairment in PD (PD-MCI) (i.e. Lewy body diseases [LBDis]). However, since DLB, PDD, and LBDis are so often conflated in the literature, it was necessary to include studies that included DLB within a larger cohort, such as LBD or LBDis.

#### Concept

The primary concept was ‘perspectives’, encompassing individuals’ views, perceptions, goals, priorities, concerns, opinions, experiences, and preferences, aligning with the FDA’s definition [23]. Our focus was on the methods used to capture these perspectives and the subject matter they addressed.

Eligible methods collected data beyond the characterisation and measurement of diagnostic clinical symptoms. Thus, while instruments measuring multidimensional concepts such as CP burden were included, unidimensional measures of core or supportive DLB symptoms defined in the fourth consensus report of the DLB Consortium were excluded [2]. This was because such measures did not capture broader aspects of perspective beyond symptom characterisation and measurement. Symptom scales that included items assessing multidimensional concepts, such as the Neuropsychiatric Inventory Caregiver Distress subscale (NPI-D) [29], were included.

#### Context

There were no limits to setting or geographical context.

#### Exclusion criteria

Non-English publications and quantitative studies relying on unidimensional measures of symptoms defined in the DLB criteria, including those relating to cognition and functioning [2].

### Screening and study selection

Google Scholar and database citations were imported into Covidence systematic review software, Veritas Health Innovation, Melbourne, Australia (available at www.covidence.org), where duplicates were removed. NDLTD citations were managed in Microsoft Excel, with duplicates manually removed.

**Fig. 1:**
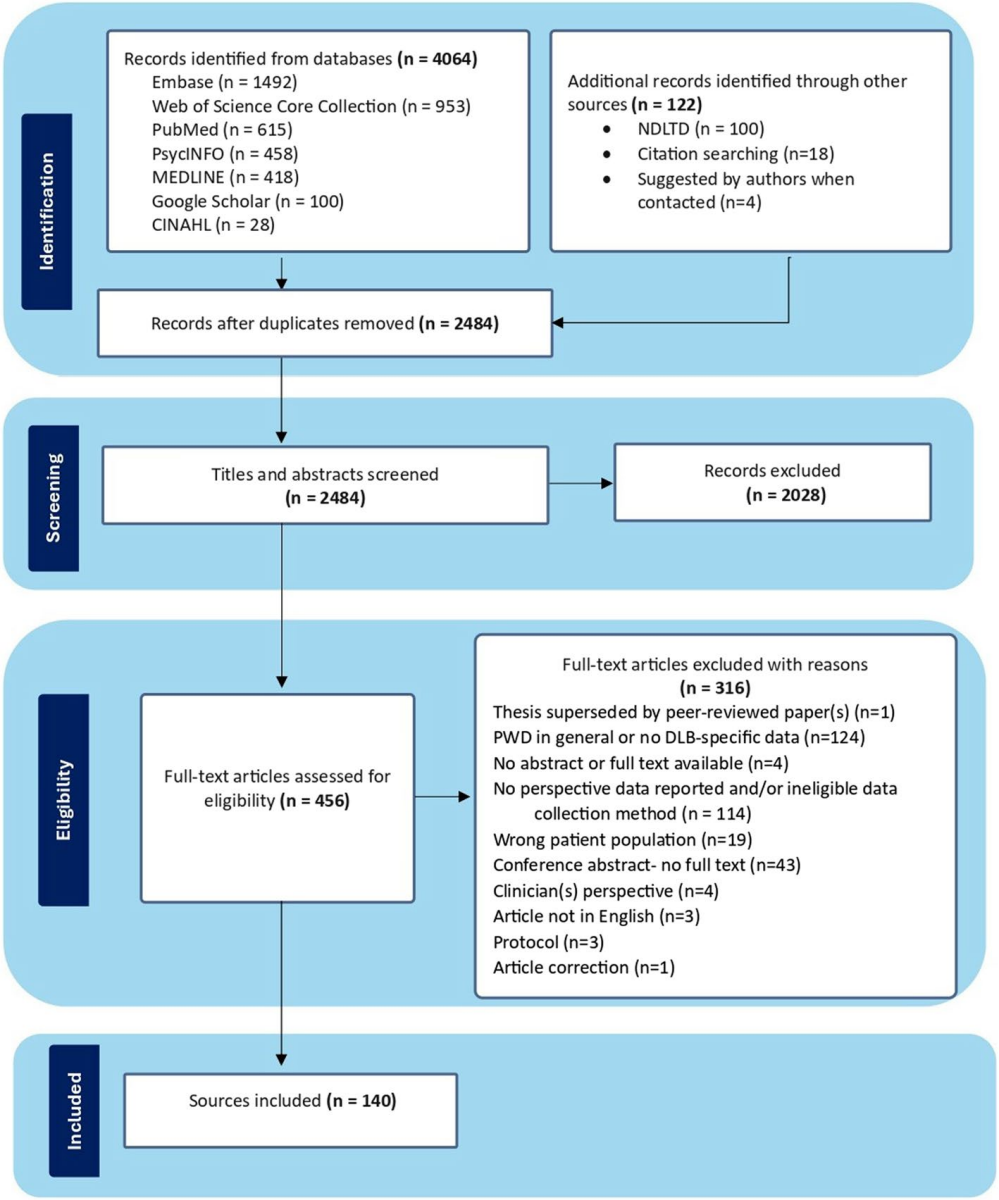
PRISMA flowchart describing the process for article selection. Abbreviations: PWD, People with dementia; NDLTD, Networked Digital Library of Theses and Dissertations

Two reviewers (PSD and AS) independently screened all titles and abstracts. Full texts were retrieved and screened independently, with disagreements resolved through discussion with a third reviewer (JK) (Fig. 1).

### Data charting

PSD developed a provisional data extraction form, which PSD and AS independently piloted with 10 sources, subsequently refining it based on discussion (Additional file 1, Appendix 3). The final form and charted data items are included in Appendix 3. PSD and AS independently charted all sources. If DLB-specific data were unavailable within a broader Lewy body disease cohort, data for the overall group were charted.

### Collating, summarising and reporting the data

We used descriptive numerical analysis and basic qualitative content analysis [26]. Characteristics of the sources are summarised quantitatively. Personal (patient) and public involvement (PPI) was charted as either present or absent. Measures were categorised as either ‘multidimensional standardised measures,’ adhering to the APA’s definition of a standardised test as “an assessment instrument administered in a predetermined manner, such that the questions, conditions of administration, scoring, and interpretation of responses are consistent from one occasion to another” [30], or ‘multidimensional non-standardised measures,’ which included all novel measures/questions. Surveys were categorised according to their contents. If perspective data were derived from bespoke questions, the method was classified as a non-standardised measure. If data came solely from standardised instruments, it was classified as a standardised measure. Where both were used, the source was charted under both categories.

To develop topic categories, an inductive approach to basic qualitative content analysis was employed. The ‘data’ examined at this stage included each study’s aims and the methods used to capture perspective data. First, in line with established procedures for the organising phase of scoping reviews, PSD familiarised herself with all included sources. Second, open coding was conducted by PSD to identify and label specific topics on which perspectives were sought, integrating data from the two fields ‘aim of source’ and ‘methods used to capture perspectives’. For example, a qualitative study using telephone interviews with CPs to explore helpful aspects of care and unmet needs was coded as ‘helpful aspects of care’ and ‘unmet needs related to care’, and a trial of a pharmacological agent which included the NPI-D was coded as ‘CP distress.’ These codes functioned as descriptive labels reflecting the focus of the perspective data in each study. Efforts were made to apply codes consistently across the data; for example, references to orthostatic hypotension and incontinence were both coded as ‘autonomic symptoms’.

Third, PSD organised codes into initial topics (i.e. content categories) that shared a common theme. This marked the development of a coding framework designed to organise the studies according to the topic(s) of the perspectives explored. The following are examples of codes included within the category of ‘patient and CP perspectives related to supportive or additional symptoms’: ‘somatoform disorders’, ‘autonomic symptoms’, and ‘sexual dysfunction’. This was an iterative process, with groupings refined over time to ensure that each category accurately reflected a coherent thematic focus.

Lastly, topic categories identified in step three were grouped into broader, overarching topics, with the former functioning as sub-topics where appropriate. For example, sub-topics including ‘patient and CP perspectives related to core DLB symptoms’ and ‘patient and CP perspectives on suicidality’ were grouped under the broader category ‘perspectives related to the symptom and illness experience’.

The final framework of topics and sub-topics was reviewed by three authors (JK, NMcC, and PP). Relationships *between* categories were not analysed, as this review aimed to descriptively map the extent and nature of available evidence, rather than to develop a conceptual framework or theory, in accordance with JBI guidance [31].

Since each source could have multiple codes, each assigned to different categories, a source could fall across multiple categories. For example, a survey providing data on CP burden and care experiences could be charted under the topics “emotional and psychological well-being” and “experiences and needs related to care”.

To identify the source of perspective data, each included source was coded as reflecting the perspective of the person with DLB, the CP, or both. Proxy-reported data were coded as CP perspective. Where CPs were not the focus and provided only supplementary information when required, the perspective was coded as the patient since they were the focus.

Findings are presented in tables, figures, and narrative summaries. Consistent with scoping review guidelines, a critical appraisal of sources was not conducted [26].

## Results

The search yielded 4,064 sources. Additional scoping, including author contact, citation searching, and grey literature, retrieved 122 sources. After removing duplicates, 2,484 abstracts were screened, with 456 eligible for full-text review. Of these, 316 were excluded, resulting in 140 included sources (Fig. 1; see Additional file 1, Appendix 5, for a complete list of included sources).

### Publication trends

Sources demonstrated a clear increase over time, starting with a single source in 1998 and the highest number of publications recorded in 2021 (*n* = 19). A slight decline was observed in the years thereafter; however, this may reflect the timing of the search which did not capture publications after 27 September 2023 (Additional file 1, Appendix 4).

### Demographic characteristics and publication details

**Fig. 2:**
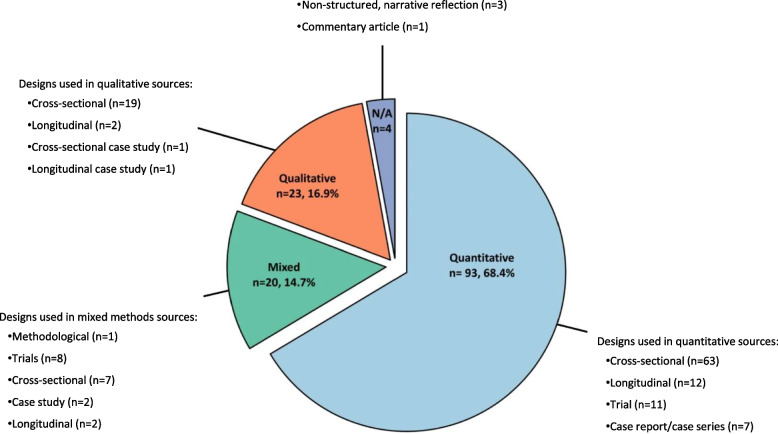
Research methodologies and study designs of included sources. Four sources were excluded from percentage counts because they were non-structured narrative reflections or commentary articles, with no applicable methodology or design [32–35]. N/A, not applicable

Characteristics of included sources are reported in Table 1 and Fig. 2 (further details in Appendices 5–8). Excluding non-structured narrative reflection articles and commentary articles (*n* = 4; 2.9%) [32–35], most research was quantitative (68.4%). Cross-sectional study designs were more common (65.4%). Sources focusing exclusively on DLB (excluding those with comparator groups, LBD or LBDis cohorts, and all-cause dementia cohorts) accounted for 43 sources (30.7%).

**Table 1:** Summary characteristics of included sources (*n* = 140)

**Characteristics (*n* = 140)**		**Count (%)**
**Geographic location**	Australia Brazil Canada China France India Israel Italy Norway Spain Sweden Switzerland Taiwan The Netherlands Turkey Japan The United States of America The United Kingdom	3 (2.1) 4 (2.9) 3 (2.1) 5 (3.6) 3 (2.1) 1 (0.7) 1 (0.7) 7 (5.0) 5 (3.6) 2 (1.4) 8 (5.7) 1 (0.7) 4 (2.9) 6 (4.3) 1 (0.7) 21 (15.0) 30 (21.4) 35 (25.0)
**Publication type**	Research article Case reports/case series Unstructured, narrative reflection Conference poster Thesis Programme Grants for Applied Research Report Brief report Commentary article	125 (89.3) 7 (5.0) 3 (2.1) 1 (0.7) 1 (0.7) 1 (0.7) 1 (0.7) 1 (0.7)
**Population of interest**	People with DLB (or LBD or LBDis) Care partners of people with DLB (or LBD or LBDis) only Both people with DLB (or LBD or LBDis) and care partners	62 (44.3) 47 (33.6) 31 (22.1)
**Number of DLB (or LBD) participants**	1–10 11–50 51–100 101–200 201–300 301–400 401–500 501–600 601–700 701–800 801–900 901 + Unclear Not applicable [32–35]	37 (26.4) 52 (37.1) 19 (13.6) 13 (9.3) 2 (1.4) 3 (2.1) 3 (2.1) 0 (0.0) 2 (1.4) 1 (0.7) 0 (0.0) 2 (1.4) 2 (1.4) 4 (2.9)
**Data on gender**	Reported data for DLB, LBD, or LBDis Not reported or no data for those with DLB, LBD, or LBDis Unclear (use of relational labels e.g., “wife”) Not applicable to report	110 (78.6) 24 (17.1) 2 (1.4) 4 (2.9)
**Data on ethnicity**	Reported data for DLB, LBD, or LBDis Not reported or no data for those with DLB, LBD, or LBDis Not applicable to report	23 (16.4) 113 (80.7) 4 (2.9)
**Data on age**	Reported data for DLB, LBD, or LBDis Not reported or no data for those with DLB, LBD, or LBDis Not applicable to report	109 (77.9) 27 (19.3) 4 (2.9)
**Data on years of education/** **educational attainment**	Reported data for DLB, LBD, or LBDis Not reported or no data for those with DLB, LBD, or LBDis Not applicable to report	64 (45.7) 72 (51.4) 4 (2.9)
**Data on socioeconomic status**	Reported data for DLB, LBD, or LBDis Not reported or no data for those with DLB, LBD, or LBDis Not applicable to report	3 (2.1) 133 (95.0) 4 (2.9)
**Care partner’s relationship to individual with DLB or LBD unspecified**	Spouse/Partner Child Sibling Formal care partner Child-in-law Friend Grandchild Niece Live-in divorcee Parent Sibling-in-law Not applicable Other or unspecified relationship (e.g., family member) Not reported or no data for those with DLB, LBD, or LBDis	60 (42.9) 40 (28.6) 13 (9.3) 9 (6.4) 8 (5.7) 6 (4.3) 6 (4.3) 3 (2.1) 2 (1.4) 1 (0.7) 1 (0.7) 12 (8.6) 24 (17.1) 59 (42.1)

In charting sample sizes, dyads were counted as single units. For charting whether a source provided data on gender, ethnicity, age, educational attainment, or socioeconomic status, a study was counted as reporting information if it provided data on either the individual with DLB (or LBD or LBDis) or the care partner. For care partner relationship, sources were charted in multiple categories. If a study was reported across multiple publications, it was charted for each source

*Abbreviations: DLB* Dementia with Lewy bodies, *LBD* Lewy body dementia, *LBDis* Lewy body disease

Diagnosis was clinician-confirmed in 106 of 136 cohorts (77.9%) and self-reported in 30 (22.1%), with some sources falling into multiple categories depending on recruitment methods (e.g., clinician confirmation in clinical settings and self-report in online recruitment avenues). Four sources were excluded from this analysis due to their publication type, which does not typically include diagnostic information (e.g., non-structured narrative reflections) [32–35].

### Methods of participant identification, sampling, and recruitment

Table 2 summarises recruitment and sampling approaches (Additional file 1, Appendix 9). Recruitment primarily occurred through healthcare and clinical settings (*n* = 104) and partner organisations (*n* = 32), including voluntary sector networks, dementia advocacy groups, research recruitment platforms, existing cohorts, and specialised recruitment and support organisations. The predominant recruitment mechanism was clinical engagement (*n* = 46). Recruitment mechanisms were not reported in 57 sources, and sampling methods were not reported in 87 sources.

**Table 2:** Sources of recruitment, mechanisms of recruitment, and sampling (*n* = 140)

Methods	Number of sources (%)	Examples
**Sources of recruitment**		
Healthcare and clinical settings	104 (74.3)	[36–49]
Partner organisations	32 (22.9)	[36, 50–57]
Support groups (online or in-person)	12 (8.6)	[46, 53, 58–60]
Social networks	4 (2.9)	[36, 61–63]
Aged care facilities	3 (2.1)	[59, 64, 65]
Continuation from preceding study	2 (1.4)	[66, 67]
Community meetings	1 (0.7)	[60]
Via former care partners	1 (0.7)	[68]
Healthcare intervention	1 (0.7)	[69]
Not applicable	9 (6.4)	[32–35, 70–74]
Unclear/not reported	5 (3.6)	[75–77]
**Mechanism of recruitment**		
Clinical engagement	46 (32.9)	[39, 40, 43, 48, 53, 57, 78–82]
Advertising	26 (18.6)	[36, 48, 62, 83, 84]
Email invite via partner organisations mailing list	13 (9.3)	[50, 54, 63, 85–88]
Research team invite via telephone, email and/or post	11 (7.9)	[37, 42, 51, 59, 68, 81]
Presentation by researcher	4 (2.9)	[36, 59, 65, 89]
Contacted via consent-to-contact database	3 (2.1)	[38, 49, 90]
Participant self-initiated response to invite within a survey	3 (2.1)	[91–93]
Invite via healthcare research entities and scientific societies	2 (1.4)	[94, 95]
Invitation via gatekeeper (unspecified)	1 (0.7)	[96]
Outreach by specialist recruitment agency	1 (0.7)	[61]
Direct messaging via research participation recruitment platform	1 (0.7)	[55]
Not applicable	9 (6.4)	[32–35, 70–74]
Unclear/not reported	57 (40.7)	[45, 47, 60, 97–103]
**Sampling methods**		
Consecutive recruitment	24 (17.1)	[49, 92, 104–107]
Purposive	12 (8.6)	[53, 61, 91, 96]
Convenience	10 (7.1)	[37, 51, 78, 108, 109]
Snowball	2 (1.4)	[51, 63]
Complete enumeration	1 (0.7)	[69]
Horizontal	1 (0.7)	[68]
Random	1 (0.7)	[97]
Not applicable	9 (6.4)	[32–35, 70–74]
Unclear/not reported	87 (62.1)	[38, 45, 110–112]

Sources using multiple strategies are charted in each category, and those reported in multiple publications are charted per source. For secondary analyses, we charted the original recruitment and sampling details. If sampling methods were not stated, it was charted as “unclear/not reported”

### Personal (patient) and public involvement

PPI was reported in 31 sources [36, 38, 39, 42, 43, 46, 50–52, 54, 56, 57, 59, 64, 67, 68, 82, 83, 86, 89–93, 95, 96, 108, 113–116], although this did not necessarily involve those affected by DLB or LBD. PPI references first appeared in 2016, peaking in 2021. However, this may reflect the timing of the search which did not capture publications after 2023 (Additional file 1, Appendix 10).

### Methods of capturing perspective data

Table 3 summarises the methods for capturing perspective data. Several methods were identified, with standardised measures most common, used in 86 sources (61.4%). Among the 63 standardised measures identified, versions of the Zarit Burden Interview (ZBI) were the most frequently cited (*n* = 45) [117–119]. Additional file 1, Appendix 11, details the frequency of use of the identified standardised measures, organised by the domains they assessed.

**Table 3:** Types of data collection methods used in included sources

Method of data collection	Number of sources (%)
Contextual inquiry sessions	1 (0.7)
Qualitative case study analysis	1 (0.7)
Semi-structured clinical interview	1 (0.7)
Think aloud interviews	1 (0.7)
Participant diaries	1 (0.7)
Interview (pre-existing guide)	1 (0.7)
Training evaluation forms	1 (0.7)
Therapy skills self-assessments	1 (0.7)
Observations	2 (1.5)
Informal feedback from family members	3 (2.2)
Repeated interviews (bespoke guide)	3 (2.2)
Interviews (no guide referenced)	3 (2.2)
Clinical interview with no structured assessment instruments	3 (2.2)
Clinical interview based on diagnostic and statistical manual	3 (2.2)
Focus group (bespoke guide)	5 (3.7)
Interview (bespoke guide)	22 (16.2)
Multidimensional non-standardised measures	33 (24.3)
Multidimensional standardised measures	86 (63.2)

Percentages are based on 136 sources; four sources were excluded because they were not research studies [32–35]. These comprised three non-structured narrative reflections (two authored pieces [33, 35], and one interview report [32]), and a commentary article that included an interview with a patient and spouse [34]. The number of methods exceeds the number of sources because many sources employed more than one data collection method and were therefore charted across multiple relevant categories. Where a source used multiple instruments of the same type (for example, several standardised measures, or a survey comprising multiple bespoke questions, i.e. non-standardised measures), the source was still counted only once as using that method

Terminology explained:

• Bespoke guide: Interview scripts developed specifically for the current study

• Pre-existing guide: Scripts originally designed for a previous study and reused in the current source

• Non-standardised multidimensional measure: Bespoke or novel questions designed for a study to assess a multidimensional concept (e.g. quality of life)

• Standardised multidimensional measure: Instruments administered in a predetermined manner, with consistent questions, administration, scoring, and interpretation (e.g. the Zarit Burden Interview)

The second most common method was non-standardised measures (*n* = 33; 24.3%). These appeared in various contexts, such as questionnaires or surveys (e.g., [50, 83, 85, 86, 120]) or novel scales (e.g., [121]). Interviews using bespoke guides were the most common qualitative method (*n* = 22; 16.2%). Less frequently used methods (all *n* = 1) included contextual inquiry sessions which examined health information behaviours [51]; qualitative case study analysis examining CP burden [69]; and think-aloud interviews capturing perspectives during instrument development [122]. Participant diaries, training evaluation forms, and therapy skills self-assessments were also used once, in combination, to capture perspectives related to an intervention [89].

### Research topic areas

Open coding generated 110 unique codes, organised into 34 topic categories, and then grouped into 13 overarching topics with sub-topics. Table 4 summarises the data collection methods used for each overarching topic, while Table 5 (a summary of Additional file 1, Appendix 12) provides a more detailed overview of specific methods used across all 13 topics and their sub-topics.

**Table 4:** Frequency of data collection methods used across 13 overarching topic categories

Overarching topics on which perspectives were sought (*n* = number of sources providing data related to the topic)	Method of data collection (*n* = number of times each method was used within a topic)													
	**Standardised measures**	**Non-standardised measures**	**Interviews** **(any type)**	**Focus groups**	**Observation**	**Qualitative case study analysis**	**Clinical interview (no structured instrument)**	**Clinical interview based on DSM manual**	**Training evaluation forms**	**Therapy skills self-assessments**	**Participant diaries**	**N/A**	**Contextual inquiry sessions**	**Informal comments recorded**
Perspectives related to research (*n* = 3)	0	**2**	**1**	0	0	0	0	0	0	0	0	0	0	0
End-of-life experiences (*n* = 4)	0	**1**	**3**	0	0	0	0	0	0	0	0	0	0	0
Resource use and costs (*n* = 4)	**2**	**2**	**1**	0	0	0	0	0	0	0	0	0	0	0
Perspectives on clinical and assessment instruments (*n* = 5)	0	0	**4**	0	0	0	0	0	0	0	0	0	0	**1**
Experience and impact of the COVID-19 pandemic and lockdown (*n* = 6)	**2**	**5**	0	0	0	0	0	0	0	0	0	0	0	0
Experiences and perspectives on non-pharmacological interventions (*n* = 12)	**3**	**2**	**6**	**3**	**1**	**1**	0	0	**1**	**1**	**1**	0	0	0
Social connection and engagement (*n* = 17)	**8**	**4**	**4**	0	0	0	0	0	0	0	0	**1**	0	0
Care partner physical and mental health (*n* = 18)	**15**	**3**	**1**	0	0	0	0	0	0	0	0	0	0	0
Self, identity, and coping (*n* = 19)	**8**	**3**	**7**	**2**	**1**	**1**	0	0	0	0	0	0	0	0
Experiences and needs related to care (*n* = 23)	**1**	**8**	**8**	**2**	**2**	**1**	0	0	0	0	0	**3**	**1**	**2**
Life satisfaction, QoL, and HRQoL (*n* = 25)	**25**	**1**	0	0	0	0	0	0	0	0	0	0	0	0
Perspectives related to the symptom and illness experience (*n* = 34)	**11**	**11**	**8**	0	0	0	**3**	**3**	0	0	0	**1**	0	0
Emotional and psychological well-being (*n* = 64)	**59**	**3**	**2**	**1**	**1**	**1**	0	0	0	0	0	0	0	0

If a source contributed to multiple sub-topics within a topic, each data collection method was recorded only once per topic. Where a source used multiple instruments of the same type (e.g. several standardised measures, or a survey comprising multiple novel questions, i.e. non-standardised measures), it was counted only once as using that method

*Abbreviations: DSM* Diagnostic and statistical manual, *QoL* Quality of life, *HRQoL* Health-related Quality of life, *NA* Not applicable, *COVID-19* Coronavirus disease 2019

**Table 5:** Summary of the methods used in each topic category

Topic category (*n* = sources)	Sub-topic (if applicable; *n* = sources)	Summary of methods used
**1. Perspectives related to research (*n* = 3)**—Captures patient and CP research priorities and perspectives related to the use of lumbar punctures in research	**-**	1. Interviews (*n* = 1) 2. Bespoke questions in a survey (*n* = 2)
**2. End-of-life experiences (*n* = 4)**—Captures CP experiences and reflections related to the end-of-life period	**-**	1. Bespoke questions in a survey (*n* = 1) 2. Interviews (*n* = 3)
**3. Resource use and costs (*n* = 4)**—Captures the use of health, social, and informal care services by patients and CPs, along with the associated financial costs and contributing factors	**-**	1. RUD-Lite (*n* = 1) 2. Interviews (*n* = 1) 3. CSRI (*n* = 1) 4. Bespoke questions (*n* = 2)
**4. Perspectives on clinical assessment instruments (*n* = 5)**—Captures views and reflections on the use, development, or acceptability of tools and measures used in clinical or research contexts	**-**	1. Think-aloud interviews (*n* = 1) 2. Dyadic interviews (*n* = 1) 3. Informal comments from CPs on suitability of a novel scale (*n* = 1) 4. Interviews during clinic visit (no guide referenced) (*n* = 2)
**5. Experience and impact of the COVID-19 pandemic and lockdown (*n* = 6)**—Captures the impact of the COVID-19 pandemic and related lockdown measures on patients and CPs. Two sub-topics were identified	**5.1** Impact of COVID-19 on patients and CPs (*n* = 5)	1. The ZBI (*n* = 1) 2. The GAD-7 (*n* = 1) 3. The PHQ-9 (*n* = 1) 4. Bespoke questions in a survey (*n* = 5)
	**5.2** Patient’s subjective experience of time (*n* = 1)	1. The STQ (*n* = 1)
**6. Experiences and perspectives on non-pharmacological interventions (*n* = 12)**—Captures patient and CP experiences of, and views related to, non-pharmacological interventions such as music therapy, cognitive rehabilitation, and psychosocial interventions	-	1. Longitudinal dyadic interviewing (*n* = 1) 2. Interviews (*n* = 1) 3. Observations (*n* = 1) 4. Qualitative case study analysis (*n* = 1) 5. The MiDAS (*n* = 1) 6. Training evaluation forms (*n* = 1) 7. Skills self-assessment (*n* = 1) 8. Therapy diaries (*n* = 1) 9. Bespoke semi-structured interview questionnaire (PPQ) (*n* = 1) 10. The BGSI (*n* = 2) 11. Bespoke questions (*n* = 2) 12. Focus groups (*n* = 3) 13. Post-intervention dyadic interviews (*n* = 3)
**7**. **Social connection and engagement (*n* = 17)**—Captures the social and relational experiences of patients and their CPs, including communication, connectedness, relationship quality, and expressions of engagement such as advocacy and campaigning. Four sub-topics were identified	**7.1** Patient and CP perspectives on communication difficulties and strategies (*n* = 2)	1. Interview based on the CAPPCI (*n* = 1) 2. Dyadic interviews (*n* = 1)
	**7.2** Patient experience of campaigning (*n* = 1)	1. Non-structured narrative reflection (*n* = 1)
	**7.3** Patient and CP perspectives on social support, isolation, and connectedness (*n* = 8)	1. The SSQ (*n* = 1) 2. The R-UCLA-LS/UCLA-LS (*n* = 2) 3. The MOS-SS (*n* = 2) 4. Bespoke questions in a web survey (*n* = 4)
	**7.4** Patient and CP perspectives on dyadic relationship quality (*n* = 6)	1. Longitudinal, narrative, dyadic interviews (*n* = 1) 2. Interviews (*n* = 2) 3. The RSS (*n* = 3) 4. The DRS (*n* = 3) 5. The FCR (*n* = 3)
**8. Care partner physical and mental health (*n* = 18)**—Captures the mental and physical health of CPs	**-**	1. Interviews (*n* = 1) 2. GAD-7 (*n* = 1) 3. GAD-9 (*n* = 1) 4. BDI-II (*n* = 1) 5. ISI (*n* = 1) 6. EQ-5D-3L (*n* = 1) 7. GDS-15 (*n* = 2) 8. PSQI (*n* = 2) 9. PHQ-9 (*n* = 3) 10. Bespoke questions (*n* = 3) 11. SF-12 (*n* = 3) 12. PHQ-2 (*n* = 4) 13. HADS (*n* = 6)
**9. Self, identity, and coping (*n* = 19)**—Captures how people with DLB and their CPs understand, adapt to, and cope with the condition. Three sub-topics were identified	**9.1** Patient and CP self-perception (*n* = 3)	1. Interviews (*n* = 1) 2. Narrative, longitudinal interviews (*n* = 1) 3. Longitudinal, narrative, dyadic interviews (*n* = 1)
	**9.2** Patient and CP self-efficacy, resilience, and coping (*n* = 16)	1. The PMS (*n* = 1) 2. The DAS (*n* = 1) 3. Observation (*n* = 1) 4. Qualitative case study analysis (*n* = 1) 5. The FCP-SEMD scale (*n* = 1) 6. Focus groups (*n* = 2) 7. The GSES (*n* = 2) 8. The BRS (*n* = 3) 9. The RSCSE (*n* = 3) 10. Bespoke questions (*n* = 3) 11. Interviews (*n* = 5)
	**9.3** Patient attitudes towards own ageing (*n* = 1)	1. The ATOA questionnaire (*n* = 1)
**10. Experiences and needs related to care (*n* = 23)**—Captures the care-related experiences, support needs, and treatment preferences of people with DLB and their CPs from the pre-diagnostic stage through to advanced stages of dementia (before end-of-life). Three sub-topics were identified	**10.1** Patient and CP needs and experiences of care, support, and treatment from pre-diagnosis to advanced stages (*n* = 21)	1. Contextual inquiry sessions (*n* = 1) 2. Commentary article reporting CP and patient perspective (*n* = 1) 3. Focus group (*n* = 1) 4. Qualitative case study analysis (*n* = 1) 5. Longitudinal, narrative, dyadic interviews (*n* = 1) 6. Non-structured narrative reflection (*n* = 2) 7. Informal family/CP comments reported (*n* = 2) 8. Observation (*n* = 2) 9. Interviews (*n* = 6) 10. Bespoke questions in a survey (*n* = 8)
	**10.2** Perspectives and experiences of formal care staff on managing LBD (*n* = 2)	1. Qualitative case study analysis (*n* = 1) 2. Bespoke questions (*n* = 1) 3. Observation (*n* = 1) 4. Paired interviews (*n* = 1) 5. Interview (*n* = 2) 6. Focus groups (*n* = 2)
	**10.3** CP desire to institutionalise (*n* = 1)	1. The DIS (*n* = 1)
**11. Life satisfaction, QoL, and HRQoL (*n* = 25)**—Captures QoL, HRQoL, and life satisfaction in patients and their CPs	**11.1** Patient’s QoL and HRQoL (*n* = 23)	1. The SF-8 (*n* = 1) 2. The ADRQL (*n* = 1) 3. The WHQOL-BREF (*n* = 1) 4. The LASA (*n* = 1) 5. The DEMQOL (*n* = 1) 6. The QOL-D (*n* = 1) 7. The EQ-5D-5L (*n* = 2) 8. The PDQ-8 (*n* = 1) 9. The PDQ-39 (*n* = 2) 10. The SF-12-v2 (*n* = 2) 11. The EQ-5D-3L (*n* = 5) 12. The QoL-AD (*n* = 9)
	**11.2** Care partner QoL and HRQoL (*n* = 11)	1. The SF-8 (*n* = 1) 2. Novel/bespoke scale (*n* = 1) 3. The LASA (*n* = 1) 4. The EQ-5D-5L (*n* = 1) 5. The WHOQOL-BREF (*n* = 2) 6. The QoL-AD (*n* = 2) 7. The EQ-5D-3L (*n* = 3)
	**11.3** Patient and CP life satisfaction (*n* = 1)	1. SwLS (*n* = 1)
**12. Perspectives related to the symptom and illness experience (*n* = 34)**—Captures patient and CP perspectives on the lived experience of DLB symptoms and illness across multiple domains. Eight sub-topics were identified	**12.1** Patient and CP perspectives related to core DLB symptoms (*n* = 8)	1. NEVHI interview (*n* = 2) 2. Clinical assessment of sleep satisfaction- no instrument referenced (*n* = 3) 3. Interviews (*n* = 3)
	**12.2** Patient and CP perspectives related to supportive or additional symptoms (*n* = 13)	1. The BDS (*n* = 1) 2. The SCOPA-Aut questionnaire (*n* = 1) 3. The NEO-FFI questionnaire (*n* = 1) 4. Clinical interview (no assessment instrument) (*n* = 2) 5. Interview based on DSM-IV-TR manual (*n* = 2) 6. The SCL-90-R (*n* = 2) 7. Bespoke questions in surveys/questionnaires (*n* = 3) 8. Interviews (*n* = 3)
	**12.3** Patient and CP perspectives on suicidality (*n* = 2)	1. Interview based on DSM-IV major depression criteria (*n* = 1) 2. The BDI-II (*n* = 1)
	**12.4** Patient experience of pain (*n* = 2)	1. Bespoke questionnaire (*n* = 1) 2. BPI (*n* = 1) 3. Clinical interview (*n* = 1)
	**12.5** Patient perspectives related to disability (*n* = 1)	1. The ‘Yesterday Interview’ (*n* = 1)
	**12.6** Patient general physical and mental health (*n* = 1)	1. EQ-5D-3L (*n* = 1)
	**12.7** Patient and CP narrative comments on the symptom and illness experience (*n* = 3)	1. Non-structured narrative reflection (*n* = 1) 2. Interviews (*n* = 2)
	**12.8** Patient and CP perspectives on symptom changes during lockdown (*n* = 4)	1. Bespoke questions in a survey (*n* = 4)
**13. Emotional and psychological well-being (*n* = 64)**—Captures the emotional and psychological well-being of both people with DLB and their CPs. Five sub-topics were identified	**13.1** CP burden, stress, and/or distress (*n* = 61)	1. Bespoke questions (*n* = 1) 2. Interviews (*n* = 1) 3. Focus groups (*n* = 1) 4. Observations (*n* = 1) 5. Qualitative case study analysis (*n* = 1) 6. PSS (*n* = 1) 7. Rel.SS (*n* = 10) 8. NPI-D (*n* = 13) 9. ZBI (*n* = 45)
	**13.2** CP grief (*n* = 4)	1. PG-12 (*n* = 1) 2. Bespoke questions (*n* = 1) 3. MM-CGI-SF (*n* = 3)
	**13.3** Patient and CP psychological well-being (*n* = 7)	1. Bespoke questions in a survey (*n* = 1) 2. Interviews (*n* = 1) 3. WHO-5 (*n* = 1) 4. PCI (*n* = 1) 5. C-PWBS (*n* = 4)
	**13.4** Patient positive affect (*n* = 1)	1. QOL-D (*n* = 1)
	**13.5** Patient empathy (*n* = 1)	1. IRI (*n* = 1)

This is a summarised version of Appendix 12; Some sources employed more than one method to collect data within a given topic category. Therefore, the total number of methods listed may exceed the number of sources addressing that topic

*Abbreviations:* Methods of data collection: *CAPPCI* Conversation Analysis Profile for People with Cognitive Impairment, *DSM* Diagnostic and statistical manual, *RUD-Lite* Resource Utilization in Dementia—Lite Version, *BPI* Brief Pain Questionnaire, *BDS* Blessed Dementia Scale, *MiDAS* The Music in Dementia Assessment Scale, *CSRI* Client Services Receipt Inventory, *SCOPA-Aut* Scales for Outcomes in PD-Autonomic, *SwLS* Satisfaction with Life Scale, *WHO-5* The WHO-5 Well-being Index, *DAS* Dementia Attitudes Scale, *PMS* Pearlin Mastery Scale, *UCLA-LS* UCLA Loneliness Scale, *R-UCLA-LS* Revised UCLA Loneliness Scale, *PG-12* The Prolonged Grief-12, *FCP-SEMD Scale* Family Care Partner Self-Efficacy for Managing Dementia Scale, *GDS-15* Geriatric Depression Scale, *PSS* Perceived Stress Scale, *C-PWBS* Condensed version of the Psychological Well-Being Scale, *IRI* The Interpersonal Reactivity Index, *PDQ‐8* Parkinson’s Disease Questionnaire-8, *LASA* Linear Analogue Self-Assessment Scale, *ADRQL* The Alzheimer Disease-related Quality of Life, *DEMQOL* Dementia Quality of Life Measure, *ED5D3L* Euroqol Questionnaire‐short version, *DIS* The Desire to Institutionalize Scale, *ISI* Insomnia Severity Index, *SSQ* Social Support Questionnaire, *STQ* Subjective Time Questionnaire, *ATOA* The Attitude Toward Own Aging questionnaire, *NEO-FFI* NEO Five Factor Inventory, *MOS-SS* The Medical Outcome Study Social Support, *PCI* Perceived Change Index, *PDQ-39* 39-item Parkinson’s Disease Questionnaire, *BDI-II* Beck Depression Inventory-II, *QOL-D* Quality of life questionnaire for dementia, *GAD-7* General Anxiety Disorder-7, *PSQI* The Pittsburgh Sleep Quality Index, *SF-8* Short-Form-8, *WHOQOL-BREF* World Health Organization Quality of Life-Brief Version, *GSES* Generalized Self-Efficacy Scale, *BGSI* The Bangor Goal Setting Interview, *PPQ* The Program Participation Questionnaire, *EQ-5D-5L* Euro-Qol 5 dimensions 5-level, *NEVHI* The North-East Visual Hallucination Interview, *SF-12-v2* Short Form Health Survey, version 2, *SCL-90-R* Symptom Checklist 90R, *MM-CGI-SF* The Marwit-Meuser Caregiver Grief Inventory Short Form, *PHQ-9* Patient Health Questionnaire-9, *J-ZBI_8* 8-item short version of the Japanese Zarit Burden Interview, *RSCSE* Revised Scale for Caregiving Self-Efficacy, *RSS* The Relationship Satisfaction Scale, *SF-12* The 12-item short-form health survey, *DRS* The Dyadic Relationship Scale, *FCR* The Family Caregiving Role Scale, *BRS* The Brief Resilience Scale, *PHQ-2* Patient Health Questionnaire-2, *HADS* Hospital Anxiety and Depression Scale, *ZBI-12* 12-item Zarit Burden Interview, *EQ-5D* EuroQol- 5 Dimension, *QoL-AD* Quality of Life in Alzheimer’s Disease, *Rel.SS* Relative’s Stress Scale, *NPI-D* Neuropsychiatric Inventory carer distress scale, *ZBI* The Zarit Burden Interview

Other: *CP* Care partner, *HRQoL* Health-related quality of life, *QoL* Quality of life, *COVID-19* Coronavirus disease 2019

The most frequently investigated topic was ‘emotional and psychological well-being’, with 64 sources providing data. Five sub-topics were identified (Table 5). Versions of the ZBI [117] and the NPI-D [29] were the most commonly used methods for capturing data on the most frequently identified sub-topic, ‘CP burden, stress, and/or distress’. In comparison, substantially fewer sources addressed ‘perspectives related to the symptom and illness experience’, for which 34 sources provided data. Eight sub-topics were identified (Table 5), the most common being ‘patient and CP perspectives related to supportive or additional symptoms’ (*n* = 13), followed by ‘patient and CP perspectives related to core DLB symptoms’ (*n* = 8). Within this overarching topic, standardised and non-standardised measures were equally common (*n* = 11; Table 4).

### Topics receiving disproportionate attention

Each of the following sub-topics was investigated in only a single source: ‘patient’s subjective experience of time’, ‘patient experience of campaigning’, ‘patient attitudes towards own ageing’, ‘CP desire to institutionalise’, ‘patient and CP life satisfaction’, ‘patient perspectives related to disability’, ‘patient general physical and mental health’, ‘patient positive affect’, and ‘patient empathy’ (Table 5).

### Source of perspective data

Table 6 summarises the source of perspective data across the 13 overarching topics, demonstrating the predominance of CP perspectives.

**Table 6:** Number of sources in each overarching topic category capturing patient, care partner, or both patient and care partner perspectives

Topic category	Total number of sources	Patient perspective only (*n* =)	Care partner perspective only (*n* =)	Both patient & care partner perspectives (*n* =)
Perspectives related to research	3	0	0	3
End-of-life experiences	4	0	4	0
Resource use and costs	4	1	1	2
Perspectives on clinical assessment instruments	5	0	2	3
Experience and impact of the COVID-19 pandemic and lockdown	6	1	3	2
Experiences and perspectives on non-pharmacological interventions	12	2	6	4
Social connection and engagement	17	2	13	2
Care partner physical and mental health	18	0	18	0
Self, identity, and coping	19	2	11	6
Experiences and needs related to care	23	2	12	9
Life satisfaction, QoL, and HRQoL	25	6	6 ^**a**^	13^**b**^
Perspectives related to the symptom and illness experience	34	15	12	7
Emotional and psychological well-being	64	1	60^**c**^	3

^**a**^ In one source, CPs reported only on the patient’s QoL, not their own [123]. In three other sources, CPs reported on both their own and the patient’s QoL [54, 121, 124]

^**b**^ In seven of the thirteen sources, CP data reflected the patient’s QoL or HRQoL rather than their own experience [41, 78, 125–129]. In four sources, patient QoL was either reported by both the patient and a proxy, or the study allowed for proxy reporting where needed [41, 43, 99, 130]

^**c**^ Two of the sixty sources captured CP perspectives on patient-relevant data, i.e. patient affect [131] and patient psychological well-being [52]

## Discussion

This review examines the extent and nature of published work on the perspectives of individuals with DLB and their CPs, contributing to the evidence base in two key ways. First, it focuses specifically on DLB, where perspectives are under-represented in all-cause dementia or aggregated LBD cohorts. Second, it identifies critical gaps in both knowledge and methodology. By mapping the topics addressed, the distribution of research attention, and the methods used, the review highlights areas where patient and CP perspectives remain under-represented. These findings can guide topic selection and methodological choices in future research.

Although a range of methods was identified, standardised measures, which offer structured assessment and facilitate comparisons across dementia groups, dominate the literature. Their specificity is valuable when measuring clearly defined aspects of experience. For example, the ZBI provides a focused measure of CP burden and has been used in DLB studies to identify contributing factors [132]. However, reliance on standardised tools limits the depth and breadth of perspective data captured. For instance, generic measures such as the EQ-5D inadequately address aspects of QoL important to people affected by dementia [133]. Many outcome measures also rely on proxy reports, which often demonstrate poor agreement with self-reports, particularly in QoL measurements [134, 135].

The reliability and validity of selected instruments are critical for ensuring credible findings, but many were initially developed for AD or PD and are often not validated in DLB [136, 137]. Vatter and colleagues [116] provide preliminary psychometric evidence for various CP-reported measures in Parkinson’s-related dementias. However, their findings may not fully capture DLB-specific nuances. Furthermore, variability in measures indicates a lack of consensus on the most appropriate tools, necessitating synthesis and evaluation of existing measures and the development of DLB-specific instruments.

Together, these challenges suggest that while existing standardised measures are valuable, they may be inadequate for capturing the nuances of perspectives in DLB, limiting the extent to which current evidence can meaningfully inform the development of person-centred care strategies. There is a need for the development of standardised, validated instruments specific to DLB. Additionally, bespoke measures may be appropriate in certain contexts, such as eliciting treatment preferences. These approaches can help explore perspectives that existing standardised tools may overlook, particularly in domains beyond their scope or where validation in DLB is lacking.

Similarly to Bentley et al., [14] we observed that the evidence base is largely quantitative, perhaps reflecting a broader trend in academic publishing, where qualitative research is undervalued [138]. Some quantitative methods, such as online surveys, enable efficient data collection from geographically diverse populations. In contrast, historical caution regarding remote qualitative data collection may have encouraged use of quantitative approaches [139, 140]. Qualitative studies are also less commonly bolted on to clinical trials or imaging studies. Furthermore, qualitative methods face debates over quality assessment and challenges related to reliance on abstraction, recall, and verbal reporting [22, 141].

However, qualitative approaches offer alternative or complementary means of exploring perspectives. We observed limited diversity in the qualitative methods employed, particularly when compared with two scoping reviews that identified innovative methods for empowering individuals with dementia to contribute to research [142, 143]. Qualitative methods, whether used alone or in combination, generate deeper understanding of perspectives and support theory development, particularly through approaches such as grounded theory [144]. Qualitative or mixed methods investigations can also be guided by existing theoretical frameworks, which provide structure when working with complex perspective data and support theoretically grounded insights that enhance credibility and transferability to practice settings [145]. These points highlight the value of qualitative approaches for exploring perspectives data and suggest opportunities for broader use in DLB.

Discrete choice experiments (DCEs) are a stated preference method increasingly used to measure patient preferences [146]. Although applied in other dementia subtypes [147–149], our search found no studies using DCEs in the context of DLB. DCEs quantify the relative importance of, and willingness to trade between, attributes of health-related states, goods, and services, and could offer valuable insights into preferences for managing trade-offs in DLB symptoms. Additionally, DCEs may represent more high-salience tasks which could better engage individuals and potentially mitigate cognitive fluctuations.

We observed a predominance of cross-sectional designs in this review, limiting understanding of how perspectives evolve across the disease course. Understanding these changes could support advance care planning by identifying how concerns, needs, and experiences evolve. This predominance may reflect recruitment challenges in longitudinal studies. Recruitment challenges in DLB are recognised and likely exacerbated by poor detection and low prevalence rates [150, 151]. While healthcare settings can provide direct access to the target population, they may introduce biases, including the under-representation of minority ethnic groups who present later to dementia services [152]. Additionally, various factors can influence clinical ‘gatekeepers’ in their decision to approach potential participants, potentially introducing biases [153, 154]. Many sources provided insufficient detail on recruitment and sampling strategies, limiting our ability to assess potential biases and their implications. Given the importance of effective recruitment for research validity and reliability, specific methodological consideration should focus on ensuring better mechanisms are in place to facilitate participant engagement.

Regarding participant characterisation, potential confusion surrounding LBD terminology and the relationship between DLB, LBD, and PDD increases the risk of inaccurate self-reported diagnoses. Ethnicity, educational attainment, and socioeconomic status were notably underreported. This has important implications for future research aiming to systematically assess representation in DLB research. The use of relational labels inadequately represented gender, posing methodological challenges for future research systematically exploring sex and gender in DLB. Moreover, the exclusion of relational labels in the charting of gender in this study illustrates how failing to acknowledge same-sex couples can impact research outcomes.

We identified 13 overarching topics, reflecting the breadth of work exploring the perspectives of those affected by DLB. The most widely investigated topic was ‘emotional and psychological well-being’, with the sub-topic ‘CP burden, stress, and/or distress’ examined in 61 sources. This echoes findings by Bentley et al. [14], who observed that existing evidence on the experiences of those affected by LBD is largely focused on CP burden, unmet need, and QoL.

However, we revealed disproportionate attention across topics and sub-topics, with several addressed in three or fewer sources (Tables 4 and 5). Several research priorities previously identified by people with DLB and their CPs, including reducing hospitalisation, disparities, and improving education, were either absent or under-represented in this review [38, 86]. These knowledge gaps weaken the evidence base needed to inform care strategies that improve well-being and quality of life, and to guide service design and delivery that is responsive to those affected*.* The recently developed DLB Core Outcome Set may offer a useful framework for assessing the relevance of these gaps, helping to distinguish between the absence of core outcomes and the underrepresentation of more nuanced or context-specific constructs. Future research should involve those affected by DLB in research priority-setting exercises to develop a strategy that addresses DLB-specific priorities.

Finally, consistent with Bentley et al. [14], most sources in this review captured CP perspectives. As preferences can differ between patients and CPs [155, 156], researchers should consider how methodological approaches can be adapted to better support participation from individuals with DLB, ensuring their voices inform future research and care strategies.

### Strengths and limitations

A strength of this study is its thorough and broad search and adherence to best practice guidelines [25–27]. However, limitations exist. First, data from individuals with LBD or LBDis were included when DLB-specific data were unavailable. Second, focusing on a source’s aims and methods to identify topics may have led to references in other categories being overlooked. However, as these references were not central to the sources’ aims, their omission is unlikely to affect overall conclusions. Unidimensional measures were excluded to maintain a manageable scope and to focus on measures that capture broader aspects of lived experience. Excluding non-English publications may have resulted in missing relevant sources. Lastly, this study did not include PPI, which might have offered additional insight into the findings from the perspective of those affected by DLB.

## Conclusion

The growing number of sources over time reflects increasing recognition of the importance of capturing the perspectives of those affected by DLB. Although various methods have been used, the evidence base is dominated by standardised measures, which, while valuable, are not always suited to capturing the complexity and nuances of perspectives in DLB. We propose that novel methods facilitating systematic data collection should be explored, particularly those capable of addressing relatively under-represented topics.

## Supplementary Information

Additional file 1. Research on the perspectives of people affected by dementia with Lewy bodies: a scoping review. This supplementary material provides additional extracted and synthesised data from all sources included in the scoping review. It details methodological aspects, study characteristics, and key findings. A full list of appendices is provided on page 1.

## Data Availability

All data generated or analysed during this study are included in this published article [and its supplementary information files].

## Abbreviations

LBD: Lewy body dementia
DLB: Dementia with Lewy bodies
PDD: Parkinson’s disease dementia
PD: Parkinson’s disease
CP: Care partner
DCE: Discrete choice experiment
NDLTD: The Networked Digital Library of Theses and Dissertations
PRISMA-ScR: Preferred Reporting Items for Systematic Reviews and Meta-Analyses Extension for Scoping Reviews
NPI-D: Neuropsychiatric Inventory Caregiver Distress subscale
PPI: Personal (patient) and public involvement
PD-MCI: Mild cognitive impairment in Parkinson’s disease
ZBI: Zarit burden interview
QoL: Quality of life
LBDis: Lewy body disease
