# Supplementary material for: Research on the perspectives of people affected by dementia with Lewy bodies: a scoping review
Source: Alzheimers Res Ther. 2025 May 26;17:117. doi: 10.1186/s13195-025-01760-4 (PMC12105336; doi:10.1186/s13195-025-01760-4)
Supplement: Supplementary file 1 — Additional file 1. Research on the perspectives of people affected by dementia with Lewy bodies: a scoping review. This supplementary material provides additional extracted and synthesised data from all sources included in the scoping review. It details methodological aspects, study characteristics, and key findings. A full list of appendices is provided on page 1. [file 13195_2025_1760_MOESM1_ESM.docx]

**Supplementary material**

**Title:** Research on the perspectives of people affected by dementia with Lewy bodies: a scoping review

**Table of contents:**

| **Appendices** | **Item** | **Pages** |
| --- | --- | --- |
| **Appendix 1** | Preferred Reporting Items for Systematic reviews and Meta-Analyses extension for Scoping Reviews (PRISMA-ScR) Checklist | 2-3 |
| **Appendix 2** | Search strings | 4-6 |
| **Appendix 3** | Modifications to the provisional charting template and final data charting template | 7-9 |
| **Appendix 4** | Fig 1. Yearly publications of sources providing perspective data from 1998 to 2024. | 10 |
| **Appendix 5** | Publication characteristics and demographic reporting | 11-60 |
| **Appendix 6** | Charted data on care partner relationships | 61-71 |
| **Appendix 7** | Methods of determining diagnosis | 72-81 |
| **Appendix 8** | Study designs and methods | 82-111 |
| **Appendix 9** | Methods of recruitment and sampling | 112-136 |
| **Appendix 10** | Personal and public involvement   1. Personal and public involvement in the included sources 2. Trend in personal and public Involvement in the included sources | 137-142 |
| **Appendix 11** | The frequency of use of reported standardised measures, organised by their assessed domains | 143-144 |
| **Appendix 12** | Table: Overview of topic categories, aims of sources and data collection methods | 145-182 |

**Appendix 1.** Preferred Reporting Items for Systematic reviews and Meta-Analyses extension for Scoping Reviews (PRISMA-ScR) Checklist

| **SECTION** | **ITEM** | **PRISMA-ScR CHECKLIST ITEM** | **REPORTED ON PAGE #** |
| --- | --- | --- | --- |
| **TITLE** | | | |
| Title | 1 | Identify the report as a scoping review. | 2 |
| **ABSTRACT** | | | |
| Structured summary | 2 | Provide a structured summary that includes (as applicable): background, objectives, eligibility criteria, sources of evidence, charting methods, results, and conclusions that relate to the review questions and objectives. | 2 |
| **INTRODUCTION** | | | |
| Rationale | 3 | Describe the rationale for the review in the context of what is already known. Explain why the review questions/objectives lend themselves to a scoping review approach. | 3-4 |
| Objectives | 4 | Provide an explicit statement of the questions and objectives being addressed with reference to their key elements (e.g., population or participants, concepts, and context) or other relevant key elements used to conceptualize the review questions and/or objectives. | 4 |
| **METHODS** | | | |
| Protocol and registration | 5 | Indicate whether a review protocol exists; state if and where it can be accessed (e.g., a Web address); and if available, provide registration information, including the registration number. | 4 |
| Eligibility criteria | 6 | Specify characteristics of the sources of evidence used as eligibility criteria (e.g., years considered, language, and publication status), and provide a rationale. | 5-6 |
| Information sources* | 7 | Describe all information sources in the search (e.g., databases with dates of coverage and contact with authors to identify additional sources), as well as the date the most recent search was executed. | 4 |
| Search | 8 | Present the full electronic search strategy for at least 1 database, including any limits used, such that it could be repeated. | Appendix 2 |
| Selection of sources of evidence† | 9 | State the process for selecting sources of evidence (i.e., screening and eligibility) included in the scoping review. | 6-7 and Figure 1 |
| Data charting process‡ | 10 | Describe the methods of charting data from the included sources of evidence (e.g., calibrated forms or forms that have been tested by the team before their use, and whether data charting was done independently or in duplicate) and any processes for obtaining and confirming data from investigators. | 7 |
| Data items | 11 | List and define all variables for which data were sought and any assumptions and simplifications made. | Appendix 3 |
| Critical appraisal of individual sources of evidence§ | 12 | If done, provide a rationale for conducting a critical appraisal of included sources of evidence; describe the methods used and how this information was used in any data synthesis (if appropriate). | N/A |
| Synthesis of results | 13 | Describe the methods of handling and summarizing the data that were charted. | 7-9 |
| **RESULTS** | | | |
| Selection of sources of evidence | 14 | Give numbers of sources of evidence screened, assessed for eligibility, and included in the review, with reasons for exclusions at each stage, ideally using a flow diagram. | 9 and Figure 1 |
| Characteristics of sources of evidence | 15 | For each source of evidence, present characteristics for which data were charted and provide the citations. | Appendices 5-10 |
| Critical appraisal within sources of evidence | 16 | If done, present data on critical appraisal of included sources of evidence (see item 12). | N/A |
| Results of individual sources of evidence | 17 | For each included source of evidence, present the relevant data that were charted that relate to the review questions and objectives. | Appendices 5-10 and 12 |
| Synthesis of results | 18 | Summarize and/or present the charting results as they relate to the review questions and objectives. | 9-12, Figure 2, Tables 1-6; Appendices 4, 10-12 |
| **DISCUSSION** | | | |
| Summary of evidence | 19 | Summarize the main results (including an overview of concepts, themes, and types of evidence available), link to the review questions and objectives, and consider the relevance to key groups. | 12-16 |
| Limitations | 20 | Discuss the limitations of the scoping review process. | 16 |
| Conclusions | 21 | Provide a general interpretation of the results with respect to the review questions and objectives, as well as potential implications and/or next steps. | 16 |
| **FUNDING** | | | |
| Funding | 22 | Describe sources of funding for the included sources of evidence, as well as sources of funding for the scoping review. Describe the role of the funders of the scoping review. | See section on funding |

JBI = Joanna Briggs Institute; PRISMA-ScR = Preferred Reporting Items for Systematic reviews and Meta-Analyses extension for Scoping Reviews.

* Where *sources of evidence* (see second footnote) are compiled from, such as bibliographic databases, social media platforms, and Web sites.

† A more inclusive/heterogeneous term used to account for the different types of evidence or data sources (e.g., quantitative and/or qualitative research, expert opinion, and policy documents) that may be eligible in a scoping review as opposed to only studies. This is not to be confused with *information sources* (see first footnote).

‡ The frameworks by Arksey and O’Malley (6) and Levac and colleagues (7) and the JBI guidance (4, 5) refer to the process of data extraction in a scoping review as data charting*.*

§The process of systematically examining research evidence to assess its validity, results, and relevance before using it to inform a decision. This term is used for items 12 and 19 instead of "risk of bias" (which is more applicable to systematic reviews of interventions) to include and acknowledge the various sources of evidence that may be used in a scoping review (e.g., quantitative and/or qualitative research, expert opinion, and policy document).


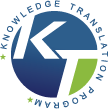


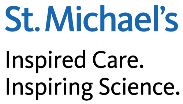
 *From:* Tricco AC, Lillie E, Zarin W, O'Brien KK, Colquhoun H, Levac D, et al. PRISMA Extension for Scoping Reviews (PRISMAScR): Checklist and Explanation. Ann Intern Med. 2018;169:467–473. [doi: 10.7326/M18-0850](http://annals.org/aim/fullarticle/2700389/prisma-extension-scoping-reviews-prisma-scr-checklist-explanation).

**Appendix 2.** Search strings

**PubMed**

(dementia-with-lewy-bodies or lewy-body-dementia or lewy-body-disease or dlb or lbd or lewy-bod*) AND (qualitative or interview* or focus-group* or narrative-inquir* or ethnograph* or personal-narrative* or life-histor* or autobiograph* or blog* or survey* or questionnaire* or discrete-choice or dce). Filters: English.

**Web of Science Core Collection**

Search #1: “dementia with lewy bodies” OR “lewy body dementia” OR “lewy body disease” OR dlb OR lbd OR “lewy bod*”

Search #2: interview* OR “focus group*” OR “narrative inquir*” OR ethnograph* OR “personal narrative*” OR “life histor*” OR autobiograph* OR blog* OR qualitative OR survey* OR questionnaire* OR “discrete choice experiment*” OR “discrete choice survey*” or dce

Search #3: #1 AND #2

Search #4: #1 AND #2 and English (Languages)

**Ovid Medline All**

1. dementia with lewy bodies.mp.

2. lewy body dementia.mp.

3. lewy body disease.mp.

4. dlb.mp.

5. lbd.mp.

6. lewy bod*.mp. or exp Lewy Bodies/

7. Lewy Body Disease/

8. 1 or 2 or 3 or 4 or 5 or 6 or 7

9. survey*.mp.

10. questionnaire*.mp.

11. discrete choice experiment*.mp.

12. discrete choice survey*.mp.

13. dce.mp.

14. “Surveys and Questionnaires”/

15. 9 or 10 or 11 or 12 or 13 or 14 or 15

16. exp Interview/ or interview*.mp.

17. focus group*.mp. or exp Focus Groups/

18. qualitative research/

19. narrative inquir*.mp.

20. ethnograph*.mp.

21. personal narrative*.mp. or exp Personal Narrative/

22. life histor*.mp.

23. autobiograph*.mp. or exp Autobiography/

24. exp Blogging/ or blog*.mp.

25. 16 or 17 or 18 or 19 or 20 or 21 or 22 or 23 or 24

26. 15 or 25

27. 8 and 26

28. limit 27 to english language

**EMBASE**

1. lewy body dementia.mp.

2. lewy body disease.mp.

3. dlb.mp.

4. lbd.mp.

5. dementia with lewy bodies.mp.

6. exp diffuse Lewy body disease/

7. lewy bod*.mp. or exp Lewy body/

8. 1 or 2 or 3 or 4 or 5 or 6 or 7

9. exp health survey/ or survey*.mp. or exp health care survey/

10. questionnaire*.mp. or exp questionnaire/

11. discrete choice experiment*.mp.

12. discrete choice survey*.mp.

13. dce.mp.

14. 9 or 10 or 11 or 12 or 13

15. qualitative.mp.

16. exp telephone interview/ or exp semi structured interview/ or exp video interview/ or interview*.mp. or exp unstructured interview/ or exp audio interview/ or exp interview/ or exp structured interview

17. focus group*.mp.

18. exp qualitative research/ or exp narrative/ or narrative inquir*.mp.

19. ethnograph*.mp. or exp ethnography/

20. personal narrative*.mp.

21. life histor*.mp.

22. autobiograph*.mp.

23. blog*.mp. or blogging/

24. 15 or 16 or 17 or 18 or 19 or 20 or 21 or 22 or 23

25. 14 or 24

26. 8 and 25

27. limit 26 to english language

**PsycINFO**

1. dementia with lewy bodies.mp. or exp Dementia with Lewy bodies/

2. lewy body dementia.mp.

3. lewy body disease.mp.

4.dlb.mp.

5. lbd.mp.

6. lewy bod*.mp.

7. 1 or 2 or 3 or 4 or 5 or 6

8. exp Mail Surveys/ or exp Telephone Surveys/ or exp Surveys/ or exp Online Surveys/ or survey*.mp.

9. questionnaire*.mp. or exp Questionnaires/

10. discrete choice experiment*.mp.

11. discrete choice survey*.mp.

12. dce.mp.

13. 8 or 9 or 10 or 11 or 12

14. exp Interviews/ or interview*.mp.

15. exp Focus Group/ or focus group*.mp.

16. narrative inquir*.mp. or exp Narrative Analysis/

17. ethnograph*.mp. or exp Ethnography/

18. personal narrative*.mp.

19. life histor*.mp.

20. autobiograph*.mp. or exp Authobiography/

21. blog*.mp. or exp Blog/

22. exp Qualitative Methods/ or qualitative.mp.

23. 14 or 15 or 16 or 17 or 18 or 19 or 20 or 21 or 22

24. 13 or 23

25. 7 and 24.

26. limit 25 to english language

**CINAHL complete**

Search #1: "dementia with lewy bodies" OR "lewy body dementia" OR "lewy body disease" OR lbd OR dlb OR "lewy bod*"

Search #2: "survey* OR questionnaire* OR "discrete choice experiment*" OR "discrete choice survey*" OR dce" OR (MH "Survey Research") OR (MH "Structured Questionnaires") OR (MH "Surveys")

Search #3: (MH "Blogs") OR (MH "Narratives") OR (MH "Focus Groups") OR "qualitative OR interview* OR "focus group*" OR "narrative inquir*" OR ethnograph* OR "personal narrative*" OR "life histor*" OR autobiograph* OR blog*"

Search #4: Search2 OR Search3

Search #5: Search1 AND Search4

Search #6: S1 AND S4 (Narrow by language: english)

**Networked Digital Library of Theses and Dissertations: Global ETD Search**

Search term: dementia with Lewy bodies

**Google Scholar**

((dementia with lewy bodies) OR (lewy body dementia) OR (lewy body disease)) AND ((qualitative) OR (interview) OR (focus group) OR (survey) OR (questionnaire))

**Appendix 3:** Modifications to the provisional charting template and final data charting template

**Modifications:**

The provisional charting template was piloted independently on ten sources by two reviewers. Following piloting, the reviewers discussed aspects of the tool that required refinement, which led to several modifications. First, a section was added to extract data on participant gender and ethnicity to determine whether sources capture these demographic aspects. A new section was also added to identify scales and instruments specifically capturing perspectives, enabling differentiation from those that did not meet eligibility criteria. The section on outcomes of interest and study outcome measures was removed due to overlap with the sections on methods and focus area. The section on author recommendations was removed because, upon further consideration, it was deemed beyond the scope of the current review. Lastly, a new section on personal and public involvement was included to reflect its importance in research design. Extracted data on key findings, methodological challenges, and methods for assessing disease severity will be reported separately from this current report.

**Final data charting template**

The final data charting template captured information on publication details, study and participant information, measures used, survey details, qualitative methods and personal (patient) and public involvement (PPI):

| **Review Title: Capturing the perspectives of people with dementia with Lewy bodies and their care partners: a scoping review** | |
| --- | --- |
| **Data extraction tool** | |
| 1. Publication details:  - Title - Author(s) - Date of publication - Country/Region - Publication type |  |
| 1. Topic/Focus area |  |
| 1. Article details  - Aim(s) - Context (year and location) - Population (age, patient or caregiver, study subject population) - Eligibility criteria - Sample size - Source of recruitment - Mechanism of recruitment - Mechanism of sampling - Design - Methodology - Methods |  |
| 1. Participant details  - n (number of participants for each subgroup if applicable) - Sex/gender - Ethnicity - Mean age - Participant(s) country of origin - Socioeconomic status of participant(s) - Participant(s) mean years of education - Patient’s stage in the disease course (mild, moderate, severe) |  |
| 1. Care partner’s relationship to individual with DLB (Formal or informal care); Spouse (husband or wife); Child (daughter or son); Relative |  |
| 1. Were data on symptoms collected?   (e.g., by measurement, self-report or case note review) |  |
| 1. Who determined diagnosis? (e.g., self-reported or clinician-judged) |  |
| 1. How was disease severity measured (e.g., clinician judged or Clinical Dementia Rating Scale) |  |
| 1. Scales used:  - Cognitive scales used - Functional scales used - Quality of life scales used - Other scales used |  |
| 1. Scales and instruments capturing perspective (and scores) |  |
| 1. In relation to qualitative methods:  - Method - Format (telephone, face-to-face, zoom) - Design - Relationship between researcher and participant - Method of qualitative analysis - Duration of time (minutes) - If applicable, how were dyadic interviews managed |  |
| 1. Source of perspective |  |
| 1. Key findings related to patients’ and caregivers’ views |  |
| 1. Any reported challenges with using the chosen methodology with the sample |  |
| 1. Was there patient and public involvement |  |

**Appendix 4**: Yearly publications of sources providing perspective data from 1998 to 2024.

**Fig. 1** Yearly publications of sources providing perspective data from 1998 to 2024. The search was completed in September 2023. As a result, the 2023 and 2024 columns do not represent all works published during this period.

**Appendix 5:** Publication characteristics and demographic reporting

| **Title and authors** | **Publication details**  • Date of publication  • Country/Region  • Publication type  • Context | **Aims** | **Population** | **Sample size**  **(DLB or LBD)** | **Participant Characteristics Reported (Yes/Not stated or no relevant data)** |
| --- | --- | --- | --- | --- | --- |
| Research priorities of caregivers and individuals with dementia with Lewy bodies: an interview study [1]  **Author(s):** Melissa J Armstrong, Noheli Gamez, Slande Alliance, Tabassum Majid, Angela Taylor, Andrea M. Kurasz, Bhavana Patel and Glenn Smith | - 2020 - USA - Research article - Setting: USA. Data collection between 22/1/2018 and 06/05/2019. | To identify the research priorities of individuals with DLB and caregivers. | Individuals with DLB and caregivers | 45 DLB  (20 individuals with DLB and 25 care partners) | - Gender: Yes - Ethnicity: Yes - Age: Yes - SES: not stated or no relevant data - YOE: Yes |
| Informal caregiver experiences at the end-of-life of individuals living with dementia with Lewy bodies: an interview study [2]  **Author(s):** Melissa J Armstrong, Slande Alliance, Pamela Corsentino, Angela Lunde and Angela Taylor | - 2022 - USA - Research article - Setting: USA. Data collection between 15/9/2017 and 30/10/2017. | To investigate the end-of-life experiences of informal caregivers of individuals with DLB who died within the prior 5 years. | Informal caregivers, family member or friend of an individual with DLB | 30 DLB | - Gender: Yes - Ethnicity: not stated or no relevant data - Age: not stated or no relevant data - SES: not stated or no relevant data - YOE: not stated or no relevant data |
| Living with dementia with Lewy bodies: an interpretative phenomenological analysis [3]  **Author(s):** Victoria Larsson, Axel Holmbom-Larsen, Gustav Torisson, Eva Lena Strandberg and Elisabet Londos | - 2019 - Sweden - Research article - Setting: Sweden. Data collection took place between 01/12/2015 and 30/06/2017. | To explore the subjective experience of living with DLB. | Males with DLB | 5 DLB | - Gender: Yes - Ethnicity: Yes - Age: Yes - SES: not stated or no relevant data - YOE: not stated or no relevant data |
| End-of-life experiences in dementia with Lewy bodies: qualitative interviews with former caregivers [4]  **Author(s):** Melissa J. Armstrong, Slande Alliance, Angela Taylor, Pamela Corsentino, James E. Galvin | - 2019 - USA - Research article - Setting: USA. Data collection between 15/9/2017 and 30/10/2017. | To investigate caregiver-reported end-of-life experiences of individuals with DLB and their families. | Caregiver, family member or friend of an individual with DLB who died with a diagnosis of DLB in the past 5 years. | 30 DLB | - Gender: Yes - Ethnicity: not stated or no relevant data - Age: not stated or no relevant data - SES: not stated or no relevant data - YOE: not stated or no relevant data |
| Caregiver-reported barriers to quality end-of-life care in dementia with Lewy bodies: a qualitative analysis [5]  **Author(s):** Melissa J. Armstrong, Slande Alliance, Pamela Corsentino, Susan M. Maixner, Henry L. Paulson and Angela Taylor | - 2020 - USA - Research article - Setting: USA.Data collection between 15/09/2017-30/10/2017. | To investigate barriers to quality end-of-life care in the context of DLB. | Caregiver, family member, or friend to an individual with DLB who died with a DLB diagnosis within 5 years | 30 DLB | - Gender: Yes - Ethnicity: not stated or no relevant data - Age: not stated or no relevant data - SES: not stated or no relevant data - YOE: not stated or no relevant data |
| Clinical care and unmet needs of individuals with dementia with Lewy bodies and caregivers: an interview study [6]  **Author(s):** Melissa J. Armstrong, Noheli Gamez, Slande Alliance, Tabassum Majid, Angela S. Taylor, Andrea M. Kurasz, Bhavana Patel and Glenn E.Smith | - 2021 - USA - Research article - Setting: USA. Telephone interviews occurred from January 2018 to May 2019. | To investigate (1) aspects of care that are helpful and (2) unmet needs. | Individuals with DLB and DLB caregivers | 45 DLB  (20 individuals with DLB and 25 care partners) | - Gender: Yes - Ethnicity: not stated or no relevant data - Age: Yes - SES: not stated or no relevant data - YOE: Yes |
| ‘It's just Clinical care and unmet needs of individuals with dementia experiences of a specialist Lewy body dementia Admiral Nurse service [7]  **Author(s):** Laura J.E.Brown, Zena Aldridge, Amy Pepper, Iracema Leroi, Karen Harrison Dening | - 2022 - UK - Research article - Setting: UK. Interviews conducted from September 2020 and September 2021. | To explore family caregivers’ experiences of the LBD Admiral Nurse service. | LBD family caregivers | 14 LBD | - Gender: Yes - Ethnicity: Yes - Age: Yes - SES: not stated or no relevant data - YOE: not stated or no relevant data |
| The unique experience of spouses in early-onset dementia [8]  **Author(s):** Francine Ducharme, Marie-Jeanne Kergoat, Pascal Antoine, Florence Pasquier and Renée Coulombe | - 2013 - Canada - Research article - Setting: Canada. No dates reported. | To document the lived experience of spouse caregivers of young patients in order to inform the development of professional support tailored to their reality. | Spouses of persons diagnosed with dementia before the age of 65. | 1 Mixed AD and LBD | - Gender: not stated or no relevant data - Ethnicity: not stated or no relevant data - Age: yes - SES: not stated or no relevant data - YOE: not stated or no relevant data |
| Exploring the impact of caring for an individual with neurogenic orthostatic hypotension: a qualitative study [9]  **Author(s):** Katy Gallop, Ngan Pham, Grant Maclaine, Emma Saunders, Bonnie Black and Sarah Acaster | - 2023 - UK - Research article - Setting: UK. Interviews conducted from August-September 2020 | To explore the impact of caring for an individual with neurogenic orthostatic hypotension. | Informal caregivers of individuals with neurogenic orthostatic hypotension and either PD, MSA, PAF or DLB. | 2 DLB | - Gender: not stated or no relevant data - Ethnicity: not stated or no relevant data - Age: not stated or no relevant data - SES: not stated or no relevant data - YOE: not stated or no relevant data |
| Needs and concerns of Lewy body disease family caregivers: a qualitative study [10]  **Author(s):** Kelly E. Stacy, Joseph Perazzo, Rhonna Shatz and Tamilyn Bakas | - 2022 - USA - Research article - Setting: USA. Interviews in January and February 2021. | Identify the needs, concerns, strategies and advice of family caregivers of persons with LBD. | LBD caregivers | 20 LBD | - Gender: Yes - Ethnicity: Yes - Age: Yes - SES: not stated or no relevant data - YOE: Yes |
| Understanding the nature and impact of cognitive fluctuations and sleep disturbances in dementia with Lewy bodies: a qualitative caregiver study [11]  **Author(s):** Ellie Matterson, Kirsty Olsen, John-Paul Taylor, Gemma Wilson-Menzfeld & Greg J.Elder | - 2024 - UK - Research article - Setting: UK. Interviews from July 2021 and August 2022 | To investigate the phenomenology of cognitive fluctuations in DLB by understanding caregiver experiences. The secondary aims of the study were to examine if caregivers considered sleep, or sleep disturbances, to influence the nature of cognitive fluctuations, and to also understand the nature of habitual sleep patterns, sleep changes and sleep disturbances, and their impact upon people with DLB and their caregivers. | DLB caregivers | 7 DLB | - Gender: Yes - Ethnicity: not stated or no relevant data - Age: yes (patients only) - SES: not stated or no relevant data - YOE: not stated or no relevant data |
| A qualitative study of female caregiving spouses’ experiences of intimate relationships as cognition declines in Parkinson’s disease [12]  **Author(s):** Sabina Vatter, Kathryn R.McDonald, Emma Stanmore, Linda Clare, Sheree A.McCormick and Iracema Leroi | - 2018 - UK - Research article - Setting: UK. Interviews from November 2016 and March 2017. | To explore the changes in long-term intimate relationships in Parkinson’s-related dementia, as perceived by spouses providing care to their partners. | Female caregiving spouses of people with PD-MCI, PDD or DLB | 3 DLB | - Gender: Yes - Ethnicity: Yes - Age: Yes - SES: not stated or no relevant data - YOE: not stated or no relevant data |
| Problems faced by people living at home with dementia and incontinence: causes, consequences and potential solutions [13]  **Author(s):** Catherine Murphy, Christine De Laine, Margaret Macaulay, Kelly Hislop Lennie and Mandy Fader | - 2021 - UK - Research article - Setting: UK. Data collection took place from January to October 2019. | To establish the range of causes, consequences and potential solutions of toilet-use and incontinence problems for PLWD and their care partners. | People with dementia, care partners and continence nurses or dementia nurses | 3 LBD  (1 individual with LBD and 2 care partners) | - Gender: Yes - Ethnicity: Yes - Age: not stated or no relevant data - SES: not stated or no relevant data - YOE: not stated or no relevant data |
| Perceived benefits of using nonpharmacological interventions in older adults with Alzheimer’s disease or dementia with Lewy bodies [14]  **Author(s):** Juyoung Park, Heather Howard, Magdalena I.Tolea and James E. Galvin | - 2020 - USA - Research article - Setting: USA. No dates provided. | Explore the perceived effects of three non-pharmacological interventions in managing symptoms in older adults with AD or DLB from family caregiver’s perspectives. | Caregivers of older adults with dementia | 1 DLB | - Gender: Yes - Ethnicity: not stated or no relevant data - Age: Yes - SES: not stated or no relevant data - YOE: not stated or no relevant data |
| Difficulties and associated coping methods regarding visual hallucinations caused by dementia with Lewy bodies [15]  **Author(s):** Akiyo Yumoto and Sayuri Suwa | - 2021 - Japan - Research article - Setting: Japan. Data collected from May and September 2015. | (1) To explore the difficulties experienced by people with DLB because of their visual hallucinations, while considering the associated impact of the realisation that these apparitions are not real, and (2) to determine the coping methods used by both people with DLB and care partners to provide suggestions for care. | People with DLB (with input also obtained from their family members) | 10 DLB | - Gender: Yes - Ethnicity: not stated or no relevant data - Age: Yes - SES: not stated or no relevant data - YOE: not stated or no relevant data |
| Carers to people with Lewy body dementia and Alzheimer's disease: experiences and coping strategies [16]  **Author(s):** Ellen J Svendsboe, | - 2018 - Sweden - Thesis - Setting: Sweden. No dates provided | Overall aim of the thesis was to analyse and describe how the care partners to people with mild AD and DLB experience their life as a care partner. The aim of the included study (study IV) was to describe the different experiences of care partners to people with DLB and AD and explore how coping strategies may be applied to support them. | DLB and AD spouses, adult children, and grandchildren | 9 DLB | - Gender: not stated or no relevant data - Ethnicity: not stated or no relevant data - Age: not stated or no relevant data - SES: not stated or no relevant data - YOE: not stated or no relevant data |
| Key components of post-diagnostic support for people with dementia and their carers: a qualitative study [17]  **Author(s):** Claire Bamford, Alison Wheatley, Greta Brunskill, Laura Booi, Louise Allan, Sube Banerjee, Karen Harrison Dening, Jill Manthorpe, Louise Robinson, on behalf of the PriDem study team | - 2021 - UK - Research article - Setting: UK. Data collection from July and December 2019. | To identify the components of post-diagnostic dementia support. | People with dementia and caregivers (as well as service managers, funders, and frontline staff) | 5 LBD  (2 individuals with LBD and 3 care partners) | - Gender: not stated or no relevant data - Ethnicity: not stated or no relevant data - Age: not stated or no relevant data - SES: not stated or no relevant data - YOE: not stated or no relevant data |
| Profiling conversation in Parkinson’s disease with cognitive impairment [18]  **Author(s):** Anne Whitworth, Ruth Lesser and Ian McKeith | - 1999 - UK - Research article - Setting: UK. No dates provided | To examine the impact of cognitive impairment on the interaction between people with PD and their care partners. | People with PD who have mild cognitive impairment as well as those who have progressed to mild-moderate DLB together with their main care partners. | 6 DLB | - Gender: not stated or no relevant data - Ethnicity: not stated or no relevant data - Age: Yes - SES: not stated or no relevant data - YOE: not stated or no relevant data |
| The human need for equilibrium: qualitative study on the ingenuity, technical competency, and changing strategies of people with dementia seeking health information [19]  **Author(s):** Emma Dixon, Jesse Anderson, Diana C Blackwelder, Mary L Radnofsky and Amanda Lazar | - 2022 - USA - Research article - Setting: USA. Interview and observation sessions were completed between July and September 2020. | (1) Discover the nature, content, and evolution of information behaviours of people living several years after a dementia diagnosis, and (2) identify the motivations for changing information behaviours over time. | People with dementia who have been living with the condition for several (4 to 26) years. | 4 mixed LBD | - Gender: Yes - Ethnicity: not stated or no relevant data - Age: Yes - SES: not stated or no relevant data - YOE: Yes |
| Development of assessment toolkits for improving the diagnosis of the Lewy body dementias: feasibility study within the DIAMOND Lewy study [20]  **Author(s):** Alan J. Thomas, John Paul Taylor, Ian McKeith, Claire Bamford, David Burn, Louise Allan and John O’Brien | - 2017 - UK - Research article - Setting: UK. DIAMOND-Lewy contractual start date was 2014. | The aim of the pilot was to ensure that the diagnosis tools were acceptable to staff and could be integrated into current assessment procedures in busy clinical services. | Clinicians, people with LBD, and caregivers | 3 DLB dyads | - Gender: not stated or no relevant data - Ethnicity: not stated or no relevant data - Age: not stated or no relevant data - SES: not stated or no relevant data - YOE: not stated or no relevant data |
| Improving the diagnosis and management of Lewy body dementia: the DIAMOND-Lewy research programme including pilot cluster RCT [21]  **Author**(s): John T O’Brien, John-Paul Taylor, Alan Thomas, Claire Bamford, Luke Vale, Sarah Hill, Louise Allan, Tracy Finch, Richard McNally, Louise Hayes, Ajenthan Surendranathan, Joseph Kane, Alexandros E Chrysos, Allison Bentley, Sally Barker, James Mason, David Burn and Ian McKeith | - 2021 - UK - Programme Grants for Applied Research report - Setting: UK. DIAMOND-Lewy project had a contractual start date of 2014. | The aim of the qualitative work as part of work package 5 was to explore stakeholder views on the acceptability of the toolkits. | For work package 5: People with DLB or PDD, care partners (and professionals) | 10 LBD  (6 individuals with LBD and 4 care partners) | Work package 5:   - Gender: not stated or no relevant data - Ethnicity: not stated or no relevant data - Age: not stated or no relevant data - SES: not stated or no relevant data - YOE: not stated or no relevant data |
| Applying an analytical process to longitudinal narrative interviews with couples living and dying with Lewy body dementia [22]  **Author(s):** Allison Bentley, Yakubu Salifu and Catherine Walshe | - 2021 - UK - Research article - Setting: UK. Interviews occurred from July 2019 to February 2020. | To provide an analytical example by applying Murray’s levels of narrative analysis in health psychology to longitudinal narrative data gathered with couples living with LBD to show how this application can reveal a richer understanding of the lived experience and provide a deeper insight into complex issues. | Person living with LBD and their spouse. | 2 LBD  (1 individual with LBD and 1 care partner) | - Gender: Yes - Ethnicity: not stated or no relevant data - Age: Yes - SES: not stated or no relevant data - YOE: not stated or no relevant data |
| Problematising carer identification: a narrative study with older partner's providing end-of-life care [23]  **Author(s):** Tessa Morgan, Robbie Duschinsky, Merryn Gott and Stephen Barclay | - 2021 - UK - Research article - Setting: UK. Study conducted from August 2018 to August 2019. | To understand the carer identification practices of older partners providing end-of-life care. | Older partners/care partners providing end-of-life care | 2 Parkinson's/LBD & LBD | - Gender: unclear (relational label used) - Ethnicity: Yes - Age: Yes - SES: not stated or no relevant data - YOE: not stated or no relevant data |
| Pacemaker implants and their influence on the daily life of patients with dementia with Lewy bodies: a qualitative case study [24]  **Author(s):** Isak Heyman, Annika Brorsson, Torbjrön Persson, Elisabet Londos | - 2023 - Sweden - Research article - Setting: Sweden. Initial interviews were conducted in 2020. | To explore how people with DLB experience daily life following a pacemaker implant to manage associated symptoms of bradyarrhythmia. | People with DLB and their family care partners | 2 DLB dyads | - Gender: Yes - Ethnicity: not stated or no relevant data - Age: Yes - SES: not stated or no relevant data - YOE: not stated or no relevant data |
| Using care navigation to address caregiver burden in dementia: a qualitative case study analysis [25]  **Author(s):** Alissa Bernstein, Jennifer Merrilees, Sarah Dulaney, Krista L. Harrison, Winston Chiong, Paulina Ong, Julia Heunis, Jeff Choi, Reilly Walker, Julie E. Feuer, Kirby Lee, Daniel Dohan, Stephen J. Bonasera, Bruce L. Miller, Katherine L. Possin | - 2020 - USA - Research article - Setting: Study conducted across multiple sites in the USA. No dates reported. | To understand specific approaches used by Care Team Navigators to address caregiver burden, and to provide an in-depth analysis of three exemplary cases to illustrate how caregiver-focused education, communication, and care strategies were incorporated into the implementation of a model of dementia care as a way to extend the reach of caregiver support. | Care Team Navigators employed by the Care Ecosystem programme. These are unlicensed trained dementia specialists who work directly through the phone and internet with caregivers and patients. Case studies included informal care partners. | 10 unspecified formal care partners | - Gender: Yes - Ethnicity: Yes - Age: Yes - SES: Yes - YOE: Yes |
| COVID-19: association between increase of behavioral and psychological symptoms of dementia during lockdown and caregivers’ poor mental health [26]  **Author(s):** Elodie Pongan, Jean-Michel Dorey, Celine Borg, Jean Claude Getenet, Romain Bachelet, Charles Lourioux, Bernard Laurent, COVCARE Group, Romain Rey and Isabelle Rouch | - 2021 - France - Research article - Setting: France. Data collection from 15 April 2020 to 15 June 2020. | 1) To describe the prevalence of behavioural changes in people with dementia during the COVID-19 lockdown, and 2) to look for associations between such behavioural changes and various aspects of caregivers’ mental health, especially burden, depression, and anxiety. | Caregivers of people with dementia living at home in France | 23 LBD | - Gender: not stated or no relevant data - Ethnicity: not stated or no relevant data - Age: not stated or no relevant data - SES: not stated or no relevant data - YOE: not stated or no relevant data |
| Comparison of the caregiving experience of grief, burden, and quality of life in dementia with Lewy bodies, Alzheimer’s disease, and Parkinson’s disease dementia [27]  **Author(s):** Taylor Rigby, David K. Johnson, Angela Taylor and James E. Galvin | - 2021 - USA - Research article - Setting: USA. No dates reported. | To examine the differences in the caregiver experience between DLB, PDD and AD. | Current caregivers of people with AD, DLB or PDD | 384 DLB | - Gender: Yes - Ethnicity: Yes - Age: Yes - SES: not stated or no relevant data - YOE: Yes |
| Lewy body dementia: caregiver burden and unmet needs [28]  **Author(s):** James E. Galvin, John E. Duda, Daniel I. Kaufer, Carol F. Lippa, Angela Taylor, and Steven H. Zarit | - 2010 - USA - Research article - Setting: USA. No dates reported. | To ascertain the unmet needs of LBD caregivers and collect data to inform educational programming and enhance caregiver support. | LBD caregivers | 962 LBD | - Gender: Yes - Ethnicity: not stated or no relevant data - Age: Yes - SES: not stated or no relevant data - YOE: Yes |
| Stress and burden among caregivers of patients with Lewy body dementia [29]  **Author(s):** Amanda N. Leggett, Steven Zarit, Angela Taylor and James E. Galvin | - 2011 - USA - Research article - Setting: USA. Survey between December 2007 and April 2008. | Examine the dimensions of subjective burden of caregivers of patients with LBD using data from a unique Internet survey. | LBD caregivers | 611 LBD | - Gender: Yes - Ethnicity: not stated or no relevant data - Age: Yes - SES: not stated or no relevant data - YOE: Yes |
| Differences in the experience of caregiving between spouse and adult child caregivers in dementia with Lewy bodies [30]  **Author(s):** Taylor Rigby, Robert T. Ashwill, David K. Johnson and James E. Galvin | - 2019 - USA - Research article - Setting: USA. No dates reported. | To examine differences in the caregiving experiences of spouses versus adult child caregivers in individuals with DLB. | Adult child and spouse caregivers of people with DLB | 415 DLB | - Gender: Yes - Ethnicity: Yes - Age: Yes - SES: not stated or no relevant data - YOE: Yes |
| Pre-loss grief in caregivers of older adults with dementia with Lewy bodies [31]  **Author(s):** Juyoung Park and James E.Galvin | - 2021 - USA - Research article - Setting: USA. Online survey available online for 3 months (2012-2013). | (1) Examine factors associated with pre-loss grief in caregivers of older adults with DLB and compare their pre-loss grief with that of caregivers of persons living with AD and other dementias, (2) determine whether overall caregiver experiences differed according to the stages of the care recipient with DLB, and (3) compare pre-loss grief between caregivers of living recipients and former caregivers of recently deceased recipients. | Caregivers of older adults with DLB, AD or other dementias | 488 DLB | - Gender: Yes - Ethnicity: Yes - Age: Yes - SES: not stated or no relevant data - YOE: Yes |
| The role of sexual disinhibition to predict caregiver burden and desire to institutionalize among family dementia caregivers [32]  **Author(s):** Kimberly R. Chapman, Geoffrey Tremont, Paul Malloy and Mary Beth Spitznagel | - 2020 - USA - Research article - Setting: USA. Study participation took place between October and December 2017. | To better understand associations between sexual disinhibition in dementia and family caregiver burden and desire to place the care recipient in a structured living facility. | Family caregivers of PLWD | 68 LBD | - Gender: not stated or no relevant data - Ethnicity: not stated or no relevant data - Age: not stated or no relevant data - SES: not stated or no relevant data - YOE: not stated or no relevant data |
| Pain in patients with different dementia subtypes, mild cognitive impairment, and subjective cognitive impairment [33]  **Author(s):** Tarik T. Binnekade, Erik J.A. Scherder, Andrea B. Maier, Frank Lobbezoo, Eduard J. Overdorp, Didi Rhebergen, Roberto S.G.M. Perez, and Joukje M. Oosterman | - 2018 - The Netherlands - Research article - Setting: the Netherlands. Data were collected in Amsterdam and Amstelveen (2014–2015) and in Zutphen (2004–2015). | (1) To assess the pain prevalence, pain intensity, and pain medication use in older patients with a diagnosed subtype of dementia, MCI and SCI, and (2) to assess whether pain prevalence differs between clinical groups (i.e., dementia subtypes and MCI) and a control group of elderly individuals with subjective memory complaints adjusted for demographic variables and mood. | People with AD, VaD, MD, FTD, DLB, MCI or as well as SCI. | 23 DLB | - Gender: Yes - Ethnicity: not stated or no relevant data - Age: Yes - SES: not stated or no relevant data - YOE: Yes |
| The insula, a grey matter of tastes: a volumetric MRI study in dementia with Lewy bodies [34]  **Author(s):** Nathalie Philippi, Vincent Noblet, Malik Hamdaoui, David Soulier, Anne Botzung, Emmanuelle Ehrhard, Benjamin Cretin, Frédéric Blanc and AlphaLewyMA study group | - 2020 - France - Research article - Setting: France. No dates reported. | To explore potential changes in personal tastes and to test a potential relationship with the insular atrophy. From a clinical perspective, the aim was to better understand behavioural changes in DLB patients and to develop a potential clinical tool that could reflect insular damage. | People with early stage DLB and healthy controls | 23 DLB | - Gender: Yes - Ethnicity: not stated or no relevant data - Age: Yes - SES: not stated or no relevant data - YOE: Yes |
| Dementia patients caregivers quality of life: the PIXEL study [35]  **Author(s):** Philippe Thomas, Fabrice Lalloué, Pierre-Marie Preux, Cyril Hazif-Thomas, Sylvie Pariel, Robcis Inscale, Joël Belmin and Jean-Pierre Clément | - 2006 - France - Research article - Setting: France. **Survey took place during the first semester 2004.** | This study aims to determine the parameters influencing caregivers’ QoL and its possible link with patients’ QoL. | People with dementia living at home and dementia caregivers | 7 LBD dyads | - Gender: not stated or no relevant data - Ethnicity: not stated or no relevant data - Age: not stated or no relevant data - SES: not stated or no relevant data - YOE: not stated or no relevant data |
| Cause of death and end-of-life experiences in individuals with dementia with Lewy bodies [36]  **Author**(s): Melissa J. Armstrong, Slande Alliance, Pamela Corsentino, Steven T. DeKosky and Angela Taylor | - 2019 - USA - Research article - Setting: USA. No dates reported. | To investigate the natural history, cause of death and end-of-life experiences of individuals with DLB. | Caregivers, family members or friend of an individual who had died with a diagnosis of DLB in the past 5 year. | 658 DLB | - Gender: Yes - Ethnicity: not stated or no relevant data - Age: Yes - SES: not stated or no relevant data - YOE: not stated or no relevant data |
| Questionnaire survey of satisfaction with medication for five symptom domains of dementia with Lewy bodies among patients, their caregivers, and their attending physicians [37]  **Author(s):** Shunji Toya, Yuta Manabe, Mamoru Hashimoto, Hajime Yamakage and Manabu Ikeda | - 2023 - Japan - Research article - Setting: Japan. Study conducted from September 2020 to July 2021 | To assess the satisfaction of DLB patients, caregivers and their attending physicians/clinicians with medication for five symptom domains in DLB: cognitive impairment, parkinsonism, psychiatric symptoms, sleep-related disorders and autonomic dysfunction. | People with DLB, DLB caregivers and attending physicians | 110 DLB dyads | - Gender: Yes - Ethnicity: not stated or no relevant data - Age: Yes - SES: not stated or no relevant data - YOE: not stated or no relevant data |
| Lewy body dementia: the caregiver experience of clinical care [38]  **Author(s):** James E. Galvin, John E. Duda, Daniel I. Kaufer, Carol F. Lippa, Angela Taylor and Steven H. Zarit | - 2010 - USA - Research article - Setting: USA. No dates reported. | To ascertain the experiences of LBD caregivers and collect data to improve diagnosis and management of LBD. | LBD caregivers | 962 LBD | - Gender: Yes - Ethnicity: not stated or no relevant data - Age: Yes - SES: not stated or no relevant data - YOE: Yes |
| Research priorities of individuals and caregivers with Lewy body dementia a web-based survey [39]  **Author(s):** Samantha K. Holden, Noheli Bedenfield, Angela S. Taylor, Ece Bayram, Chris Schwilk, Jori Fleisher, John Duda, Holly Shill, Henry L. Paulson, Kelly Stacy, Julia Wood, Pamela Corsentino, Sharon J. Sha, Irene Litvan, David J. Irwin, Joseph F. Quinn, Jennifer G. Goldman, Katherine Amodeo, John-Paul Taylor, Bradley F. Boeve and Melissa J. Armstrong | - 2023 - USA - Research article - Setting: USA. Survey conducted between 07/04/2021 and 01/07/2021. | To identify the research priorities of people personally affected by LBD. Specific caregiving needs in LBD and perceptions of research participation were also investigated. | People with LBD and LBD caregivers | 721 DLB  (97 people with DLB, 624 care partners and 21 unspecified) | - Gender: Yes - Ethnicity: Yes - Age: Yes - SES: not stated or no relevant data - YOE: Yes |
| Treatment needs of dementia with Lewy bodies according to patients, caregivers, and physicians: a cross-sectional, observational questionnaire-based study in Japan [40]  **Author(s):** Mamoru Hashimoto, Yuta Manabe, Takuhiro Yamaguchi, Shunji Toya and Manabu Ikeda | - 2022 - Japan - Research article - Setting: Japan. Conducted from September 2020 to July 2021. | Clarify (1) the treatment needs of patients with DLB and their caregivers, (2) the extent to which the attending clinicians understand the treatment needs of their patients with DLB and their caregivers, and (3) what factors contribute to the lack of understanding of the attending clinicians regarding the treatment needs of their patients with DLB and their caregivers. | DLB patients, caregivers and attending clinicians | 263 DLB dyads | - Gender: Yes - Ethnicity: not stated or no relevant data - Age: Yes - SES: not stated or no relevant data - YOE: Yes |
| The impact of covid-19 quarantine on patients with dementia and family caregivers: a nation-wide survey [41]  **Author(s):** Innocenzo Rainero, Amalia C. Bruni, Camillo Marra, Annachiara Cagnin, Laura Bonanni, Chiara Cupidi, Valentina Laganà, Elisa Rubino, Alessandro Vacca, Raffaele Di Lorenzo, Paolo Provero, Valeria Isella, Nicola Vanacore, Federica Agosta, Ildebrando Appollonio, Paolo Caffarra, Cinzia Bussè, Renato Sambati, Davide Quaranta, Valeria Guglielmi, Giancarlo Logroscino, Massimo Filippi, Gioacchino Tedeschi, Carlo Ferrarese and the SINdem COVID-19 Study Group | - 2021 - Italy - Research article - Setting: Italy. Interviews between 14 April 2020 and 17 April 2020. | To investigate the clinical changes in patients with AD and other dementia and to evaluate caregivers’ distress during COVID-19 quarantine. | Family caregivers of people with dementia living at home | 360 DLB | - Gender: Yes - Ethnicity: not stated or no relevant data - Age: Yes - SES: not stated or no relevant data - YOE: not stated or no relevant data |
| The importance of educating the Lewy body dementia community on risks and benefits of lumbar punctures in LBD biomarker research [42]  **Author(s):** Angela Taylor, Pamela Corsentino, Bethany Peterson, Ian Richard, Anna Long and James B. Leverenz | - 2018 - USA - Conference poster - Setting: USA. No dates provided. | To attain a better understanding of the knowledge, concerns, and opinions of people with LBD and their caregivers on research lumbar punctures. | People with LBD and their caregivers | 76 LBD  (63 care partners and 13 individuals with LBD) | - Gender: not stated or no relevant data - Ethnicity: not stated or no relevant data - Age: not stated or no relevant data - SES: not stated or no relevant data - YOE: not stated or no relevant data |
| Behavioral and psychological effects of coronavirus disease-19 quarantine in patients with dementia [43]  **Author(s):** Annachiara Cagnin, Raffaele Di Lorenzo, Camillo Marra, Laura Bonanni, Chiara Cupidi, Valentina Laganà, Elisa Rubino, Alessandro Vacca, Paolo Provero, Valeria Isella, Nicola Vanacore, Federica Agosta, Ildebrando Appollonio, Paolo Caffarra, Ilaria Pettenuzzo, Renato Sambati, Davide Quaranta, Valeria Guglielmi, Giancarlo Logroscino, Massimo Filippi, Gioacchino Tedeschi, Carlo Ferrarese, Innocenzo Rainero, Amalia C. Bruni and SINdem COVID-19 Study Group | - 2020 - Italy - Research article - Setting: Italy. Survey administered between 14 and 24th April 2020 | To assess modifications of neuropsychiatric symptoms during quarantine in PLWD and their caregivers. Specifically, the aim was to investigate the frequency and type of changes in BPSD during the first month of COVID-19 quarantine in patients with different types of brain diseases leading to dementia and the psychological effects in their caregivers. | Family caregivers of people with AD, DLB, FTD and VaD | 360 DLB | - Gender: Yes - Ethnicity: not stated or no relevant data - Age: Yes - SES: not stated or no relevant data - YOE: not stated or no relevant data |
| Video research visits for atypical parkinsonian syndromes among Fox Trial Finder participants [44]  **Author(s):** Christopher G. Tarolli, Grace A. Zimmerman, Steven Goldenthal, Blake Feldman, Sarah Berk, Bernadette Siddiqi, Catherine M. Kopil, Sohini Chowdhury, Kevin M. Biglan, E. Ray Dorsey and Jamie L. Adams | - 2020 - USA - Research article - Setting: USA. No dates reported. | To evaluate the diagnostic concordance between video-based versus self-reported diagnoses of MSA, PSP, DLB, and CBS. They also assessed patient satisfaction with video-based visits. | People with atypical parkinsonian disorders | 3 DLB | - Gender: Yes - Ethnicity: Yes - Age: Yes - SES: not stated or no relevant data - YOE: Yes |
| Support and information needs following a diagnosis of dementia with Lewy bodies [45]  **Author(s):** Alison Killen, Darren Flynn, Aoife De Brún, Nicola O’Brien, John O’Brien, Alan J. Thomas, Ian McKeith and John-Paul Taylor | - 2016 - UK - Research article - Setting: UK. Online survey conducted during May 2014. | To explore the information and support needs of people with DLB and their caregivers around the point of diagnosis, in order to inform the development of theory-based, directly delivered interventions to improve coping with stress and increase QoL. | People with DLB and their family members | 125 DLB  (3 individuals with DLB and  122 care partners) | - Gender: Yes - Ethnicity: not stated or no relevant data - Age: not stated or no relevant data - SES: not stated or no relevant data - YOE: not stated or no relevant data |
| Characteristics of eating and swallowing problems in patients who have dementia with Lewy bodies [46]  **Author(s):** Shunichiro Shinagawa, Hiroyoshi Adachi, Yasutaka Toyota, Takaaki Mori, Izumi Matsumoto, Ryuji Fukuhara and Manabu Ikeda | - 2009 - Japan - Research article - Setting: Japan. No dates provided. | To clarify the frequency and characteristics of eating problems in patients with DLB. | People with DLB or AD | 29 DLB | - Gender: Yes - Ethnicity: not stated or no relevant data - Age: Yes - SES: not stated or no relevant data - YOE: Yes |
| Pain in extrapyramidal neurodegenerative diseases [47]  **Author(s):** Shlomit Yust-Katz, Ronly Hershkovitz, Tanya Gurevich and Ruth Djaldetti | - 2017 - Israel - Research article - Setting: Israel. No dates reported. | To examine the prevalence and characteristics of pain in PD and other Parkinson plus syndromes and patients’ use and response to pain medications. | People with PD or Parkinson plus syndromes (MSA, DLBD, PSP, CBD) | 8 DLB | - Gender: Yes - Ethnicity: not stated or no relevant data - Age: Yes - SES: not stated or no relevant data - YOE: not stated or no relevant data |
| Self-efficacy and social support for psychological well-being of family caregivers of care recipients with dementia with Lewy bodies, Parkinson’s disease dementia, or Alzheimer’s disease [48]  **Author(s):** Juyoung Park, Magdalena I. Tolea, Victoria Arcay, Yve Lopes & James E. Galvin | - 2019 - USA - Research article - Setting: USA. No dates reported. | Study aimed to identify factors associated with depressive symptoms in family caregivers of persons with DLB, AD or PDD. It compared the burden, grief, and depressive symptoms of caregivers across these conditions. Additionally, it examined the role of caregivers' self-efficacy and received social support in improving psychological well-being and investigated whether self-efficacy mediates the relationship between caregiver burden and psychological well-being, as well as between caregiver grief and psychological well-being. | Spouses or adult child caregivers of living persons with any of the various types of dementia. | 453 DLB | - Gender: Yes - Ethnicity: yes - Age: yes - SES: yes - YOE: yes |
| Dementia subtype and living well: results from the Improving the experience of Dementia and Enhancing Active Life (IDEAL) study [49]  **Author(s):** Yu-Tzu Wu, Linda Clare, John V. Hindle, Sharon M. Nelis, Anthony Martyr, Fiona E. Matthews and on behalf of the Improving the experience of Dementia and Enhancing Active Life study | - 2018 - UK - Research article - Setting: UK. Recruited between July 2014 and August 2016. | To investigate the potential impact of dementia subtypes on the capability to live well for both PLWD and their care partners. | Community-dwelling PLWD and, where possible, their care partner. | 43 DLB dyads | - Gender: not stated or no relevant data - Ethnicity: not stated or no relevant data - Age: not stated or no relevant data - SES: not stated or no relevant data - YOE: not stated or no relevant data |
| Factors of dementia caregiver burden differentially contribute to desire to institutionalize [50]  **Author(s):** John T. Martin, Kimberly R. Chapman, Christopher Was and Mary Beth Spitznagel | - 2022 - USA - Research article - Setting: USA. Participation took place between October and December 2017. | To examine a model of caregiver burden using the Zarit Burden Interview, and then to explore whether individual factors differentially contribute to a caregiver’s steps toward the decision to place a dementia care recipient in a structured living facility. | Caregivers of people with dementia | 25 LBD | - Gender: not stated or no relevant data - Ethnicity: not stated or no relevant data - Age: not stated or no relevant data - SES: not stated or no relevant data - YOE: not stated or no relevant data |
| Caregiver self-efficacy and associated factors among caregivers of patients with dementia with Lewy bodies and caregivers of patients with Alzheimer’s disease [51]  **Author(s):** Hirofumi Sato, Shutaro Nakaaki, Junko Sato, Ryo Shikimoto, Toshi A. Furukawa, Masaru Mimura and Tatsuo Akechi | - 2021 - Japan - Research article - Setting: Japan. Recruitment between September 2016 and March 2019. | To examine the differences in caregiver self-efficacy and their associated factors between DLB and AD caregivers | DLB and AD caregivers | 46 DLB | - Gender: Yes - Ethnicity: not stated or no relevant data - Age: Yes - SES: not stated or no relevant data - YOE: Yes |
| Comparison of QOL between patients with different degenerative dementias, focusing especially on positive and negative affect [52]  **Author(s):** Kairi Kurisu, Seishi Terada, Etsuko Oshima, Makiko Horiuchi, Nao Imai, Mayumi Yabe, Osamu Yokota, Takeshi Ishihara and Norihito Yamada | - 2016 - Japan - Research article - Setting: Japan. Data collection took place between September 2008 and April 2012 | To compare the QoL of individuals with different dementias, especially focusing on positive and negative affect, using an objective QoL scale for dementia. | Outpatients with AD, DLB or FTD | 28 DLB | - Gender: Yes - Ethnicity: not stated or no relevant data - Age: Yes - SES: not stated or no relevant data - YOE: Yes |
| Patients with Lewy body dementia use more resources than those with Alzheimer’s disease [53]  **Author(s):** Fredrik Boström, Linus Jönsson, Lennart Minthon and Elisabet Londos | - 2007 - Sweden - Research article - Setting: Sweden. Patients were included from May to July 2005. | To compare resource use and costs in patients with DLB and AD and assess determinants of costs of care in DLB. | People with DLB or AD | 34 DLB | - Gender: Yes - Ethnicity: not stated or no relevant data - Age: Yes - SES: not stated or no relevant data - YOE: not stated or no relevant data |
| Examining carer stress in dementia: the role of subtype diagnosis and neuropsychiatric symptoms [54]  **Author(s):** David R Lee, Ian McKeith, Urs Mosimann, Arunima Ghosh-Nodyal and Alan J Thomas | - 2013 - UK - Research article - Setting: UK. No dates reported. | To examine the specific impacts of dementia subtype diagnosis and neuropsychiatric symptoms on the stress of a group of dementia care partners | Care partners of PLWD | 29 DLB | - Gender: Yes - Ethnicity: not stated or no relevant data - Age: Yes - SES: not stated or no relevant data - YOE: not stated or no relevant data |
| Attitudes toward own aging and cognition among individuals living with and without dementia: Findings from the IDEAL programme and the PROTECT study [55]  **Author(s):** Serena Sabatini, Anthony Martyr, Obioha C. Ukoumunne, Clive Ballard, Rachel Collins, Claire Pentecost, Jennifer M. Rusted, Catherine Quinn, Kaarin J. Anstey, Sarang Kim, Anne Corbett, Helen Brooker and Linda Clare | - 2022 - UK - Research article - Setting: UK. Data collection for people with dementia: 2014–2016; for people without dementia: 01/01/2019 to 31/03/2019. | To investigate whether PLWD have more negatives attitudes towards own ageing than individuals without dementia and whether cognition and dementia subtype are associated with attitudes towards own ageing in PLWD. | PLWD and people without dementia | 52 DLB | - Gender: not stated or no relevant data - Ethnicity: not stated or no relevant data - Age: not stated or no relevant data - SES: not stated or no relevant data - YOE: not stated or no relevant data |
| Multidimensional care burden in Parkinson-related dementia [56]  **Author(s):** Sabina Vatter, Kathryn R. McDonald, Emma Stanmore, Linda Clare and Iracema Leroi | - 2018 - UK - Research article - Setting: UK. Postal questionnaire ran from July 2017 to January 2018. INVEST study assessments were performed between April 2016 and July 2017. | To explore the factor structure of the ZBI in life partners of people with Parkinson-related dementia and to examine the relationships among the emerging factors and the demographic and clinical features. | Life partners of people with Parkinson-related dementia | 49 DLB | - Gender: Yes - Ethnicity: yes - Age: yes - SES: no - YOE: yes |
| Care burden and mental ill health in spouses of people with Parkinson disease dementia and Lewy body dementia [57]  **Author(s):** Sabina Vatter, Emma Stanmore, Linda Clare, Kathryn R. McDonald, Sheree A. McCormick and Iracema Leroi | - 2020 - UK - Research article - Setting: UK. Nested within the INVEST study. According to the protocol, the INVEST study and trial started in January 2016 and recruited dyads from March 2016. | To explore and compare levels of mental health, care burden, and relationship satisfaction among caregiving spouses of people with mild cognitive impairment or dementia in Parkinson disease (PD-MCI or PDD) or DLB. | Spouses of people with PD-MCI/PDD/DLB | 49 DLB | - Gender: Yes - Ethnicity: yes - Age: yes - SES: not stated or no relevant data - YOE: yes |
| Capgras syndrome in dementia with Lewy bodies [58]  **Author(s):** Papan Thaipisutikul, Iryna Lobach, Yael Zweig, Ashita Gurnani, and James E. Galvin | - 2013 - USA - Research article - Setting: USA. Participants drawn from a series of consecutive referrals from August 2010 to August 2011. | To review clinical characteristics of DLB with Capgras syndrome compared to those without Capgras syndrome and its potential impact on DLB caregivers. | People with DLB (and a caregiver as a study informant [paid and unpaid]) | 55 DLB | - Gender: Yes - Ethnicity: not stated or no relevant data - Age: yes - SES: not stated or no relevant data - YOE: yes |
| Autonomic symptoms are predictive of dementia with Lewy bodies [59]  **Author(s):** Wenzheng Hu, Shuai Liu, Fei Wang, Han Zhu, Xiaoshan Du, Lingyun Ma, Jinghuan Gan, Hao Wu, Xiaodan Wang, Yong Ji | - 2022 - China - Research article - Setting: China. Recruitment between June 2016 and March 2019. | To evaluate the time of onset of autonomic symptoms in patients with DLB using the SCOP-Aut questionnaire. | People with DLB or AD and controls | 106 DLB | - Gender: Yes - Ethnicity: not stated or no relevant data - Age: yes - SES: not stated or no relevant data - YOE: yes |
| The frequency and correlates of anxiety in patients with first-time diagnosed mild dementia [60]  **Author(s):** Minna J. Hynninen, Monica H. Breitve, Arvid Rongve, Dag Aarsland and Inger Hilde Nordhus | - 2012 - Norway - Research article - Setting: Norway. Screening took place from March 2005 to March 2007. | To assess the frequency of caregiver- and patient-reported anxiety and associations between anxiety and socio-demographic and clinical variables in an outpatient sample with first-time diagnosed mild dementia. | Outpatients with first-time diagnosed mild dementia and care partners | 29 DLB | - Gender: Yes - Ethnicity: not stated or no relevant data - Age: yes - SES: not stated or no relevant data - YOE: yes |
| Caregiver burden in family carers of people with dementia with Lewy bodies and Alzheimer’s disease [61]  **Author(s):** Ellen Svendsboe, Toril Terum, Ingelin Testad, Dag Aarsland, Ingun Ulstein, Anne Corbett and Arvid Rongve | - 2016 - Norway - Research article - Setting: Norway. Data collected between 2005 and 2013. | To characterise the differences in caregiver distress between care partners of people diagnosed with DLB and people with AD, with a view to differentiating and improving support for caregivers. | AD or DLB care partners | 86 DLB | - Gender: Yes - Ethnicity: not stated or no relevant data - Age: yes (for patients) - SES: not stated or no relevant data - YOE: yes |
| Patients with dementia with Lewy bodies have more impaired quality of life than patients with Alzheimer disease [62]  **Author(s):** Fredrik Boström; Linus Jönsson; Lennart Minthon, and Elisabet Londos | - 2007 - Sweden - Research article - Setting: Sweden. No dates reported | (1) To compare QoL in patients with DLB and AD, and (2) to investigate determinants of QoL in DLB | Patients with DLB or AD | 34 DLB | - Gender: Yes - Ethnicity: not stated or no relevant data - Age: yes - SES: not stated or no relevant data - YOE: not stated or no relevant data |
| Study of prevalence of neuropsychiatric symptoms in elderly dementia patients [63]  **Author(s):** Mohan Mahale, Pradeep Behal, Nitul M Bewal, Vivek Aggarwal, Anuj Singhal, Arun Kumar Yadav and Vivek Vasdev | - 2021 - India - Research article - Setting: India. Conducted between September 2015 and August 2019. | To study the prevalence and type of neuropsychiatric symptoms in dementia patients presenting to a Tertiary Care Hospital and to find out the association between neuropsychiatric symptoms and the type of dementia and to assess the association of the caregiver stress in this setting. | People with dementia | 17 LBD | - Gender: not stated or no relevant data - Ethnicity: not stated or no relevant data - Age: Yes - SES: not stated or no relevant data - YOE: not stated or no relevant data |
| The association between specific neuropsychiatric disturbances in people with Alzheimer's disease and dementia with Lewy bodies and carer distress [64]  **Author(s):** Toril Marie Terum, Ingelin Testad, Arvid Rongve, Dag Aarsland, Ellen Svendsboe and John Roger Andersen | - 2019 - Norway - Research article - Setting: Norway. Participants were recruited between 2005 and 2007 and continued selectively until 2013. | (1) To investigate multivariate associations between individual neuropsychiatric symptoms in people with mild dementia living at home and care partner distress, and (2) To explore whether there are differences in the way individual neuropsychiatric symptoms are associated with care partner distress in relation to diagnosis. | People with AD or DLB and their care partners | 62 DLB dyads | - Gender: Yes - Ethnicity: not stated or no relevant data - Age: Yes - SES: not stated or no relevant data - YOE: not stated or no relevant data |
| Association of premorbid personality with behavioral and psychological symptoms in dementia with Lewy bodies: Comparison with Alzheimer’s disease patients [65]  **Author(s):** Kazuki Tabata, Yasuaki Saijo, Fumiyoshi Morikawa, Juichiro Naoe, Eiji Yoshioka, Yasuyuki Kawanishi, Yoshihiko Nakagi and Takahiko Yoshida | - 2017 - Japan - Research article - Setting: Japan. No dates reported. | To elucidate the relation between premorbid personality traits and BPSD in DLB and AD patients. | People with DLB or AD | 41 DLB | - Gender: Yes - Ethnicity: not stated or no relevant data - Age: yes - SES: not stated or no relevant data - YOE: yes |
| Visual hallucinations in eye disease and Lewy body disease [66]  **Author(s):** Prabitha Urwyler, Tobias Nef, René Müri, Neil Archibald, Selina Margaret Makin, Daniel Collerton, John-Paul Taylor, David Burn, Ian McKeith and Urs Peter Mosimann | - 2016 - Switzerland - Research article - Setting: Switzerland. No dates provided. | To compare the phenomenology and characteristics of visual hallucinations in eye disease, PD, and LBD patients. Also explored the effects of gender on hallucinatory content. | People with eye disease, PD, DLB or PDD and controls | 31 DLB | - Gender: Yes - Ethnicity: not stated or no relevant data - Age: yes - SES: not stated or no relevant data - YOE: yes |
| Cognition, hallucination severity and hallucination-specific insight in neurodegenerative disorders and eye disease [67]  **Author(s):** Marcella Montagnese, Miriam Vignando, Daniel Collerton, Dominic ffytche, Urs Peter Mosimann, John-Paul Taylor, Katrina daSilva Morgan & Prabitha Urwyler | - 2022 - UK - Research article - Setting: UK. No dates provided. | Investigated the relationships between cognition, hallucination-specific insight, temporal, emotion and severity aspects of hallucinations and what aspects of the relationships might be the same or different when comparing neurodegenerative disease and eye disease. | People with different neurological disorders that experience visual hallucinations | 27 DLB | - Gender: Yes - Ethnicity: not stated or no relevant data - Age: yes - SES: not stated or no relevant data - YOE: not stated or no relevant data |
| Health related quality of life in individuals with cognitive decline and discrepancies between patients and their proxies [68]  **Author(s):** Iban Onandia-Hinchado and Unai Diaz-Orueta | - 2019 - Spain - Research article - Setting: Spain. No dates provided. | To measure HRQoL in individuals with different types of dementia, MCI and healthy controls, and assess agreement levels between participants and proxies. | PLWD, MCI and healthy controls | 19 DLB | - Gender: Yes - Ethnicity: not stated or no relevant data - Age: yes - SES: not stated or no relevant data - YOE: yes |
| Health related quality of life and cognitive decline in older populations: preliminary results from NeuroDemeNPsia study [69]  **Author(s):** Iban Onandia-Hinchado and Unai Diaz-Orueta | - 2020 - Spain - Research article - Setting: Spain. No dates provided. | To measure HRQoL in people with different primary degenerative dementias and compare them with 50+ years old older individuals with MCI and healthy controls. | People with dementia, MCI and healthy controls | 27 DLB | - Gender: Yes - Ethnicity: not stated or no relevant data - Age: yes - SES: not stated or no relevant data - YOE: yes |
| Caregiver burden, sleep quality, depression, and anxiety in dementia caregivers: a comparison of frontotemporal lobar degeneration, dementia with Lewy bodies, and Alzheimer’s disease [70]  **Author(s):** Shuai Liu, Jing Liu, Xiao-Dan Wang, Zhihong Shi, Yuying Zhou, Jing Li, Tao Yu and Yong Ji | - 2018 - China - Research article - Setting: China. Study conducted from March 2011 to January 2014. | To investigate caregiver burden, sleep quality, and stress in caregivers of patients with frontotemporal lobar degeneration and DLB, as compared with caregivers of patients with AD. | Caregivers of people with frontotemporal lobar degeneration, DLB or AD | 36 DLB dyads | - Gender: Yes - Ethnicity: not stated or no relevant data - Age: yes - SES: not stated or no relevant data - YOE: not stated or no relevant data |
| Patient affect and caregiver burden in dementia [71]  **Author(s):** Yoshiko Kawano, Seishi Terada, Shintaro Takenoshita, Satoshi Hayashi, Yoshitaka Oshima, Tomoko Miki, Osamu Yokota and Norihito Yamada | - 2020 - Japan - Research article - Setting: Japan. Recruited patients referred between September 2008 and February 2012. | To compare the caregiver burden of AD, DLB and behavioural variant FTD, paying attention to differences in the positive affect of patients. | Caregivers of people with AD, DLB or FTD | 26 DLB | - Gender: Yes - Ethnicity: not stated or no relevant data - Age: yes - SES: not stated or no relevant data - YOE: Yes (only for patients) |
| Comparison of caregiver burden between dementia with Lewy bodies and Alzheimer’s disease [72]  **Author(s):** Seiji Yuuki, Mamoru Hashimoto, Asuka Koyama, Masateru Matsushita,Tomohisa Ishikawa, Ryuji Fukuhara, Kazuki Honda, Yusuke Miyagawa, Manabu Ikeda and Minoru Takebayashi | - 2023 - Japan - Research article - Setting: Japan. Patients had attended the hospital between October 2010 and 2017. | Compare levels of caregiver burden and the possible influential factors on caregiver burden between DLB and AD. | Caregivers of people with DLB or AD | 93 DLB | - Gender: Yes - Ethnicity: not stated or no relevant data - Age: Yes - SES: not stated or no relevant data - YOE: Yes (only for patients) |
| Impact of behavioral and psychological symptoms on caregiver burden in patients with dementia with Lewy bodies [73]  **Author(s):** Hideki Kanemoto, Shunsuke Sato, Yuto Satake, Fuyuki Koizumi, Daiki Taomoto, Atsushi Kanda, Tamiki Wada, Kenji Yoshiyama and Manabu Ikeda | - 2021 - Japan - Research article - Setting: Japan. Retrospectively recruited patients included in the database from April 2009 to December 2019. | Sought to classify BPSD and to reveal what type of BPSD affects caregiver burden in patients with DLB. | Caregivers of people with DLB | 102 DLB | - Gender: Yes - Ethnicity: not stated or no relevant data - Age: yes - SES: not stated or no relevant data - YOE: not stated or no relevant data |
| Factors associated with burden among male caregivers for people with dementia [74]  **Author(s):** Chia-Fen Tsai, Mao-Hsuan Huang, Jun-Jun Lee, Kai-Ming Jhang, Ling-Chun Huang, Li-Kai Huang, Wei-Ju Lee, Pi-Shan Sung, Yi-Chien Liu, Wen-Chen Ouyang, Chih-Ming Cheng, Chih-Cheng Hsu, Jong-Ling Fuh | - 2022 - Taiwan - Research article - Setting: Taiwan. Used data from the National Dementia Registry Study in Taiwan which was initiated in 2017. | Aimed to identify factors that may be associated with burden among male caregivers of PLWD. | Male caregivers of people with AD, mixed dementia, FTD, DLB, PDD or unknown dementia | 10 DLB dyads | - Gender: not stated or no relevant data - Ethnicity: not stated or no relevant data - Age: not stated or no relevant data - SES: not stated or no relevant data - YOE: not stated or no relevant data |
| Differential associations of clinical features with cerebrospinal fluid biomarkers in dementia with Lewy bodies and Alzheimer’s disease [75]  **Author(s):** Fabricio Ferreira de Oliveira, Marjorie Câmara Miraldo, Eduardo Ferreira de Castro‑Neto, Sandro Soares de Almeida, Sandro Luiz de Andrade Matas, Paulo Henrique Ferreira Bertolucci and Maria da Graça Nafah‑Mazzacoratti | - 2023 - Brazil - Research article - Setting: Brazil. Recruited from September 2015 to October 2017. | To explore associations of cerebrospinal fluid biomarkers of neurodegeneration and amyloidosis with caregiver burden, cognition and functionality in DLB paired with late-onset AD and healthy older people. | People with a diagnosis of DLB or late-onset AD or healthy controls | 27 DLB | - Gender: Yes - Ethnicity: not stated or no relevant data - Age: yes - SES: not stated or no relevant data - YOE: yes |
| Clinical findings, functional abilities and caregiver distress in the early stage of dementia with Lewy bodies (DLB) and Alzheimer’s disease (AD) [76]  **Author(s):** Monica Ricci, Silvia Vittoria Guidoni, Micaela Sepe-Monti, Giuseppe Bomboi, Giovanni Antonini, Carlo Blundo, Franco Giubilei | - 2009 - Italy - Research article - Setting: Italy. No dates reported. | Assess neuropsychiatric disorders, functional abilities and caregiver distress in DLB and in AD subjects. | People with DLB or AD and their caregivers | 16 DLB | - Gender: Yes - Ethnicity: not stated or no relevant data - Age: yes (patients only) - SES: not stated or no relevant data - YOE: yes (patients only) |
| The effects of behavioral and psychological symptoms on caregiver burden in frontotemporal dementia, Lewy body dementia, and Alzheimer's disease: clinical experience in China [77]  **Author(s):** Shuling Liu, Yi Jin, Zhihong Shi, Ya Ruth Huo, Yalin Guan, Mengyuan Liu, Shuai Liu & Yong Ji | - 2017 - China - Research article - Setting: China. Recruited patients attending clinic between June 2014 and July 2015. | (1) Determine which BPSD have the greatest impact on burden in caregivers for FTD, DLB, and AD patients, (2) compare the effects of BPSD on caregiver burden across FTD variants, DLB, and AD, and (3) identify patient and caregiver variables that contribute to caregiver burden. | Caregivers of people with FTD, DLB or AD | 22 DLB | - Gender: Yes - Ethnicity: not stated or no relevant data - Age: yes - SES: yes - YOE: yes |
| Subjective experience of time in dementia with Lewy bodies during COVID-19 lockdown [78]  **Author(s):** Dylan Torboli, Giovanna Mioni, Cinzia Bussé & Annachiara Cagnin & Antonino Vallesi | - 2023 - Italy - Research article - Setting: Italy. The first run of interviews was carried out in April 2020, while the second run was in July 2020. | Assess the subjective experience of the passage of time for present and past time intervals during the lockdown due to coronavirus disease for people with DLB. | People with DLB and DLB caregivers | 36 DLB  (22 individuals with DLB and  14 care partners) | - Gender: Yes - Ethnicity: not stated or no relevant data - Age: yes - SES: not stated or no relevant data - YOE: yes |
| A brief psychometric and clinimetric evaluation of self-report burden and mental health measures completed by care partners of people with Parkinson's-related dementia [79]  **Author(s):** Sabina Vatter, Kathryn R. McDonald, Emma Stanmore, Sheree A. McCormick, Linda Clare and Iracema Leroi | - 2020 - UK - Brief report - Setting: UK. Data were obtained from a cross-sectional study with informal care partners of people with PD-MCI/ PDD/DLB, which was nested in the INVEST trial. According to the protocol, the INVEST study and trial started in January 2016. | To assess psychometric properties and describe clinimetric properties of self-report measures completed by informant care partners of people with PD-MCI/PDD/DLB to gain insight into the suitability, reliability and usability of the scales in this population and to help future researchers and clinicians with decision-making regarding appropriate instruments. | Care partners of people with PD-MCI/PDD/DLB | 49 DLB | - Gender: Yes - Ethnicity: yes - Age: yes - SES: not stated or no relevant data - YOE: yes |
| Tau in dementia with Lewy bodies [80]  **Author(s):** Kai Sin Chin, Leonid Churilov, Vincent Doré, Victor L Villemagne, Christopher C Rowe, Nawaf Yassi and Rosie Watson | - 2024 - Australia - Research article - Setting: Australia. No dates reported. | Investigate tau in people with DLB using a second-generation tau positron emission tomography tracer as well as plasma p-tau181. | People with DLB | 27 DLB | - Gender: Yes - Ethnicity: not stated or no relevant data - Age: yes - SES: not stated or no relevant data - YOE: yes |
| Correlates of neuropsychiatric and motor tests with language assessment in patients with Lewy body dementia [81]  **Author(s):** Fernando Chiodini Machado, Fabricio Ferreira de Oliveira, Sheilla de Medeiros Correia Marin, Gustavo Sampaio and Paulo Henrique Ferreira Bertolucci | - 2020 - Brazil - Research article - Setting: Brazil. Participants assessed from January 2014 to April 2015. | To correlate neuropsychiatric and motor assessments with language and visual organization tests in LBD. | People with LBD | 22 DLB | - Gender: Yes - Ethnicity: not stated or no relevant data - Age: yes - SES: not stated or no relevant data - YOE: yes |
| Association between amyloid-beta deposition and cortical thickness in dementia with Lewy bodies [82]  **Author(s):** Kai Sin Chin, Sanuji Gajamange, Patricia M Desmond, Victor L Villemagne, Christopher C Rowe, Leonid Churilov, Nawaf Yassi and Rosie Watson | - 2023 - Australia - Research article - Setting: Australia. No dates reported. | To investigate the clinical and imaging correlates of amyloid-beta deposition in DLB, particularly its relationship with cortical thickness in Alzheimer’s disease–prone regions and hippocampal volume. | People with DLB | 24 DLB | - Gender: Yes - Ethnicity: not stated or no relevant data - Age: yes - SES: not stated or no relevant data - YOE: yes |
| Neuropsychiatric feature profiles of patients with Lewy body dementia [83]  **Author(s):** Fabricio Ferreira de Oliveira, Fernando Chiodini Machado, Gustavo Sampaio, Sheilla de Medeiros Correia Marin, Maria da Graça Naffah-Mazzacoratti and Paulo Henrique Ferreira Bertolucci | - 2020 - Brazil - Research article - Setting: Brazil. Recruited from January 2014 to June 2017. | Assessed risk factors for age at dementia onset, as well as which neuropsychiatric features are associated with pharmacotherapy and signs and symptoms of LBD. | People with PDD or DLB | 37 DLB | - Gender: Yes - Ethnicity: not stated or no relevant data - Age: yes - SES: not stated or no relevant data - YOE: yes |
| Delusions in patients with dementia with Lewy bodies and the associated factors [84]  **Author(s):** Ray-Chang Tzeng, Ching-Fang Tsai, Ching-Tsu Wang, Tzu-Yuan Wang and Pai-Yi Chiu | - 2018 - Taiwan - Research article - Setting: Taiwan.From 1 October 2015 to 21 June 2017, patients who fulfilling the criteria for DLB were included. | To investigate the associated factors of delusions in patients with DLB. | Outpatients with DLB registered in a regional hospital’s database | 207 DLB | - Gender: Yes - Ethnicity: not stated or no relevant data - Age: yes - SES: not stated or no relevant data - YOE: yes |
| Contrasts between patients with Lewy body dementia syndromes and APOE- ε3/ε3 patients with late-onset Alzheimer disease dementia [85]  **Author(s):** Fabricio F. Oliveira, Fernando C. Machado, Gustavo Sampaio, Sheilla M. C. Marin, Elizabeth S. Chen, Marilia C. Smith and Paulo H. F. Bertolucci | - 2015 - Brazil - Research article - Setting: Brazil. All patients with LBD who had consultations in the first semester of 2014 were invited. | Aimed to compare and distinguish demographic and neuropsychiatric features between LBD and *APOE*- **ε**3/**ε**3 late-onset AD. | Patients with PDD, DLB or *APOE*- **ε**3/**ε**3 late-onset AD | 25 DLB | - Gender: Yes - Ethnicity: not stated or no relevant data - Age: yes - SES: not stated or no relevant data - YOE: yes |
| Neuropsychological study of amyotrophic lateral sclerosis and parkinsonism-dementia complex in Kii peninsula, Japan [86]  **Author(s):** Akihiro Shindo, Yukito Ueda, Shigeki Kuzuhara and Yasumasa Kokubo | - 2014 - Japan - Research article - Setting: Japan. No dates reported | To clarify the neuropsychological features of the patients with ALS/PDC of the Kii peninsula | People with Kii ALS/PDC or AD or PSP, or frontotemporal lobar degeneration or DLB | 10 DLB | - Gender: Yes - Ethnicity: not stated or no relevant data - Age: yes - SES: not stated or no relevant data - YOE: yes |
| Clinical features of delusional jealousy in elderly patients with dementia [87]  **Author(s):** Mamoru Hashimoto, Shinichi Sakamoto, Manabu Ikeda | - 2015 - Japan - Research article - Setting: Japan. study period: September 2011- August 2012. | To investigate the clinical features of delusional jealousy and possible mechanisms whereby delusional jealousy arises in PLWD. | Outpatients with dementia | 38 DLB | - Gender: not stated or no relevant data - Ethnicity: not stated or no relevant data - Age: not stated or no relevant data - SES: not stated or no relevant data - YOE: not stated or no relevant data |
| Depression in dementia with Lewy bodies: A comparison with Alzheimer’s disease [88]  **Author(s):** Pai-Yi Chiu, Chein-Wei Wang, Chun-Tang Tsai, Shin-Hua Li, Chih-Li Lin and Te-Jen Lai | - 2017 - Taiwan - Research article - Setting: Taiwan. Participants enrolled from 10/02/10 to 09/02/12. | To compare the frequencies, severity and symptoms of depression between DLB and AD. | People with DLB or AD | 71 DLB | - Gender: Yes - Ethnicity: not stated or no relevant data - Age: yes - SES: not stated or no relevant data - YOE: yes |
| Costs of dementia with Lewy bodies: A Chinese multicenter cross-sectional study [89]  **Author(s):** Xia Guo, Qingbo Meng, Meimei Zuo, Zhou Su, Jinghuan Gan, Xu‐Dong Li, Hongcan Zhu, Baozhi Gang, Jinping Niu, Shuai Liu and Yong Ji | - 2023 - China - Research article - Setting: China. Data collected from August to December 2021. | To evaluate the current economic costs of DLB and its related factors. | People with DLB (caregivers as supporting informants) | 193 DLB | - Gender: Yes - Ethnicity: not stated or no relevant data - Age: yes - SES: not stated or no relevant data - YOE: yes |
| Characterization of symptoms and determinants of disease burden in dementia with Lewy bodies: DEvELOP design and baseline results [90]  **Author(s):** M. van de Beek, I. van Steenoven, J. J. van der Zande, I. Porcelijn, F. Barkhof, C. J. Stam, P. G. H. M. Raijmakers, P. Scheltens, C. E. Teunissen, W. M. van der Flier and A. W. Lemstra | - 2021 - The Netherlands - Research article - Setting: the Netherlands. Inclusion of patients started in March 2016. | To describe the design and baseline results of DEvELOP (The DEmEntia with LEwy bOdies Project). To investigate the associations between core and suggestive DLB symptoms and different aspects of disease burden i.e., IADL functioning, QoL and caregiver burden. | People with DLB and MCI-LB from the DEvELOP cohort | 100 DLB | - Gender: Yes - Ethnicity: not stated or no relevant data - Age: yes - SES: not stated or no relevant data - YOE: yes |
| Patterns of carer distress over time in mild dementia [91]  **Author(s):** Ellen J. Svendsboe, Ingelin Testad, Toril Terum, Assmus Jörg, Anne Corbett, Dag Aarsland, Arvid Rongve | - 2018 - Norway - Research article - Setting: Norway. No dates provided | To study the level of care partner reported distress in mild dementia over a 3‐year period. | Caregivers of people with DLB or AD | 63 DLB | - Gender: Yes - Ethnicity: not stated or no relevant data - Age: yes (only for patients) - SES: not stated or no relevant data - YOE: Yes (only for patients) |
| Time until nursing home admission in people with mild dementia: comparison of dementia with Lewy bodies and Alzheimer’s dementia [92]  **Author(s):** Arvid Rongve, Corinna Vossius, Sabine Nore, Ingelin Testad and Dag Aarsland | - 2014 - Norway - Research article - Setting: Norway. Study termination: June 2011. Recruited outpatients referred during 2005-2007. | Studied time until nursing home admission in mild dementia and predictors for nursing home admission in people with DLB and how it compares to AD. | People with mild dementia | 43 DLB | - Gender: Yes - Ethnicity: not stated or no relevant data - Age: yes - SES: not stated or no relevant data - YOE: yes |
| Trajectories and determinants of quality of life in dementia with Lewy bodies and Alzheimer’s disease [93]  **Author(s):** Marleen van de Beek, Inger van Steenoven, Inez H.G.B. Ramakers, Pauline Aalten, Huiberdina L. Koek, Marcel G.M. Olde Rikkert, Judith Manniën, Janne M. Papma, Frank Jan de Jong, Afina W. Lemstra and Wiesje M. van der Flier | - 2019 - The Netherlands - Research article - Setting: The Netherlands. No dates reported. | To investigate determinants and trajectories of QoL in DLB compared to AD and controls. | People with DLB or AD and healthy controls | 29 DLB | - Gender: Yes - Ethnicity: not stated or no relevant data - Age: yes - SES: not stated or no relevant data - YOE: yes |
| Disease progression in dementia with Lewy bodies: a longitudinal study on clinical symptoms, quality of life and functional impairment [94]  **Author(s):** Marleen van de Beek, Annemartijn van Unnik, Inger van Steenoven, Jessica van der Zande, Frederik Barkhof, Charlotte E. Teunissen, Wiesje van der Flier and Afina W. Lemstra | - 2022 - The Netherlands - Research article - Setting: the Netherlands. The first patient was included in 2016. | To identify associations with rate of change in cognition, everyday functioning and quality of life. | People with DLB and MCI-LB from the DEvELOP cohort | 121 DLB | - Gender: Yes - Ethnicity: not stated or no relevant data - Age: yes - SES: not stated or no relevant data - YOE: yes |
| Personality traits distinguishing dementia with Lewy bodies from Alzheimer disease [95]  **Author**(s): James E. Galvin, Heather Malcom, David Johnson and John C. Morris | - 2007 - USA - Research article - Setting: USA. Enrolment began in 1979. | To identify personality traits that distinguish DLB from AD | Patients with a neuropathological diagnosis of DLB or AD and nondemented controls | 128 DLB | - Gender: Yes - Ethnicity: yes - Age: yes - SES: not stated or no relevant data - YOE: yes |
| The use and costs of paid and unpaid care for people with dementia: longitudinal findings from the IDEAL cohort [96]  **Author(s):** Catherine Henderson, Martin Knapp, Anthony Martyr, Laura D. Gamble, Sharon M. Nelis, Catherine Quinn, Claire Pentecost, Rachel Collins, Yu-Tzu Wu, Ian R. Jones, Christina R. Victor, James A. Pickett, Roy W. Jones, Fiona E. Matthews, Robin G. Morris, Jennifer Rusted, Jeanette M. Thom and Linda Clare on behalf of the IDEAL programme team | - 2022 - UK - Research article - Setting: Britain. Study utilised data from waves 1 to 3, collected over the periods 2014-2016, 2015-2017, and 2016-2018 respectively. | To characterise use and costs of paid and unpaid care over time in a cohort of people with dementia living in Britain. To explore the relationship between cohort members’ demographic and clinical characteristics and service costs. | Community-dwelling people with dementia and their caregivers | 53 DLB dyads | - Gender: not stated or no relevant data - Ethnicity: not stated or no relevant data - Age: not stated or no relevant data - SES: not stated or no relevant data - YOE: not stated or no relevant data |
| Frequency of suicidal ideation and associated clinical features in Lewy body dementia [97]  **Author(s):** Melissa J. Armstrong, Kathryn Moore, Charles E. Jacobson, Noheli Bedenfield, Bhavana Patel, Jennifer L. Sullivan | - 2021 - USA - Research article - Setting: USA. The clinic started collecting the BDI-II in 2010 and the data query occurred in January 2021. | To use a single-centre database to identify the frequency of suicidal ideation in LBD at a specialty movement disorders clinic and factors associated with suicidal ideation in this population. | People with a diagnosis of LBD | 67 DLB | - Gender: Yes - Ethnicity: yes - Age: yes - SES: not stated or no relevant data - YOE: not stated or no relevant data |
| A comparison of caregiver burden for different types of dementia: an 18-month retrospective cohort study [98]  **Author(s):** Wen-Chien Huang, Ming-Che Chang, Wen-Fu Wang and Kai-Ming Jhang | - 2022 - Taiwan - Research article - Setting: Taiwan. Included PLWD and their caregivers who were assessed between October 2015 and November 2020. | To elucidate the influence of dementia etiologies on the degree of caregiver burden and determine which factors predict a high caregiving burden. | Caregivers of people with AD, VCI, Mixed AD and VCI, LBD, FTD and other | 61 LBD dyads | - Gender: not stated or no relevant data - Ethnicity: not stated or no relevant data - Age: not stated or no relevant data - SES: not stated or no relevant data - YOE: not stated or no relevant data |
| Impact of the COVID-19 pandemic and lockdown on anxiety, depression and nursing burden of caregivers in Alzheimer’s disease, dementia with Lewy bodies and mild cognitive impairment in China: a 1-year follow-up study [99]  **Author(s):** Xinran Bao, Junying Xu, Qingbo Meng, Jinghuan Gan, Xiao-Dan Wang, Hao Wu, Shuai Liu and Yong Ji | - 2022 - China - Research article - Setting: China. Patients and their caregivers recruited at T0 (from 30 September to 31 December 2019) before the pandemic of COVID-19 and followed-up on face-to-face at T1 (from 30 September to 31 December 2020). | (1) Investigate effects of the pandemic on anxiety, depression and care burden in caregivers of nursing patients with AD, DLB and MCI, over a one-year period, and (2) investigate predictive factors, especially in terms of physical activity, social contact and sleep disturbance, with respect to the worsening or improvement of the issues of concern. | Caregivers of people with AD, DLB or MCI | 22 DLB dyads | - Gender: Yes - Ethnicity: not stated or no relevant data - Age: yes - SES: not stated or no relevant data - YOE: yes |
| Prodromal dementia with Lewy bodies: clinical characterization and predictors of progression [100]  **Author(s):** Marleen van de Beek, Inger van Steenoven, Jessica J. van der Zande, Frederik Barkhof, Charlotte E. Teunissen, Wiesje M. van der Flier and Afina W. Lemstra | - 2020 - The Netherlands - Research article - Setting: the Netherlands. No dates reported. | To examine clinical characteristics, cognitive decline, and predictors for time to dementia in MCI-LB compared with MCI-AD. | People with MCI-LB or MCI-AD | 73 DLB | - Gender: Yes - Ethnicity: not stated or no relevant data - Age: yes - SES: not stated or no relevant data - YOE: yes |
| Updates on somatoform disorders (SFMD) in Parkinson's disease and dementia with Lewy bodies and discussion of phenomenology [101]  **Author(s):** Marco Onofrj, Astrid Thomas, Pietro Tiraboschi, Gregor Wenning, Francesco Gambi, Gianna Sepede, Massimo Di Giannantonio, Caterina Di Carmine, Daniela Monaco, Valerio Maruotti, Fausta Ciccocioppo, Maria Chiara D'Amico and Laura Bonanni | - 2011 - Italy - Research article - Setting: Italy. This report updates a previous study based on patients evaluated from 1999 to 2006. | To update a previous report [102] of observations in a cohort of patients and further detail clinical phenomenology of SFMD. | People with PD, DLB, AD, FTD, MSA and PSP | 162 DLB | - Gender: Yes - Ethnicity: not stated or no relevant data - Age: yes - SES: not stated or no relevant data - YOE: yes |
| Cohort study on somatoform disorders in Parkinson disease and dementia with Lewy bodies [102]  **Author(s):** Marco Onofrj, Laura Bonanni, Lamberto Manzoli and Astrid Thomas | - 2010 - Italy - Research article - Setting: Italy. The study was based on a cohort of 1,360 new patients evaluated from 1999 to 2008. | To assess SFMD prevalence and impact in PD and DLB. | People with PD, DLB, AD, MSA, PSP and FTD | 124 DLB | - Gender: Yes - Ethnicity: not stated or no relevant data - Age: yes - SES: not stated or no relevant data - YOE: yes |
| Memantine for patients with Parkinson’s disease dementia or dementia with Lewy bodies: a randomised, double-blind, placebo-controlled trial [103]  **Author(s):** Murat Emre, Magda Tsolaki, Ubaldo Bonuccelli, Alain Destée, Eduardo Tolosa, Alexandra Kutzelnigg, Andrés Ceballos-Baumann, Slobodan Zdravkovic, Anna Bladström, Roy Jones, on behalf of the 11018 Study Investigators | - 2010 - Turkey - Research article - Setting: Study took place in specialist centres in Austria, France, Germany, the UK, Greece, Italy, Spain, and Turkey. Recruitment between January 2007 to December 2009. | To investigate the efficacy and safety of memantine | Patients (≥50 years of age) with mild to moderate PDD or DLB. | 75 DLB | - Gender: Yes - Ethnicity: yes - Age: yes - SES: not stated or no relevant data - YOE: not stated or no relevant data |
| Music playlists for people with dementia: trialling a guide for caregivers [104]  **Author(s):** Sandra Garrido, Laura Dunne, Catherine J. Stevens and Esther Chang | - 2020 - Australia - Research article - Setting: Australia. No dates provided. | Report on the trial of a Guide for use of music with 45 PLWD and their caregivers in residential care facilities and home-based care. | PLWD (AD, non-specific dementia, DLB or FTD) | Unclear | - Gender: not stated or no relevant data - Ethnicity: not stated or no relevant data - Age: not stated or no relevant data - SES: not stated or no relevant data - YOE: not stated or no relevant data |
| Bilateral nucleus basalis of Meynert deep brain stimulation for dementia with Lewy bodies: a randomised clinical trial [105]  **Author(s):** James Gratwicke, Ludvic Zrinzo, Joshua Kahan, Amy Peters, Una Brechany, Ann McNichol, Mazda Beigi, Harith Akram, Jonathan Hyam, Ashwini Oswal, Brian Day, Laura Mancini, John Thornton, Tarek Yousry, Sebastian J. Crutch, John-Paul Taylor, Ian McKeith, Lynn Rochester, Jonathan M. Schott, Patricia Limousin, David Burn, Martin N. Rossor, Marwan Hariz, Marjan Jahanshahi and Thomas Foltynie | - 2020 - UK - Research article - Setting: UK. Patients assessed between May 2014 and February 2016. | (1) To evaluate the general safety and tolerability of a nucleus basalis of Meynert (NBM) deep brain stimulation (DBS) procedure in DLB patients, and (2) to compare scores on a battery of standardised cognitive, psychiatric and motor tests pre-operatively and repeated after six weeks of bilateral NBM DBS and six weeks of sham deep brain stimulation. | People with DLB | 6 DLB | - Gender: Yes - Ethnicity: not stated or no relevant data - Age: yes - SES: not stated or no relevant data - YOE: not stated or no relevant data |
| Adjunct zonisamide to levodopa for DLB parkinsonism: a randomized double-blind phase 2 study [106]  **Author(s):** Miho Murata, Toshinari Odawara, Kazuko Hasegawa, Sayaka Iiyama, Masatoshi Nakamura, Masaaki Tagawa and Kenji Kosaka | - 2018 - Japan - Research article - Setting: Japan. Conducted between March 2013 and April 2014. | To investigate the efficacy and safety of zonisamide as an adjunct to levodopa therapy for parkinsonism in patients with DLB. | Outpatients with probable DLB | 158 DLB | - Gender: Yes - Ethnicity: not stated or no relevant data - Age: yes - SES: not stated or no relevant data - YOE: not stated or no relevant data |
| Donepezil for dementia with Lewy bodies: a randomized, placebo-controlled trial [107]  **Author(s):** Etsuro Mori, Manabu Ikeda and Kenji Kosaka on behalf of the Donepezil-DLB Study Investigators | - 2012 - Japan - Research article - Setting: Japan. Recruited from October 2007 to February 2010. | To investigate the effects of donepezil. | People with DLB | 140 DLB | - Gender: Yes - Ethnicity: not stated or no relevant data - Age: yes - SES: not stated or no relevant data - YOE: not stated or no relevant data |
| Quality of life and the effect of memantine in dementia with Lewy bodies and Parkinson’s disease dementia [108]  **Author(s):** Victoria Larsson, Knut Engedal, Dag Aarsland, Carina Wattmo, Lennart Minthon and Elisabet Londos | - 2011 - Sweden - Research article - Setting: Sweden. Conducted between 2005 and 2008. | To investigate QoL and the effect of memantine treatment in patients with LBD. | People with DLB or PDD | 22 DLB | - Gender: Yes - Ethnicity: not stated or no relevant data - Age: yes - SES: not stated or no relevant data - YOE: not stated or no relevant data |
| Long-term donepezil use for dementia with Lewy bodies: results from an open-label extension of Phase III trial [109]  **Author(s):** Etsuro Mori, Manabu Ikeda, Reiko Nagai, Kazutaka Matsuo, Masaki Nakagawa and Kenji Kosaka | - 2015 - Japan - Research article - Setting: Japan. Recruited throughout February to March 2012. | Assess the long-term efficacy and safety of donepezil 10mg in patients with DLB. | People with DLB | 110 DLB | - Gender: Yes - Ethnicity: not stated or no relevant data - Age: yes - SES: not stated or no relevant data - YOE: not stated or no relevant data |
| Efficacy of adjunctive therapy with zonisamide versus increased dose of levodopa for motor symptoms in patients with dementia with Lewy bodies: the randomized, controlled, non-Inferiority DUEL study [110]  **Author(s):** Manabu Ikeda, Etsuro Mori, Satoshi Orimo, Tomomi Yamada and Osamu Konishi | - 2023 - Japan - Research article - Setting: Japan. Study conducted between March 2021 and July 2022. | To compare adjunct zonisamide 25mg/day versus an increased levodopa dose in patients with DLB treated with levodopa ≤300 mg/day for parkinsonism. | People with DLB who had residual parkinsonism on doses of levodopa or ≤300 mg/day. | 50 DLB | - Gender: Yes - Ethnicity: not stated or no relevant data - Age: yes - SES: not stated or no relevant data - YOE: not stated or no relevant data |
| Goal-orientated cognitive rehabilitation for dementias associated with Parkinson's disease- A pilot randomised controlled trial [111]  **Author(s):** John V. Hindle, Tamlyn J. Watermeyer, Julie Roberts, Andrew Brand, Zoe Hoare, Anthony Martyr and Linda Clare | - 2018 - UK - Research article - Setting: UK. According to the protocol, the CORD-PD trial started in January 2015. | To examine the appropriateness and feasibility of cognitive rehabilitation for people with dementias associated with Parkinson's. Additional aims included assessing the usefulness of outcome measures and obtaining effect sizes to inform the development of future RCTs of cognitive rehabilitation in PDD and DLB. | People with PD, PDD or DLB and their caregivers | 4 DLB | - Gender: Yes - Ethnicity: not stated or no relevant data - Age: yes - SES: not stated or no relevant data - YOE: yes |
| Efficacy, safety, and tolerability of armodafinil therapy for hypersomnia associated with dementia with Lewy bodies: a pilot study [112]  **Author(s):** Maria I. Lapid, Karen M. Kuntz, Sara S. Mason, Jeremiah A. Aakre, Emily S. Lundt, Walter Kremers, Laura A. Allen, Daniel A. Drubach and Bradley F. Boeve | - 2017 - USA - Research article - Setting: USA. Recruitment between January 2010 and April 2012. | To assess the efficacy, safety and tolerability of armodafinil for hypersomnia associated with DLB. | People with DLB | 20 DLB | - Gender: Yes - Ethnicity: not stated or no relevant data - Age: yes - SES: not stated or no relevant data - YOE: not stated or no relevant data |
| A comparison of the efficacy of donepezil in Parkinson’s disease with Dementia and Dementia with Lewy bodies [113]  **Author(s):** Alan J. Thomas, David J. Burn, Elise N. Rowan, Elizabeth Littlewood, Jane Newby, David Cousins, Sanjeet Pakrasi, Jonathan Richardson, Jonathan Sanders and Ian G. McKeith | - 2005 - UK - Research article - Setting: UK. 20-week study. No dates provided. | To compare the efficacy of donepezil in PDD and DLB. | People with DLB or PDD. | 30 DLB | - Gender: Yes - Ethnicity: not stated or no relevant data - Age: yes - SES: not stated or no relevant data - YOE: not stated or no relevant data |
| Long-term safety and efficacy of donepezil in patients with dementia with Lewy bodies: results from a 52-week, open-label, multicenter extension study [114]  **Author(s):** Manabu Ikeda, Etsuro Mori, Kenji Kosaka, Eizo Iseki, Mamoru Hashimoto, Noriyuki Matsukawa, Kazutaka Matsu, Masaki Nakagawa, on behalf of the Donepezil-DLB Study Investigators | - 2013 - Japan - Research article - Setting: Japan. Conducted between February 2008 and March 2011. | To investigate the safety and efficacy of long-term administration (52 weeks) of donepezil in people with DLB | People living with DLB | 108 DLB | - Gender: Yes - Ethnicity: not stated or no relevant data - Age: yes - SES: not stated or no relevant data - YOE: not stated or no relevant data |
| Introduction of a management toolkit for Lewy body dementia: a pilot cluster-randomized trial [115]  **Author(s):** John T. O'Brien, Ian G. McKeith, Alan J. Thomas, Claire Bamford, Luke Vale, Sarah Hill, Louise Allan, Tracy Finch, Richard McNally, Louise Hayes, Ajenthan Surendranathan, Joseph P.M. Kane, Sarah Dunn, Allison Bentley, Sally Barker, James Mason, David Burn & John-Paul Taylor | - 2021 - UK - Research article - Setting: UK. DIAMOND-Lewy contractual start date was 2014. | To assess the feasibility of undertaking a cluster randomised study of the introduction of an evidence-based management toolkit for LBD, assessing the outcomes for patients and care partners as secondary measures. | People with DLB or PDD and their care partners | 77 DLB dyads | - Gender: Yes - Ethnicity: not stated or no relevant data - Age: yes - SES: not stated or no relevant data - YOE: not stated or no relevant data |
| Lumateperone for treatment of psychotic symptoms in Lewy body: a case report [116]  **Author(s):** Adam M. Bied, Susan W. Njuguna, and Ritvij M. Satodiya | - 2024 - USA - Case report - Setting: USA. Treatment administered over three weeks (September 2022-Novemeber 2022) | To investigate the use of lumateperone for the treatment of psychotic symptoms in LBD and offer a meaningful report to guide clinical practice. | Individual with LBD | 1 LBD | - Gender: Yes - Ethnicity: not stated or no relevant data - Age: yes - SES: not stated or no relevant data - YOE: not stated or no relevant data |
| Cognitive effects of quetiapine in a patient with dementia with Lewy bodies [117]  **Author(s):** Timothy Rice, Yuriy Dobry, Eugene Wang, Vladan Novakovic and Leo Sher | - 2013 - USA - Case report - Setting: USA. Patient presented in 2011. | To describe the cognitive improvement observed in a patient with DLB following the use of an atypical antipsychotic. | Person with DLB | 1 DLB | - Gender: Yes - Ethnicity: not stated or no relevant data - Age: yes - SES: not stated or no relevant data - YOE: yes |
| Open label trial to evaluate the efficacy and safety of Yokukansan, a traditional Asian medicine, in dementia with Lewy bodies [118]  **Author(s):** Koh Iwasaki, Kenji Kosaka, Hideo Mori, Reina Okitsu, Katsutoshi Furukawa, Yuta Manabe, Mitsuhiro Yoshita, Aya Kanamori, Nobuo Ito, Kenji Wada, Jun Horiguchi, Shuhei Yamaguchi, Ryuji Fukuhara, Shinji Ouma, Seigo Nakano, Mamoru Hashimoto and Toru Kinoshita | - 2011 - Japan - Multicentre case series - Setting: Japan. No dates provided. | To investigate the efficacy and safety of yokukansan in DLB. | People with DLB | 63 DLB | - Gender: Yes - Ethnicity: not stated or no relevant data - Age: yes - SES: not stated or no relevant data - YOE: not stated or no relevant data |
| Improvement in delusions and hallucinations in patients with dementia with Lewy bodies upon administration of yokukansan, a traditional Japanese medicine [119]  **Author(s):** Koh Iwasaki, Kenji Kosaka, Hideo Mori, Reina Okitsu, Katsutoshi Furukawa, Yuta Manabe, Mitsuhiro Yoshita, Aya Kanamori, Nobuo Ito, Kenji Wada, Michio Kitayama, Jun Horiguchi, Shuhei Yamaguchi, Shin Takayama, Ryuji Fukuhara, Shinji Ouma, Seigo Nakano, Mamoru Hashimoto and Toru Kinoshita | - 2012 - Japan - Research article - Setting: Japan. No dates provided. | To investigate the efficacy and safety of yokukansan in DLB patients. | People with DLB | 63 DLB | - Gender: Yes - Ethnicity: not stated or no relevant data - Age: yes - SES: not stated or no relevant data - YOE: not stated or no relevant data |
| Effectiveness of ramelteon for treatment of visual hallucinations in dementia with Lewy bodies: a report of 4 cases [120]  **Author(s):** Koji Kasanuki, Eizo Iseki, Yoshiaki Nishida, Hiroshige Fujishiro, Yuhei Chiba, Kiyoshi Sato and Heii Arai | - 2013 - Japan - Case report - Setting: Japan. No dates reported. | To report four cases of patients with DLB treated with ramelteon, producing apparent reductions in visual hallucinations as well as excessive daytime sleepiness and rapid eye movement sleep behaviour disorder. | People with DLB | 4 DLB | - Gender: Yes - Ethnicity: not stated or no relevant data - Age: yes - SES: not stated or no relevant data - YOE: not stated or no relevant data |
| Administration of zonisamide in three cases of dementia with Lewy bodies [121]  **Author(s):** Toshinari Odawara, Kazumasa Shiozaki, Takashi Togo and Yoshio Hirayasu | - 2010 - Japan - Case report - Setting: Japan. No dates reported. | No aim stated but reports the effects of zonisamide in improving the motor function of DLB patients. | People with DLB |  | - Gender: Yes - Ethnicity: not stated or no relevant data - Age: yes - SES: not stated or no relevant data - YOE: not stated or no relevant data |
| Donepezil for treatment of dementia with Lewy bodies: a case series of nine patients [122]  **Author(s):** Catherine Shea, Chris Macknight, and Kenneth Rockwood | - 1998 - Canada - Case series - Setting: Canada. Patients seen between September 1997 and March 1998 | To report an open-label case series of the response to donepezil treatment of individual symptoms in nine patients with DLB. | People with DLB | 3 DLB | - Gender: Yes - Ethnicity: not stated or no relevant data - Age: yes - SES: not stated or no relevant data - YOE: not stated or no relevant data |
| Development and validation of the Lewy body disease caregiver activities scale [123]  **Author(s):** Stacy, Kelly E.; Lambert, Joshua; Shatz, Rhonna | - 2023 - USA - Research article - Setting: USA. No dates provided. | To describe the development and validity testing of the Lewy Body Dementia Caregiver Activities Scale (LBD-CAS). | LBD care partners and experts | 6 LBD | - Gender: Yes - Ethnicity: not stated or no relevant data - Age: not stated or no relevant data - SES: not stated or no relevant data - YOE: not stated or no relevant data |
| Goal setting for cognitive rehabilitation in mild to moderate Parkinson’s disease dementia and dementia with Lewy bodies [124]  **Author(s):** Tamlyn J. Watermeyer, John V. Hindle, Julie Roberts, Catherine L. Lawrence, Anthony Martyr, Huw Lloyd-Williams, Andrew Brand, Petra Gutting, Zoe Hoare, Rhiannon Tudor Edwards and Linda Clare | - 2016 - UK - Research article - Setting: UK. According to the protocol, the CORD-PD trial started in January 2015. | To assess goal setting in early-PDD or DLB. It examined the types of goals set and deemed as important, as well as the nature and themes of goal statements. It also compared goal performance ratings from the perspectives of PDD and DLB participants and their caregivers. | People with a diagnosis of PD, PDD or DLB and caregivers | 4 DLB patients (unclear how many DLB carer partners) | - Gender: Yes - Ethnicity: not stated or no relevant data - Age: yes - SES: not stated or no relevant data - YOE: yes |
| Parkinson’s-adapted cognitive stimulation therapy: a pilot randomized controlled clinical trial [125]  **Author(s):** Iracema Leroi, Sabina Vatter, Lesley-Anne Carter, Sarah J. Smith, Vasiliki Orgeta, Ellen Poliakoff, Monty A. Silverdale, Jason Raw, David J. Ahearn, Christine Taylor, Joanne Rodda, Tarek Abdel-Ghany and Sheree A. McCormick | - 2019 - UK - Research article - Setting: UK.The INVEST study and trial started in January 2016 and recruited dyads from 1 March 2016. | (1) Explore the impact of ‘CST-PD’, which is home-based, individualised cognitive stimulation therapy adapted for a population of people with PD-MCI, PDD or DLB, and (2) evaluate the acceptability of the intervention and the feasibility of conducting a full-scale RCT. | Dyads consisting of people with PD-MCI/PDD/DLB and their care partners | 21 DLB dyads | - Gender: Yes - Ethnicity: yes - Age: yes - SES: not stated or no relevant data - YOE: yes |
| Parkinson’s-adapted cognitive stimulation therapy: feasibility and acceptability in Lewy body spectrum disorders [126]  **Author(s):** Sheree A. McCormick, Sabina Vatter, Lesley‑Anne Carter, Sarah J. Smith, Vasiliki Orgeta, Ellen Poliakoff, Monty A. Silverdale, Jason Raw, David J. Ahearn, Christine Taylor, Joanne Rodda, Tarek Abdel‑Ghany, Benjamin Kwapong and Iracema Leroi | - 2019 - UK - Research article - Setting: UK. According to the protocol, the INVEST study and trial started in January 2016 and recruited dyads from March 2016. | To evaluate the feasibility, acceptability, and tolerability of a home-based, care partner-delivered Cognitive Stimulation Therapy (CST-PD) for individuals with PDD or DLB and their care partners | People with Parkinson’s-related dementia (PDD, DLB or PD-MCI) and their care partners | 21 DLB dyads | - Gender: Yes - Ethnicity: yes - Age: yes - SES: not stated or no relevant data - YOE: yes |
| The feasibility and acceptability of a psychosocial intervention to support people with dementia with Lewy bodies and family care partners [127]  **Author(s):** Alison Killen, Darren Flynn, Nicola O’Brien and John-Paul Taylor | - 2022 - UK - Research article - Setting: UK. No dates provided. | To evaluate a co-designed psychosocial intervention for individuals with DLB and their family care partners. The primary objective was to assess the feasibility and acceptability of recruitment and completion. The three secondary objectives were to: (1) identify the most appropriate primary outcome measure to inform a future multi-centre evaluation study, (2) collect preliminary data on the potential effectiveness of the intervention in enhancing self-efficacy—a construct associated with higher self-esteem, improved well-being, and better adaptation to chronic diseases, and (3) optimise the intervention content. | Dyads consisting of a person with DLB and a family care partner. | 19 DLB dyads | - Gender: Yes - Ethnicity: not stated or no relevant data - Age: yes - SES: not stated or no relevant data - YOE: not stated or no relevant data |
| Learning to PERSEVERE: a pilot study of peer mentor support and caregiver education in Lewy body dementia [128]  **Author(s):** Jori E. Fleisher, Madhuvanthi Suresh, Melissa E. Levin, Serena P. Hess, Faizan Akram, Danielle Dodson, Michelle Tosin, Glenn T. Stebbins, Katheryn Woo, Bichun Ouyang, Joshua Chodosh | - 2023 - USA - Research article - Setting: USA. No dates reported. | Overall objective was to assess the feasibility of a peer mentor-led educational intervention and its impact on caregivers’ knowledge, dementia attitudes and mastery. Specifically, the aims were to (1) incorporate stakeholder input in the intervention via focus group feedback, and 2) assess fidelity, satisfaction, and efficacy on knowledge, attitudes, and mastery among 30 LBD family caregiver mentees, and secondarily, their 30 mentors, in a 16-week caregiver peer mentoring intervention. | LBD caregivers | 71 LBD | - Gender: Yes - Ethnicity: yes - Age: yes - SES: not stated or no relevant data - YOE: yes |
| Tailoring and evaluating the web-based ‘Partner in Balance’ intervention for family caregivers of persons with young-onset dementia [129]  **Author(s):** Jeroen Bruinsma, Kirsten Peetoom, Christian Bakker, Lizzy Boots, Joany Millenaar, Frans Verhey and Marjolein de Vugt | - 2021 - The Netherlands - Research article - Setting: Netherlands. No dates provided. | To prospectively evaluate how end-users perceive newly incorporated content in the Partner in Balance intervention in terms of usability, feasibility, acceptability, and perceptions on content. | Spouses and other family members of people with young onset dementia. | 1 LBD | - Gender: unclear - Ethnicity: not stated or no relevant data - Age: not stated or no relevant data - SES: not stated or no relevant data - YOE: not stated or no relevant data |
| Measuring disability in patients with neurodegenerative disease using the ‘Yesterday Interview’ [130]  **Author(s):** Claire L. Lomax, Richard G. Brown and Robert J. Howard | - 2004 - UK - Research article - Setting: UK. No dates provided. | To illustrate the use of time-budget methodology for measuring disability within the World Health Organization International Classification of Functioning, Disability, and Health framework in a diverse group of patients with neurodegenerative diseases. | Individuals with a range of neurodegenerative conditions and elderly subjects with no chronic neurological condition. | 12 DLB | - Gender: Yes - Ethnicity: not stated or no relevant data - Age: yes - SES: not stated or no relevant data - YOE: yes |
| “I felt like I had been put on the shelf and forgotten about” – lasting lessons about the impact of COVID-19 on people affected by rarer dementias [131]  **Author(s):** Emma Harding, Sam Rossi-Harries, Esther Vera Gerritzen, Nikki Zimmerman, Zoe Hoare, Danielle Proctor, Emilie Brotherhood, Sebastian Crutch and Aida Suárez-González | - 2023 - UK - Research article - Setting: UK. Survey was available from the 11/08/20 until 30/09/20. | To describe the impact of lockdowns on individuals affected by young-onset, non-memory-led, and inherited dementias, as well as their caregivers. | People living with young onset, non-memory-led or inherited forms of dementia supported by Rare Dementia Support, or informal care partners for someone who was. | 13 DLB | - Gender: not stated or no relevant data - Ethnicity: not stated or no relevant data - Age: not stated or no relevant data - SES: not stated or no relevant data - YOE: not stated or no relevant data |
| Caring for people with dementia with Lewy bodies and Parkinson’s dementia in UK care homes – a mixed methods study [132]  **Author(s):** G.A. Jackson, L. Newbronner, R. Chamberlain, R. Borthwick, C. Yardley and K. Boyle | - 2017 - UK - Research article - Setting: UK. Survey disseminated in January 2015. | To estimate the prevalence of DLB and PDD in care home settings and assess staff’s level of knowledge about these conditions. | Care home staff and a small number of local health care professionals involved in the care of residents with LBD | Unclear (69 care homes took part in the survey; 9 homes took part in the interview stage; n=51 care home staff interviews/focus groups) | - Gender: not stated or no relevant data - Ethnicity: not stated or no relevant data - Age: not stated or no relevant data - SES: not stated or no relevant data - YOE: not stated or no relevant data |
| A pilot study on the use of dolls for people with dementia [133]  **Author(s):** Lorna Mackenzie, Ian Andrew James, Rachel Morse, Elizabeta Mukaetova-ladinska and F. Katharina Reichelt | - 2006 - UK - Research article - Setting: UK. No dates provided. | To examine the use of dolls in two care homes. | Care home staff and key workers of people with dementia | 2 LBD  (1 individual with LBD and 1 formal care partner) | - Gender: not stated or no relevant data - Ethnicity: not stated or no relevant data - Age: not stated or no relevant data - SES: not stated or no relevant data - YOE: not stated or no relevant data |
| Presence phenomena in parkinsonian disorders: phenomenology and neuropsychological correlates [134]  **Author(s):** Erin Reckner, Lisa Cipolotti and Jennifer A.Foley | - 2020 - UK - Research article - Setting: UK. No dates provided. | (1) To examine Parkinsonian disorder patients' subjective experiences of presence phenomena, and (2) to compare those who endorse presence phenomena with those who deny it on objective measures of cognitive function. | Parkinsonian disorder patients | 7 DLB | - Gender: Yes - Ethnicity: not stated or no relevant data - Age: yes - SES: not stated or no relevant data - YOE: not stated or no relevant data |
| Conversations in dementia with Lewy bodies: resources and barriers in communication [135]  **Author(s):** Sophia Lindeberg, Nicole Müller and Christina Samuelsson | - 2023 - Sweden - Research article - Setting: Sweden. Data collection took place over two days, but no specific dates reported. | To contribute to the understanding of resources and barriers in daily communication involving individuals with DLB. | DLB patients and caregivers | 1 DLB dyad | - Gender: not stated or no relevant data - Ethnicity: not stated or no relevant data - Age: yes - SES: not stated or no relevant data - YOE: not stated or no relevant data |
| Preclinical polymodal hallucinations for 13 years before dementia with Lewy bodies [136]  **Author(s):** Carlo Abbate, Pietro Davide Trimarchi, Silvia Inglese, Niccolò Viti, Alessandra Cantatore, Lisa De Agostini, Federico Pirri, Lorenza Marino, Renzo Bagarolo, and Daniela Mar | - 2014 - Italy - Case report - Setting: Italy. Patient followed for 4 years. Interview took place in October 2004. | (1) To highlight the challenges in differentiating complex hallucinations in a patient and to assess how studying the phenomenology of hallucinations contributes to differential diagnoses, (2) to review current understandings of DLB hallucinations in the context of findings from the patient's case, and (3) to provide data supporting and refining the concept of MCI-DLB. | Person with DLB | 1 DLB | - Gender: Yes - Ethnicity: not stated or no relevant data - Age: yes - SES: not stated or no relevant data - YOE: yes |
| Raising awareness and mutual support: using the internet [137]  **Author(s):** Nada Savitch and Ken Clasper | - 2011 - UK - Non-structured, narrative reflection - Setting: UK. | Not applicable | Not applicable | Not applicable | - Not applicable |
| Parkinson’s/ Lewy body dementia: a carer’s perspective[138]  **Author(s):** Janet Stuart and Sarah Kenny | - 2010 - UK - Non-structured, narrative reflection - Setting: UK. | Not applicable | Not applicable | Not applicable | - Not applicable |
| Practical treatment of Lewy body disease in the clinic: patient and physician perspectives[139]  **Author(s):** Elisabet Londos | - 2018 - Sweden - Commentary article - Setting: Sweden | Not applicable | Not applicable | Not applicable | - Not applicable |
| My Friend Lewy [140]  **Author(s):** Christine Miller | - 2021 - Canada - Non-structured, narrative reflection - Setting: Canada. | Not applicable | Not applicable | Not applicable | - Not applicable |
| "Not stated or no relevant data": Data was either not reported or not specifically reported for individuals with DLB or for a combined sample of those on the Lewy body dementia or Lewy body disease.  **Abbreviations:** People living with dementia (PLWD); Dementia with Lewy bodies (DLB); Lewy body dementia (LBD); Parkinson’s disease (PD); Multiple system atrophy (MSA); Pure autonomic failure (PAF); Parkinson’s disease and mild cognitive impairment (PD-MCI); Parkinson’s disease dementia (PDD); Alzheimer’s disease (AD); Vascular dementia (VaD); mixed AD and vascular pathology (MD); Frontotemporal dementia (FTD); Mild cognitive impairment (MCI); Subjective cognitive impairment (SCI); Corticobasal degeneration (CBD); Corticobasal syndrome (CBS); Diffuse Lewy body disease (DLBD); Progressive supranuclear palsy (PSP); Amyotrophic lateral sclerosis and parkinsonism-dementia complex (ALS/PDC); Mild cognitive impairment due to Lewy Bodies (MCI‐LB); Vascular cognitive impairment (VCI); Prodromal Alzheimer's disease (MCI-AD); United Kingdom (UK); United States of America (USA); INdiVidualised cognitivE Stimulation Therapy study (INVEST); The Cognitive Rehabilitation for Parkinson's disease dementia: a pilot randomised controlled trial (CORD‐PD); Socioeconomic status (SES); Years of education (YOE); Quality of life (QoL); Health-related quality of life (HRQoL); Behavioural and psychological symptoms of dementia (BPSD); randomised control trial (RCT) | | | | | |

**References (full list of included sources):**

1. Armstrong MJ, Gamez N, Alliance S, Majid T, Taylor A, Kurasz AM, et al. Research priorities of caregivers and individuals with dementia with Lewy bodies: an interview study. PLoS ONE. 2020;15:e0239279. https://doi.org/:10.1371/journal.pone.0239279

2. Armstrong MJ, Alliance S, Corsentino P, Lunde A, Taylor A. Informal caregiver experiences at the end-of-life of individuals living with dementia with Lewy bodies: an interview study. Dementia (London). 2022;21:287–303. https://doi.org/:10.1177/14713012211038428

3. Larsson V, Holmbom-Larsen A, Torisson G, Strandberg EL, Londos E. Living with dementia with Lewy bodies: an interpretative phenomenological analysis. BMJ Open. 2019;9:e024983. https://doi.org/:10.1136/bmjopen-2018-024983

4. Armstrong MJ, Alliance S, Taylor A, Corsentino P, Galvin JE. End-of-life experiences in dementia with Lewy bodies: Qualitative interviews with former caregivers. PLoS One. 2019;14:e0217039. https://doi.org/:10.1371/journal.pone.0217039

5. Armstrong MJ, Alliance S, Corsentino P, Maixner SM, Paulson HL, Taylor A. Caregiver-reported barriers to quality end-of-life care in dementia with Lewy bodies: a qualitative analysis. Am J Hosp Palliat Care. 2020;37:728–37. https://doi.org/:10.1177/1049909119897241

6. Armstrong M.J., Gamez N., Alliance S., Majid T., Taylor A.S., Kurasz A.M., et al. Clinical care and unmet needs of individuals with dementia with Lewy bodies and caregivers: an interview study. Alzheimer Dis Assoc Disord. 2021;35:327–34. https://doi.org/:10.1097/WAD.0000000000000459

7. Brown LJE, Aldridge Z, Pepper A, Leroi I, Dening KH. ‘It’s just incredible the difference it has made’: Family carers’ experiences of a specialist Lewy body dementia Admiral Nurse service. Age Ageing. 2022;51:afac207. https://doi.org/:10.1093/ageing/afac207

8. Ducharme F, Kergoat M-J, Antoine P, Pasquier F, Coulombe R. The unique experience of spouses in early-onset dementia. Am J Alzheimers Dis Other Demen. 2013;28:634–41. https://doi.org/:10.1177/1533317513494443

9. Gallop K, Pham N, Maclaine G, Saunders E, Black B, Acaster S. Exploring the impact of caring for an individual with neurogenic orthostatic hypotension: a qualitative study. Neurodegener Dis Manag. 2023;13:191–201. https://doi.org/:10.2217/nmt-2022-0016

10. Stacy KE, Perazzo J, Shatz R, Bakas T. Needs and concerns of Lewy body disease family caregivers: A qualitative study. West J Nurs Res. 2022;44:227–38. https://doi.org/:10.1177/01939459211050957

11. Matterson E, Wilson-Menzfeld G, Olsen K, Taylor J-P, Elder GJ. Understanding the nature and impact of cognitive fluctuations and sleep disturbances in dementia with Lewy bodies: a qualitative caregiver study. SAGE Open Med. 2024;12:20503121241271827. https://doi.org/:10.1177/20503121241271827

12. Vatter S, McDonald KR, Stanmore E, Clare L, McCormick SA, Leroi I. A qualitative study of female caregiving spouses’ experiences of intimate relationships as cognition declines in Parkinson’s disease. Age Ageing. 2018;47:604–10. https://doi.org/:10.1093/ageing/afy049

13. Murphy C., Laine C.D., Macaulay M., Lennie K.H., Fader M. Problems faced by people living at home with dementia and incontinence: causes, consequences and potential solutions. Age Ageing. 2021;50:944–54. https://doi.org/:10.1093/ageing/afaa262

14. Park J, Howard H, Tolea MI, Galvin JE. Perceived benefits of using nonpharmacological interventions in older adults with Alzheimer’s disease or dementia with Lewy bodies. J Gerontol Nurs. 2020;46:37–46. https://doi.org/:10.3928/00989134-20191217-01

15. Yumoto A, Suwa S. Difficulties and associated coping methods regarding visual hallucinations caused by dementia with Lewy bodies. Dementia (London). 2021;20:291–307. https://doi.org/:10.1177/1471301219879541

16. Svendsboe EJ. Carers to people with Lewy body dementia and Alzheimer’s disease: Experiences and coping strategies. PhD. Thesis. [Sweden]: Karolinska Institutet; 2018.

17. Bamford C, Wheatley A, Brunskill G, Booi L, Allan L, Banerjee S, et al. Key components of post-diagnostic support for people with dementia and their carers: a qualitative study. PLoS ONE. 2021;16:e0260506. https://doi.org/:10.1371/journal.pone.0260506

18. Whitworth A, Lesser R, McKeith I. Profiling conversation in Parkinson’s disease with cognitive impairment. Aphasiology. 1999;13:407–25. https://doi.org/:10.1080/026870399402154

19. Dixon E., Anderson J., Blackwelder D.C., Radnofsky M.L., Lazar A. The human need for equilibrium: qualitative study on the ingenuity, technical competency, and changing strategies of people with dementia seeking health information. J Med Internet Res. 2022;24:e35072. https://doi.org/:10.2196/35072

20. Thomas AJ, Taylor JP, McKeith I, Bamford C, Burn D, Allan L, et al. Development of assessment toolkits for improving the diagnosis of the Lewy body dementias: feasibility study within the DIAMOND Lewy study. Int J Geriatr Psychiatry. 2017;32:1280–304. https://doi.org/:10.1002/gps.4609

21. O’Brien JT, Taylor J-P, Thomas A, Bamford C, Vale L, Hill S, et al. Improving the diagnosis and management of Lewy body dementia: the DIAMOND-Lewy research programme including pilot cluster RCT. Programme Grants Appl Res. 2021;9:1–120. https://doi.org/:10.3310/pgfar09070

22. Bentley A, Salifu Y, Walshe C. Applying an analytical process to longitudinal narrative interviews with couples living and dying with Lewy body dementia. Int J Qual Methods. 2021;20:16094069211060653. https://doi.org/:10.1177/16094069211060653

23. Morgan T., Duschinsky R., Gott M., Barclay S. Problematising carer identification: a narrative study with older partner’s providing end-of-life care. SSM- Qual Res Health. 2021;1:100015. https://doi.org/:10.1016/j.ssmqr.2021.100015

24. Heyman I, Brorsson A, Persson T, Londos E. Pacemaker implants and their influence on the daily life of patients with dementia with Lewy bodies: A qualitative case study. Neurol Ther. 2023;12:1359–73. https://doi.org/:10.1007/s40120-023-00513-5

25. Bernstein A., Merrilees J., Dulaney S., Harrison K.L., Chiong W., Ong P., et al. Using care navigation to address caregiver burden in dementia: a qualitative case study analysis. Alzheimers Dement (N Y). 2020;6:e12010. https://doi.org/:10.1002/trc2.12010

26. Pongan E., Dorey J.-M., Borg C., Getenet J.C., Bachelet R., Lourioux C., et al. COVID-19: Association between increase of behavioral and psychological symptoms of dementia during lockdown and caregivers’ poor mental health. J Alzheimer’s Dis. 2021;80:1713–21. https://doi.org/:10.3233/JAD-201396

27. Rigby T, Johnson DK, Taylor A, Galvin JE. Comparison of the caregiving experience of grief, burden, and quality of life in dementia with Lewy bodies, Alzheimer’s disease, and Parkinson’s disease dementia. J Alzheimer’s Dis. 2021;80:421–32. https://doi.org/:10.3233/JAD-201326

28. Galvin JE, Duda JE, Kaufer DI, Lippa CF, Taylor A, Zarit SH. Lewy body dementia: caregiver burden and unmet needs. Alzheimer Dis Assoc Disord. 2010;24:177–81. https://doi.org/:10.1097/WAD.0b013e3181c72b5d

29. Leggett AN, Zarit S, Taylor A, Galvin JE. Stress and burden among caregivers of patients with Lewy body dementia. Gerontologist. 2011;51:76–85. https://doi.org/:10.1093/geront/gnq055

30. Rigby T, Ashwill RT, Johnson DK, Galvin JE. Differences in the experience of caregiving between spouse and adult child caregivers in dementia with Lewy bodies. Innov Aging. 2019;3:igz027. https://doi.org/:10.1093/geroni/igz027

31. Park J, Galvin JE. Pre-loss grief in caregivers of older adults with dementia with Lewy bodies. J Alzheimer’s Dis. 2021;82:1847–59. https://doi.org/:10.3233/JAD-210616

32. Chapman K.R., Tremont G., Malloy P., Spitznagel M.B. The role of sexual disinhibition to predict caregiver burden and desire to institutionalize among family dementia caregivers. J Geriatr Psychiatry Neurol. 2020;33:42–51. https://doi.org/:10.1177/0891988719856688

33. Binnekade TT, Scherder EJA, Maier AB, Lobbezoo F, Overdorp EJ, Rhebergen D, et al. Pain in patients with different dementia subtypes, mild cognitive impairment, and subjective cognitive impairment. Pain Med. 2018;19:920–7. https://doi.org/:10.1093/pm/pnx162

34. Philippi N., Noblet V., Hamdaoui M., Soulier D., Botzung A., Ehrhard E., et al. The insula, a grey matter of tastes: A volumetric MRI study in dementia with Lewy bodies. Alzheimer’s Res Ther. 2020;12:79. https://doi.org/:10.1186/s13195-020-00645-y

35. Thomas P, Lalloué F, Preux P, Hazif‐Thomas C, Pariel S, Inscale R, et al. Dementia patients caregivers quality of life: the PIXEL study. Int J Geriatr Psychiatry. 2006;21:50–6. https://doi.org/:10.1002/gps.1422

36. Armstrong MJ, Alliance S, Corsentino P, DeKosky ST, Taylor A. Cause of death and end-of-life experiences in individuals with dementia with Lewy bodies. J Am Geriatr Soc. 2019;67:67–73. https://doi.org/:10.1111/jgs.15608

37. Toya S, Manabe Y, Hashimoto M, Yamakage H, Ikeda M. Questionnaire survey of satisfaction with medication for five symptom domains of dementia with Lewy bodies among patients, their caregivers, and their attending physicians. Psychogeriatrics. 2023;23:752–62. https://doi.org/:10.1111/psyg.12993

38. Galvin JE, Duda JE, Kaufer DI, Lippa CF, Taylor A, Zarit SH. Lewy body dementia: the caregiver experience of clinical care. Parkinsonism Relat Disord. 2010;16:388–92. https://doi.org/:10.1016/j.parkreldis.2010.03.007

39. Holden SK, Bedenfield N, Taylor AS, Bayram E, Schwilk C, Fleisher J, et al. Research priorities of individuals and caregivers with Lewy body dementia: a web-based survey. Alzheimer Dis Assoc Disord. 2023;37:50–8. https://doi.org/:10.1097/WAD.0000000000000545

40. Hashimoto M, Manabe Y, Yamaguchi T, Toya S, Ikeda M. Treatment needs of dementia with Lewy bodies according to patients, caregivers, and physicians: a cross-sectional, observational, questionnaire-based study in Japan. Alzheimers Res Ther. 2022;14:188. https://doi.org/:10.1186/s13195-022-01130-4

41. Rainero I, Bruni AC, Marra C, Cagnin A, Bonanni L, Cupidi C, et al. The impact of COVID-19 quarantine on patients with dementia and family caregivers: a nation-wide survey. Front Aging Neurosci. 2021;12:625781. https://doi.org/:10.3389/fnagi.2020.625781

42. Taylor A., Corsentino P., Peterson B., Richard I., Long A., Leverenz J.B. P4-067: The importance of educating the lewy body dementia community on risks and benefits of lumbar punctures in LBD biomarker research. Alzheimers Dement. 2018;14:1458. https://doi.org/:10.1016/j.jalz.2018.06.2470

43. Cagnin A, Di Lorenzo R, Marra C, Bonanni L, Cupidi C, Laganà V, et al. Behavioral and psychological effects of coronavirus disease-19 quarantine in patients with dementia. Front Psychiatry. 2020;11:578015. https://doi.org/:10.3389/fpsyt.2020.578015

44. Tarolli CG, Zimmerman GA, Goldenthal S, Feldman B, Berk S, Siddiqi B, et al. Video research visits for atypical parkinsonian syndromes among Fox Trial Finder participants. Neurol Clin Pract. 2020;10:7–14. https://doi.org/:10.1212/CPJ.0000000000000680

45. Killen A, Flynn D, De Brún A, O’Brien N, O’Brien J, Thomas AJ, et al. Support and information needs following a diagnosis of dementia with Lewy bodies. Int Psychogeriatr. 2016;28:495–501. https://doi.org/:10.1017/S1041610215001362

46. Shinagawa S, Adachi H, Toyota Y, Mori T, Matsumoto I, Fukuhara R, et al. Characteristics of eating and swallowing problems in patients who have dementia with Lewy bodies. Int Psychogeriatr. 2009;21:520–5. https://doi.org/:10.1017/S1041610209008631

47. Yust-Katz S., Hershkovitz R., Gurevich T., Djaldetti R. Pain in extrapyramidal neurodegenerative diseases. Clin J Pain. 2017;33:635–9. https://doi.org/:10.1097/AJP.0000000000000437

48. Park J, Tolea MI, Arcay V, Lopes Y, Galvin JE. Self-efficacy and social support for psychological well-being of family caregivers of care recipients with dementia with Lewy bodies, Parkinson’s disease dementia, or Alzheimer’s disease. Soc Work Ment Health. 2019;17:253–78. https://doi.org/:10.1080/15332985.2018.1526756

49. Wu Y-T, Clare L, Hindle JV, Nelis SM, Martyr A, Matthews FE, et al. Dementia subtype and living well: results from the Improving the experience of Dementia and Enhancing Active Life (IDEAL) study. BMC Med. 2018;16:140. https://doi.org/:10.1186/s12916-018-1135-2

50. Martin J.T., Chapman K.R., Was C., Spitznagel M.B. Factors of dementia caregiver burden differentially contribute to desire to institutionalize. J Geriatr Psychiatry Neurol. 2022;35:594–600. https://doi.org/:10.1177/08919887211036183

51. Sato H, Nakaaki S, Sato J, Shikimoto R, Furukawa TA, Mimura M, et al. Caregiver self-efficacy and associated factors among caregivers of patients with dementia with lewy bodies and caregivers of patients with alzheimer’s disease. Psychogeriatrics. 2021;21:783–94. https://doi.org/:10.1111/psyg.12740

52. Kurisu K, Terada S, Oshima E, Horiuchi M, Imai N, Yabe M, et al. Comparison of QOL between patients with different degenerative dementias, focusing especially on positive and negative affect. Int Psychogeriatr. 2016;28:1355–61. https://doi.org/:10.1017/S1041610216000491

53. Boström F, Jönsson L, Minthon L, Londos E. Patients with Lewy body dementia use more resources than those with Alzheimer’s disease. Int J Geriatr Psychiatry. 2007;22:713–9. https://doi.org/:10.1002/gps.1738

54. Lee DR, McKeith I, Mosimann U, Ghosh-Nodyal A, Thomas AJ. Examining carer stress in dementia: the role of subtype diagnosis and neuropsychiatric symptoms. Int J Geriatr Psychiatry. 2013;28:135–41. https://doi.org/:10.1002/gps.3799

55. Sabatini S, Martyr A, Ukoumunne OC, Ballard C, Collins R, Pentecost C, et al. Attitudes toward own aging and cognition among individuals living with and without dementia: Findings from the IDEAL programme and the PROTECT study. BMC Geriatr. 2022;22:641. https://doi.org/:10.1186/s12877-022-03336-5

56. Vatter S, McDonald KR, Stanmore E, Clare L, Leroi I. Multidimensional care burden in Parkinson-related dementia. J Geriatr Psychiatry Neurol. 2018;31:319–28. https://doi.org/:10.1177/0891988718802104

57. Vatter S., Stanmore E., Clare L., McDonald K.R., McCormick S.A., Leroi I. Care burden and mental ill health in spouses of people with Parkinson disease dementia and Lewy body dementia. J Geriatr Psychiatry Neurol. 2020;33:3–14. https://doi.org/:10.1177/0891988719853043

58. Marantz AG, Verghese J. Capgras’ syndrome in dementia with Lewy bodies. J Geriatr Psychiatry Neurol. 2002;15:239–41.

59. Hu W., Liu S., Wang F., Zhu H., Du X., Ma L., et al. Autonomic symptoms are predictive of dementia with Lewy bodies. Parkinsonism Relat Disord. 2022;95:1–4. https://doi.org/:10.1016/j.parkreldis.2021.11.023

60. Hynninen M.J., Breitve M.H., Rongve A., Aarsland D., Nordhus I.H. The frequency and correlates of anxiety in patients with first-time diagnosed mild dementia. Int Psychogeriatr. 2012;24:1771–8. https://doi.org/:10.1017/S1041610212001020

61. Svendsboe E, Terum T, Testad I, Aarsland D, Ulstein I, Corbett A, et al. Caregiver burden in family carers of people with dementia with Lewy bodies and Alzheimer’s disease. Int J Geriatr Psychiatry. 2016;31:1075–83. https://doi.org/:10.1002/gps.4433

62. Boström F, Jönsson L, Minthon L, Londos E. Patients with dementia with Lewy bodies have more impaired quality of life than patients with Alzheimer disease. Alzheimer Dis Assoc Disord. 2007;21:150–4. https://doi.org/:10.1097/WAD.0b013e318065c4a9

63. Mahale M., Behal P., Bewal N.M., Aggarwal V., Singhal A., Yadav A.K., et al. Study of prevalence of neuropsychiatric symptoms in elderly dementia patients. J Clin Diagnostic Res. 2021;15:14–7. https://doi.org/:10.7860/JCDR/2021/44440.14510

64. Terum TM, Testad I, Rongve A, Aarsland D, Svendsboe E, Andersen JR. The association between specific neuropsychiatric disturbances in people with Alzheimer’s disease and dementia with Lewy bodies and carer distress. Int J Geriatr Psychiatry. 2019;34:1421–8. https://doi.org/:10.1002/gps.5134

65. Tabata K, Saijo Y, Morikawa F, Naoe J, Yoshioka E, Kawanishi Y, et al. Association of premorbid personality with behavioral and psychological symptoms in dementia with Lewy bodies: Comparison with Alzheimer’s disease patients. Psychiatry Clin Neurosci. 2017;71:409–16. https://doi.org/:10.1111/pcn.12511

66. Urwyler P, Nef T, Müri R, Archibald N, Makin SM, Collerton D, et al. Visual hallucinations in eye disease and Lewy body disease. Am J Geriatr Psychiatry. 2016;24:350–8. https://doi.org/:10.1016/j.jagp.2015.10.007

67. Montagnese M, Vignando M, Collerton D, Ffytche D, Mosimann UP, Taylor J-P, et al. Cognition, hallucination severity and hallucination-specific insight in neurodegenerative disorders and eye disease. Cogn Neuropsychiatry. 2022;27:105–21. https://doi.org/:10.1080/13546805.2021.1960812

68. Onandia-Hinchado I, Diaz-Orueta U. Health related quality of life in individuals with cognitive decline and discrepancies between patients and their proxies. Arch Gerontol Geriatr. 2019;85:103914. https://doi.org/:10.1016/j.archger.2019.103914

69. Onandia-Hinchado I, Diaz-Orueta U. Health related quality of life and cognitive decline in older populations: preliminary results from NeuroDemeNPsia study. J Appl Gerontol. 2020;39:618–26. https://doi.org/:10.1177/0733464819866587

70. Liu S, Liu J, Wang X-D, Shi Z, Zhou Y, Li J, et al. Caregiver burden, sleep quality, depression, and anxiety in dementia caregivers: a comparison of frontotemporal lobar degeneration, dementia with Lewy bodies, and Alzheimer’s disease. Int Psychogeriatr. 2018;30:1131–8. https://doi.org/:10.1017/S1041610217002630

71. Kawano Y, Terada S, Takenoshita S, Hayashi S, Oshima Y, Miki T, et al. Patient affect and caregiver burden in dementia. Psychogeriatrics. 2020;20:189–95. https://doi.org/:10.1111/psyg.12487

72. Yuuki S, Hashimoto M, Koyama A, Matsushita M, Ishikawa T, Fukuhara R, et al. Comparison of caregiver burden between dementia with Lewy bodies and Alzheimer’s disease. Psychogeriatrics. 2023;23:682–9. https://doi.org/:10.1111/psyg.12978

73. Kanemoto H., Sato S., Satake Y., Koizumi F., Taomoto D., Kanda A., et al. Impact of behavioral and psychological symptoms on caregiver burden in patients with dementia with Lewy bodies. Front Psychiatry. 2021;12:753864. https://doi.org/:10.3389/fpsyt.2021.753864

74. Tsai C.-F., Huang M.-H., Lee J.-J., Jhang K.-M., Huang L.-C., Huang L.-K., et al. Factors associated with burden among male caregivers for people with dementia. J Chin Med Assoc. 2022;85:462–8. https://doi.org/:10.1097/JCMA.0000000000000704

75. De Oliveira FF, Miraldo MC, De Castro-Neto EF, De Almeida SS, Matas SLDA, Bertolucci PHF, et al. Differential associations of clinical features with cerebrospinal fluid biomarkers in dementia with Lewy bodies and Alzheimer’s disease. Aging Clin Exp Res. 2023;35:1741–52. https://doi.org/:10.1007/s40520-023-02452-5

76. Ricci M, Guidoni SV, Sepe-Monti M, Bomboi G, Antonini G, Blundo C, et al. Clinical findings, functional abilities and caregiver distress in the early stage of dementia with Lewy bodies (DLB) and Alzheimer’s disease (AD). Arch Gerontol Geriatr. 2009;49:e101–4. https://doi.org/:10.1016/j.archger.2008.10.001

77. Liu S, Jin Y, Shi Z, Huo YR, Guan Y, Liu M, et al. The effects of behavioral and psychological symptoms on caregiver burden in frontotemporal dementia, Lewy body dementia, and Alzheimer’s disease: Clinical experience in China. Aging Ment Health. 2017;21:651–7. https://doi.org/:10.1080/13607863.2016.1146871

78. Torboli D, Mioni G, Bussé C, Cagnin A, Vallesi A. Subjective experience of time in dementia with Lewy bodies during COVID-19 lockdown. Curr Psychol. 2023;42:4653–62. https://doi.org/:10.1007/s12144-021-01811-7

79. Vatter S., McDonald K.R., Stanmore E., McCormick S.A., Clare L., Leroi I. A brief psychometric and clinimetric evaluation of self-report burden and mental health measures completed by care partners of people with Parkinson’s-related dementia. Int Psychogeriatr. 2020;32:875–80. https://doi.org/:10.1017/S1041610220000605

80. Chin KS, Churilov L, Doré V, Villemagne VL, Rowe CC, Yassi N, et al. Tau in dementia with Lewy bodies. Aust N Z J Psychiatry. 2024;58:175–82. https://doi.org/:10.1177/00048674231177219

81. Machado FC, Oliveira FFD, Marin SDMC, Sampaio G, Bertolucci PHF. Correlates of neuropsychiatric and motor tests with language assessment in patients with Lewy body dementia. Arch Clin Psychiatry. 2020;47:75–81. https://doi.org/:10.1590/0101-60830000000236

82. Chin KS, Gajamange S, Desmond PM, Villemagne VL, Rowe CC, Churilov L, et al. Association between amyloid-beta deposition and cortical thickness in dementia with Lewy bodies. Aust N Z J Psychiatry. 2023;57:594–602. https://doi.org/:10.1177/00048674221081773

83. de Oliveira FF, Machado FC, Sampaio G, Marin S de MC, Naffah-Mazzacoratti M da G, Bertolucci PHF. Neuropsychiatric feature profiles of patients with Lewy body dementia. Clin Neurol Neurosurg. 2020;194:105832. https://doi.org/:10.1016/j.clineuro.2020.105832

84. Tzeng R-C, Tsai C-F, Wang C-T, Wang T-Y, Chiu P-Y. Delusions in patients with dementia with Lewy bodies and the associated factors. Behav Neurol. 2018;2018:6707291. https://doi.org/:10.1155/2018/6707291

85. Oliveira FF, Machado FC, Sampaio G, Marin SM, Chen ES, Smith MC, et al. Contrasts between patients with Lewy body dementia syndromes and APOE-ε3/ε3 patients with late-onset Alzheimer disease dementia. Neurologist. 2015;20:35–41. https://doi.org/:https://doi.org/10.1097/nrl.0000000000000045

86. Shindo A, Ueda Y, Kuzuhara S, Kokubo Y. Neuropsychological study of amyotrophic lateral sclerosis and parkinsonism-dementia complex in Kii peninsula, Japan. BMC Neurol. 2014;14:151. https://doi.org/:10.1186/1471-2377-14-151

87. Hashimoto M, Sakamoto S, Ikeda M. Clinical features of delusional jealousy in elderly patients with dementia. J Clin Psychiatry. 2015;76:691–5. https://doi.org/:10.4088/JCP.14m09018

88. Chiu P-Y, Wang C-W, Tsai C-T, Li S-H, Lin C-L, Lai T-J. Depression in dementia with Lewy bodies: A comparison with Alzheimer’s disease. PLoS ONE. 2017;12:e0179399. https://doi.org/:10.1371/journal.pone.0179399

89. Guo X., Meng Q., Zuo M., Su Z., Gan J., Li X.-D., et al. Costs of dementia with lewy bodies: A Chinese multicenter cross-sectional study. Int J Geriatr Psychiatry. 2023;38:e5848. https://doi.org/:10.1002/gps.5848

90. van de Beek M, van Steenoven I, van der Zande JJ, Porcelijn I, Barkhof F, Stam CJ, et al. Characterization of symptoms and determinants of disease burden in dementia with Lewy bodies: DEvELOP design and baseline results. Alzheimer’s Res Ther. 2021;13:53. https://doi.org/:10.1186/s13195-021-00792-w

91. Svendsboe EJ, Testad I, Terum T, Jörg A, Corbett A, Aarsland D, et al. Patterns of carer distress over time in mild dementia. Int J Geriatr Psychiatry. 2018;33:987–93. https://doi.org/:10.1002/gps.4882

92. Rongve A, Vossius C, Nore S, Testad I, Aarsland D. Time until nursing home admission in people with mild dementia: Comparison of dementia with Lewy bodies and Alzheimer’s dementia. Int J Geriatr Psychiatry. 2014;29:392–8. https://doi.org/:10.1002/gps.4015

93. van de Beek M, van Steenoven I, Ramakers IHGB, Aalten P, Koek HL, Olde Rikkert MGM, et al. Trajectories and determinants of quality of life in dementia with Lewy bodies and Alzheimer’s disease. J Alzheimer’s Dis. 2019;70:389–97. https://doi.org/:10.3233/JAD-190041

94. van De Beek M, Van Unnik A, Van Steenoven I, Van Der Zande J, Barkhof F, Teunissen CE, et al. Disease progression in dementia with Lewy bodies: a longitudinal study on clinical symptoms, quality of life and functional impairment. Int J Geriatr Psychiatry. 2022;37:1–9. https://doi.org/:10.1002/gps.5839

95. Galvin JE, Malcom H, Johnson D, Morris JC. Personality traits distinguishing dementia with Lewy bodies from Alzheimer disease. Neurology. 2007;68:1895–901. https://doi.org/:10.1212/01.wnl.0000263131.80945.ad

96. Henderson C., Knapp M., Martyr A., Gamble L.D., Nelis S.M., Quinn C., et al. The use and costs of paid and unpaid care for people with dementia: longitudinal findings from the IDEAL cohort. J Alzheimer’s Dis. 2022;86:135–53. https://doi.org/:10.3233/JAD-215117

97. Armstrong M.J., Moore K., Jacobson C.E., Bedenfield N., Patel B., Sullivan J.L. Frequency of suicidal ideation and associated clinical features in Lewy body dementia. Parkinsonism Relat Disord. 2021;90:33–7. https://doi.org/:10.1016/j.parkreldis.2021.07.029

98. Huang W-C, Chang M-C, Wang W-F, Jhang K-M. A comparison of caregiver burden for different types of dementia: an 18-month retrospective cohort study. Front Psychol. 2022;12:798315. https://doi.org/:10.3389/fpsyg.2021.798315

99. Bao X., Xu J., Meng Q., Gan J., Wang X.-D., Wu H., et al. Impact of the COVID-19 pandemic and lockdown on anxiety, depression and nursing burden of caregivers in Alzheimer’s disease, dementia with Lewy bodies and mild cognitive impairment in China: a 1-year follow-up study. Front Psychiatry. 2022;13:921535. https://doi.org/:10.3389/fpsyt.2022.921535

100. van de Beek M, van Steenoven I, van der Zande JJ, Barkhof F, Teunissen CE, van der Flier WM, et al. Prodromal dementia with Lewy bodies: Clinical characterization and predictors of progression. Mov Disord. 2020;35:859–67. https://doi.org/:10.1002/mds.27997

101. Onofrj M, Thomas A, Tiraboschi P, Wenning G, Gambi F, Sepede G, et al. Updates on somatoform disorders (SFMD) in Parkinson’s disease and dementia with Lewy bodies and discussion of phenomenology. J Neurol Sci. 2011;310:166–71. https://doi.org/:10.1016/j.jns.2011.07.010

102. Onofrj M., Bonanni L., Manzoli L., Thomas A. Cohort study on somatoform disorders in Parkinson disease and dementia with Lewy bodies. Neurology. 2010;74:1598–606. https://doi.org/:10.1212/WNL.0b013e3181df09dd

103. Emre M, Tsolaki M, Bonuccelli U, Destée A, Tolosa E, Kutzelnigg A, et al. Memantine for patients with Parkinson’s disease dementia or dementia with Lewy bodies: a randomised, double-blind, placebo-controlled trial. Lancet Neurol. 2010;9:969–77. https://doi.org/:10.1016/S1474-4422(10)70194-0

104. Garrido S., Dunne L., Stevens C.J., Chang E., Clements-Cortes A. Music playlists for people with dementia: trialing a guide for caregivers. J Alzheimer’s Dis. 2020;77:219–26. https://doi.org/:10.3233/JAD-200457

105. Gratwicke J, Zrinzo L, Kahan J, Peters A, Brechany U, McNichol A, et al. Bilateral nucleus basalis of Meynert deep brain stimulation for dementia with Lewy bodies: a randomised clinical trial. Brain Stimul. 2020;13:1031–9. https://doi.org/:10.1016/j.brs.2020.04.010

106. Murata M, Odawara T, Hasegawa K, Iiyama S, Nakamura M, Tagawa M, et al. Adjunct zonisamide to levodopa for DLB parkinsonism: a randomized double-blind phase 2 study. Neurology. 2018;90:e664–72. https://doi.org/:10.1212/WNL.0000000000005010

107. Mori E, Ikeda M, Kosaka K, Donepezil-DLB Study Investigators. Donepezil for dementia with Lewy bodies: A randomized, placebo-controlled trial. Ann Neurol. 2012;72:41–52. https://doi.org/:10.1002/ana.23557

108. Larsson V, Engedal K, Aarsland D, Wattmo C, Minthon L, Londos E. Quality of life and the effect of memantine in dementia with Lewy bodies and Parkinson’s disease dementia. Dement Geriatr Cogn Disord. 2011;32:227–34. https://doi.org/:10.1159/000334523

109. Mori E, Ikeda M, Nagai R, Matsuo K, Nakagawa M, Kosaka K. Long-term donepezil use for dementia with Lewy bodies: Results from an open-label extension of Phase III trial. Alzheimer’s Res Ther. 2015;7:5. https://doi.org/:10.1186/s13195-014-0081-2

110. Ikeda M, Mori E, Orimo S, Yamada T, Konishi O. Efficacy of adjunctive therapy with zonisamide versus increased dose of levodopa for motor symptoms in patients with dementia with Lewy bodies: the randomized, controlled, non-inferiority DUEL study. J Alzheimer’s Dis. 2023;95:251–64. https://doi.org/:10.3233/JAD-230335

111. Hindle JV, Watermeyer TJ, Roberts J, Brand A, Hoare Z, Martyr A, et al. Goal‐orientated cognitive rehabilitation for dementias associated with Parkinson’s disease―a pilot randomised controlled trial. Int J Geriatr Psychiatry. 2018;33:718–28. https://doi.org/:10.1002/gps.4845

112. Lapid MI, Kuntz KM, Mason SS, Aakre JA, Lundt ES, Kremers W, et al. Efficacy, safety, and tolerability of armodafinil therapy for hypersomnia associated with dementia with Lewy bodies: a pilot study. Dement Geriatr Cogn Disord. 2017;43:269–80. https://doi.org/:10.1159/000471507

113. Thomas AJ, Burn DJ, Rowan EN, Littlewood E, Newby J, Cousins D, et al. A comparison of the efficacy of donepezil in Parkinson’s disease with dementia and dementia with Lewy bodies. Int J Geriatr Psychiatry. 2005;20:938–44. https://doi.org/:10.1002/gps.1381

114. Ikeda M, Mori E, Kosaka K, Iseki E, Hashimoto M, Matsukawa N, et al. Long-term safety and efficacy of donepezil in patients with dementia with Lewy bodies: results from a 52-week, open-label, multicenter extension study. Dement Geriatr Cogn Disord. 2013;36:229–41. https://doi.org/:10.1159/000351672

115. O’Brien JT, McKeith IG, Thomas AJ, Bamford C, Vale L, Hill S, et al. Introduction of a management toolkit for Lewy body dementia: a pilot cluster‐randomized trial. Mov Disord. 2021;36:143–51. https://doi.org/:10.1002/mds.28282

116. Bied AM, Njuguna SW, Satodiya RM. Lumateperone for treatment of psychotic symptoms in Lewy body disease: a case report. Exp Clin Psychopharmacol. 2024;32:136–9. https://doi.org/:10.1037/pha0000663

117. Rice T, Dobry Y, Wang E, Novakovic V, Sher L. Cognitive effects of quetiapine in a patient with dementia with Lewy bodies. Psychogeriatrics. 2013;13:52–7. https://doi.org/:10.1111/j.1479-8301.2012.00414.x

118. Iwasaki K, Kosaka K, Mori H, Okitsu R, Furukawa K, Manabe Y, et al. Open label trial to evaluate the efficacy and safety of yokukansan, a traditional Asian medicine, in dementia with Lewy bodies. J Am Geriatr Soc. 2011;59:936–8. https://doi.org/:10.1111/j.1532-5415.2011.03373.x

119. Iwasaki K, Kosaka K, Mori H, Okitsu R, Furukawa K, Manabe Y, et al. Improvement in delusions and hallucinations in patients with dementia with Lewy bodies upon administration of yokukansan, a traditional Japanese medicine. Psychogeriatrics. 2012;12:235–41. https://doi.org/:10.1111/j.1479-8301.2012.00413.x

120. Kasanuki K, Iseki E, Nishida Y, Fujishiro H, Chiba Y, Sato K, et al. Effectiveness of ramelteon for treatment of visual hallucinations in dementia with Lewy bodies: a report of 4 cases. J Clin Psychopharmacol. 2013;33:581–3. https://doi.org/:10.1097/JCP.0b013e318295fdf4

121. Odawara T, Shiozaki K, Togo T, Hirayasu Y. Administration of zonisamide in three cases of dementia with Lewy bodies. Psychiatry Clin Neurosci. 2010;64:327–9. https://doi.org/:10.1111/j.1440-1819.2010.02075.x

122. Shea C, MacKnight C, Rockwood K. Donepezil for treatment of dementia with Lewy bodies: A case series of nine patients. Int Psychogeriatr. 1998;10:229–38. https://doi.org/:10.1017/S1041610298005341

123. Stacy KE, Lambert J, Shatz R, Bakas T. Development and validation of the Lewy body disease caregiver activities scale. J Nurs Meas. 2023;31:606–14. https://doi.org/:10.1891/JNM-2021-0100

124. Watermeyer T, Hindle J, Roberts J, Lawrence C, Martyr A, Lloyd-Williams H, et al. Goal setting for cognitive rehabilitation in mild to moderate Parkinson’s disease dementia and dementia with Lewy bodies. Parkinsons Dis. 2016;2016:8285041. https://doi.org/:10.1155/2016/8285041

125. Leroi I, Vatter S, Carter L-A, Smith SJ, Orgeta V, Poliakoff E, et al. Parkinson’s-adapted cognitive stimulation therapy: a pilot randomized controlled clinical trial. Ther Adv Neurol Disord. 2019;12:1756286419852217. https://doi.org/:10.1177/1756286419852217

126. McCormick SA, Vatter S, Carter L-A, Smith SJ, Orgeta V, Poliakoff E, et al. Parkinson’s-adapted cognitive stimulation therapy: feasibility and acceptability in Lewy body spectrum disorders. J Neurol. 2019;266:1756–70. https://doi.org/:10.1007/s00415-019-09329-6

127. Killen A., Flynn D., O’Brien N., Taylor J.-P. The feasibility and acceptability of a psychosocial intervention to support people with dementia with Lewy bodies and family care partners. Dementia (London). 2022;21:77–93. https://doi.org/:10.1177/14713012211028501

128. Fleisher J, Suresh M, Levin M, Hess S, Akram F, Dodson D, et al. Learning to PERSEVERE: a pilot study of peer mentor support and caregiver education in Lewy body dementia. Parkinsonism Relat Disord. 2023;113:105492. https://doi.org/:10.1016/j.parkreldis.2023.105492

129. Bruinsma J., Peetoom K., Bakker C., Boots L., Millenaar J., Verhey F., et al. Tailoring and evaluating the web-based ‘Partner in Balance’ intervention for family caregivers of persons with young-onset dementia. Internet Interv. 2021;25:100390. https://doi.org/:10.1016/j.invent.2021.100390

130. Lomax CL, Brown RG, Howard RJ. Measuring disability in patients with neurodegenerative disease using the ‘Yesterday Interview’. Int J Geriatr Psychiatry. 2004;19:1058–64. https://doi.org/:10.1002/gps.1210

131. Harding E, Rossi-Harries S, Gerritzen EV, Zimmerman N, Hoare Z, Proctor D, et al. ‘I felt like I had been put on the shelf and forgotten about’ - lasting lessons about the impact of COVID-19 on people affected by rarer dementias. BMC Geriatr. 2023;23:392. https://doi.org/:10.1186/s12877-023-03992-1

132. Jackson G.A., Newbronner L., Chamberlain R., Borthwick R., Yardley C., Boyle K. Caring for people with dementia with Lewy bodies and Parkinson’s dementia in UK care homes - a mixed methods study. Eur Geriatr Med. 2017;8:146–52. https://doi.org/:10.1016/j.eurger.2017.01.003

133. Mackenzie L., James I.A., Morse R., Mukaetova-Ladinska E., Reichelt F.K. A pilot study on the use of dolls for people with dementia. Age Ageing. 2006;35:441–4. https://doi.org/:10.1093/ageing/afl007

134. Reckner E., Cipolotti L., Foley J.A. Presence phenomena in parkinsonian disorders: Phenomenology and neuropsychological correlates. Int J Geriatr Psychiatry. 2020;35:785–93. https://doi.org/:10.1002/gps.5303

135. Lindeberg S., Muller N., Samuelsson C. Conversations in dementia with Lewy bodies: Resources and barriers in communication. Int J Lang Commun Disord. 2023;58:419–32. https://doi.org/:10.1111/1460-6984.12799

136. Abbate C, Trimarchi PD, Inglese S, Viti N, Cantatore A, De Agostini L, et al. Preclinical polymodal hallucinations for 13 years before dementia with Lewy bodies. Behav Neurol. 2014;2014:694296. https://doi.org/:10.1155/2014/694296

137. Clasper K, Savitch N. Raising awareness and mutual support: using the internet. J Dement Care. 2011;19:30–2.

138. Stuart J. Parkinson’s/Lewy body dementia: a carer’s perspective. J Dement Care. 2010;18:18–22.

139. Londos E. Practical treatment of Lewy body disease in the clinic: patient and physician perspectives. Neurol Ther. 2018;7:13–22. https://doi.org/:10.1007/s40120-017-0090-8

140. Miller C. My Friend Lewy. J Am Geriatr Soc. 2021;69:1399–400. https://doi.org/:10.1111/jgs.17084

**Appendix 6:** Charted data on care partner relationships

| **Study characteristics** | **Care Partner Relationship** | **Categorisation of care partner relationship** |
| --- | --- | --- |
| ‘It's just incredible the difference it has made': family carers' experiences of a specialist Lewy body dementia Admiral Nurse service | - n=9 married spouses - n=4 sons or daughters - n=1 sibling | Spouse Child Sibling |
| “I felt like I had been put on the shelf and forgotten about” – lasting lessons about the impact of COVID-19 on people affected by rarer dementias | - 6 DLB carers lived with the PLWD - 4 DLB carers were carers to those in care homes. | Relationship not specified |
| A brief psychometric and clinimetric evaluation of self-report burden and mental health measures completed by care partners of people with Parkinson's-related dementia | For the full sample (PD-MCI/ PDD/ DLB), 94.9% were spouses. | Spouse Other |
| A comparison of caregiver burden for different types of dementia: An 18-month retrospective cohort study | For the whole sample (PD-MCI/PDD/DLB) at baseline:   - Spouse: n= 111 (17.6%) - Children: 369 (58.6%) - Other: 150 (23.8%) | Spouse Child Other |
| A comparison of the efficacy of donepezil in Parkinson’s disease with dementia and dementia with Lewy bodies | Not reported | Not reported |
| A pilot study on the use of dolls for people with dementia | Formal care partners | Formal care partners |
| A qualitative study of female caregiving spouses’ experiences of intimate relationships as cognition declines in Parkinson’s disease | n=3 DLB spousal caregivers | Spouse |
| Adjunct zonisamide to levodopa for DLB parkinsonism: a randomized double-blind phase 2 study | Not reported | Not reported |
| Administration of zonisamide in three cases of dementia with Lewy bodies | Not reported | Not reported |
| Applying an analytical process to longitudinal narrative interviews with couples living and dying with Lewy body dementia | Spouse | Spouse |
| Association between amyloid-beta deposition and cortical thickness in dementia with Lewy bodies | Not reported | Not reported |
| Association of premorbid personality with behavioral and psychological symptoms in dementia with Lewy bodies: comparison with Alzheimer’s disease patients | No DLB-specific data | No relevant data |
| Attitudes toward own aging and cognition among individuals living with and without dementia: findings from the IDEAL programme and the PROTECT study | In the IDEAL cohort, carers were the designated primary carers of PLWD who met inclusion criteria. | Not reported |
| Autonomic symptoms are predictive of dementia with Lewy bodies | Not reported | Not reported |
| Behavioral and psychological effects of coronavirus disease-19 quarantine in patients with dementia | • 43.1% spouse (n=154)  • 48.7% sons or daughters (n=140) • 8.2% other (n=29) | Spouse  Child Other |
| Bilateral nucleus basalis of Meynert deep brain stimulation for dementia with Lewy bodies: a randomised clinical trial | Not reported | Not reported |
| Capgras syndrome in dementia with Lewy bodies | • Spouses 69% • Adult children 24% • Paid caregivers 7% | Spouse Child Formal care partner |
| Care burden and mental ill health in spouses of people with Parkinson disease dementia and Lewy body dementia | 94.9% spouses  Other | Spouse Other |
| Caregiver burden in family carers of people with dementia with Lewy bodies and Alzheimer’s disease | - n=53 (61.6%) spouse - n= 33 (38.4%) children | Spouse Child |
| Caregiver burden, sleep quality, depression, and anxiety in dementia caregivers: a comparison of frontotemporal lobar degeneration, dementia with Lewy bodies, and Alzheimer’s disease | - n=21 (57.69%) spouse - n=15 (42.31%) child | Spouse Child |
| Caregiver self-efficacy and associated factors among caregivers of patients with dementia with Lewy bodies and caregivers of patients with Alzheimer’s disease | - n=18 spouse - n=28 child | Spouse Child |
| Caregiver-reported barriers to quality end-of-life care in dementia with Lewy bodies: a qualitative analysis | - n=13 daughters - n=11 wives - n=1 sister - n=1 daughter-in-law - n=1 niece - n=2 husbands - n=1 son | Child Spouse Sibling  Child-in-law Niece |
| Carers to people with Lewy body dementia and Alzheimer's disease: experiences and coping strategies | - Spouses - Adult children - Grandchildren | Spouses Child Grandchild |
| Caring for people with dementia with Lewy bodies and Parkinson’s dementia in UK care homes – a mixed methods study | Formal care partners | Formal care partner |
| Cause of death and end-of-life experiences in individuals with dementia with Lewy bodies | - 42% spouse or partner - 53% child - 6% other | Spouse Child Other |
| Characteristics of eating and swallowing problems in patients who have dementia with Lewy bodies | Not reported | Not reported |
| Characterization of symptoms and determinants of disease burden in dementia with Lewy bodies: DEvELOP design and baseline results | Not reported | Not reported |
| Clinical care and unmet needs of individuals with dementia with Lewy bodies and caregivers: an interview study | - n=20 wives - n=3 husbands - n=2 daughters | Spouse Child |
| Clinical features of delusional jealousy in elderly patients with dementia | All spouses | Spouse |
| Clinical findings, functional abilities and caregiver distress in the early stage of dementia with Lewy bodies (DLB) and Alzheimer’s disease (AD) | Not reported | Not reported |
| Cognition, hallucination severity and hallucination-specific insight in neurodegenerative disorders and eye disease | Not applicable (No caregiver data) | Not applicable |
| Cognitive effects of quetiapine in a patient with dementia with Lewy bodies | Not reported | Not reported |
| Cohort study on somatoform disorders in Parkinson disease and dementia with Lewy bodies | Not reported | Not reported |
| Comparison of caregiver burden between dementia with Lewy bodies and Alzheimer’s disease | - n=45 (48.4%) spouse - n=39 (41.9%) child - n=9 (9.7%) other | Spouse Child Other |
| Comparison of QOL between patients with different degenerative dementias, focusing especially on positive and negative affect | - Partner: n= 19 (67.9%) - Child: n= 7 (25.0%) - Other: n= 2 (7.1%) | Partner Child Other |
| Comparison of the caregiving experience of grief, burden, and quality of life in dementia with Lewy bodies, Alzheimer’s disease, and Parkinson’s disease dementia | Spouse: 62.5%  Other | Spouse Other |
| Contrasts between patients with Lewy body dementia syndromes and APOE- ε3/ε3 patients with late-onset Alzheimer disease dementia | Not reported | Not reported |
| Conversations in dementia with Lewy bodies: Resources and barriers in communication | n=1 spouse | Spouse |
| Correlates of neuropsychiatric and motor tests with language assessment in patients with Lewy body dementia | Not reported | Not reported |
| Costs of dementia with Lewy bodies: a Chinese multicenter cross-sectional study | Not reported | Not reported |
| COVID-19: association between increase of behavioral and psychological symptoms of dementia during lockdown and caregivers’ poor mental health | No LBD specific data | No relevant data |
| Delusions in patients with dementia with Lewy bodies and the associated factors | Not reported | Not reported |
| Dementia patients caregivers quality of life: the PIXEL study | No DLB/LBD-specific data | No relevant data |
| Dementia subtype and living well: results from the Improving the experience of Dementia and Enhancing Active Life (IDEAL) study | Carers were the designated primary carers of people with dementia who met inclusion criteria. Carers were considered to be someone who looks after a relative or friend and provides practical or emotional unpaid support | Not reported |
| Depression in dementia with Lewy bodies: a comparison with Alzheimer’s disease | Not reported | Not reported |
| Development and validation of the Lewy body disease caregiver activities scale | Not reported | Not reported |
| Differences in the experience of caregiving between spouse and adult child caregivers in dementia with Lewy bodies | - n= 255 spouse - n= 160 adult child | Spouse Child |
| Differential associations of clinical features with cerebrospinal fluid biomarkers in dementia with Lewy bodies and Alzheimer’s disease | Not reported | Not reported |
| Difficulties and associated coping methods regarding visual hallucinations caused by dementia with Lewy bodies | - Spouses: 8/14 (57.1%) - Children: 5/14 (35.7%) - Child-in-law: 1/14 (7.1) | Spouse Child Child-in-law |
| Disease progression in dementia with Lewy bodies: a longitudinal study on clinical symptoms, quality of life and functional impairment | Not reported | Not reported |
| Donepezil for dementia with Lewy bodies: a randomized, placebo-controlled trial | Not reported | Not reported |
| Donepezil for treatment of dementia with Lewy bodies: a case series of nine patients | All family members | Relationship not specified |
| Effectiveness of ramelteon for treatment of visual hallucinations in dementia with Lewy bodies a report of 4 cases | Not reported | Not reported |
| Efficacy of adjunctive therapy with zonisamide versus increased dose of levodopa for motor symptoms in patients with dementia with Lewy bodies: the randomized, controlled, non-inferiority DUEL study | Not reported | Not reported |
| Efficacy, safety, and tolerability of armodafinil therapy for hypersomnia associated with dementia with Lewy bodies: a pilot study | - Spouses - Other | Spouses Other |
| End-of-life experiences in dementia with Lewy bodies: qualitative interviews with former caregivers | - n=13 daughters - n=11 wives - n=1 sister - n=1 daughter-in-law - n=1 niece - n=2 husbands - n=1 son | Child Spouse Sibling Child-in-law Niece |
| Examining carer stress in dementia: the role of subtype diagnosis and neuropsychiatric symptoms | Primary caregivers | Relationship not specified |
| Exploring the impact of caring for an individual with neurogenic orthostatic hypotension: a qualitative study | No DLB-specific data | No relevant data |
| Factors associated with burden among male caregivers for people with dementia | NO LBD-specific data | No relevant data |
| Factors of dementia caregiver burden differentially contribute to desire to institutionalize | NO LBD-specific data | No relevant data |
| Frequency of suicidal ideation and associated clinical features in Lewy body dementia | Not reported- database did not capture whether intake forms were patient- or caregiver-completed | Not reported |
| Goal setting for cognitive rehabilitation in mild to moderate Parkinson’s disease dementia and dementia with Lewy bodies | For whole LBD carer sample:   - Spouses/partners of the person with PDD or DLB (84.6%) - Adult children of the person with PDD or DLB (15.4%) | Spouse/partner Child |
| Goal-orientated cognitive rehabilitation for dementias associated with Parkinson's disease- a pilot randomised controlled trial | For all LBD carers:   - n=23 spouse - n=3 child | Spouse Child |
| Health related quality of life and cognitive decline in older populations: preliminary results from NeuroDemeNPsia Study | Not reported | Not reported |
| Health related quality of life in individuals with cognitive decline and discrepancies between patients and their proxies | Not reported | Not reported |
| Impact of behavioral and psychological symptoms on caregiver burden in patients with dementia with Lewy bodies | Not reported | Not reported |
| Impact of the COVID-19 pandemic and lockdown on anxiety, depression and nursing burden of caregivers in Alzheimer’s disease, dementia with Lewy bodies and mild cognitive impairment in China: a 1-year follow-up study | - n=14 spouses (63.64%) - n=8 child (36.36%) | Spouse Child |
| Improvement in delusions and hallucinations in patients with dementia with Lewy bodies upon administration of yokukansan, a traditional Japanese medicine | Not reported | Not reported |
| Improving the diagnosis and management of Lewy body dementia: the DIAMOND-Lewy research programme including pilot cluster RCT | Not reported | Not reported |
| Informal caregiver experiences at the end-of-life of individuals living with dementia with Lewy bodies: an interview study | - n=15 children - n=13 spouses - n=2 other family members | Child Spouse Relationship not specified |
| Key components of post-diagnostic support for people with dementia and their carers: a qualitative study | - n=1 spouse but others not reported | Spouse Not reported |
| Learning to PERSEVERE: A pilot study of peer mentor support and caregiver education in Lewy body dementia | Focus group:   - Spouse/domestic partner: 75% - Parent: 15% - Sibling: 0 - Extended family member: 5%   Mentees:   - Spouse/domestic partner: 81.25% - Parent: 15.63% - Sibling: 0 - Extended family member: 3.125%   Mentors:   - Spouse/domestic partner: 71.05% - Parent: 23.68% - Sibling: 2.63% - Extended family member: 2.63% | Spouse/partner Parent Sibling Relationship not specified |
| Lewy body dementia: caregiver burden and unmet needs | - Spouse: 40.6% - Child: 51.7% - Other relative: 3.8% - Friend: 3.8% | Spouse Child Relationship not specified Friend |
| Lewy body dementia: the caregiver experience of clinical care | - 44% spouse - Other | Spouse Other |
| Living with dementia with Lewy bodies: an interpretative phenomenological analysis | Not applicable | Not applicable |
| Long-term donepezil use for dementia with Lewy bodies: results from an open-label extension of Phase III trial | Not reported | Not reported |
| Long-term safety and efficacy of donepezil in patients with dementia with Lewy bodies: results from a 52-week, open-label, multicenter extension study | Not reported | Not reported |
| Lumateperone for treatment of psychotic symptoms in Lewy body: a case report | Not reported | Not reported |
| Measuring disability in patients with neurodegenerative disease using the ‘Yesterday Interview’ | Not reported | Not reported |
| Memantine for patients with Parkinson’s disease dementia or dementia with Lewy bodies: a randomised, double-blind, placebo-controlled trial | Not reported | Not reported |
| Multidimensional care burden in Parkinson-related dementia | Life partners | Partner |
| Music playlists for people with dementia: trialling a guide for caregivers | Not reported | Not reported |
| My friend Lewy | Child | Child |
| Needs and concerns of Lewy body disease family caregivers: a qualitative study | - 85% spouses - 5% friend - 5% daughter - 5% sister | Spouse  Friend Child Sibling |
| Neuropsychiatric feature profiles of patients with Lewy body dementia | Not reported | Not reported |
| Neuropsychological study of amyotrophic lateral sclerosis and parkinsonism-dementia complex in Kii peninsula, Japan | Not reported | Not reported |
| Open label trial to evaluate the efficacy and safety of Yokukansan, a traditional Asian medicine, in dementia with Lewy bodies | Not reported | Not reported |
| Pacemaker implants and their influence on the daily life of patients with dementia with Lewy bodies: a qualitative case study | n=2 spouses | Spouse |
| Pain in extrapyramidal neurodegenerative diseases | Not applicable | Not applicable |
| Pain in patients with different dementia subtypes, mild cognitive impairment, and subjective cognitive impairment | Not applicable | Not applicable |
| Parkinson’s/ Lewy body dementia: a carer’s perspective | Partner | Partner |
| Parkinson’s-adapted cognitive stimulation therapy: a pilot randomized controlled clinical trial | For the full PD-MCI/PDD/DLB sample:   - n=59 spouse/partner (77.6%) - n=13 Son/daughter (17.1%) - n=1 grandchild - n=1 friend - n=1 live-in-carer - n=1 divorcee | Spouse/partner Child Grandchild Friend Formal care partner Divorcee |
| Parkinson’s-adapted cognitive stimulation therapy: Feasibility and acceptability in Lewy body spectrum disorders | For the full sample (PD-MCI/PDD/DLB):   - n=59 spouses or partners (77.6%) - n=13 relatives (17.1%) - n=1 live-in-carer - n=1 live-in divorcee - n=1 friend - n=1 grandchild | Spouse/partner Relationship not specified Formal care partner Divorcee Friend Grandchild |
| Patient affect and caregiver burden in dementia | - n=17 spouse - n=7 child - n=2 others | Spouse Child Other |
| Patients with dementia with Lewy bodies have more impaired quality of life than patients with Alzheimer disease | - n=20 spouses - n=11 child - n=3 sibling   (15 carers lived with patient) | Spouse Child Sibling |
| Patients with Lewy body dementia use more resources than those with Alzheimer’s disease | - n=20 spouses - n=11 children - n=3 siblings | Spouse Child Sibling |
| Patterns of carer distress over time in mild dementia | n=38 spouse | Spouse |
| Perceived benefits of using nonpharmacological interventions in older adults with Alzheimer’s disease or dementia with Lewy bodies | n=1 niece | Niece |
| Personality traits distinguishing dementia with Lewy bodies from Alzheimer disease | “Usually spouse or a close family member" | Spouse Relationship not specified |
| Practical treatment of Lewy body disease in the clinic: patient and physician perspectives | Spouse/partner | Spouse |
| Preclinical polymodal hallucinations for 13 years before dementia with Lewy bodies | Not applicable | Not applicable |
| Pre-loss grief in caregivers of older adults with dementia with Lewy bodies | Specific relationships referred to but not reported in manuscript | Relationship not specified |
| Presence phenomena in parkinsonian disorders: phenomenology and neuropsychological correlates | Not applicable | Not applicable |
| Problematising carer identification: a narrative study with older partner's providing end-of-life care | 2 spouses | Spouse |
| Problems faced by people living at home with dementia and incontinence: causes, consequences and potential solutions | 2 spouses | Spouse |
| Prodromal dementia with Lewy bodies: clinical characterization and predictors of progression | Not reported | Not reported |
| Profiling conversation in Parkinson’s disease with cognitive impairment | For DLB subgroup:   - n=1 daughter - n=3 wives - n=1 son - n=1 husband | Child Spouse |
| Quality of life and the effect of memantine in dementia with Lewy bodies and Parkinson’s disease dementia | Not reported | Not reported |
| Questionnaire survey of satisfaction with medication for five symptom domains of dementia with Lewy bodies among patients, their caregivers, and their attending physicians | - n=139 spouse (52.9%) - n=4 sibling (1.5%) - n=104 sons or daughters (39.5%) - n=12 son-or-daughter-in-law (4.6%) - n=2 grandchild (0.8%) - n=2 care provider (0.8%) | Spouse Sibling Child-in-law Child Grandchild Formal care partner |
| Raising awareness and mutual support: using the internet | Not applicable | Not applicable |
| Research priorities of caregivers and individuals with dementia with Lewy bodies: an interview study | - n=23 spouse (92%) (20 of these were wives - n= 2 child (8%) | Spouse Child |
| Research priorities of individuals and caregivers with Lewy body dementia a web-based survey | Full LBD sample:   - 66.3% (n= 550) current caregivers - 33.7% (n=280) were former caregivers - Spouse or partner: 63% - Child (son or daughter): 26.6% - Brother or sister: 1.7% - Friend: 0.4% - Other: 8.3% | Spouse/partner Child Sibling Friend Other |
| Self-efficacy and social support for psychological well-being of family caregivers of care recipients with dementia with Lewy bodies, Parkinson’s disease dementia, or Alzheimer’s disease | No DLB-specific data | No relevant data |
| Stress and burden among caregivers of patients with Lewy body dementia | - Wife (35.8%) - Husband (6.2%) - Daughter/daughter-in-law (43%) - Son/son-in-law (5.5%) - Other (9.3%) | Spouse  Child Child-in-law Other |
| Study of prevalence of neuropsychiatric section symptoms in elderly dementia patients | No LBD-specific data. | No relevant data |
| Subjective experience of time in dementia with Lewy bodies during COVID-19 lockdown | Not reported | Not reported |
| Support and information needs following a diagnosis of dementia with Lewy bodies | - 68% child - 17.6% spouses - 12% sisters, sons/daughters in law and grandchildren | Child Spouse Child-in-law Sibling-in-law Grandchild |
| Tailoring and evaluating the web-based ‘Partner in Balance’ intervention for family caregivers of persons with young-onset dementia | Brother | Sibling |
| Tau in dementia with Lewy bodies | Not reported | Not reported |
| The association between specific neuropsychiatric disturbances in people with Alzheimer's disease and dementia with Lewy bodies and carer distress | - n=32 spouse - n=31 adult child | Spouse Child |
| The effects of behavioral and psychological symptoms on caregiver burden in frontotemporal dementia, Lewy body dementia, and Alzheimer's disease: clinical experience in China | - n=3 son (13.6%) - n=8 daughter (36.4%) - n=4 husband (18.2%) - n=7 wife (31.8%) | Child Spouse |
| The feasibility and acceptability of a psychosocial intervention to support people with dementia with Lewy bodies and family care partners | Spouse: n=13 Daughter: n=2 Brother: n=1 | Spouse Child Sibling |
| The frequency and correlates of anxiety in patients with first-time diagnosed mild dementia | Not reported | Not reported |
| The human need for equilibrium: qualitative study on the ingenuity, technical competency, and changing strategies of people with dementia seeking health information | Not applicable | Not applicable |
| The impact of COVID-19 quarantine on patients with dementia and family caregivers: A nation-wide survey | - Spouses: 43.1% - Son/daughter: 48.7% - Others: 8.2% | Spouse Child Other |
| The importance of educating the Lewy body dementia community on risks and benefits of lumbar punctures in LBD biomarker research | Not reported | Not reported |
| The insula, a grey matter of tastes: a volumetric MRI study in dementia with Lewy bodies | Not applicable | Not applicable |
| The role of sexual disinhibition to predict caregiver burden and desire to institutionalize among family dementia caregivers | No relevant data | No relevant data |
| The unique experience of spouses in early-onset dementia | 1 Spouse | Spouse |
| The use and costs of paid and unpaid care for people with dementia: Longitudinal findings from the IDEAL cohort | No DLB specific data | No relevant data |
| Time until nursing home admission in people with mild dementia: comparison of dementia with Lewy bodies and Alzheimer’s dementia | Not reported | Not reported |
| Trajectories and determinants of quality of life in dementia with Lewy bodies and Alzheimer’s disease | Not applicable | Not applicable |
| Treatment needs of dementia with Lewy bodies according to patients, caregivers, and physicians: A cross-sectional, observational questionnaire-based study in Japan | - n=139 spouse (52.9%) - n=4 sibling (1.5%) - n=104 sons or daughters (39.5%) - n=12 son-or-daughter-in-law (4.6%) - n=2 grandchild (0.8%) - n=2 care provider (0.8%) | Spouse Sibling Child-in-law Child Grandchild Formal care partner |
| Understanding the nature and impact of cognitive fluctuations and sleep disturbances in dementia with Lewy bodies: a qualitative caregiver study | n=7 spouse | Spouse |
| Updates on somatoform disorders (SFMD) in Parkinson's disease and dementia with Lewy bodies and discussion of phenomenology | Not reported | Not reported |
| Using care navigation to address caregiver burden in dementia: a qualitative case study analysis | Formal care partners  Daughter | Formal care partners  Child |
| Video research visits for atypical parkinsonian syndromes among Fox Trial Finder participants | Not applicable | Not applicable |
| Visual hallucinations in eye disease and Lewy body disease | Not applicable | Not applicable |
| Development of assessment toolkits for improving the diagnosis of the Lewy body dementias: feasibility study within the DIAMOND Lewy study | Not reported | Not reported |
| Introduction of a management toolkit for Lewy body dementia: a pilot cluster-randomized trial | For the full LBD sample:   - n=87 spouses/partners - n=20 children/children in law - n=4 siblings - n=4 other family members - n=6 friends - n=5 paid carers | Spouse/Partner Child Child-in-law Sibling Relationship not specified Friend Formal care partner |
| “No relevant data”: data was not reported for individuals with DLB or for a combined sample of those with Lewy body dementia or Lewy body disease  **Abbreviations:** People living with dementia (PLWD); Dementia with Lewy bodies (DLB); Parkinson’s disease dementia (PPD); Parkinson’s disease with mild cognitive impairment (PD-MCI) | | |

**Appendix 7:** Methods of determining diagnosis

| **Source title** | **Method of determining diagnosis** | **Categorisation of methods for determining diagnosis** |
| --- | --- | --- |
| ‘It's just incredible the difference it has made': family carers' experiences of a specialist Lewy body dementia Admiral Nurse service | Unclear | Unclear |
| “I felt like I had been put on the shelf and forgotten about” – lasting lessons about the impact of COVID-19 on people affected by rarer dementias | Individuals self-reported their/their loved one’s diagnosis | self-report |
| A brief psychometric and clinimetric evaluation of self-report burden and mental health measures completed by care partners of people with Parkinson's-related dementia | If recruited through route 1 (memory and movement disorder clinics), the clinician made the diagnosis. If recruited through route 2, spouses self-reported the diagnosis of the care recipient. | clinician(s) Self-report |
| A comparison of caregiver burden for different types of dementia: an 18-month retrospective cohort study | Neurologists and psychiatric specialists made the dementia diagnosis through clinical interviews and biomarkers, including neuropsychological tests and brain images. The diagnosis of each dementia was established according to the international consensus criteria. | Clinician(s) |
| A comparison of the efficacy of donepezil in Parkinson’s disease with Dementia and Dementia with Lewy bodies | clinician | clinician(s) |
| A pilot study on the use of dolls for people with dementia | Unclear | unclear |
| A qualitative study of female caregiving spouses’ experiences of intimate relationships as cognition declines in Parkinson’s disease | Clinician | clinician(s) |
| Adjunct zonisamide to levodopa for DLB parkinsonism: a randomized double-blind phase 2 study | Clinician | clinician(s) |
| Administration of zonisamide in three cases of dementia with Lewy bodies | Clinician | clinician(s) |
| Applying an analytical process to longitudinal narrative interviews with couples living and dying with Lewy body dementia | Clinician or self-report | clinician(s)  self-report |
| Association between amyloid-beta deposition and cortical thickness in dementia with Lewy bodies | Referring clinician | clinician(s) |
| Association of premorbid personality with behavioral and psychological symptoms in dementia with Lewy bodies: comparison with Alzheimer’s disease patients | Clinician | clinician(s) |
| Attitudes toward own aging and cognition among individuals living with and without dementia: findings from the IDEAL programme and the PROTECT study | Participants’ medical records | Clinician(s) |
| Autonomic symptoms are predictive of dementia with Lewy bodies | Clinician | clinician(s) |
| Behavioral and psychological effects of coronavirus disease-19 quarantine in patients with dementia | self-report | self-report |
| Bilateral nucleus basalis of Meynert deep brain stimulation for dementia with Lewy bodies: a randomised clinical trial | referring clinician | clinician(s) |
| Capgras syndrome in dementia with Lewy bodies | Clinician-judged on the basis of the clinical interview, physical examination and cognitive evaluation in accordance with the 2005 revised DLB consensus criteria for the clinical diagnosis of probable DLB. | clinician(s) |
| Care burden and mental ill health in spouses of people with Parkinson disease dementia and Lewy body dementia | If recruited through route 1 (memory and movement disorder clinics), the clinician made the diagnosis. If recruited through route 2, spouses self-reported the diagnosis of the care recipient. | clinician(s) Self-report |
| Caregiver burden in family carers of people with dementia with Lewy bodies and Alzheimer’s disease | For DemVest, two study clinicians independently applied the diagnostic criteria. In cases of disagreement, and in patients fulfilling more than one set of operationalised diagnostic criteria, the final ascertainment was made based on consensus. For The Norwegian Dementia Register, diagnoses were made by clinicians and discussed in interdisciplinary consensus meetings. | Clinician(s) |
| Caregiver burden, sleep quality, depression, and anxiety in dementia caregivers: a comparison of frontotemporal lobar degeneration, dementia with Lewy bodies, and Alzheimer’s disease | Clinician | clinician(s) |
| Caregiver self-efficacy and associated factors among caregivers of patients with dementia with Lewy bodies and caregivers of patients with Alzheimer’s disease | Clinician | clinician(s) |
| Caregiver-reported barriers to quality end-of-life care in dementia with Lewy bodies: a qualitative analysis | Self-report | self-report |
| Carers to people with Lewy body dementia and Alzheimer's disease: experiences and coping strategies | Clinician | clinician(s) |
| Caring for people with dementia with Lewy bodies and Parkinson’s dementia in UK care homes – A mixed methods study | Not applicable (study population was care home staff) | Not applicable |
| Cause of death and end-of-life experiences in individuals with dementia with Lewy bodies | Self-report | self-report |
| Characteristics of eating and swallowing problems in patients who have dementia with Lewy bodies | Clinician | clinician(s) |
| Characterization of symptoms and determinants of disease burden in dementia with Lewy bodies: DEvELOP design and baseline results | Multidisciplinary team | clinician(s) |
| Clinical care and unmet needs of individuals with dementia with Lewy bodies and caregivers: an interview study | clinician | clinician(s) |
| Clinical features of delusional jealousy in elderly patients with dementia | Senior neuropsychiatrists | Clinician(s) |
| Clinical findings, functional abilities and caregiver distress in the early stage of dementia with Lewy bodies (DLB) and Alzheimer’s disease (AD) | Clinician | clinician(s) |
| Cognition, hallucination severity and hallucination-specific insight in neurodegenerative disorders and eye disease | Diagnosis made according to diagnostic criteria | clinician(s) |
| Cognitive effects of quetiapine in a patient with dementia with Lewy bodies | clinician | clinician(s) |
| Cohort study on somatoform disorders in Parkinson disease and dementia with Lewy bodies | Clinician | clinician(s) |
| Comparison of caregiver burden between dementia with Lewy bodies and Alzheimer’s disease | Clinician | clinician(s) |
| Comparison of QOL between patients with different degenerative dementias, focusing especially on positive and negative affect | Clinician | clinician(s) |
| Comparison of the caregiving experience of grief, burden, and quality of life in dementia with Lewy bodies, Alzheimer’s disease, and Parkinson’s disease dementia | Self-report | self-report |
| Contrasts between patients with Lewy body dementia syndromes and APOE- ε3/ε3 patients with late-onset Alzheimer disease dementia | Clinician | clinician(s) |
| Conversations in dementia with Lewy bodies: resources and barriers in communication | clinician | clinician(s) |
| Correlates of neuropsychiatric and motor tests with language assessment in patients with Lewy body dementia | Clinician | clinician(s) |
| Costs of dementia with Lewy bodies: a Chinese multicenter cross-sectional study | Final diagnosis was confirmed by two experienced neurologists following a case review according to protocol. | Clinician(s) |
| COVID-19: association between increase of behavioral and psychological symptoms of dementia during lockdown and caregivers’ poor mental health | Caregiver reported care recipient’s diagnosis | Self-report |
| Delusions in patients with dementia with Lewy bodies and the associated factors | Retrieved from a health system’s dementia database- Dementia and subtypes of dementia were diagnosed by a consensus meeting composed of three neurologists, one geriatric psychiatrist, and one neuropsychologist. | Clinician(s) |
| Dementia patients caregivers quality of life: the PIXEL study | Clinician | clinician(s) |
| Dementia subtype and living well: results from the Improving the experience of Dementia and Enhancing Active Life (IDEAL) study. | Dementia diagnoses were made by different clinicians across the country. The diagnoses were obtained from medical records of the participants and classified in seven groups: AD, VaD, mixed AD and VaD, FTD, PDD, LBD and other/ unspecified. For those who selected other or an unspecified diagnosis in the interviews, open-ended text descriptions provided by the interviewer were reviewed by two clinicians and re-categorised into the six empirical groups where possible. | Clinician(s) |
| Depression in dementia with Lewy bodies: a comparison with Alzheimer’s disease | Dementia and subtype of dementia were made by a consensus meeting composed of two neurologists, one geriatric psychiatrist, and one neuropsychologist who were all part of the research team. All patients received cerebral CT or MRI to rule out other possible causes of dementia. | Clinician(s) |
| Development and validation of the Lewy body disease caregiver activities scale | Family caregivers of persons with LBD were recruited from a cognitive disorders centre. Potential participants were informed of the study during their visit to the centre, or through a letter signed by the Medical Director along with a study information sheet mailed to their home. | Clinician(s) |
| Differences in the experience of caregiving between spouse and adult child caregivers in dementia with Lewy bodies | self-report | self-report |
| Differential associations of clinical features with cerebrospinal fluid biomarkers in dementia with Lewy bodies and Alzheimer’s disease | Clinicians | clinician(s) |
| Difficulties and associated coping methods regarding visual hallucinations caused by dementia with Lewy bodies | Clinician | clinician(s) |
| Disease progression in dementia with Lewy bodies: a longitudinal study on clinical symptoms, quality of life and functional impairment. | Multidisciplinary team | clinician(s) |
| Donepezil for dementia with Lewy bodies: a randomized, placebo-controlled trial | Diagnosis of each patient was validated after discussion by the central committee. | clinician(s) |
| Donepezil for treatment of dementia with Lewy bodies: a case series of nine patients | Clinician | clinician(s) |
| Effectiveness of ramelteon for treatment of visual hallucinations in dementia with Lewy bodies a report of 4 cases | Clinician | clinician(s) |
| Efficacy of adjunctive therapy with zonisamide versus increased dose of levodopa for motor symptoms in patients with dementia with Lewy bodies: the randomized, controlled, non-inferiority DUEL study | clinicians | clinician(s) |
| Efficacy, safety, and tolerability of armodafinil therapy for hypersomnia associated with dementia with Lewy bodies: a pilot study | Clinicians | clinician(s) |
| End-of-life experiences in dementia with Lewy bodies: qualitative interviews with former caregivers | Self-report | self-report |
| Examining carer stress in dementia: the role of subtype diagnosis and neuropsychiatric symptoms | Case notes were examined by two Old Age Psychiatrists in the research team to confirm that diagnoses met appropriate internationally recognised diagnostic criteria before recruitment of their carers into the study. Where there was disagreement over patients’ diagnoses, case notes were reviewed to reach a consensus agreement on diagnosis. | clinician(s) |
| Exploring the impact of caring for an individual with neurogenic orthostatic hypotension: a qualitative study | The agency identified potential participants through clinician referral, patient associations, patient research databases and social media. | Self-report Clinician(s) |
| Factors associated with burden among male caregivers for people with dementia | Clinicians | clinician(s) |
| Factors of dementia caregiver burden differentially contribute to desire to institutionalize | Self-report | self-report |
| Frequency of suicidal ideation and associated clinical features in Lewy body dementia | A fellowship-trained movement disorders specialist. | Clinician(s) |
| Goal setting for cognitive rehabilitation in mild to moderate Parkinson’s disease dementia and dementia with Lewy bodies | Clinician | clinician(s) |
| Goal-orientated cognitive rehabilitation for dementias associated with Parkinson's disease- a pilot randomised controlled trial | Clinicians | clinician(s) |
| Health related quality of life and cognitive decline in older populations: preliminary results from NeuroDemeNPsia Study | Neurologists | clinician(s) |
| Health related quality of life in individuals with cognitive decline and discrepancies between patients and their proxies | Neurologists | clinician(s) |
| Impact of behavioral and psychological symptoms on caregiver burden in patients with dementia with Lewy bodies | clinicians | clinician(s) |
| Impact of the covid-19 pandemic and lockdown on anxiety, depression and nursing burden of caregivers in Alzheimer’s disease, dementia with Lewy bodies and mild cognitive impairment in China: a 1-year follow-up study | Clinical diagnoses were made by at least two expert neurologists in agreement. Blood tests, neurological examination, neuroimaging, and positron emission computerised tomography were performed to confirm the diagnosis if necessary. | clinician(s) |
| Improvement in delusions and hallucinations in patients with dementia with Lewy bodies upon administration of yokukansan, a traditional Japanese medicine | Clinician | clinician(s) |
| Improving the diagnosis and management of Lewy body dementia: the DIAMOND-Lewy research programme including pilot cluster RCT | Clinician-judged | clinician(s) |
| Informal caregiver experiences at the end-of-life of individuals living with dementia with Lewy bodies: an interview study | Self-report | self-report |
| Key components of post-diagnostic support for people with dementia and their carers: a qualitative study | Recruited via gatekeepers from the six services identified during phase 1 of the study | Clinician |
| Learning to PERSEVERE: a pilot study of peer mentor support and caregiver education in Lewy body dementia | Both self-reported and clinician (clinic-based referrals)- Most mentees and mentors were recruited through the Parkinson’s Foundation and Lewy Body Dementia Association, which disseminated a recruitment survey link via e-mail and social media. Recruitment flyers were also distributed to regional PD and LBD support groups, shared by neurologists with eligible caregivers, and provided to prior Parkinson’s disease mentoring study participants who met the mentor eligibility criteria. | Self-report Clinician(s) |
| Lewy body dementia: caregiver burden and unmet needs | self-report | self-report |
| Lewy body dementia: the caregiver experience of clinical care | Self-report | self-report |
| Living with dementia with Lewy bodies: an interpretative phenomenological analysis | clinician | clinician(s) |
| Long-term donepezil use for dementia with Lewy bodies: results from an open-label extension of Phase III trial | clinician | clinician(s) |
| Long-term safety and efficacy of donepezil in patients with dementia with Lewy bodies: results from a 52-week, open-label, multicenter extension study | Clinician | clinician(s) |
| Lumateperone for treatment of psychotic symptoms in Lewy body: a case report | Clinician | clinician(s) |
| Measuring disability in patients with neurodegenerative disease using the ‘Yesterday Interview’ | clinician | clinician(s) |
| Memantine for patients with Parkinson’s disease dementia or dementia with Lewy bodies: a randomised, double-blind, placebo-controlled trial | clinician | clinician(s) |
| Multidimensional care burden in Parkinson-related dementia | Self-reported by life partners in the postal questionnaire survey.  For the INVEST trial, diagnosis was determined by the referring clinician and verified on screening. | Clinician(s) Self-report |
| Music playlists for people with dementia: trialling a guide for caregivers | Self-report | self-report |
| My Friend Lewy | Not applicable | N/A |
| Needs and concerns of Lewy body disease  family caregivers: a qualitative study | Recruited from a cognitive disorders centre. Potential participants were informed of the study during their visit, or through a letter signed by the Medical Director along with a mailed study information sheet. | Clinician(s) |
| Neuropsychiatric feature profiles of patients with Lewy body dementia | Clinician. Cerebrospinal fluid biomarkers measured by way of enzyme-linked immunosorbent assays were employed for diagnostic confirmation when cognitive decline was slower than expected or atypical behavioural features were presented. | Clinician(s) |
| Neuropsychological study of amyotrophic lateral sclerosis and parkinsonism-dementia complex in Kii peninsula, Japan | Clinician | clinician(s) |
| Open label trial to evaluate the efficacy and safety of Yokukansan, a traditional Asian medicine, in dementia with Lewy bodies | Clinicians | clinician(s) |
| Pacemaker implants and their influence on the daily life of patients with dementia with Lewy bodies: a qualitative case study | clinician | clinician(s) |
| Pain in extrapyramidal neurodegenerative diseases | Clinician | clinician(s) |
| Pain in patients with different dementia subtypes, mild cognitive impairment, and subjective cognitive impairment | The diagnosis of dementia was determined by consensus within a multidisciplinary team using established diagnostic criteria. The clinical diagnosis process included computed tomography or magnetic resonance imaging as standard procedures. | clinician(s) |
| Parkinson’s/ Lewy body dementia: a carer’s perspective | Not applicable | Not applicable |
| Parkinson’s-adapted cognitive stimulation therapy: a pilot randomized controlled clinical trial | Clinician | clinician(s) |
| Parkinson’s-adapted cognitive stimulation therapy: feasibility and acceptability in Lewy body spectrum disorders | Clinician | clinician(s) |
| Patient affect and caregiver burden in dementia | Clinician | clinician(s) |
| Patients with dementia with Lewy bodies have more impaired quality of life than patients with Alzheimer disease | study clinicians | clinician(s) |
| Patients with Lewy body dementia use more resources than those with Alzheimer’s disease | clinician | clinician(s) |
| Patterns of carer distress over time in mild dementia | clinician | clinician(s) |
| Perceived benefits of using nonpharmacological interventions in older adults with Alzheimer’s disease or dementia with Lewy bodies | clinician | clinician(s) |
| Personality traits distinguishing dementia with Lewy bodies from Alzheimer disease | Neuropathologically defined. | Neuropathologically defined. |
| Practical treatment of Lewy body disease in the clinic: patient and physician perspectives | Not applicable | Not applicable |
| Preclinical polymodal hallucinations for 13 years before dementia with Lewy bodies | clinician | clinician(s) |
| Pre-loss grief in caregivers of older adults with dementia with Lewy bodies | Self-report | self-report |
| Presence phenomena in parkinsonian disorders: phenomenology and neuropsychological correlates | clinician | clinician(s) |
| Problematising carer identification: a narrative study with older partner's providing end-of-life care | Unclear which recruitment routes were used for participants of interest, as multiple methods were employed. | unclear |
| Problems faced by people living at home with dementia and incontinence: causes, consequences and potential solutions | self-report | self-report |
| Prodromal dementia with Lewy bodies: clinical characterization and predictors of progression | Multidisciplinary team | Clinician(s) |
| Profiling conversation in Parkinson’s disease with cognitive impairment | Clinician | Clinician(s) |
| Quality of life and the effect of memantine in dementia with Lewy bodies and Parkinson’s disease dementia | Clinician | clinician(s) |
| Questionnaire survey of satisfaction with medication for five symptom domains of dementia with Lewy bodies among patients, their caregivers, and their attending physicians | Clinician | clinician(s) |
| Raising awareness and mutual support: using the internet | Not applicable | Not applicable |
| Research priorities of caregivers and individuals with dementia with Lewy bodies: An interview study | Clinician | clinician(s) |
| Research priorities of individuals and caregivers with Lewy body dementia a web-based survey | self-report | self-report |
| Self-efficacy and social support for psychological well-being of family caregivers of care recipients with dementia with Lewy bodies, Parkinson’s disease dementia, or Alzheimer’s disease | self-report | self-report |
| Stress and burden among caregivers of patients with Lewy body dementia | self-report | self-report |
| Study of prevalence of neuropsychiatric symptoms in elderly dementia patients | Clinician | clinician(s) |
| Subjective experience of time in dementia with Lewy bodies during COVID-19 lockdown | Clinician | clinician(s) |
| Support and information needs following a diagnosis of dementia with Lewy bodies | Self-report | self-report |
| Tailoring and evaluating the web-based ‘Partner in Balance’ intervention for family caregivers of persons with young-onset dementia | Self-report | self-report |
| Tau in dementia with Lewy bodies | Clinician | clinician(s) |
| The association between specific neuropsychiatric disturbances in people with Alzheimer's disease and dementia with Lewy bodies and carer distress | A diagnosis of dementia was made according to the Diagnostic and Statistical Manual of Mental Disorders, Fourth Edition (DSM-IV), by an experienced clinician and licensed specialist in geriatric medicine or psychiatry. DLB was diagnosed according to the revised Consensus Guidelines for the Clinical Diagnosis of Probable and Possible DLB, while AD was diagnosed according to the National Institute of Neurological and Communicative Disorders and Stroke-Alzheimer's Disease and Related Disorders Association. A pathological diagnosis was available for 56 patients in the DemVest cohort, with a diagnostic accuracy of over 80%. | Clinician(s) Pathological diagnosis (for some) |
| The effects of behavioral and psychological symptoms on caregiver burden in frontotemporal dementia, Lewy body dementia, and Alzheimer's disease: clinical experience in China | Clinician | clinician(s) |
| The feasibility and acceptability of a psychosocial intervention to support people with dementia with Lewy bodies and family care partners | Clinician | clinician(s) |
| The frequency and correlates of anxiety in patients with first-time diagnosed mild dementia | Two study clinicians independently applied the diagnostic criteria. In cases of disagreement, and in patients fulfilling more than one set of operationalised diagnostic criteria, the final ascertainment was made based on consensus. | Clinician(s) Pathological diagnosis (for some) |
| The human need for equilibrium: qualitative study on the ingenuity, technical competency, and changing strategies of people with dementia seeking health information | Self-report | self-report |
| The impact of covid-19 quarantine on patients with dementia and family caregivers: a nation-wide survey | clinician | clinician(s) |
| The importance of educating the Lewy body dementia community on risks and benefits of lumbar punctures in LBD biomarker research | Online survey therefore self-report | Self-report |
| The insula, a grey matter of tastes: a volumetric MRI study in dementia with Lewy bodies | Clinician | clinician(s) |
| The role of sexual disinhibition to predict caregiver burden and desire to institutionalize among family dementia caregivers | Participants self-reported as dementia caregivers and provided the diagnosis of the person they cared for. | self-report |
| The unique experience of spouses in early-onset dementia | Clinician | clinician(s) |
| The use and costs of paid and unpaid care for people with dementia: Longitudinal findings from the IDEAL cohort | Unclear which recruitment routes were used for participants of interest, as multiple methods were employed. | unclear |
| Time until nursing home admission in people with mild dementia: comparison of dementia with Lewy bodies and Alzheimer’s dementia | Clinician  Neuropathological confirmation | clinician(s) Neuropathological confirmation |
| Trajectories and determinants of quality of life in dementia with Lewy bodies and Alzheimer’s disease | Clinician | clinician(s) |
| Treatment needs of dementia with Lewy bodies according to patients, caregivers, and physicians: a cross-sectional, observational questionnaire-based study in Japan | Referring clinician | clinician(s) |
| Understanding the nature and impact of cognitive fluctuations and sleep disturbances in dementia with Lewy bodies: a qualitative caregiver study | Clinician | Clinician |
| Updates on somatoform disorders (SFMD) in Parkinson's disease and dementia with Lewy bodies and discussion of phenomenology | clinician | clinician(s) |
| Using care navigation to address caregiver burden in dementia: a qualitative case study analysis | clinician | clinician(s) |
| Video research visits for atypical parkinsonian syndromes among Fox Trial Finder participants | Self-report | self-report |
| Visual hallucinations in eye disease and Lewy body disease | clinician | clinician(s) |
| Development of assessment toolkits for improving the diagnosis of the Lewy body dementias: feasibility study within the DIAMOND Lewy study | clinician | clinician(s) |
| Introduction of a management toolkit for Lewy body dementia: a pilot cluster-randomized trial | clinician | clinician(s) |

**Appendix 8:** Study designs and methods

| **Research design and title of the source** | **Overview of methods capturing perspective data** | **Standardised measures capturing perspectives** | **Information on qualitative methods** |
| --- | --- | --- | --- |
| **1. Sources using a qualitative approach (n=23):** | | | |
| **1A. Cross-sectional design (n=19)** | | | |
| Research priorities of caregivers and individuals with dementia with Lewy bodies: an interview study | Telephone interviews using a bespoke, semi-structured questionnaire. | None | - Method: Interview - Format: Telephone - Design: semi-structured - Relationship between researcher and participant: no prior relationship. - Method of qualitative analysis: content analysis - Duration of time needed: Average interview duration was 28:11 minutes for individuals with DLB and 37:10 minutes for caregivers. - Managing dyadic interviews: preferred approach was to interview separately (if both participated). If interviewed together, responses coded separately. |
| Informal caregiver experiences at the end-of-life of individuals living with dementia with Lewy bodies: an interview study | Semi-structured telephone interviews (bespoke guide). | None | - Method: Interview - Format: telephone interview - Design: semi-structured - Relationship between researcher and participant: no prior relationship. - Method of qualitative analysis: qualitative descriptive approach and content analysis - Duration of time: Interviews averaged 31 minutes. - Managing dyadic interviews: Not applicable. |
| Living with dementia with Lewy bodies: an interpretative phenomenological analysis | Face to face in-depth interviews using a bespoke guide. Afterwards, participants completed a QoL-AD questionnaire to assess perceived quality of life. | QoL-AD questionnaire | - Method: Interview - Format: face-to-face in the participant’s home - Design: semi-structured - Relationship between researcher and participant: no prior relationship with the participants. - Method of qualitative analysis: interpretative phenomenological analysis. - Duration of time: Between 60 and 134 minutes. - Managing dyadic interviews: Participants were encouraged to be interviewed alone. |
| End-of-life experiences in dementia with Lewy bodies: qualitative interviews with former caregivers | Semi-structured telephone interviews (bespoke guide) | None | - Method: Interviews - Format: Telephone interview - Design: Semi-structured - Relationship between researcher and participant: no prior relationship - Method of qualitative analysis: content analysis - Duration of time: Average was 31 minutes - Managing dyadic interviews: Not applicable |
| Caregiver-reported barriers to quality end-of-life care in dementia with Lewy bodies: a qualitative analysis | Semi-structured telephone interviews (bespoke guide) | None | - Method: telephone interviews conducted by MJA (PI) who is a clinician specialising in DLB. - Format: telephone - Design: semi-structured - Relationship between researcher and participant: No prior relationship with participants. - Method of qualitative analysis: content analysis - Duration of time (minutes): Average was 31 minutes. - How were dyadic interviews managed: not applicable |
| Clinical care and unmet needs of individuals with dementia with Lewy bodies and caregivers: an interview study | Telephone interviews (bespoke guide) | None | - Method: Interviews - Format: Telephone - Design: semi-structured - Relationship between researcher and participant: No prior relationship - Method of qualitative analysis: content analysis - Duration of time (minutes): Patient average interview duration: 28:11 minutes. Interview duration of caregivers (mean, minutes): 37:10. Note- This is the total interview duration, including reflections on both clinical care and research priorities which was reported in a separate paper. - How were dyadic interviews managed: preferred approach was to interview separately (if both participated). If interviewed together, responses coded separately. |
| ‘It's just incredible the difference it has made': family carers' experiences of a specialist Lewy body dementia Admiral Nurse service | Semi-structured interviews (bespoke guide) | None | - Method: interview - Format: either by telephone or over a video conferencing platform. - Design: semi-structured - Relationship between researcher and participant: Unclear - Method of qualitative analysis: thematic analysis - Duration of time (minutes): not reported - How were dyadic interviews managed: Not applicable. |
| The unique experience of spouses in early-onset dementia | Semi-structured, face-to-face interviews based on the interviews developed by Clare and colleagues [1]. | None | - Method: interview - Format: in-person (either in the cognition clinic or at home at the participant’s discretion and convenience). - Design: semi-structured - Relationship between researcher and participant: not reported - Method of qualitative analysis: thematic content analysis - Duration of time (minutes): 90 minutes on average (for the whole sample) - How were dyadic interviews managed: not applicable |
| Exploring the impact of caring for an individual with neurogenic orthostatic hypotension: a qualitative study | Semi-structured telephone interviews (bespoke guide) | None | - Method: interviews - Format: telephone - Design: semi-structured - Relationship between researcher and participant: not reported - Method of qualitative analysis: thematic analysis - Duration of time (minutes): 40-70 minutes. - How were dyadic interviews managed: not applicable |
| Needs and concerns of Lewy body disease family caregivers: a qualitative study | Semi-structured telephone interviews. Pre-existing guide used which was based on the work of Bakas and colleagues [2]. | None | - Method: interview - Format: Telephone - Design: Semi-structured - Relationship between researcher and participant: no prior relationship - Method of qualitative analysis: content analysis - Duration of time (minutes): ranged from 30 to 63 minutes, averaging 47.5 minutes. - How were dyadic interviews managed: not applicable |
| Understanding the nature and impact of cognitive fluctuations and sleep disturbances in dementia with Lewy bodies: a qualitative caregiver study | Semi-structured telephone interviews (bespoke guide) | None | - Method: interview - Format: telephone - Design: semi-structured - Relationship between researcher and participant: No prior relationship - Method of qualitative analysis: thematic analysis - Duration of time (minutes): mean duration = 19.47 mins - How were dyadic interviews managed: not applicable |
| A qualitative study of female caregiving spouses’ experiences of intimate relationships as cognition declines in Parkinson’s disease | Semi-structured, face-to-face interviews (bespoke guide informed by the Personal Assessment of Intimacy in Relationships). Participants were also asked to rate their current and premorbid relationship satisfaction quantitatively on a horizontal visual analogue scale. | None | - Method: interview - Format: face-to-face - Design: semi-structured - Relationship between researcher and participant: Interviewees were known to the interviewer - Method of qualitative analysis: inductive thematic analysis - Duration of time (minutes): 35-97 minutes. - How were dyadic interviews managed: not applicable |
| Problems faced by people living at home with dementia and incontinence: causes, consequences and potential solutions | Semi structured, face-to-face interviews. Bespoke guide. | None | - Method: Interview - Format: Face-to-face in participants’ homes or workplaces. - Design: Semi-structured - Relationship between researcher and participant: not reported - Method of qualitative analysis: Framework analysis - Duration of time (minutes): not reported - How were dyadic interviews managed: not applicable |
| Perceived benefits of using nonpharmacological interventions in older adults with Alzheimer’s disease or dementia with Lewy bodies | Three focus groups (using bespoke guide) conducted with family caregivers of older adults with dementia following completion of the three different 12-week interventions. | None | - Method: focus group - Format: face-to-face - Design: A generic focus group questionnaire - Relationship between researcher and participant: not reported - Method of qualitative analysis: constant comparative analysis - Duration of time (minutes): approximately 1.5 hours. - How were dyadic interviews managed: Not applicable |
| Difficulties and associated coping methods regarding visual hallucinations caused by dementia with Lewy bodies | Semi-structured interviews (bespoke guide). Each participant underwent one interview in the presence of a family member. | None | - Method: interview - Format: face-to-face in the participant’s preferred location. - Design: semi-structured - Relationship between researcher and participant: Not reported - Method of qualitative analysis: content analysis - Duration of time (minutes): approximately 60 minutes. - How were dyadic interviews managed: When necessary, family members were asked to supplement the participants’ comments. The participants and family members were also given the opportunity to be interviewed separately. |
| Carers to people with Lewy body dementia and Alzheimer's disease: experiences and coping strategies | Two DLB focus groups (bespoke guide) | None | - Method: focus group interviews - Format: face-to-face - Design: semi-structured - Relationship between researcher and participant: not reported - Method of qualitative analysis: Systematic Text Condensation - Duration of time (minutes): 60-90 minutes - How were dyadic interviews managed: not applicable |
| Key components of post-diagnostic support for people with dementia and their carers: a qualitative study | Interviews explored perceptions of current service use and views on post-diagnostic support (bespoke guide used). Forty-eight sessions of observation were also completed and recorded in fieldnotes. Observation included direct service provision. | None | - Method: In phase 2, interviews were conducted to capture the perspectives of PLWD and carers. - Format: either face-to-face in the home or over the phone. - Design: semi-structured - Relationship between researcher and participant: participants not previously known to the researchers - Duration of time: average duration was 37 minutes. - Method: Forty-eight sessions of observation were completed and recorded in fieldnotes. Observation included direct service provision and relevant meetings. - Method of qualitative analysis: inductive, thematic approach to analysis. |
| Profiling conversation in Parkinson’s disease with cognitive impairment | Conversation analysis tool- Interview based on the CAPPCI. | CAPPCI | - Method: Interview - Format: Face-to-face - Design: Based on the CAPPCI. - Relationship between researcher and participant: not reported - Method of qualitative analysis: conversational analysis using the CAPPCI - Duration of time: 45-60 minutes - Managing dyadic interviews: not applicable |
| The human need for equilibrium: qualitative study on the ingenuity, technical competency, and changing strategies of people with dementia seeking health information | Contextual inquiry sessions comprising 2 parts: (1) semi-structured interviews; and (2) an observation session. | None | - Method: interview - Format: via Zoom - Design: semi-structured - Relationship between researcher and participant: not reported - Method of qualitative analysis: A constructivist grounded theory approach - Duration of time (minutes): 60 minutes - How were dyadic interviews managed: not applicable |
| **1.B. Longitudinal designs (n=2)** | | | |
| Applying an analytical process to longitudinal narrative interviews with couples living and dying with Lewy body dementia | The couple was interviewed three times over a six-month period using in-depth, open-ended narrative interviews. A bespoke interview guide was utilised. | None | - Method: (Repeated) interviews offered flexibly over three months. - Format: face-to-face (reported in thesis) - Design: in-depth, open-ended narrative interviews which were conversational in nature. - Method of qualitative analysis: Narrative analysis - Duration of time needed: interview 1= 64 mins, interview 2= 1 hr 42 mins (60 mins with carer only), interview 3= 67 mins - Managing dyadic interviews: The interviews were offered to take place as dyads or separately. Participants were allowed to stimulate ideas in each other and probe each other in the context of their narratives. If the person LBD deteriorated it was acknowledged that interviews may be with a family carer only. |
| Problematising carer identification: a narrative study with older partner's providing end-of-life care | Two LBD care partners took part in repeated semi-structured, in-person, narrative interviews held approximately a month apart (using a bespoke guide). | None | - Method: Both LBD care partners took part in two interviews held approximately a month apart - Format: in-person interviews - Design: semi-structured - Relationship between researcher and participant: not reported - Method of qualitative analysis: narrative analysis - Duration of time (minutes): No LBD specific data. For the whole group, interviews averaged one-and-a-half hours but ranged from 30 minutes to 6 hours - How were dyadic: unclear if this applied to LBD |
| **1C. Longitudinal case study designs (n=1)** | | | |
| Pacemaker implants and their influence on the daily life of patients with dementia with Lewy bodies: a qualitative case study | Patient-care partner dyads repeatedly interviewed within 1 year following implant of a dual-chamber rate-adaptive pacemaker to manage sick sinus syndrome. | None | - Method: (repeated) dyadic interviews - Format: repeated face-to-face. Data was collected at the Memory clinic where the men were patients. - Design: Semi-structured - Relationship between researcher and participant: during interview 1, interviewees were known to interviewer but not in the second interview. - Method of qualitative analysis: Content analysis - Duration of time: Initial interviews: 11 and 17 minutes. Follow-up interviews: 31 and 45 minutes. - Managing dyadic interviews: Not specifically discussed. Coded separately |
| **1D. Cross-sectional case study designs (n=1)** | | | |
| Using care navigation to address caregiver burden in dementia: a qualitative case study analysis | Interviews, focus groups, observations and qualitative case study analysis. | None | 1. Method: interviews   - Format: not reported - Design: semi-structured - Relationship between researcher and participant: Not reported - Method of qualitative analysis: thematic analysis - Duration of time: not reported - Managing dyadic interviews: Not applicable   2. Method: Focus groups   - Format: not reported - Design: semi-structured - Relationship between researcher and participant: Not reported - Method of qualitative analysis: thematic analysis as above. - Duration of time: 60 minutes - Managing dyadic interviews: Not applicable   3. Method: Observations (n=20) at all-staff meetings and clinical support team debriefings   - Method of qualitative analysis: First author reviewed observation notes to assess and triangulate these data in relationship to interview and focus group findings.   4. Method: qualitative case study analysis |
| **2. Sources using a quantitative approach (n= 93)** | | | |
| **2A. Cross-sectional (n=63)** | | | |
| COVID-19: Association Between Increase of Behavioral and Psychological Symptoms of Dementia During Lockdown and Caregivers’ Poor Mental Health | Anonymous online survey. A hard copy of the survey, along with a stamped envelope, was provided to people without web access. The survey comprised both standardised and non-standardised measures, however no DLB/LBD specific results were reported for the standardised measures. A novel, investigator-generated measure provided Lewy body-specific data. The question asked, “Since lockdown, has your relative presented psychological or behavioural manifestations?” | No DLB scores for scales | Not applicable |
| Lewy body dementia: caregiver burden and unmet needs | An internet survey. The survey comprised standardised measures (ZBI reported) as well as investigator-generated Likert scales capturing descriptive assessments of the LBD caregiver experience. | ZBI (11-item) | Not applicable |
| Comparison of the Caregiving Experience of Grief, Burden, and Quality of Life in Dementia with Lewy Bodies, Alzheimer’s Disease, and Parkinson’s Disease Dementia | A 230-question web-survey including sociodemographics, disease severity, neuropsychiatric symptoms, and standardised and non-standardised measures. The respondent’s confidence in their ability to provide care to the PLWD was measured using 4 investigator-generated questions that were adapted from the Dementia Care Confidence scale. Caregiver mastery and self-efficacy were measured using 12 investigator-generated questions. Open-text fields were included. | - CGI - ZBI (12-item) - PHQ-2 - QoL-AD - MOS-SS - PCI - C-PWBS | Not applicable |
| Stress and burden among caregivers of patients with Lewy body dementia | Used data from an internet survey conducted by the LBDA. The survey included the ZBI and investigator-generated measures of social isolation, difficulty finding and evaluation of a physician/clinician, and overall evaluation of help received. | ZBI (12-item) | Not applicable |
| Pre-loss grief in caregivers of older adults with dementia with Lewy bodies | Online survey consisting of questions on social and demographic characteristics, the Quick Dementia Rating Scale, standardised measures, and investigator-generated measures to characterise pre-loss grief. | - PG-12 - Brief ZBI (12-item) - PCI - C-PWBS - PHQ-2 | Not applicable |
| Differences in the experience of caregiving between spouse and adult child caregivers in dementia with Lewy bodies | A retrospective online survey that included standardised measures and investigator-generated measures of caregiver social support and the caregiver's social network. | - ZBI-12 item - MM-CGI-SF - PHQ2 - C-PWBS - QoL-AD | Not applicable |
| Cause of death and end-of-life experiences in individuals with dementia with Lewy bodies | Bespoke, 20 question online survey. The survey included three questions about the respondent's background and 17 about the end-of-life experiences of the person with DLB. The survey consisted of multiple-choice questions and some free-text response options. | - None | Not applicable |
| The role of sexual disinhibition to predict caregiver burden and desire to institutionalize among family dementia caregivers | Participants completed measures online in the format of a self-administered questionnaire. No DLB or LBD-specific data was reported for standardised measures. A novel measure of sexual disinhibition was employed. Participants also reported any behaviour they found “socially inappropriate and/or embarrassing” in a free-text response format. | None | Not applicable |
| Pain in patients with different dementia subtypes, mild cognitive impairment, and subjective cognitive impairment | Included patients recruited from three outpatient memory clinics. In two clinics, pain was assessed using the BPI. In the third clinic, pain was assessed in two ways. The first assessment took place during a general physical examination as part of the clinical diagnostic procedure, in which all patients were questioned about their physical complaints, including pain complaints. Pain intensity was assessed using an 11-point numerical rating scale, which was identical to that used in the other two clinics. | - BPI | Not applicable |
| The insula, a grey matter of tastes: a volumetric MRI study in dementia with Lewy bodies | During a clinical evaluation, a bespoke questionnaire assessed potential changes in personal tastes. A section for non-food domains was included since the investigators were particularly interested in exploring the potential role of the insula in personal tastes in general, beyond food preferences. The questionnaire contained Likert-scale questions, and patients had the opportunity to exemplify their changes in taste. | None | Not applicable |
| Dementia patients caregivers quality of life: the PIXEL study | A scale measuring caregivers' quality of life, developed using data from previous PIXEL studies, was used. The caregiver scale was related to the socio-demographic data of both patients and their main caregivers, the ADRQL scale which measured the quality of life of the PLDW, and the patient's medical and therapeutic data. | - ADRQL scale | Not applicable |
| Questionnaire survey of satisfaction with medication for five symptom domains of dementia with Lewy bodies among patients, their caregivers, and their attending physicians | Patients and caregivers who participated in this study underwent several screening tests, including the ZBI. They then each completed a bespoke questionnaire. Surveys were individually prepared for patients, caregivers and physicians/clinicians. The questionnaire choices for patients and caregivers included seven items: ‘very satisfied’, ‘satisfied’, ‘neither’, ‘unsatisfied’, ‘very unsatisfied’, ‘no medication’, and ‘unknown’ to evaluate their satisfaction with the effectiveness of their current medication for five symptom domains of cognitive impairment, parkinsonism, psychiatric symptoms, sleep-related disorders, and autonomic dysfunction. | - J-ZBI_8 | Not applicable |
| Lewy body dementia: the caregiver experience of clinical care | 15-minute, bespoke, web-based survey of 962 self-reported LBD caregivers over a 6-month period to address issues of challenges, burdens and frustrations facing LBD caregivers in obtaining a diagnoses and care for the patient. The survey is not available. There was no reference to scales or free-text response options. | None | Not applicable |
| Research priorities of individuals and caregivers with Lewy body dementia a web-based survey | A bespoke, web-based survey consisting of 15–20 questions was used to query research priorities through forced ranking and to explore the burden of LBD symptoms. A free-text response space was included to allow participants to provide additional comments on the theme of LBD research. | None | Not applicable |
| Treatment needs of dementia with Lewy bodies according to patients, caregivers, and physicians: a cross-sectional, observational questionnaire-based study in Japan | Patients and caregivers who participated underwent several screening tests using standardised measures. A bespoke questionnaire containing open- and closed-ended question was then administered to understand the treatment needs of patients with DLB and their caregivers, as well as the extent to which the attending physicians/clinicians understand these treatment needs. Patient survey had 40 questions, while the caregiver survey consisted of 78 questions. | - J-ZBI -8 - SF-8 | Not applicable |
| The impact of covid-19 quarantine on patients with dementia and family caregivers: a nation-wide survey | Family caregivers of people with dementia were interviewed by phone using a bespoke survey that consisted of closed-ended, multiple choice questions. The survey examined the impact of COVID-19 quarantine on patients and family care partners. | None | Not applicable |
| The importance of educating the Lewy body dementia community on risks and benefits of lumbar punctures in LBD biomarker research | Bespoke online survey assessing knowledge and attitudes about lumbar puncture from people with LBD and their caregivers. The survey included true/false and multiple-choice questions. There was no reference to free-text response options. | None | Not applicable |
| Behavioral and psychological effects of coronavirus disease-19 quarantine in patients with dementia | A structured, bespoke telephone interview survey was conducted with family caregivers of PLWD. The survey consisted of closed-ended questions. Variations in BPSD were recorded one month after the quarantine declaration, and associations with disease type, severity, gender, and caregiver’s stress burden were analysed. | None | Not applicable |
| Video research visits for atypical parkinsonian syndromes among Fox Trial Finder participants | Following a real-time video clinic visit, participants completed a bespoke survey of participant satisfaction. Eight questions were answered either on a Likert scale or as a binary yes/no. Two questions had free text responses. | None | Not applicable |
| Support and information needs following a diagnosis of dementia with Lewy bodies | A retrospective, bespoke, publicly available web survey was utilised, comprising ten short questions on past experiences with support and information, difficulties that could benefit from additional resources, and relevant topics for future resource development. The survey included both multiple-choice and free-text response options. | None | Not applicable |
| Characteristics of eating and swallowing problems in patients who have dementia with Lewy bodies | A revised version of a questionnaire originally designed to assess eating/swallowing problems in patients with FTD/AD was used [3]. Four additional questions added that were relevant to DLB and seven questions relating to FTD were deleted (closed-ended Likert scale questions) | None | Not applicable |
| Pain in extrapyramidal neurodegenerative diseases | Clinical interview involving the administration of a structured, bespoke questionnaire which asked patients whether they experienced low-back pain and whether they experienced pain other than low-back pain. Those who answered affirmatively to the second query were asked to specify time of pain onset, pain localization and frequency, and other pain characteristics and these responses were categorised as (1) musculoskeletal (2) radicular/neuropathic, (3) dystonic or (4) central. | None | Not applicable |
| Self-efficacy and social support for psychological well-being of family caregivers of care recipients with dementia with Lewy bodies, Parkinson’s disease dementia, or Alzheimer’s disease | Utilised an internet-based survey comprising standardised measures. The survey was not available. Scales were referred to and there was no reference to free-text responses. | - RSCSE - MOS-SS - PHQ2 - ZBI (12-item) - MM-CGI-SF - C-PWBS | Not applicable |
| Factors of dementia caregiver burden differentially contribute to desire to institutionalize | Self-reported standardised instruments were reported by caregivers in the format of an online questionnaire. | - DIS | Not applicable |
| Caregiver self-efficacy and associated factors among caregivers of patients with dementia with Lewy bodies and caregivers of patients with Alzheimer’s disease | A comprehensive caregiver questionnaire was conducted, capturing perspectives through assessment instruments. (questionnaires). Caregivers completed all self-report questionnaires, while psychiatrists administered the NPI. Caregiver self-efficacy was evaluated using three domains of the RSCSE (Self-Efficacy for Obtaining Respite, self-Efficacy for Responding to Disruptive Patient Behaviour, and self-Efficacy for Controlling Upsetting Thoughts about Caregiving). Additional data on caregiver depression, sleep disturbance, burden, executive function, loneliness, social support, and distress associated with neuropsychiatric symptoms were also assessed. | - The NPI-D - The BDI - The ISI - The ZBI (22-item Japanese version) - The RSCSE - The R-UCLA-LS - The SSQ | Not applicable |
| Comparison of QOL between patients with different degenerative dementias, focusing especially on positive and negative affect | Scores of the QOL-D were evaluated by a trained clinical psychologist based on the information from family care partners. | - QOL-D | Not applicable |
| Patients with Lewy body dementia use more resources than those with Alzheimer’s disease | People with DLB were either examined in their homes or at the clinic together with their primary caregiver. Resource use was measured using a standardised instrument (the RUD Lite). | - RUD-Lite | Not applicable |
| Examining carer stress in dementia: the role of subtype diagnosis and neuropsychiatric symptoms | Care partners were interviewed using the NPI-D. | - NPI-D | Not applicable |
| Attitudes toward own aging and cognition among individuals living with and without dementia: findings from the IDEAL programme and the PROTECT study | Cross-sectional data from the IDEAL and PROTECT studies were used to compare attitude towards own aging between PLWD and individuals without dementia. A standardised measure was used in both studies. In the IDEAL cohort, this questionnaire was administered as part of a questionnaire administered during interviews. In the PROTECT cohort, the measure was self-completed through the PROTECT online platform. | - The ATOA questionnaire | Not applicable |
| Multidimensional care burden in Parkinson-related dementia | Life partners participated in home-based quantitative assessments or self-completed postal questionnaires, which comprised the assessment battery. The assessment battery included the ZBI. Results of an exploratory factor of the ZBI is reported. | - ZBI (22-item) | Not applicable |
| Autonomic symptoms are predictive of dementia with Lewy bodies | A sexual score was obtained from the SCOPA-Aut questionnaire which assessed the domain of sexual dysfunction. The occurrence of each symptom was scored at 0 (never), 1 (sometimes), 2 (regularly), or 3 (often), allowing the severity rating. In the current study, they combined men’s erectile and ejaculation problems and women’s vaginal lubrication and orgasm problems and analysed them as an item of sexual dysfunction. | - SCOPA-Aut | Not applicable |
| Care burden and mental ill health in spouses of people with Parkinson disease dementia and Lewy body dementia | Data from 57 spouses were extracted from the INVEST study. Additionally, 79 spouses participated in the postal questionnaire study. Spouses completed a battery of rating scales, which were administered either during baseline assessments in the INVEST study (conducted via a home-based interview between the researcher and the couple), or through a postal questionnaire for spouses only. The measures were of mood, stress, resilience, general health, quality of life, care burden, and relationship satisfaction, and sociodemographic factors. | - The NPI-D - ZBI (22-item) - The Rel.SS - The DRS - The FCR scale - The BRS - The RSS - The SF-12 - HADS - EQ-5D-3L | Not applicable |
| Capgras syndrome in dementia with Lewy bodies | Semi-structured interviews were conducted with the patient and an informant. Caregivers were assessed for caregiver burden and depression using standardised measures. | - ZBI (12-item) - PHQ-9 | Not applicable |
| Caregiver burden in family carers of people with dementia with Lewy bodies and Alzheimer’s disease | Caregiver burden was rated by the Rel.SS | - Rel.SS | Not applicable |
| Patients with dementia with Lewy bodies have more impaired quality of life than patients with Alzheimer disease | Two standardised measures assessing quality of life were administered. Both instruments were administered to both patients and care partners as proxies. | - EQ-5D-3L - QoL-AD | Not applicable |
| Study of prevalence of neuropsychiatric symptoms in elderly dementia patients | A clinician assessed care partner stress using the ZBI. | - ZBI (22-item) | Not applicable |
| The association between specific neuropsychiatric disturbances in people with Alzheimer's disease and dementia with Lewy bodies and carer distress | The Rel.SS assessed the level of reported distress in care partners. The effect of neuropsychiatric symptoms on care partner distress was analysed. | - Rel.SS | Not applicable |
| Association of premorbid personality with behavioral and psychological symptoms in dementia with Lewy bodies: Comparison with Alzheimer’s disease patients | In a face-to-face clinical interview with a hospital clinical psychologist, family members completed the NEO-FFI to assess each patient’s premorbid personality. | - NEO-FFI | Not applicable |
| Visual hallucinations in eye disease and Lewy body disease | Data from five controlled cross-sectional studies were combined and analysed. The prevalence, phenomenology, frequency, duration, and contents of visual hallucinations, assessed using the NEVHI, were compared across diseases and sex. | - NEVHI | Not applicable |
| Cognition, hallucination severity and hallucination-specific insight in neurodegenerative disorders and eye disease | Extant samples of patients experiencing visual hallucinations were included in the analyses. In the included studies, patients had been administered the NEVHI (secondary analysis). | - NEVHI | Not applicable |
| Health related quality of life in individuals with cognitive decline and discrepancies between patients and their proxies | HRQoL was measured via the SF-12-v2 separately for participants and proxies. The questionnaires were self-reported, but the evaluator was present to clarify any doubts participants might have. | - SF-12-v2 | Not applicable |
| Health related quality of life and cognitive decline in older populations: preliminary results from NeuroDemeNPsia study | HRQoL indices obtained via the SF-12-v2 were compared between different subgroups. Both patient’s and informant’s questionnaires were collected for all groups. Informants were asked to answer the questions as if they were the patients themselves. | - SF-12-v2 | Not applicable |
| Caregiver burden, sleep quality, depression, and anxiety in dementia caregivers: a comparison of frontotemporal lobar degeneration, dementia with Lewy bodies, and Alzheimer’s disease | Patients were compared in terms of neuropsychiatric symptoms, while care partners were compared with respect to burden, sleep quality, anxiety and depression, all of which were assessed using standardised measures. | - ZBI (22-item) - PHQ-9 - GAD-7 - PSQI | Not applicable |
| Patient affect and caregiver burden in dementia | Standardised assessment instruments assessed caregiver burden and the patient’s positive affect. | - QOL-D - J-ZBI-8 | Not applicable |
| Comparison of caregiver burden between dementia with Lewy bodies and Alzheimer’s disease | Caregiver burden was assessed by the J-ZBI and compared between AD and DLB caregivers. | - J-ZBI (22 item) | Not applicable |
| Impact of behavioral and psychological symptoms on caregiver burden in patients with dementia with Lewy bodies | Collected data on caregiver burden using a standardised measure with the aim of exploring which behavioural and psychological symptoms affect caregiver burden. | - ZBI (22-item) | Not applicable |
| Factors associated with burden among male caregivers for people with dementia | Data were collected from a national dementia registration survey. Caregiver burden was measured using a standardised measure (ZBI). | - ZBI (22-item) | Not applicable |
| Differential associations of clinical features with cerebrospinal fluid biomarkers in dementia with Lewy bodies and Alzheimer’s disease | A standardised measure assessed caregiver burden (ZBI). Associations with tau and a-synuclein were investigated. | - ZBI (22-item Brazilian version) | Not applicable |
| Clinical findings, functional abilities and caregiver distress in the early stage of dementia with Lewy bodies (DLB) and Alzheimer’s disease (AD) | The NPI-12 item scale (standardised instrument) was reported which included ratings of caregivers’ emotional distress caused by each neuropsychiatric symptom as well as a total caregiver distress score. | - NPI-D | Not applicable |
| The effects of behavioral and psychological symptoms on caregiver burden in frontotemporal dementia, Lewy body dementia, and Alzheimer's disease: clinical experience in China | Caregiver burden was assessed using a standardised measure (ZBI). A semi-structured questionnaire was used to assess variables identified in the literature to influence caregiver burden, health, and emotional well-being. | - ZBI (22-item) | Not applicable |
| Subjective experience of time in dementia with Lewy bodies during COVID-19 lockdown | Experimenter interviewed each participant by telephone call using the STQ to assess the subjective experience of the passage of time for present and past time intervals during the lockdown due to coronavirus disease in 22 patients with DLB (17 of which were re-tested in a post-lockdown period). Their experience was compared with that of 14 caregivers with similar age. | - STQ | - Method: interview - Format: telephone - Design: used a shortened Italian version of the STQ. The questionnaire is composed by two groups of items to be answered on Likert scale. Part 1 is “Personal Time Experience of Present and Past”. Part 2 is “Statements on Subjective Time Experience”. - Relationship between researcher and participant: not reported - Method of qualitative analysis: not applicable - Duration of time (minutes): The STQ took an average administration time of ten minutes per participant. - How were dyadic interviews managed: not applicable |
| A brief psychometric and clinimetric evaluation of self-report burden and mental health measures completed by care partners of people with Parkinson's-related dementia | Data were obtained from a cross-sectional study with informal care partners of people with PD-MCI, PDD, or DLB, nested within the INVEST trial. The psychometric and clinimetric properties of care partner-reported measures were explored. | - The ZBI (22-item) - The Rel.SS - The DRS - The FCR scale - The BRS - The RSS - The SF-12 - The HADS - The EQ-5D-3L | Not applicable |
| Tau in dementia with Lewy bodies | Standardised measures of general quality of life (EQ-5D-3L) and caregiver burden (ZBI) were reported. | - EQ-5D-3L and VAS - ZBI (22-item Brazilian version) | Not applicable |
| Correlates of neuropsychiatric and motor tests with language assessment in patients with Lewy body dementia | As part of a clinical assessment, sleep satisfaction was assessed using a non-standardised measure, and caregiver burden was assessed using a standardised measure. | - ZBI (22-item Brazilian version) | Not applicable |
| Association between amyloid-beta deposition and cortical thickness in dementia with Lewy bodies | Study partners completed a standardised measures of caregiver burden and general quality of life. | - EQ-5D-3L and VAS - ZBI (22-item Brazilian version) | Not applicable |
| Neuropsychiatric feature profiles of patients with Lewy body dementia | Sleep satisfaction was assessed using a non-standardised measure, while caregiver burden and distress were assessed using standardised measures as part of the clinical assessment | - ZBI (22-item Brazilian version) - NPI-D | Not applicable |
| Delusions in patients with dementia with Lewy bodies and the associated factors | A retrospective study of outpatients with DLB registered in a regional hospital’s database was performed. All patients and their main caregivers were interviewed for assessment of the NPI domain of delusions. All of the 12 NPI domains were rated for symptom frequency, symptom severity and caregiver burden. | - NPI-D | Not applicable |
| Contrasts between patients with Lewy body dementia syndromes and APOE- ε3/ε3 patients with late-onset Alzheimer Disease dementia | As part of a clinical assessment, sleep satisfaction was assessed using a non-standardised measure, and caregiver burden was measured using a standardised measure. | - ZBI (22-item Brazilian version) | Not applicable |
| Neuropsychological study of amyotrophic lateral sclerosis and parkinsonism-dementia complex in Kii peninsula, Japan | Clinical interview performed with no assessment instruments or quantitative scoring. Patients and their care partners were asked to report any history of abulia, apathy, hallucinations, personality changes, and other symptoms. | None | Not applicable |
| Clinical features of delusional jealousy in elderly patients with dementia | Clinical interview performed with no assessment instruments or quantitative scoring. Each patient with delusional jealousy and their primary care partner were interviewed about the clinical features of the syndrome. | None | Not applicable |
| Depression in dementia with Lewy bodies: a comparison with Alzheimer’s disease | To capture perspectives, they compared each depression symptom in DSM-IV major depression criteria between DLB and AD groups. Of interest here is suicidality. | None | Not applicable |
| Costs of dementia with Lewy bodies: A Chinese multicenter cross-sectional study | All consecutive patients with DLB and their caregivers were interviewed in person at each centre during the study period. Clinical information, statistics on the costs of the disease over a 1-year period, and details from caregiver interviews were collected. Electronic medical records were used to gather data on specialised inpatient and outpatient healthcare costs. When information could not be obtained from the electronic medical records system, investigators conducted face-to-face interviews using a standard electronic questionnaire. Caregiver burden, assessed using the ZBI, was also reported. | - ZBI (22-item) | Only when necessary were face-to-face interviews conducted by investigators using a unified standard electronic questionnaire. |
| The frequency and correlates of anxiety in patients with first-time diagnosed mild dementia | Symptoms of anxiety were rated by a caregiver on the NPI which included an assessment of carer distress in relation to the symptoms. The Rel.SS was also reported to assess caregiver burden when taking care of a PWLD. | - NPI-D - Rel.SS | Not applicable |
| **2B. Longitudinal designs (n=12)** | | | |
| Characterization of symptoms and determinants of disease burden in dementia with Lewy bodies: DEvELOP design and baseline results | Assessed disease burden based on three outcomes, two of which were quality of life and caregiver burden. These were evaluated using standardised measures. | - QoL-AD - ZBI (22-item) | Not applicable |
| Patterns of carer distress over time in mild dementia | The Rel.SS assessed the level of reported distress in care partners. 3 years of annual follow-ups were performed. | - Rel.SS | Not applicable |
| Time until nursing home admission in people with mild dementia: comparison of dementia with Lewy bodies and Alzheimer’s dementia | Caregiver's distress was measured using the Rel.SS. The patients were followed until the study termination date, death, exclusion, or nursing home admission. | - Rel.SS | Not applicable |
| Trajectories and determinants of quality of life in dementia with Lewy bodies and Alzheimer’s disease | Patients were invited annually for follow-up for at least two years. In both cohorts, participants completed a standardised measure of quality of life. | - EQ-5D-3L (and VAS) | Not applicable |
| Disease progression in dementia with Lewy bodies: a longitudinal study on clinical symptoms, quality of life and functional impairment | Participants in DEvELOP underwent assessments and were invited for annual follow-ups for at least one year. These annual assessments included standardised measures of caregiving burden (ZBI) and quality of life (QoL-AD). | - QoL-AD - ZBI (22-item) | Not applicable |
| Personality traits distinguishing dementia with Lewy bodies from Alzheimer disease | Examined the clinical records of participants enrolled in a longitudinal study that included annual assessments of personality from PLWD as well as nondemented individuals, some of whom eventually developed AD or DLB. Individuals were followed to autopsy. As part of the annual interview, the clinician asked the informant about specific changes in personality, interests, and drives, based on items from the BDS. | - BDS | Not applicable |
| Frequency of suicidal ideation and associated clinical features in Lewy body dementia | Retrospective review of a prospectively collected database at a tertiary movement disorders clinic. Database participants with an LBD diagnosis at their most recent visit and at least one complete BDI-II were included. BDI-II data was used to assess suicidal ideation. Additional standardised measures included the PDQ-39. | - BDI-II - PDQ-39 | Not applicable |
| A comparison of caregiver burden for different types of dementia: an 18-month retrospective cohort study | The care team performed face-to-face interviews every 6 months for 18 months from the time of the dementia diagnosis, during which various instruments were administered. Among these, caregiver burden was assessed using the ZBI. | - ZBI (22-item Croatian version) | Not applicable |
| Impact of the COVID-19 Pandemic and Lockdown on Anxiety, Depression and Nursing Burden of Caregivers in Alzheimer’s Disease, Dementia with Lewy Bodies and Mild Cognitive Impairment in China: a 1-Year Follow-Up Study | A survey was repeated at 1-year follow up. The survey consisted of caregiver standardised and non-standardised measures. The non-standardised measures were a semi-structured questionnaire measuring social contact and a semi-structured health-related questionnaire assessing physical activity and caring time. | - ZBI (22-item) - GAD-7 - PHQ-9 - PSQI | Not applicable |
| Prodromal dementia with Lewy bodies: clinical characterization and predictors of progression | The clinical assessment included a measurement of caregiver burden using the ZBI. | - ZBI (22-item) | Not applicable |
| Updates on somatoform disorders (SFMD) in Parkinson's disease and dementia with Lewy bodies and discussion of phenomenology | SFMD assessments were based on multiple methods: DSM-IV-TR-based semi-structured interviews with patients and caregivers, focusing on somatic complaints using examples and a checklist; and neuropsychological scale ratings, including the Somatisation Subscale of the SCL-90-R. | - SCL-90R Somatisation Subscale | Not applicable |
| Cohort study on somatoform disorders in Parkinson disease and dementia with Lewy bodies | Patients were categorised into somatoform disorder (SFMD) and non-SFMD groups based on multiple methods, one of which was a DSM-IV-TR-based semi-structured interview that investigated somatic complaints with examples and a checklist presented to both patients and caregivers, focusing on SFMD traits. Two comparison studies were conducted between patients with PD and those with DLB, both with and without SFMD. In the first study, patients were evaluated using multiple standardised scales, including the SCL-90R Somatisation Subscale. | - SCL-90R Somatisation Subscale | Not applicable |
| **2C. Trial designs (n= 11)** | | | |
| Memantine for patients with Parkinson’s disease dementia or dementia with Lewy bodies: a randomised, double-blind, placebo-controlled trial | Caregiver burden, assessed using the ZBI, was included as a trial outcome (no primary outcomes was defined). | - ZBI (22-item) | Not applicable |
| Music playlists for people with dementia: trialling a guide for caregivers | DLB-related data was collected only through the MiDAS which assessed changes in mood and behaviour at each listening session. This was completed prior and after each listening session by a caregiver or family member. | - MiDAS | Not applicable |
| Bilateral nucleus basalis of Meynert deep brain stimulation for dementia with Lewy bodies: A randomised clinical trial | Trial endpoints included caregiver distress, assessed using the NPI-D, and the quality of life of both patients and caregivers, assessed using the QoL-AD. | - NPI-D - QoL-AD | Not applicable |
| Adjunct zonisamide to levodopa for DLB parkinsonism: A randomized double-blind phase 2 study | Caregiver burden, measured using the ZBI, was included as a secondary trial endpoint. | - J-ZBI (22 item) | Not applicable |
| Donepezil for dementia with Lewy bodies: a randomized, placebo-controlled trial | Caregiver burden, measured using the ZBI, was identified as a core efficacy outcome, although no formal predefined primary endpoint was selected. | - ZBI (22-item) | Not applicable |
| Quality of life and the effect of memantine in dementia with Lewy bodies and Parkinson’s disease dementia | Conducted a secondary analysis of a randomized controlled study over 24 weeks using caregiver-rated QOL- AD in domains according to the World Health Organisation's classification of health. | - QoL-AD | Not applicable |
| Long-term donepezil use for dementia with Lewy bodies: results from an open-label extension of Phase III trial | Caregiver burden, measured using the ZBI, was included as a core trial outcome. | - ZBI (22 item) | Not applicable |
| Efficacy of adjunctive therapy with zonisamide versus increased dose of levodopa for motor symptoms in patients with dementia with Lewy bodies: the randomized, controlled, non-Inferiority DUEL study | Secondary trial endpoints included the ZBI and the EQ-5D-5L. | - ED-5D-5L - ZBI (22 item) | Not applicable |
| Efficacy, safety, and tolerability of armodafinil therapy for hypersomnia associated with dementia with Lewy bodies: a pilot study | The quality of life of patients and caregivers was measured using the LASA, which was included as a secondary efficacy measure. | - LASA | Not applicable |
| A comparison of the efficacy of donepezil in Parkinson’s disease with Dementia and Dementia with Lewy bodies | The NPI-D was included as a primary outcome measure. | - NPI-D | Not applicable |
| Long-term safety and efficacy of donepezil in patients with dementia with Lewy bodies: results from a 52-week, open-label, multicenter extension study | Caregiver burden, measured using the ZBI, was assessed at 0, 24 and 52 weeks. No formal primary endpoint was predefined in the analysis due to the exploratory nature of the study. | - ZBI (22-item) | Not applicable |
| **2D. Case reports/case series designs (n= 7)** | | | |
| Lumateperone for treatment of psychotic symptoms in Lewy body: A case report | Distress associated with neuropsychiatric symptoms was measured using the NPI. The source also alludes to patients and care partner/family comments. Informal feedback from family members also reported. | - NPI-D | Not applicable |
| Cognitive effects of quetiapine in a patient with dementia with Lewy bodies | Distress associated with neuropsychiatric symptoms was measured using the NPI. | - NPI-D | Not applicable |
| Open label trial to evaluate the efficacy and safety of Yokukansan, a traditional Asian medicine, in dementia with Lewy bodies | The J-ZBI was included as a trial outcome to assess caregiver burden. | - J-ZBI (22-item) | Not applicable |
| Improvement in delusions and hallucinations in patients with dementia with Lewy bodies upon administration of yokukansan, a traditional Japanese medicine | The burden of BPSD on caregivers was evaluated with the J-ZBI. | - J-ZBI (22 item) | Not applicable |
| Effectiveness of ramelteon for treatment of visual hallucinations in dementia with Lewy bodies: a report of 4 cases | The effectiveness of ramelteon on caregiver burden was measured using the ZBI. | - ZBI (22-item) | Not applicable |
| Administration of zonisamide in three cases of dementia with Lewy bodies | Measures administered after 12 weeks of zonisamide administration included the ZBI to assess caregiver burden. | - ZBI (22-item) | Not applicable |
| Donepezil for treatment of dementia with Lewy bodies: a case series of nine patients | Retrospectively used observational data toreview consecutive patients with DLB who had been treated with donepezil. Comments from family members were reported. | None | Not applicable |
| **3. Mixed methods (n=20)** | | | |
| **3A. Methodological design (n=1)** | | | |
| Development and validation of the Lewy body disease caregiver activities scale | To assess how the target group perceived the instrument, six participants from a prior group of 20 caregivers, who had participated in qualitative interviews in Step 2, evaluated the instrument’s face validity through think-aloud interviews. During these interviews, participants completed the instrument and were asked to verbalise their thoughts, commenting on the clarity, relevance, appropriateness, and wording of the items. Probes were used to gain further insight when participants hesitated or indicated a lack of understanding. | None | - Method: Think aloud interviews - Relationship between researcher and participant: Not reported, but participants known to researcher from interviews at earlier steps in the study - Method of qualitative analysis: not reported - Duration: not reported - How were dyadic interviews managed: not applicable |
| **3B. Trial designs (n=8)** | | | |
| Goal-orientated cognitive rehabilitation for dementias associated with Parkinson's disease- A pilot randomised controlled trial | After goal setting (reported elsewhere [4]), participants were randomised to cognitive rehabilitation, relaxation therapy, or treatment‐as‐usual. The trial's primary outcome was ratings of goal attainment and satisfaction with goal attainment. This was measured using the BGSI. The trial's secondary outcomes included relevant standardised measures reported by people with LBD and care partners. | For patients:   - BGSI - PQD-8 - ED5D3L - WHOQOL-BREF - GSES.   For care partners:   - BGSI - HADS - GSES - WHOQOL-BREF - EQ5D3L - NPI-D - The Rel.SS. | - Method: BGSI interview – details reported elsewhere [4] |
| Goal setting for cognitive rehabilitation in mild to moderate Parkinson’s disease dementia and dementia with Lewy bodies | Participants were recruited for an ongoing single-blind pilot trial. During the baseline visits, participants identified up to three rehabilitation goals and rated their performance and satisfaction with these goals using the BGSI, a semi-structured interview. This report examines the goal statements provided by participants, using qualitative content analysis to explore the types and nature of the goals set. | - BGSI | - Method: interview based on the BGSI - Format: face-to-face - Design: semi-structured - Relationship between researcher and participant: not reported - Method of qualitative analysis: qualitative content analysis was applied - Duration of time (minutes): not reported - How were dyadic interviews managed: not reported |
| Parkinson’s-adapted cognitive stimulation therapy: a pilot randomized controlled clinical trial | The primary outcomes of the overall INVEST study were tolerability, acceptability, and feasibility. This source reports a range of exploratory participant (patient) and care-partner standardised outcome measures. Qualitative outcomes, based on observational data from participant–dyad diaries (which recorded therapy adherence and therapy acceptability, but are reported elsewhere), completed after each therapy session, and semi-structured interviews (using a bespoke guide) in a sub-sample of the CST-PD group, were also included. | For patients:   - The PDQ-39 - The EQ-5D-3L - The Rel.SS - The RSS - The BRS - The IRI   For care partners:   - The EQ-5D-3L - The HADS - The SF-12 - The Rel.SS - The RSS - The DRS - The FCR - The ZBI (22 item) - The NPI-D - The BRS | - Method: interviews - Format: - Design: semi-structured - Relationship between researcher and participant: not reported - Method of qualitative analysis: thematic analysis - Duration of time (minutes): not reported - How were dyadic interviews managed: not applicable - Method: observational data from participant-dyad diaries - Design: semi-structured - Method of qualitative analysis: thematic analysis |
| Parkinson’s-adapted cognitive stimulation therapy: feasibility and acceptability in Lewy body spectrum disorders | Care partners' perceptions of their partner's interest, motivation, and sense of achievement were assessed following each therapy session. These aspects were rated in a therapy diary on a Likert-scale. To assess care partners’ ability to deliver the intervention, measures such as a training evaluation form, a therapy skills self-assessment, and diary entries (including acceptability ratings and free-text entries) were used. Post-intervention, semi-structured interviews were conducted with a purposive sample of eleven participant-dyads who undertook the therapy. | Scales reported elsewhere | - Method: (1) post-intervention interviews, and (2) therapy diaries - Format: not reported - Design: semi-structured interviews - Relationship between researcher and participant: not reported - Method of qualitative analysis: thematic analysis - Duration of time (minutes): not reported - How were dyadic interviews managed: not reported |
| The feasibility and acceptability of a psychosocial intervention to support people with dementia with Lewy bodies and family care partners | Interviews were conducted with participants to optimise a third intervention group. Each dyad also discussed their experiences in a semi-structured, home-based interview with an intervention facilitator. To meet the secondary objectives, participants completed pre- and post-intervention standardised measures of self-efficacy. As part of identifying the most appropriate primary outcome measure for future use, interview feedback was gathered regarding their acceptability. Additional pre- and post- standardised and non-standardised measures were also administered. The non-standardised measure was a 4-point Likert scale evaluation in in which participants indicated perceived changes in their capability to cope in each topic areas. | - GSES - **FCP-SEMD Scale** - RSCSE - GDS-15 - PSS - Rel.SS | - Method: 15 dyads were interviewed post-intervention - Format: face-to-face/ home-based - Design: semi-structured - Relationship between researcher and participant: intervention facilitator conducted the interviews - Method of qualitative analysis: thematic analysis - Duration of time (minutes): 30-45 minutes - How were dyadic interviews managed: not reported |
| Learning to PERSEVERE: a pilot study of peer mentor support and caregiver education in Lewy body dementia | Focus groups (using a bespoke guide) guided intervention material refinement. An online screening mentor-mentee matching questionnaire obtained mentor-mentee matching preferences. Biweekly, online questionnaires (online diaries) assessed satisfaction, matching priorities, the proportion of participants matched on their top priority, call frequency and duration, and handbook use. During the week 16 survey, participants were asked if they would recommend PERSEVERE to other caregivers, and if they would consider mentoring in the future. Pre and post standardised and non-standardised measures were also employed. These included an investigator-generated, non-standardised measures of satisfaction as well standardised measures. | - DAS - PMS - HADS - GDS-15 - ZBI (12 item) - UCLA-LS | - Method: Focus groups - Format: virtual - Design: semi-structured - Relationship between researcher and participant: not reported - Method of qualitative analysis: Framework analysis - Duration of time (minutes): 90-120 minutes - How were dyadic interviews managed: Not applicable |
| Tailoring and evaluating the web-based ‘Partner in Balance’ intervention for family caregivers of persons with young-onset dementia | After the intervention, participants were interviewed for approximately 60 minutes in-person or by telephone using the PPQ (a semi-structured interview developed for the Partner in Balance study [5]). This was a novel interview questionnaire designed for the Partner in Balance study. | None | - Method: Interview - Format: in-person or by telephone - Design: Semi-structured by means of the PPQ - Relationship between researcher and participant- not reported - Method of qualitative analysis: a deductive qualitative content analysis was performed using fieldnotes to interpret the quantitative scores on the PPQ. - Duration of time needed: approximately 60 minutes - How were dyadic interviews managed: Not applicable |
| Introduction of a management toolkit for Lewy body dementia: a pilot cluster-randomized trial | People with PDD or DLB underwent trial assessments. There was no prespecified primary outcome, but standardised measures were reported. Health economic data on the use of health and social care resources were captured using a bespoke service use questionnaire that was developed based on questions included in the CSRI and administered to care partners at baseline and 3 and 6 months. The participants were asked about their use of a broad range of services including in-patient services, out-patient services, day activity services, and community care services during the preceding 3 months. | For patients:   - DEMQOL - EQ-5D-5L   For care partners:   - ZBI (22 item) - HADS - EQ-5D-5L | - Not applicable |
| **3C. Cross-sectional designs (n= 7)** | | | |
| Measuring disability in patients with neurodegenerative disease using the ‘Yesterday Interview’ | A semi-structured interview method (the 'Yesterday Interview') was used to reconstruct the preceding 24-hour period in terms of activity, social and environmental context, and subjective enjoyment. | - The ‘Yesterday Interview’ | - Method: interviews - Format: face-to-face in participant’s own home - Design: semi-structured using the ‘yesterday interview’ - Relationship between researcher and participant: not reported - Method of qualitative analysis: Every action or activity elicited by interview was identified in the ICF manual and assigned to one of the seven ICF domains. - Duration of time (minutes): up to 30 minutes - How were dyadic interviews managed: The patient was the primary informant, although the carer was invited to supplement, clarify or correct the information as appropriate. |
| “I felt like I had been put on the shelf and forgotten about” – lasting lessons about the impact of COVID-19 on people affected by rarer dementias | An 11-question online survey, collecting both qualitative and quantitative data, was administered to PLWD and family care partners. Three versions of the survey were created to address different respondent types: 1) PLWD, 2) Care partners of PLWD in the community, and 3) Care partners of people residing in care homes. | None | Not applicable |
| Caring for people with dementia with Lewy bodies and Parkinson’s dementia in UK care homes – a mixed methods study | The aim was addressed in two ways: (1) Bespoke survey (collecting quantitative and qualitative data) completed by care home managers, and (2) a combination of individual interviews, paired interviews and focus groups with formal care partners. | None | - Method: individual interviews, paired interviews and focus groups - Format: face-to-face - Design: Two semi-structured topic guides were used in the interviews and discussion — one for managers/deputy managers and nurses, and one for other care staff. - Relationship between researcher and participant: not reported - Method of qualitative analysis: analysed thematically using framework analysis. - Duration of time (minutes): not reported - How were dyadic interviews managed**:** not applicable |
| A pilot study on the use of dolls for people with dementia | Fourteen dolls were introduced into two homes. After a minimum of 3 weeks, a questionnaire was administered to staff. The questionnaire included open and closed-ended questions. There were two questionnaire versions: (1) A five-item questionnaire containing general questions about the approach, and (2) A 14-item questionnaire, an extended version of the five-item questionnaire, comprising both quantitative and qualitative questions. The additional questions asked key workers to identify the impact on a specific resident. They were also required to hypothesise on the mechanisms of change occurring via the use of dolls and provide details of their overall impression of doll use. | None | Not applicable |
| Presence phenomena in parkinsonian disorders: phenomenology and neuropsychological correlates | Patients who endorsed presence phenomena were asked to complete a semi-structured interview about their experiences. A bespoke guide was used. The cognitive profiles of these patients were then compared to those of age- and education-matched patients who denied presence phenomena. | None | - Method: interview - Format: not reported - Design: semi-structured - Relationship between researcher and participant: not reported - Method of qualitative analysis: grounded theory - Duration of time (minutes): not reported - How were dyadic interviews managed: not applicable |
| Development of assessment toolkits for improving the diagnosis of the Lewy body dementias: feasibility study within the DIAMOND Lewy study | Qualitative interviews with patients. No reference to a guide. | None | - Qualitative interviews. No reference to a guide |
| Improving the diagnosis and management of Lewy body dementia: the DIAMOND-Lewy research programme including pilot cluster RCT | Work package five involved semi-structured interviews to explore perspectives on the acceptability of the toolkits. | None | - Work package 5: Qualitative interviews (no reference to a guide) |
| **3D. Case study designs (n=2)** | | | |
| Conversations in dementia with Lewy bodies: Resources and barriers in communication | The dyad’s perception of communicative function in daily life was explored through semi-structured interviews using a bespoke guide. Interactional patterns and participation in casual conversation was analysed with conversation analysis. | None | - Method: interview - Format: face-to-face - Design: semi-structured - Relationship between researcher and participant: not reported - Method of qualitative analysis: content analysis - Duration of time (minutes): the recorded interview and testing each lasted approximately 1 hour. - How were dyadic interviews managed: Responses were coded according to each participant. The two participants’ utterances were analysed and coded together, rather than separating their contributions, since the analysis aimed at arriving at a joint representation of their perspectives. |
| Preclinical polymodal hallucinations for 13 years before dementia with Lewy bodies | The patient was followed for 4 years, and a selective phenomenological and cognitive study was performed at the predementia stage. A clinical examination was performed. To collect perspective data, a semi-structured, open-ended interview was administered to describe the phenomenology of patient’s hallucinations. | None | - Method: interview - Format: Face-to-face - Design: Semi-structured - Relationship between researcher and participant: clinician(s) - Method of qualitative analysis: Not reported - Duration of time needed: Not reported - Managing dyadic interviews: Not applicable |
| **3E. Longitudinal study designs (n=2)** | | | |
| The use and costs of paid and unpaid care for people with dementia: Longitudinal findings from the IDEAL cohort | Used questionnaire survey data from waves 1 to 3 from the IDEAL programme. At each wave, PLWD completed questionnaires in face-to-face interviews and caregivers self-completed their questionnaires. The questionnaire survey was derived from short versions of available measures, or identification of sub-scales or single items with known psychometric properties from these measures. The survey included questions on personal and background details, capitals, assets and resources, challenges, access to and use of services (CSRI), adaption and living well outcomes. In this source, perspective data is collected via a standardised instrument (CSRI) which was included in the questionnaire survey. | CSRI | - Not applicable |
| Dementia subtype and living well: Results from the Improving the experience of Dementia and Enhancing Active Life (IDEAL) study | This study was based on the IDEAL baseline data. At baseline, PLWD completed questionnaires in face-to-face interviews and caregivers self-completed their questionnaires. The questionnaire survey was derived from short versions of available measures, or identification of sub-scales or single items with known psychometric properties from these measures. Perspective data was captured through the living well outcomes which were standardised, quantitative measures. | For patients:   - SwLS - WHO-5 - QOL-AD   For care partners:   - SwLS - WHO-5 - WHOQOL-BREFs | - Not applicable |
| **4. Not applicable (n=4)** | | | |
| Raising awareness and mutual support: using the internet | Article describes an interview between Nada Savitch and Ken Clasper who has a diagnosis of DLB. Ken is an active campaigner trying to raise awareness of dementia. One of the ways he does this is via the internet. Ken talks about his experiences of using various websites and internet services. | Not applicable | Not applicable |
| Parkinson’s/ Lewy body dementia: a carer’s perspective | The article reports a care partner’s perspective on the development of DLB, detailing the challenges and delays in obtaining a diagnosis, the complexities of arranging suitable care, and their recommendations based on personal experience. | Not applicable | Not applicable |
| Practical treatment of Lewy body disease in the clinic: Patient and physician perspectives | The article presents a clinician’s experiences with treatment strategies in DLB, developed over 15 years of collaboration between the same doctor and nurse, and illustrates these experiences with the perspectives of a patient and his wife. | Not applicable | Not applicable |
| My Friend Lewy | Article written from the perspective of a child reflecting on their experience of their parent living with DLB. The author discusses the symptoms and difficulty getting a diagnosis. | Not applicable | Not applicable |
| **Abbreviations:** People living with dementia (PLWD);Dementia with Lewy bodies (DLB); Lewy body dementia (LBD); Parkinson’s disease (PD); international Classiﬁcation of Functioning, Disability and Health model (ICF); INdiVidualised cognitivE Stimulation Therapy study (INVEST); the Improving the experience of Dementia and Enhancing Active Life (IDEAL); Platform for Research Online to investigate Genetics and Cognition in Aging (PROTECT); Conversation Analysis Profile for People with Cognitive Impairment (CAPPCI); Resource Utilization in Dementia - Lite Version (RUD-Lite); Brief Pain Questionnaire (BPI); Blessed Dementia Scale (BDS); The Music in Dementia Assessment Scale (MiDAS); Client Services Receipt Inventory (CSRI); Scales for Outcomes in PD-Autonomic (SCOPA-Aut); Satisfaction with Life Scale (SwLS); The WHO-5 Well-being Index (WHO-5); Dementia Attitudes Scale (DAS); Pearlin Mastery Scale (PMS); UCLA Loneliness Scale (UCLA-LS); Revised UCLA Loneliness Scale (R-UCLA-LS); The Prolonged Grief-12 (PG-12); Family Care Partner Self-Efficacy for Managing Dementia Scale (**FCP-SEMD Scale);** Geriatric Depression Scale (GDS-15); Perceived Stress Scale (PSS); Condensed version of the Psychological Well-Being Scale (C-PWBS); The Alzheimer Disease-related Quality of Life (ADRQL); Dementia Quality of Life Measure (DEMQOL); The Desire to Institutionalize Scale (DIS); Insomnia Severity Index (ISI); Social Support Questionnaire (SSQ); Subjective Time Questionnaire (STQ); The Attitude Toward Own Aging questionnaire (ATOA); NEO Five Factor Inventory (NEO-FFI); The Medical Outcome Study Social Support (MOS-SS); Perceived Change Index (PCI); 39-item Parkinson's Disease Questionnaire (PDQ-39); Beck Depression Inventory-II (BDI-II); Quality of life questionnaire for dementia (QOL-D); General Anxiety Disorder-7 (GAD-7); The Pittsburgh Sleep Quality Index(PSQI); Short-Form-8 (SF-8); World Health Organization Quality of Life-Brief Version (WHOQOL-BREF); Generalized Self-Efficacy Scale (GSES); The Bangor Goal Setting Interview (BGSI); Euro-Qol 5 dimensions 5-level (EQ-5D-5L); The North-East Visual Hallucination Interview (NEVHI); Short Form Health Survey, version 2 (SF-12-v2); The Marwit-Meuser Caregiver Grief Inventory Short Form (MM-CGI-SF); Patient Health Questionnaire-9 (PHQ-9); Revised Scale for Caregiving Self-Efficacy (RSCSE); The Relationship Satisfaction Scale (RSS); The 12-item short-form health survey (SF-12); The Dyadic Relationship Scale (DRS); The Family Caregiving Role Scale (FCR); The Brief Resilience Scale (BRS); Patient Health Questionnaire-2 (PHQ-2); Hospital Anxiety and Depression Scale (HADS); 12-item Zarit Burden Interview (ZBI-12); EuroQol- 5 Dimension (EQ-5D); Quality of Life in Alzheimer’s Disease (QoL-AD); Relative’s Stress Scale (Rel.SS); Neuropsychiatric Inventory caregiver distress scale (NPI-D); The Zarit Burden Interview (ZBI); Euroqol Questionnaire‐short version (ED5D3L);The Interpersonal Reactivity Index (IRI); Linear Analogue Self-Assessment Scale (LASA); Symptom Checklist 90R (SCL-90-R); Parkinson's Disease Questionnaire―8 (PDQ‐8); Japanese Zarit Burden Interview (J-ZBI); The Program Participation Questionnaire (PPQ) | | | |

**References:**

1. Clare L, Rowlands J, Bruce E, Surr C, Downs M. ‘I don’t do like I used to do’: A grounded theory approach to conceptualising awareness in people with moderate to severe dementia living in long-term care. Soc sci med. 2008;66:2366–77. https://doi.org/:10.1016/j.socscimed.2008.01.045

2. Bakas T, Austin JK, Okonkwo KF, Lewis RR, Chadwick L. Needs, Concerns, Strategies, and Advice of Stroke Caregivers the First 6 Months After Discharge: J Neurosci Nurs. 2002;34:242–51. https://doi.org/:10.1097/01376517-200210000-00004

3. Ikeda M. Changes in appetite, food preference, and eating habits in frontotemporal dementia and Alzheimer’s disease. J Neurol Neurosurg Psychiatry . 2002;73:371–6. https://doi.org/:10.1136/jnnp.73.4.371

4. Watermeyer T, Hindle J, Roberts J, Lawrence C, Martyr A, Lloyd-Williams H, et al. Goal setting for cognitive rehabilitation in mild to moderate Parkinson’s disease dementia and dementia with Lewy bodies. Parkinsons Dis. 2016;2016:8285041. https://doi.org/:10.1155/2016/8285041

5. Boots LM, De Vugt ME, Withagen HE, Kempen GI, Verhey FR. Development and Initial Evaluation of the Web-Based Self-Management Program “Partner in Balance” for Family Caregivers of People With Early Stage Dementia: An Exploratory Mixed-Methods Study. JMIR Res Protoc. 2016;5:e33. https://doi.org/:10.2196/resprot.5142

**Appendix 9**: Methods of recruitment and sampling

| **Title of evidence source** | **Source of recruitment** | **Sources of recruitment by category** | **Mechanism of recruitment** | **Mechanism of recruitment by category** | **Mechanism of sampling** |
| --- | --- | --- | --- | --- | --- |
| Research priorities of caregivers and individuals with dementia with Lewy bodies: an interview study | UF Health Norman Fixed Institute for Neurological Diseases | Healthcare and clinical settings | - Recruited when presenting for clinical visits - Consent-to-contact research database | - Clinical engagement - Contacted via consent-to-contact database | Not reported |
| Informal caregiver experiences at the end-of-life of individuals living with dementia with Lewy bodies: an interview study | Through an online survey which was advertised by the LBDA | Partner organisations | One survey question queried respondent willingness to participate in a telephone interview and if they answered affirmatively, they received the PI’s contact information to learn study details. Potential participants contacted the PI and received the informed consent form and semi-structured interview guide. | Participant self-initiated response (invite in survey) | Consecutive; purposive sampling |
| Living with dementia with Lewy bodies: an interpretative phenomenological analysis | Memory clinic | Healthcare and clinical settings | Senior clinician identified participants. PI then contacted the identified patients. | clinical engagement | Purposeful sampling |
| End-of-life experiences in dementia with Lewy bodies: qualitative interviews with former caregivers | Recruited through a survey that was advertised by the LBDA. | Partner organisations | Participants completing an online survey were asked at the end if they were willing to take part in a brief telephone interview on the topic. Respondents indicating “yes” received the PI’s contact information. | Participant self-initiated response (invite in survey) | Consecutive; purposive sampling |
| Caregiver-reported barriers to quality end-of-life care in dementia with Lewy bodies: a qualitative analysis | Recruited through a survey that was advertised by the LBDA. | Partner organisations | At the survey conclusion, respondents were asked if they were willing to participate in a 30-minute telephone interview. Willing respondents were given the PI’s contact information to learn study details. | Participant self-initiated response (invite in survey) | Consecutive; purposive sampling |
| Clinical care and unmet needs of individuals with dementia with Lewy bodies and caregivers: an interview study | A LBDA Research Centre of Excellence/ a tertiary referral centre | Healthcare and clinical settings | - Recruited consecutively at clinical visits - Through consent to contact | - Clinical engagement - Contacted via consent-to-contact database | Consecutive sampling |
| ‘It's just incredible the difference it has made': family carers' experiences of a specialist Lewy body dementia Admiral Nurse service | LBD Admiral Nurse Case Load | Healthcare and clinical settings | Eligible carers were informed about the study by the LBD Admiral Nurse. Interested carers were then sent an information pack containing an invitation letter signed by one of the researchers, along with an information sheet and consent form, reply slip and stamped envelope addressed to the researcher. | Clinical engagement | Not reported |
| The unique experience of spouses in early-onset dementia | The memory clinics of the centre for excellence in cognitive health of the integrated university health network of the Université de Montréal, which brings together various care centres | Healthcare and clinical settings | Participants recruited sequentially as they met the selection criteria. A healthcare provider was mandated in each clinical facility to contact potential participants to obtain their consent to forward their contact information to the project investigators. Thereafter, the project coordinator contacted the spouses to explain the purpose of the study, solicit their participation, and verify the inclusion criteria. | Clinical engagement | Consecutive sampling |
| Exploring the impact of caring for an individual with neurogenic orthostatic hypotension: a qualitative study | The agency identified potential participants through clinician referral, patient associations, patient research databases and social media. | - Healthcare and clinical settings - Partner organisations - Social networks | Specialist recruitment agency | Outreach by specialist recruitment agency | purposive sampling |
| Needs and concerns of Lewy body disease family caregivers: a qualitative study | Cognitive disorders centre in a metropolitan hospital | Healthcare and clinical settings | Potential participants were informed about the study during their visit to the centre or via a letter signed by the Medical Director, along with a study information sheet mailed to their home. Caregivers were contacted by telephone one week after the information was sent. | - Clinical engagement - Research team invite via telephone, e-mail and/or post | convenience |
| Understanding the nature and impact of cognitive fluctuations and sleep disturbances in dementia with Lewy bodies: a qualitative caregiver study | Recruited from specialist clinical services (e.g. memory clinics) in the north-east of England | Healthcare and clinical settings | Caregivers were initially approached by telephone or e-mail by a familiar member of the research team. | Research team invite via telephone, e-mail and/or post | Convenience |
| A qualitative study of female caregiving spouses’ experiences of intimate relationships as cognition declines in Parkinson’s disease | - Memory or movement disorder clinics - UK-based charity website - Join Dementia Research | - Healthcare and clinical settings - Partner organisations | Brochures in memory clinics  Advertisements on charity websites Advertisements on research recruitment platforms | Advertising (brochures, charity websites, recruitment platforms) | criterion sampling |
| Problems faced by people living at home with dementia and incontinence: causes, consequences and potential solutions | - Join Dementia Research - Dementia care groups - Care teams | - Partner organisations - Support groups - Healthcare and clinical settings | - Join Dementia Research advertisement - Invite via care groups - Invited via care teams | - Advertising (recruitment platform) - Clinical engagement | purposive sampling |
| Perceived benefits of using nonpharmacological interventions in older adults with Alzheimer’s disease or dementia with Lewy bodies | Participants enrolled following the intervention. The intervention recruited participants via caregiver support groups and other community meetings. | - Support groups - Community meetings | At completion of the interventions and data collection, caregivers whose family member with dementia had completed one of the three interventions were invited to the focus group to share their perspectives on the effects of the interventions. | Unclear/ not reported | not reported |
| Difficulties and associated coping methods regarding visual hallucinations caused by dementia with Lewy bodies | DLB specialist clinician's patients | Healthcare and clinical settings | Physician referral followed by informed consent during outpatient consultation | clinical engagement | not reported |
| Carers to people with Lewy body dementia and Alzheimer's disease: experiences and coping strategies | Outpatient clinics in western part of Norway | Healthcare and clinical settings | Carer partners were recruited by written letters sent by nurses at outpatient clinics. | clinical engagement | purposeful sampling |
| Key components of post-diagnostic support for people with dementia and their carers: a qualitative study | They selected six of the services identified during phase 1 for an in-depth case study. | - Healthcare and clinical settings - Partner organisations | People with dementia and carers were initially approached by gatekeepers and opted into the study by mail. | Invite via gatekeeper (unspecific approach) | purposive to identify sites |
| Goal setting for cognitive rehabilitation in mild to moderate Parkinson’s disease dementia and dementia with Lewy bodies | Movement Disorder Clinics and Memory Services. | Healthcare and clinical settings | Recruitment process was part of an ongoing single-blind pilot trial where potential participants were approached consecutively in clinics and invited to an initial screening interview with the researcher. | Clinical engagement | consecutive sampling |
| Measuring disability in patients with neurodegenerative disease using the ‘Yesterday Interview’ | specialist clinical services | Healthcare and clinical settings | unclear/ not reported | unclear/ not reported | not reported |
| Profiling conversation in Parkinson’s disease with cognitive impairment | Not reported | Not reported | unclear/ not reported | unclear/ not reported | not reported |
| The human need for equilibrium: qualitative study on the ingenuity, technical competency, and changing strategies of people with dementia seeking health information | Investigators’ networks which included members from peer support groups and large dementia advocacy organisations. | - Support groups - Partner organisations | The research team sent 30 potential participants an initial recruitment e-mail with details outlining the study and the criteria for participation in the study. | Research team invite via telephone, e-mail and/or post | convenience sampling snowball sampling |
| Applying an analytical process to longitudinal narrative interviews with couples living and dying with Lewy body dementia | - Voluntary sector services (Join Dementia Research, Parkinson's UK, Lewy Body Society, Alzheimer's Society and local hospices) - Support groups - Statutory service sector (memory clinics, care teams and the psychiatry liaison service within the local NHS trust) | - Partner organisations - Healthcare and clinical settings - Support groups | Relevant charities were approached to place a poster on their websites and display it at local support groups. The ‘Join Dementia Research’ added the study to its portfolio. Within the NHS trusts, initial contact was made through team managers to request clinical staff assistance in providing an introductory letter and participant information sheet to eligible participants. | - Advertising (organisation's website and at support groups) - Clinical engagement | A convenience sample |
| Problematising carer identification: a narrative study with older partner's providing end-of-life care | - Via two General Practitioner surgeries - Two former carers - Carers’ organisations. | - Healthcare and clinical settings - Via former carers - Support groups | Recruited via two General Practitioner surgeries, two former carers and the dissemination of a recruitment flyer to carers’ organisations. All potential participants were first contacted via the telephone to explain the study, to confirm their willingness to take part and arrange in-person meetings. | - Clinical engagement - Advertising - Research team invite via telephone, e-mail and/or post | Horizontal sampling |
| Pacemaker implants and their influence on the daily life of patients with dementia with Lewy bodies: a qualitative case study | Memory clinic | Healthcare and clinical settings | Senior physician/ co-author suggested participants | clinical engagement | Purposeful and criterion sampling |
| Using care navigation to address caregiver burden in dementia: a qualitative case study analysis | Care team navigators and participants in the Care Ecosystem programme at the time of the study were included | Healthcare intervention | Mechanism of recruiting Care team navigators is unclear. | Unclear/ not reported | For the case study, purposive; Complete enumeration for the interviews;  Unclear for CTNs |
| Cause of death and end-of-life experiences in individuals with dementia with Lewy bodies | LBDA | Partner organisations | LBDA website banner ad, a website study landing page, social media postings, and e-mails to individuals subscribed to the LBDA’s mailing list | - Advertising (organisation's website, organisation's social media) - E-mail invite via organisation's mailing list | not reported |
| COVID-19: Association between increase of behavioral and psychological symptoms of dementia during lockdown and caregivers’ poor mental health | - France Alzheimer Association - Memory centres - Social networks | Partner organisations Healthcare and clinical settings social networks | E-mails sent to members of the France Alzheimer association; unspecified mechanism of recruiting from memory centres; via social networks; information given during memory centre video appointments; visits carried out in the month following the end of the lockdown. | - E-mail invite via organisation's mailing list - Advertising (social networks)  clinical engagement | snowball sampling |
| Questionnaire survey of satisfaction with medication for five symptom domains of dementia with Lewy bodies among patients, their caregivers, and their attending physicians | Facilities in Japan, including university and non-university hospitals and clinics. | Healthcare and clinical settings | unclear/ not reported | unclear/ not reported | not reported |
| Lewy body dementia: the caregiver experience of clinical care | LBDA | Partner organisations | Survey posted on LBDA website. An announcement and an e-mail reminder 1-week prior to the survey closing was sent to all people subscribed to the LBDA's free e-newsletter | - Advertising (organisation's website) - E-mail invite via organisation's mailing list | not reported |
| Research priorities of individuals and caregivers with Lewy body dementia a web-based survey | LBDA | Partner organisations | Invitations to participate were distributed through the LBDA electronic mailing list. Additional recruitment was solicited through a banner ad on the LBDA landing page and through official LBDA social media posts. | - Advertising (organisation's website; organisation's social media) - E-mail invite via organisation's mailing list | not reported |
| Treatment needs of dementia with Lewy bodies according to patients, caregivers, and physicians: a cross-sectional, observational questionnaire-based study in Japan | Facilities in Japan, including university and non-university hospitals and clinics | Healthcare and clinical settings | Unclear/ not reported | unclear/ not reported | not reported |
| The impact of covid-19 quarantine on patients with dementia and family caregivers: a nation-wide survey | Centres for Cognitive Disorders and Dementia were recruiting centres | Healthcare and clinical settings | Clinical staff members of each centre consecutively contacted by phone the family caregivers of patients. | clinical engagement | consecutive sampling |
| The importance of educating the Lewy body dementia community on risks and benefits of lumbar punctures in LBD biomarker research | - LBD support groups - LBDA - Private Facebook group | - Support groups - Partner organisations | Survey distributed via e-mail invitation via LBD support groups, LBDA volunteers and a private Facebook group | - Advertising (social networks) - E-mail invite via organisation's mailing list | not reported |
| Behavioral and psychological effects of coronavirus disease-19 quarantine in patients with dementia | Centers for Cognitive Disorders and Dementia were recruiting centres | Healthcare and clinical settings | Invitation to participate in the survey was made through two Italian scientific societies involved in dementia care and research, the Italian Neurological Society for Dementia, and the Italian Association of Psychogeriatrics, to all their participants working in the Centres for Cognitive Disorders and Dementia. | Invite via healthcare research entities and scientific societies | not reported |
| Video research visits for atypical parkinsonian syndromes among Fox Trial Finder participants | Participants enrolled in the Fox Trial Finder | Partner organisations | Identified eligible Fox Trial Finder users and invited them to participate through a site posting and direct messaging to all users reporting one of these diagnoses via the site’s messaging system. A study coordinator contacted interested participants by phone. | - Advertising (via a research participation recruitment platform) - Direct messaging via research participation recruitment platform | not reported |
| The insula, a grey matter of tastes: A volumetric MRI study in dementia with Lewy bodies | Participants who were participating in a larger cohort study were enrolled. Patients were recruited from the tertiary memory clinic of the University Hospital of Strasbourg, France. | Healthcare and clinical settings | Unclear/ not reported | Unclear/ not reported | not reported |
| Pain in extrapyramidal neurodegenerative diseases | The Movement Disorders Outpatient Clinic of Rabin Medical Centre and Tel Aviv Sourasky Medical Centre. | Healthcare and clinical settings | Unclear/ not reported | Unclear/ not reported | not reported |
| Characteristics of eating and swallowing problems in patients who have dementia with Lewy bodies | The Higher Brain Function Clinic of the Department of Neuropsychiatry, Ehime University Hospital, Japan. | Healthcare and clinical settings | Recruited consecutive outpatients | Unclear/ not reported | consecutive sampling |
| Comparison of the caregiving experience of grief, burden, and quality of life in dementia with Lewy bodies, Alzheimer’s disease, and Parkinson’s disease dementia | LBDA; Alzheimer's Foundation of America; Family Caregiver Alliance, Alzheimer's Daily News; Parkinson's Disease Foundation; National Institute on Ageing Alzheimer Disease Centre Program | - Partner organisations - Healthcare and clinical settings | Advertisements on websites and social media  Contacted through caregiver e-mailing lists | - Advertising (organisations' websites and social media) - E-mail invite via organisation's mailing list | Not reported |
| Lewy body dementia: Caregiver burden and unmet needs | LBDA | Partner organisations | Visitors to the LBDA website. A cluster of respondents also came when the biannual LBDA newsletter was issued (n=150 responses), and a second cluster was in response to a reminder e-mail to the newsletter subscribers that the survey would end in the next week. | - Advertising (organisation's newsletter and organisation's website) - E-mail invite via organisation's mailing list | not reported |
| Pre-loss grief in caregivers of older adults with dementia with Lewy bodies | LBDA and partner caregiving organisations (mailing lists, website and social media page) | Partner organisations | Recruitment information was sent to individuals on caregiver email lists of organisations and was posted on their webpages and social media sites. Interested individuals were invited to participate by following a link to the online survey. | - Advertising (organisation’s website and organisation's social media) - E-mail invite via organisation's mailing list | convenience |
| Differences in the experience of caregiving between spouse and adult child caregivers in dementia with Lewy bodies | LBDA and partner organisations | Partner organisations | Contacted via LBDA and partner organisation e-mailing lists; Advertised on website; Advertised on social media website. Interested individuals followed link to the survey | - Advertising (organisation's website and organisation's social media) - E-mail invite via organisation's mailing list | Not reported |
| Dementia patients caregivers quality of life: the PIXEL study | Caregivers identified by referring clinicians | Healthcare and clinical settings | Voluntary patient/caregiver pairs were selected during an assessment by the medical doctor. | clinical engagement | Not reported |
| The role of sexual disinhibition to predict caregiver burden and desire to institutionalize among family dementia caregivers | Online from caregiver groups | Support groups | Study advertisement posted in 58 social media dementia caregiver groups | Advertising (social media dementia caregiver groups) | Not reported |
| Neuropsychological study of amyotrophic lateral sclerosis and parkinsonism-dementia complex in Kii peninsula, Japan | Not reported | Not reported | Unclear/ not reported | Unclear/ not reported | not reported |
| Clinical features of delusional jealousy in elderly patients with dementia | Two dementia clinics at Kumamoto University Hospital or Heisei Hospital | Healthcare and clinical settings | Patients selected from a consecutive series of patients | Unclear/ not reported | consecutive sampling |
| Costs of dementia with Lewy bodies: a Chinese multicenter cross-sectional study | Memory clinics | Healthcare and clinical settings | Eligible patients consecutively enrolled | Unclear/ not reported | consecutive sampling |
| Depression in dementia with Lewy bodies: a comparison with Alzheimer’s disease | Regional hospital | Healthcare and clinical settings | Consecutive series of outpatients | Unclear/ not reported | consecutive sampling |
| Patients with Lewy body dementia use more resources than those with Alzheimer’s disease | Centres in Sweden, Finland and Norway. | Healthcare and clinical settings | Patients were randomly selected from a sample of 272 patients from centres in Sweden, Finland and Norway. | Unclear/ not reported | random |
| Comparison of QOL between patients with different degenerative dementias, focusing especially on positive and negative affect | Outpatient unites of a memory centre | Healthcare and clinical settings | Patients consecutively recruited | Unclear/ not reported | consecutive sampling |
| Pain in patients with different dementia subtypes, mild cognitive impairment, and subjective cognitive impairment | Three outpatient memory clinics | Healthcare and clinical settings | Recruited referrals with cognitive complaints | Unclear/ not reported | Not reported |
| Neuropsychiatric feature profiles of patients with Lewy body dementia | Department of Neurology and Neurosurgery at Hospital São Paulo, Federal University of São Paulo | Healthcare and clinical settings | Eligible consecutive outpatients invited | Unclear/ not reported | consecutive sampling |
| Capgras syndrome in dementia with Lewy bodies | A dementia speciality practice | Healthcare and clinical settings | Consecutive series of referrals to a specialised centre | Unclear/ not reported | consecutive sampling |
| Autonomic symptoms are predictive of dementia with Lewy bodies | The Department of Neurology of Beijing Tiantan Hospital, Capital Medical University, and the Department of Neurology of Tianjin Huanhu Hospital. | Healthcare and clinical settings | Referred participants were included from the Department of Neurology of Beijing Tiantan Hospital, Capital Medical University, and the Department of Neurology of Tianjin Huanhu Hospital. | Unclear/ not reported | Not reported |
| Patients with dementia with Lewy bodies have more impaired quality of life than patients with Alzheimer disease | Memory clinic | Healthcare and clinical settings | Attending patients prospectively interviewed | clinical engagement | Not reported |
| Study of prevalence of neuropsychiatric symptoms in elderly dementia patients | Tertiary Care Government Hospital of Western Maharashtra | Healthcare and clinical settings | Not reported | Unclear/ not reported | Not reported |
| Differential associations of clinical features with cerebrospinal fluid biomarkers in dementia with Lewy bodies and Alzheimer’s disease | The Behavioural Neurology Section of Hospital São Paulo, Federal University of São Paulo | Healthcare and clinical settings | Consecutive outpatients were recruited from the Behavioral Neurology Section of Hospital São Paulo, Federal University of São Paulo (UNIFESP). | Unclear/ not reported | consecutive sampling |
| Association between amyloid-beta deposition and cortical thickness in dementia with Lewy bodies | Referring clinicians/ local memory clinics | Healthcare and clinical settings | Referred by clinics and clinicians | clinical engagement | not reported |
| Impact of behavioral and psychological symptoms on caregiver burden in patients with dementia with Lewy bodies | Database of neuropsychology clinics in the Department of Psychiatry at Osaka University Hospital from April 2009 to December 2019 | Healthcare and clinical settings | Retrospectively recruited patients at the first diagnosis in their clinic from the database | Unclear/ not reported | not reported |
| Patient affect and caregiver burden in dementia | Outpatient memory clinic of Okayama University Hospital | Healthcare and clinical settings | Recruited consecutive caregivers of individuals who had been referred to the outpatient memory clinic and were diagnosed as having dementia. | Unclear/ not reported | consecutive sampling |
| Examining carer stress in dementia: the role of subtype diagnosis and neuropsychiatric symptoms | Dementia referrals from Old Age Psychiatry and Neurology NHS in the North East of England | Healthcare and clinical settings | Examined case notes to confirm the diagnosis before recruiting their carers into the study | Unclear/ not reported | not reported |
| The effects of behavioral and psychological symptoms on caregiver burden in frontotemporal dementia, Lewy body dementia, and Alzheimer's disease: Clinical experience in China | Memory clinic | Healthcare and clinical settings | unclear/ not reported | Unclear/ not reported | not reported |
| Caregiver burden, sleep quality, depression, and anxiety in dementia caregivers: a comparison of frontotemporal lobar degeneration, dementia with Lewy bodies, and Alzheimer’s disease | Outpatients from the Tianjin Huanhu Hospital | Healthcare and clinical settings | unclear/ not reported | Unclear/ not reported | not reported |
| Factors of dementia caregiver burden differentially contribute to desire to institutionalize | Online dementia caregiver social media groups | Social networks | Study advertisement was posted in 58 dementia caregiver social media groups. | Advertising (dementia caregiver social media groups) | not reported |
| Comparison of caregiver burden between dementia with Lewy bodies and Alzheimer’s disease | A dementia referral centre of Kumamoto University Hospital | Healthcare and clinical settings | Patients selected from a consecutive series of patients who attended a dementia referral centre at the hospital | Unclear/ not reported | consecutive sampling |
| Health related quality of life in individuals with cognitive decline and discrepancies between patients and their proxies | Neurology clinic | Healthcare and clinical settings | Eligible people attending a neurology clinic were invited to voluntarily participate in NeuroDemeNPSia study | clinical engagement | Incidental / convenience |
| Health related quality of life and cognitive decline in older populations: Preliminary results from NeuroDemeNPsia Study | Neurology clinic | Healthcare and clinical settings | Eligible people attending a neurology clinic were invited to voluntarily participate in NeuroDemeNPSia study | clinical engagement | Incidental / convenience |
| Correlates of neuropsychiatric and motor tests with language assessment in patients with Lewy body dementia | The Department of Neurology and Neurosurgery at Hospital São Paulo, Federal University of São Paulo (Unifesp) | Healthcare and clinical settings | All patients with LBD who were followed at the outpatient clinic were assessed from January 2014 to April 2015. | Unclear/ not reported | consecutive sampling |
| Contrasts between patients with Lewy body dementia syndromes and APOE- ε3/ε3 patients with late-onset Alzheimer disease dementia | The Department of Neurology and Neurosurgery at Hospital São Paulo, Federal University of São Paulo-UNIFESP. | Healthcare and clinical settings | Recruited consecutive outpatients with LBD.All patients with LBD who had consultations in the first semester of 2014 were invited. | clinical engagement | consecutive sampling |
| Clinical findings, functional abilities and caregiver distress in the early stage of dementia with Lewy bodies (DLB) and Alzheimer’s disease (AD) | the outpatient memory clinic of the ‘‘Sant’Andrea’’ and ‘‘San Camillo’’ hospitals in Rome | Healthcare and clinical settings | Patients consecutively recruited | Unclear/ not reported | consecutive sampling |
| Tau in dementia with Lewy bodies | Referring clinicians | Healthcare and clinical settings | Individuals with DLB were referred by local memory clinics, neurologists, old age psychiatrists and geriatricians to participate. | clinical engagement | not reported |
| Association of premorbid personality with behavioral and psychological symptoms in dementia with Lewy bodies: Comparison with Alzheimer’s disease patients | Memory clinic at Asahikawa Keisenkai Hospital, Asahikawa, Japan (authors’ hospital) | Healthcare and clinical settings | Unclear/ not reported | unclear/ not reported | not reported |
| Characterization of symptoms and determinants of disease burden in dementia with Lewy bodies: DEvELOP design and baseline results | Amsterdam Dementia Cohort | Healthcare and clinical settings | All patients in the Amsterdam Dementia Cohort were referred to the authors’ memory clinic for analysis of cognitive complaints by their local specialist or general practitioner. | Clinical engagement | not reported |
| Care burden and mental ill health in spouses of people with Parkinson disease dementia and Lewy body dementia | - Memory and movement disorder clinics - "Screen failed" participants list of the INVEST study - Patient databases - UK-based charity and research websites - Join Dementia Research | Healthcare and clinical settings Partner organisations | Referred by clinician Postal questionnaire and invitation etc sent to participant with a prepaid envelope  Advertising (organisation websites and recruitment platforms) | - clinical engagement - Research team invite via telephone, e-mail and/or post - Advertising (organisation websites and recruitment platforms) | not reported |
| Multidimensional care burden in Parkinson-related dementia | - Memory or movement disorder clinics - Those on the “screen-failed” list of the INVEST study - Patient databases - Website adverts on: Parkinson’s UK, LBS and Dementia Research websites. | Healthcare and clinical settings Partner organisations | A postal questionnaire pack, containing an invitation letter, a participant information sheet, a consent form, a survey and a prepaid envelope with the researcher’s postal address, was sent to potential participants who decided whether to take part or not. Referring clinicians used for INVEST study as well as voluntary responses to advertisements. | - Clinical engagement - Research team invite via telephone, e-mail and/or post - Advertising (organisation websites and recruitment platforms) | not reported |
| Caregiver self-efficacy and associated factors among caregivers of patients with dementia with Lewy bodies and caregivers of patients with Alzheimer’s disease | Outpatient clinic of Yagoto Hospital | Healthcare and clinical settings | Invited those attending clinic | clinical engagement | not reported |
| Delusions in patients with dementia with Lewy bodies and the associated factors | A health system’s dementia database- (study was conducted in 3 hospitals in Taiwan but it does not explicitly state whether database participants were recruited from these three hospitals) | Healthcare and clinical settings | unclear | unclear/ not reported | not reported |
| Self-efficacy and social support for psychological well-being of family caregivers of care recipients with dementia with Lewy bodies, Parkinson’s disease dementia, or Alzheimer’s disease | LBDA and several partner dementia-related organisations via mailing lists | Partner organisations | Recruitment information was provided to persons on the organisation's caregiver e-mailing lists. Instructions on how to participate in the survey were provided. Those who agreed to complete the survey were guided to a link to the online survey. | E-mail invite via organisation's mailing list | not reported |
| Factors associated with burden among male caregivers for people with dementia | Data from the T-NDRS database were included. Nine hospitals participated in the T-NDRS project. | Healthcare and clinical settings | unclear/ not reported | unclear/ not reported | not reported |
| Subjective experience of time in dementia with Lewy bodies during COVID-19 lockdown | Outpatient clinics for memory disorders of the neurology department of Padua’s Hospital. | Healthcare and clinical settings | Participants selected among the patients who refer to the outpatient clinics for memory disorders of the neurology department of Padua’s Hospital. Caregivers were chosen from the family members who are mostly engaged in the care of the recruited patients. | unclear/ not reported | not reported |
| Stress and burden among caregivers of patients with Lewy body dementia | LBDA | Partner organisations | The website invited family members who were caring for someone with LBD to complete a survey about their experiences. The survey was advertised with a LBDA homepage announcement and a clickthrough to an internal webpage with more information on the survey. An advertisement was also put in an article in the LBDA newsletter that directed people to the homepage, and e-mail reminders were sent to newsletter subscribers. Caregivers voluntarily clicked on the survey tab. | - Advertising (organisation's website, newsletter and social media) - E-mail invite via organisation's mailing list | not reported |
| A brief psychometric and clinimetric evaluation of self-report burden and mental health measures completed by care partners of people with Parkinson's-related dementia | Reported elsewhere^3^. Sources of recruitment included:   - Memory or movement - The “screen-failed” participant list of the INVEST study, - Patient databases - UK-based charity and research websites | Healthcare and clinical settings Partner organisations | If route 1, were identified in clinic. If route 2, the postal questionnaire, together with an invitation letter, a participant information sheet, a consent form, and a prepaid envelope were posted out to potential participants. Advertisements also used | - Clinical engagement - Research team invite via telephone, e-mail and/or post - Advertising (organisation websites and recruitment platforms) | not reported |
| The frequency and correlates of anxiety in patients with first-time diagnosed mild dementia | Outpatient clinics in geriatric medicine and old age psychiatry for a longitudinal dementia study | Healthcare and clinical settings | Participants were recruited for a longitudinal dementia study. The three neurology outpatient clinics in the area were contacted and agreed to refer new dementia cases to one of the participating centres. | clinical engagement | not reported |
| Caregiver burden in family carers of people with dementia with Lewy bodies and Alzheimer’s disease | DEMVEST participants who were recruited from outpatient clinics; The Norwegian Dementia Register recruited from memory clinics | Healthcare and clinical settings | In the DemVest study, participantswere identified by screening all referrals to outpatient clinics between 2005 and 2008, followed by a further recruitment phase to selectively identify people with DLB and their caregivers up to the end of 2013. For the Norwegian Dementia Register, participation was voluntary, and enrolment required written informed consent. | unclear/not reported | not reported |
| Dementia subtype and living well: results from the Improving the experience of Dementia and Enhancing Active Life (IDEAL) study | - Memory Services and other specialist clinics, - Databases listing PLWD - community mental health teams - GP practices - Social services - voluntary sector groups | - Healthcare and clinical settings - Partner organisations | Recruitment was carried out by staff of the UK research networks. Eligible individuals were contacted by telephone or letter or spoken to in person during clinic appointments to establish whether they were interested in participating. | clinical engagement | not reported |
| The association between specific neuropsychiatric disturbances in people with Alzheimer's disease and dementia with Lewy bodies and carer distress | Recruited from a long-term dementia cohort study. Participants in the study were recruited by screening all referrals to outpatient clinics in western Norway between 2005 and 2007, followed by a recruitment phase to selectively identify people diagnosed with DLB and PDD and their carers to the end of 2013. A diagnosis of dementia was made according to the Diagnostic and Statistical Manu | Healthcare and clinical settings | Participants in the DemVest cohort were recruited by screening all referrals to outpatient clinics in western Norway, followed by a recruitment phase to selectively identify people diagnosed with DLB and PDD and their carers to the end of 2013. | unclear/ not reported | not reported |
| Cognition, hallucination severity and hallucination-specific insight in neurodegenerative disorders and eye disease | Secondary data analysis. Sources of recruitment for people with DLB in the included studies were;  • Archibald et al., 2011 – No reference to DLB ; Makin et al., 2013- No reference to DLB; Mosimann et al., 2008- patients were recruited from the Department of Ophthalmology, from voluntary support groups for the visually impaired and from the Centre for the Health of the Elderly • Taylor et al., 2011- recruited from a local community-dwelling population of patients in the North-East of England • Urwyler et al., 2014-No DLB participants | - Healthcare and clinical settings - Support groups - Unclear/not reported (regarding community dwelling) | not reported | unclear/not reported | not reported |
| Visual hallucinations in eye disease and Lewy body disease | Secondary data analysis. Sources of recruitment for people with DLB in the included studies were;  • Archibald et al., 2011 – No reference to DLB ; Makin et al., 2013- No reference to DLB; Mosimann et al., 2008- patients were recruited from the Department of Ophthalmology, from voluntary support groups for the visually impaired and from the Centre for the Health of the Elderly • Taylor et al., 2011- recruited from a local community-dwelling population of patients in the North-East of England • Urwyler et al., 2014- No DLB participants | - Healthcare and clinical settings - Support groups - Unclear/not reported (regarding community dwelling) | not reported | unclear/not reported | not reported |
| Attitudes toward own aging and cognition among individuals living with and without dementia: findings from the IDEAL programme and the PROTECT study | In the IDEAL cohort:   - Memory Services and other specialist clinics - Databases listing PLWD who are interested in research participation - Community mental health teams - GP practices - social services - Voluntary sector groups   The PROTECT study was advertised through national publicity and through existing cohorts of older adults. | - Healthcare and clinical settings - Partner organisations | In the IDEAL cohort, Recruitment was carried out by staff of the UK research networks. Eligible individuals were contacted by telephone or letter or spoken to in person during clinic appointments to establish whether they were interested in participating. The PROTECT study was advertised through national publicity and through existing cohorts of older adults (Exeter 10,000; Join Dementia Research; and Brains for Dementia Research). | - Clinical engagement - Advertising (via national publicity and through existing cohorts of older adults | Not reported (for the quantitative aspect) |
| The use and costs of paid and unpaid care for people with dementia: Longitudinal findings from the IDEAL cohort | - Memory services and specialist clinics - Database listings of PLWD interested in research participation - Community health teams - General Practitioner practices - Social services - Voluntary sector groups | - Healthcare and clinical settings - Partner organisations | Recruitment was carried out by staff from the UK research network. Eligible participants were contacted by telephone or letter or spoken to in person during clinic appointments. Those expressing interest were sent further information and later visited at home. Non-responses to the initial contact were followed up on one occasion by research network staff to address the possibility that letters and messages could be mislaid due to memory difficulties. | - Clinical engagement - Invite via healthcare research entities and scientific societies | not reported (for the quantitative aspect) |
| Patterns of carer distress over time in mild dementia | Outpatient clinics in Norway | Healthcare and clinical settings | Screened all referrals to outpatients and then a further recruitment phase to selectively identify people diagnosed with DLB and their care partners. | Unclear/ not reported | Not reported |
| Time until nursing home admission in people with mild dementia: comparison of dementia with Lewy bodies and Alzheimer’s dementia | Outpatient clinics in geriatric medicine, neurology and geriatric psychiatry in Western Norway. | Healthcare and clinical settings | All eligible outpatients were invited | clinical engagement | not reported |
| Trajectories and determinants of quality of life in dementia with Lewy bodies and Alzheimer’s disease | Patients from the Dutch Parelsnoer Institute Neurodegenerative Diseases, and the Amsterdam Dementia Cohort. Cohorts are drawn from clinical services. | Healthcare and clinical settings | In both cohorts, participants are referred to clinics and data prospectively collected | clinical engagement | not reported |
| Disease progression in dementia with Lewy bodies: a longitudinal study on clinical symptoms, quality of life and functional impairment. | Patients were referred to Alzheimer Center Amsterdam and those diagnosed with DLB or MCI‐LB, patients were invited to participate in DEvELOP. | Healthcare and clinical settings | Patients were referred to Alzheimer Center Amsterdam and those diagnosed with DLB or MCI‐LB were invited to participate in DEvELOP | clinical engagement | not reported |
| Impact of the COVID-19 pandemic and lockdown on anxiety, depression and nursing burden of caregivers in Alzheimer’s disease, dementia with Lewy bodies and mild cognitive impairment in China: a 1-year follow-up study | Memory clinic | Healthcare and clinical settings | Collected data on consecutive patients and their caregivers | unclear/ not reported | consecutive sampling |
| Updates on somatoform disorders (SFMD) in Parkinson's disease and dementia with Lewy bodies and discussion of phenomenology | Movement disorder and memory outpatient clinics in Central Italy | Healthcare and clinical settings | consecutive referrals to the authors' institutions | unclear/ not reported | consecutive sampling |
| Cohort study on somatoform disorders in Parkinson disease and dementia with Lewy bodies | Movement disorder and memory outpatient clinics in Central Italy. As a comparator prevalence estimate from the general population of the same area referral system, they accessed patients with somatoform disorders from their psychiatry clinic. | Healthcare and clinical settings | Recruited new patients evaluated in the authors’ movement disorder and memory outpatient clinics. | unclear/ not reported | Not reported |
| Prodromal dementia with Lewy bodies: clinical characterization and predictors of progression | The Amsterdam Dementia Cohort. | Healthcare and clinical settings | Patients in the ADC are referred to the Alzheimer Centre Amsterdam. All patients that visit the Alzheimer Center Amsterdam are asked to consent to the use of their clinical data for scientific purpose. | clinical engagement | Not reported |
| Personality traits distinguishing dementia with Lewy bodies from Alzheimer disease | Clinical records (individuals already participating in a longitudinal study). Unclear where these participants were recruited from | Unclear/ not reported | Examined clinical records of participants in a longitudinal study - unclear how participants were initially recruited | unclear/ not reported | convenience |
| Frequency of suicidal ideation and associated clinical features in Lewy body dementia | Recruited all database participants meeting eligibility. The cohort represents patients at a single tertiary care movement disorders centre. | Healthcare and clinical settings | Screening database for eligible participants- unclear how participants were initially recruited | unclear/ not reported | Not reported |
| A comparison of caregiver burden for different types of dementia: An 18-month retrospective cohort study | The Dementia Centre of Changhua Christian Hospital | Healthcare and clinical settings | Recruitment occurred through the hospital's care team. | clinical engagement | Not reported |
| Memantine for patients with Parkinson’s disease dementia or dementia with Lewy bodies: a randomised, double-blind, placebo-controlled trial | Specialist centres globally | Healthcare and clinical settings | Recruited directly from specialist centres | unclear/ not reported | Not reported |
| Long-term safety and efficacy of donepezil in patients with dementia with Lewy bodies: Results from a 52-week, open-label, multicenter extension study | Participants who completed preceding phase 2 study | Continuation from preceding study | Phase 2 participants were recruited for this extension study | unclear/ not reported | Not reported |
| Music playlists for people with dementia: Trialling a guide for caregivers | - Aged care facilities - Support groups for home-based carers | Aged care facilities Support groups | Aged care facilities and support groups approached by phone and e-mail. Where interest was shown, the facilities and groups were visited, a presentation given, and information sheets distributed. | - Research team invite via telephone, e-mail and/or post - Researcher presentation | Not reported |
| Bilateral nucleus basalis of Meynert deep brain stimulation for dementia with Lewy bodies: A randomised clinical trial | Two neurological referral centres in the UK | Healthcare and clinical settings | Unclear/ not reported | Unclear/ not reported | not reported |
| Adjunct zonisamide to levodopa for DLB parkinsonism: A randomized double-blind phase 2 study | Medical institutions | Healthcare and clinical settings | Unclear/ not reported | unclear/ not reported | Not reported |
| Donepezil for dementia with Lewy Bodies: A randomized, placebo-controlled trial | Psychiatric or neurological speciality centres throughout Japan | Healthcare and clinical settings | Unclear/ not reported | Unclear/ not reported | not reported |
| Long-term donepezil use for dementia with Lewy bodies: Results from an open-label extension of Phase III trial | Psychiatric or neurologic specialty centres throughout Japan | Healthcare and clinical settings | Unclear/ not reported | Unclear/ not reported | not reported |
| Efficacy, safety, and tolerability of armodafinil therapy for hypersomnia associated with dementia with Lewy bodies: a pilot study | The behavioural neurology outpatient clinic and Alzheimer’s Disease Research Centre program at Mayo Clinic, Rochester, MN, USA | Healthcare and clinical settings | Unclear/ not reported | Unclear/ not reported | not reported |
| Efficacy of adjunctive therapy with zonisamide versus increased dose of levodopa for motor symptoms in patients with dementia with Lewy bodies: the randomized, controlled, non-inferiority DUEL study | Centres in Japan | Healthcare and clinical settings | Investigators explained the study procedures to suitable patients and their caregivers or alternative representatives, who provided written informed consent once they understood the study procedures and agreed to participate. | clinical engagement | not reported |
| A comparison of the efficacy of donepezil in Parkinson’s disease with dementia and dementia with Lewy bodies | Old Age Psychiatry and Neurology Departments | Healthcare and clinical settings | Unclear/ not reported | Unclear/ not reported | not reported |
| Goal-orientated cognitive rehabilitation for dementias associated with Parkinson's disease- a pilot randomised controlled trial | Movement Disorder clinics and Memory clinics in Betsi Cadwaladr University Health Board, North Wales, UK. | Healthcare and clinical settings | Potential participants were approached consecutively and invited to an initial screening interview with the researcher | clinical engagement | consecutive sampling |
| Quality of life and the effect of memantine in dementia with Lewy bodies and Parkinson’s disease dementia | Secondary analysis of RCT data. In the RCT, participants were recruited from four psychiatric and neurological outpatient clinics in Norway, Sweden, and the UK | Healthcare and clinical settings | not reported | unclear/ not reported | not reported |
| Improvement in delusions and hallucinations in patients with dementia with Lewy bodies upon administration of yokukansan, a traditional Japanese medicine | Hospitals in Japan | Healthcare and clinical settings | Not reported | Not reported (expanded case series) | Not reported (case study) |
| Preclinical polymodal hallucinations for 13 years before dementia with Lewy bodies | Not applicable | Not applicable | Not applicable | Not applicable | Not applicable |
| Donepezil for treatment of dementia with Lewy bodies: A case series of nine patients | The Memory Disability Clinic of the Queen Elizabeth II Health Sciences Centre (QEII HSC). | Healthcare and clinical settings | The clinic has a comprehensive research program, so that all dementia patients are offered enrolment in observational or interventional studies. | Contacted via consent-to-contact database | consecutive |
| Lumateperone for treatment of psychotic symptoms in Lewy body | Not applicable | Not applicable | Not applicable | Not applicable | Not applicable |
| Cognitive effects of quetiapine in a patient with dementia with Lewy bodies | Not applicable | Not applicable | Not applicable | Not applicable | Not applicable |
| Open label trial to evaluate the efficacy and safety of Yokukansan, a traditional Asian medicine, in dementia with Lewy bodies | Hospitals in Japan | Healthcare and clinical settings | not reported | not reported | Not reported (prospective case series) |
| Effectiveness of ramelteon for treatment of visual hallucinations in dementia with Lewy bodies a report of 4 cases | Not applicable | Not applicable | Not applicable | Not applicable | Not applicable |
| Administration of zonisamide in three cases of dementia with Lewy bodies | Not applicable | Not applicable | Not applicable | Not applicable | Not applicable |
| Raising awareness and mutual support: using the internet | Not applicable | Not applicable | Not applicable | Not applicable | Not applicable |
| Parkinson’s/ Lewy body dementia: a carer’s perspective | Not applicable | Not applicable | Not applicable | Not applicable | Not applicable |
| Practical treatment of Lewy body disease in the clinic: Patient and physician perspectives | Not applicable | Not applicable | Not applicable | Not applicable | Not applicable |
| My Friend Lewy | Not applicable | Not applicable | Not applicable | Not applicable | Not applicable |
| Improving the diagnosis and management of Lewy body dementia: The DIAMOND-Lewy research programme including pilot cluster RCT | Work package 5: clinics | Healthcare and clinical settings | Work package 5: patients and carers for interview were identified by participating clinicians and the local research nurse. | Clinical engagement | convenience (all available participants were recruited) |
| Presence phenomena in parkinsonian disorders: phenomenology and neuropsychological correlates | The Neuropsychology Department of the National Hospital for Neurology and Neurosurgery, Queen’s Square, London. | Healthcare and clinical settings | Eligible participants identified during routine screening were invited | clinical engagement | Not reported |
| Support and information needs following a diagnosis of dementia with Lewy bodies | Lewy body society website | Partner organisations | Respondents accessed the survey by a self-initiated search of the site where information about the survey was posted on and linked from the home page | Advertising (organisation's website) | Not reported |
| “I felt like I had been put on the shelf and forgotten about” – lasting lessons about the impact of COVID-19 on people affected by rarer dementias | Rare Dementia Support | Partner organisations | Survey distributed to mailing list | E-mail invite via organisation's mailing list | Not reported |
| Conversations in dementia with Lewy bodies: resources and barriers in communication | Clinical services | Healthcare and clinical settings | Participants first received information on the project by one of their healthcare providers. | - clinical engagement - Research team invite via telephone, email and/or post. | Not reported |
| The feasibility and acceptability of a psychosocial intervention to support people with dementia with Lewy bodies and family care partners | Specialist Lewy body disease clinic | Healthcare and clinical settings | Unclear/ not reported | Unclear/ not reported | Not reported |
| Parkinson’s-adapted cognitive stimulation therapy: a pilot randomized controlled clinical trial | - Multiple centres in the UK with established memory or movement disorder clinics. - UK-based charity websites - Join Dementia Research. - Support groups | - Healthcare and clinical settings - Partner organisations - Support groups | Eligible dyads were identified at recruiting centres by trial collaborators and research nurses and therapists; User-friendly information brochures were available at memory and movement disorder clinics and support groups, and information talks were given at Parkinson’s UK community group | - clinical engagement - Advertising | Not reported |
| Caring for people with dementia with Lewy bodies and Parkinson’s dementia in UK care homes – a mixed methods study | Care homes for older people in England and Scotland | Aged care facilities | The survey was sent to all 144 care homes for older people in the two areas identified from social care listings. Homes were initially contacted by both e-mail and letter, and following this, non-responders were contacted by telephone to ask if they wished to participate. As part of the survey, managers were asked whether they wanted their home to participate in the interview phase of the study. | Research team invite via telephone, e-mail and/or post | Not reported |
| Parkinson’s-adapted cognitive stimulation therapy: feasibility and acceptability in Lewy body spectrum disorders | Four UK centres with established memory or movement disorder clinics | Healthcare and clinical settings | The study was advertised on UK-based charity websites and through Join Dementia Research. User-friendly information brochures were available at memory and movement disorder clinics and support groups, and information talks were given at Parkinson’s UK community groups | - Advertising (organisations' websites, recruitment platforms, brochures) - Researcher presentation (to community groups) | Unclear Purposive sample selected for the qualitative aspect |
| Learning to PERSEVERE: a pilot study of peer mentor support and caregiver education in Lewy body dementia | Participants for the focus group were recruited nationally via stakeholder organisations. For the intervention, most mentee and mentor participants were recruited through Parkinson’s Foundation and the LBDA. Other sources include regional PD and LBD support groups, RUMC neurologists and prior PD mentoring participants from the PD intervention study. | - Partner organisations - Support groups - Healthcare and clinical settings - Continuation from preceding study | To recruit mentors and mentees, Parkinson’s Foundation and LBDA disseminated a recruitment survey link via e-mail and social media. Recruitment flyers were distributed to regional PD and LBD support groups, RUMC neurologists to share with eligible caregivers, and prior PD mentoring participants who met mentor eligibility criteria. Following clinic-based referral or online survey completion, a study coordinator called potential participants, confirmed eligibility for the mentor/mentee role, and completed consent process. | - Advertising (Flyers in clinics, for previous study participants, and in support groups; organisations' social media postings) - Clinical engagement - E-mail invite via organisation's mailing list - Unclear how prior PD mentoring participants were recruited | Not reported |
| A pilot study on the use of dolls for people with dementia | Two Elderly Mentally Ill homes | Aged care facilities | Before the introduction of the dolls, an RMN visited each home and discussed the use of dolls with the manager and the staff and left an article on the approach at the home. All staff in the homes were invited to participate. | - Researcher presentation (to care home staff) | Not reported |
| Tailoring and evaluating the web-based ‘Partner in Balance’ intervention for family caregivers of persons with young-onset dementia | 1. Network of Alzheimer Centre Limburg/MUMC+ 2. Dutch Young-onset Dementia Knowledge Centre  3. Social media | - Healthcare and clinical settings - Partner organisations  Social networks | - Spreading info at the centre - Providing info at HCP meetings - Advertising on social media - Sharing info at peer-support meetings Interested individual contacted first author | - Unclear - Advertising (social media) - Researcher presentation (support groups and HCP meetings) | Not reported |
| Development and validation of the Lewy body disease caregiver activities scale | A cognitive disorders centre in a metropolitan hospital in a large midwestern city. For the face validity interviews, six participants from an earlier step in the study participated. | Healthcare and clinical settings | Potential participants were informed of the study during their visit to the centre or through a letter signed by the Medical Director, along with a study information sheet mailed to their home. Caregivers were contacted by telephone a week after information was mailed. | - Clinical engagement - Research team invite via telephone, e-mail and/or post | Convenience |
| Development of assessment toolkits for improving the diagnosis of the Lewy body dementias: feasibility study within the DIAMOND Lewy study | Clinical services within the UK | Healthcare and clinical settings | Patients were recruited by individual clinicians | Clinical engagement | Not reported |
| Introduction of a management toolkit for Lewy body dementia: a pilot cluster-randomized trial | Memory clinics, movement disorder clinics, and general secondary care clinics/services for older people | Healthcare and clinical settings | The identified services recruited the participants. | Clinical engagement | Not reported |
| **Abbreviations:** Dementia with Lewy bodies (DLB); Parkinson’s disease dementia (PDD); Lewy body dementia (LBD); Lewy Body Dementia Association (LBDA);The DEmEntia with LEwy bOdies Project (DEvELOP); INdiVidualised cognitivE Stimulation Therapy study (INVEST); The Improving the experience of Dementia and Enhancing Active Life (IDEAL); Platform for Research Online to investigate Genetics and Cognition in Aging (PROTECT); The dementia study of Western Norway (DemVest); Health care professional (HCP); Principal investigator (PI). | | | | | |

**Appendix 10:** Personal (patient) and public involvement

A. Personal (patient) and public involvement in the included sources

| **Personal and Public Involvement in studies** | **Yes/No** |
| --- | --- |
| Research priorities of caregivers and individuals with dementia with Lewy bodies: An interview study | Yes |
| Informal caregiver experiences at the end-of-life of individuals living with dementia with Lewy bodies: an interview study | Yes |
| Living with dementia with Lewy bodies: an interpretative phenomenological analysis | No |
| End-of-life experiences in dementia with Lewy bodies: qualitative interviews with former caregivers | Yes |
| Caregiver-reported barriers to quality end-of-life care in dementia with Lewy bodies: a qualitative analysis | Yes |
| Clinical care and unmet needs of individuals with dementia with Lewy bodies and caregivers: an interview study | Yes |
| ‘It's just incredible the difference it has made': family carers' experiences of a specialist Lewy body dementia admiral nurse service | No |
| The unique experience of spouses in early-onset dementia | No |
| Exploring the impact of caring for an individual with neurogenic orthostatic hypotension: A qualitative study | No |
| Needs and concerns of Lewy body disease family caregivers: A qualitative study | No |
| Understanding the nature and impact of cognitive fluctuations and sleep disturbances in dementia with Lewy bodies: a qualitative caregiver study | Yes |
| A qualitative study of female caregiving spouses’ experiences of intimate relationships as cognition declines in Parkinson’s disease | Yes |
| Problems faced by people living at home with dementia and incontinence: causes, consequences and potential solutions | No |
| Perceived benefits of using nonpharmacological interventions in older adults with Alzheimer’s disease or dementia with Lewy bodies | No |
| Difficulties and associated coping methods regarding visual hallucinations caused by dementia with Lewy bodies | No |
| Carers to people with Lewy body dementia and Alzheimer's disease: Experiences and coping strategies. | No |
| Key components of post-diagnostic support for people with dementia and their carers: A qualitative study | Yes |
| Goal setting for cognitive rehabilitation in mild to moderate Parkinson’s disease dementia and dementia with Lewy bodies | No |
| Measuring disability in patients with neurodegenerative disease using the ‘Yesterday Interview’ | No |
| Profiling conversation in Parkinson’s disease with cognitive impairment | No |
| The human need for equilibrium: qualitative study on the ingenuity, technical competency, and changing strategies of people with dementia seeking health information | Yes |
| Applying an analytical process to longitudinal narrative interviews with couples living and dying with Lewy body dementia | Yes |
| Problematising carer identification: a narrative study with older partner's providing end-of-life care | Yes |
| Pacemaker implants and their influence on the daily life of patients with dementia with Lewy bodies: A qualitative case study | No |
| Using care navigation to address caregiver burden in dementia: A qualitative case study analysis | No |
| Cause of death and end-of-life experiences in individuals with dementia with Lewy bodies | Yes |
| COVID-19: association between increase of behavioral and psychological symptoms of dementia during lockdown and caregivers’ poor mental health | No |
| Questionnaire survey of satisfaction with medication for five symptom domains of dementia with Lewy bodies among patients, their caregivers, and their attending physicians | No |
| Lewy body dementia: The caregiver experience of clinical care | No |
| Research priorities of individuals and caregivers with Lewy body dementia a web-based survey | Yes |
| Treatment needs of dementia with Lewy bodies according to patients, caregivers, and physicians: a cross-sectional, observational questionnaire-based study in Japan | No |
| The impact of COVID-19 quarantine on patients with dementia and family caregivers: A nation-wide survey | No |
| The importance of educating the Lewy body dementia community on risks and benefits of lumbar punctures in LBD biomarker research | No |
| Behavioral and psychological effects of coronavirus disease-19 quarantine in patients with dementia | No |
| Video research visits for atypical parkinsonian syndromes among Fox Trial Finder participants | No |
| The insula, a grey matter of tastes: A volumetric MRI study in dementia with Lewy bodies | No |
| Pain in extrapyramidal neurodegenerative diseases | No |
| Characteristics of eating and swallowing problems in patients who have dementia with Lewy bodies | No |
| Comparison of the caregiving experience of grief, burden, and quality of life in dementia with Lewy bodies, Alzheimer’s disease, and Parkinson’s disease dementia | Yes |
| Lewy body dementia: caregiver burden and unmet needs | No |
| Pre-loss grief in caregivers of older adults with dementia with Lewy bodies | No |
| Differences in the experience of caregiving between spouse and adult child caregivers in dementia with Lewy bodies | No |
| Dementia patients caregivers quality of life: the PIXEL study | No |
| The role of sexual disinhibition to predict caregiver burden and desire to institutionalize among family dementia caregivers | No |
| Neuropsychological study of amyotrophic lateral sclerosis and parkinsonism-dementia complex in Kii peninsula, Japan | No |
| Clinical features of delusional jealousy in elderly patients with dementia | No |
| Costs of dementia with Lewy bodies: a Chinese multicenter cross-sectional study | No |
| Depression in dementia with Lewy bodies: a comparison with Alzheimer’s disease | No |
| Patients with Lewy body dementia use more resources than those with Alzheimer’s disease | No |
| Comparison of QOL between patients with different degenerative dementias, focusing especially on positive and negative affect | No |
| Pain in patients with different dementia subtypes, mild cognitive impairment, and subjective cognitive impairment | No |
| Neuropsychiatric feature profiles of patients with Lewy body dementia | No |
| Capgras syndrome in dementia with Lewy bodies | No |
| Autonomic symptoms are predictive of dementia with Lewy bodies | No |
| Patients with dementia with Lewy bodies have more impaired quality of life than patients with Alzheimer disease | No |
| Study of prevalence of neuropsychiatric symptoms in elderly dementia patients | No |
| Differential associations of clinical features with cerebrospinal fluid biomarkers in dementia with Lewy bodies and Alzheimer’s disease | No |
| Association between amyloid-beta deposition and cortical thickness in dementia with Lewy bodies | No |
| Impact of behavioral and psychological symptoms on caregiver burden in patients with dementia with Lewy bodies | No |
| Patient affect and caregiver burden in dementia | No |
| Examining carer stress in dementia: the role of subtype diagnosis and neuropsychiatric symptoms | No |
| The effects of behavioral and psychological symptoms on caregiver burden in frontotemporal dementia, Lewy body dementia, and Alzheimer's disease: clinical experience in China | No |
| Caregiver burden, sleep quality, depression, and anxiety in dementia caregivers: a comparison of frontotemporal lobar degeneration, dementia with Lewy bodies, and Alzheimer’s disease | No |
| Factors of dementia caregiver burden differentially contribute to desire to institutionalize | No |
| Comparison of caregiver burden between dementia with Lewy bodies and Alzheimer’s disease | No |
| Health related quality of life in individuals with cognitive decline and discrepancies between patients and their proxies | No |
| Health related quality of life and cognitive decline in older populations: preliminary results from NeuroDemeNPsia Study | No |
| Correlates of neuropsychiatric and motor tests with language assessment in patients with Lewy body dementia | No |
| Contrasts between patients with Lewy body dementia syndromes and APOE- ε3/ε3 patients with late-onset Alzheimer disease dementia | No |
| Clinical findings, functional abilities and caregiver distress in the early stage of dementia with Lewy bodies (DLB) and Alzheimer’s disease (AD) | No |
| Tau in dementia with Lewy bodies | No |
| Association of premorbid personality with behavioral and psychological symptoms in dementia with Lewy bodies: comparison with Alzheimer’s disease patients | No |
| Characterization of symptoms and determinants of disease burden in dementia with Lewy bodies: DEvELOP design and baseline results | No |
| Care burden and mental ill health in spouses of people with Parkinson disease dementia and Lewy body dementia | Yes |
| Multidimensional care burden in Parkinson-related dementia | Yes |
| Caregiver self-efficacy and associated factors among caregivers of patients with dementia with Lewy bodies and caregivers of patients with Alzheimer’s disease | No |
| Delusions in patients with dementia with Lewy bodies and the associated factors | No |
| Self-efficacy and social support for psychological well-being of family caregivers of care recipients with dementia with Lewy bodies, Parkinson’s disease dementia, or Alzheimer’s disease | No |
| Factors associated with burden among male caregivers for people with dementia | No |
| Subjective experience of time in dementia with Lewy bodies during COVID-19 lockdown | No |
| Stress and burden among caregivers of patients with Lewy body dementia | No |
| A brief psychometric and clinimetric evaluation of self-report burden and mental health measures completed by care partners of people with Parkinson's-related dementia | Yes |
| The frequency and correlates of anxiety in patients with first-time diagnosed mild dementia | No |
| Caregiver burden in family carers of people with dementia with Lewy bodies and Alzheimer’s disease | No |
| Dementia subtype and living well: Results from the Improving the experience of Dementia and Enhancing Active Life (IDEAL) study | Yes |
| The association between specific neuropsychiatric disturbances in people with Alzheimer's disease and dementia with Lewy bodies and carer distress | No |
| Cognition, hallucination severity and hallucination-specific insight in neurodegenerative disorders and eye disease | No |
| Visual hallucinations in eye disease and Lewy body disease | No |
| Attitudes toward own aging and cognition among individuals living with and without dementia: findings from the IDEAL programme and the PROTECT study | No |
| The use and costs of paid and unpaid care for people with dementia: Longitudinal findings from the IDEAL cohort | Yes |
| Patterns of carer distress over time in mild dementia | No |
| Time until nursing home admission in people with mild dementia: comparison of dementia with Lewy bodies and Alzheimer’s dementia | No |
| Trajectories and determinants of quality of life in dementia with Lewy bodies and Alzheimer’s disease | No |
| Disease progression in dementia with Lewy bodies: A longitudinal study on clinical symptoms, quality of life and functional impairment. | No |
| Impact of the COVID-19 pandemic and lockdown on anxiety, depression and nursing burden of caregivers in Alzheimer’s disease, dementia with Lewy bodies and mild cognitive impairment in China: A 1-year follow-up study | No |
| Updates on somatoform disorders (SFMD) in Parkinson's disease and dementia with Lewy bodies and discussion of phenomenology | No |
| Cohort study on somatoform disorders in Parkinson disease and dementia with Lewy bodies | No |
| Prodromal dementia with Lewy bodies: clinical characterization and predictors of progression | No |
| Personality traits distinguishing dementia with Lewy bodies from Alzheimer disease | No |
| Frequency of suicidal ideation and associated clinical features in Lewy body dementia | No |
| A comparison of caregiver burden for different types of dementia: an 18-month retrospective cohort study | No |
| Donepezil for treatment of dementia with Lewy bodies: a case series of nine patients | No |
| Memantine for patients with Parkinson’s disease dementia or dementia with Lewy bodies: a randomised, double-blind, placebo-controlled trial | No |
| Long-term safety and efficacy of donepezil in patients with dementia with Lewy bodies: results from a 52-week, open-label, multicenter extension study | No |
| Music playlists for people with dementia: Trialing a guide for caregivers | Yes |
| Bilateral nucleus basalis of Meynert deep brain stimulation for dementia with Lewy bodies: a randomised clinical trial | No |
| Adjunct zonisamide to levodopa for DLB parkinsonism: a randomized double-blind phase 2 study | No |
| Donepezil for dementia with Lewy bodies: a randomized, placebo-controlled trial | No |
| Long-term donepezil use for dementia with Lewy bodies: Results from an open-label extension of phase III trial | No |
| Efficacy, safety, and tolerability of armodafinil therapy for hypersomnia associated with dementia with Lewy bodies: A pilot study | No |
| Efficacy of adjunctive therapy with zonisamide versus increased dose of levodopa for motor symptoms in patients with dementia with Lewy bodies: the randomized, controlled, non-inferiority DUEL study | No |
| A comparison of the efficacy of donepezil in Parkinson’s disease with Dementia and Dementia with Lewy bodies | No |
| Goal-orientated cognitive rehabilitation for dementias associated with Parkinson's disease- a pilot randomised controlled trial | No |
| Quality of life and the effect of memantine in dementia with Lewy bodies and Parkinson’s disease dementia | No |
| Lumateperone for treatment of psychotic symptoms in Lewy body | No |
| Cognitive effects of quetiapine in a patient with dementia with Lewy bodies | No |
| Open label trial to evaluate the efficacy and safety of Yokukansan, a traditional Asian medicine, in dementia with Lewy bodies | No |
| Improvement in delusions and hallucinations in patients with dementia with Lewy bodies upon administration of yokukansan, a traditional Japanese medicine | No |
| Effectiveness of ramelteon for treatment of visual hallucinations in dementia with Lewy bodies: A report of 4 cases | No |
| Administration of zonisamide in three cases of dementia with Lewy bodies | No |
| Improving the diagnosis and management of Lewy body dementia: the DIAMOND-Lewy research programme including pilot cluster RCT | Yes |
| Presence phenomena in parkinsonian disorders: Phenomenology and neuropsychological correlates | No |
| Preclinical polymodal hallucinations for 13 years before dementia with Lewy bodies | No |
| Support and information needs following a diagnosis of dementia with Lewy bodies | Yes |
| “I felt like I had been put on the shelf and forgotten about” – lasting lessons about the impact of COVID-19 on people affected by rarer dementias | Yes |
| Conversations in dementia with Lewy bodies: Resources and barriers in communication | No |
| The feasibility and acceptability of a psychosocial intervention to support people with dementia with Lewy bodies and family care partners | Yes |
| Parkinson’s-adapted cognitive stimulation therapy: a pilot randomized controlled clinical trial | Yes |
| Caring for people with dementia with Lewy bodies and Parkinson’s dementia in UK care homes – a mixed methods study | Yes |
| Parkinson’s-adapted cognitive stimulation therapy: feasibility and acceptability in Lewy body spectrum disorders | Yes |
| Learning to PERSEVERE: A pilot study of peer mentor support and caregiver education in Lewy body dementia | Yes |
| A pilot study on the use of dolls for people with dementia | No |
| Tailoring and evaluating the web-based ‘Partner in Balance’ intervention for family caregivers of persons with young-onset dementia | Yes |
| Development and validation of the Lewy body disease caregiver activities scale | No |
| Introduction of a management toolkit for Lewy body dementia: A pilot cluster-randomized trial | Yes |
| Development of assessment toolkits for improving the diagnosis of the Lewy body dementias: Feasibility study within the DIAMOND Lewy study | Yes |
| Raising awareness and mutual support: Using the internet |  |
| Parkinson’s/ Lewy body dementia: A carer’s perspective |  |
| Practical treatment of Lewy body disease in the clinic: Patient and physician perspectives |  |
| My friend Lewy |  |
| Total number of studies including PPI | 31 |

**B.** Trend in personal (patient) and public Involvement in the included sources

**Fig.** Trend in personal (patient) and public Involvement in the included sources. The search was completed in September 2023. As a result, the 2023 and 2024 columns do not represent all studies published during this period.

**Appendix 11:** The frequency of use of reported standardised measures, organised by their assessed domains

This table presents the frequency of use of standardised instruments across all sources (n=140), alongside the domains they assessed. The ‘Frequency of use’ column indicates the total number of times each instrument was employed. The ‘Standardised instrument and the domain(s) assessed’ column categorises these instruments based on the specific domains they addressed.

A single instrument may appear multiple times in the same frequency row. This does not mean it was used multiple times for the same domain but rather that it was used to assess different domains. For example, the QOL-D was used twice in total, once for quality of life and once for positive affect.

| **Frequency of use** | **Standardised instruments and the domain(s) assessed** |
| --- | --- |
| 1 | - **Which aspects of conversation care partners perceive to be problematic, and strategies used to deal with conversational challenges:** CAPPCI - **Resource use:** RUD-Lite - **Pain:** BPI - **Changes in personality, interests and drive:** BDS - **Changes in mood and behaviour in response to music therapy:** MiDAS - **Information on paid care and unpaid care activities:** CSRI - **Sexual symptoms and sexual dysfunction:** SCOPA-Aut - **Satisfaction with life:** SwLS - **Well-being:** WHO-5 - **Feelings towards and confidence caring for individuals with dementia:** DAS - **The extent that caregivers believe they control their own lives:** PMS - **Self-perceived isolation, relational and social connectedness:** UCLA-LS;R-UCLA-LS - **Pre-loss grief:** PG-12 - **Care partner self-efficacy: FCP-SEMD Scale** - **Stress:** PSS - **Exploring activity and subjective enjoyment:** ‘Yesterday Interview’ - **Empathy:** IRI - **Quality of Life:** PDQ‐8; LASA; ADRQL; DEMQOL; SF-8 - **Steps an informal caregiver has taken in considering institutionalising their care recipient:** DIS - **Subjective sleep quality:** ISI - **Social support (number of individuals available to provide social support and degree of satisfaction with support provided):** SSQ - **subjective experience of the passage of time:** STQ - **Attitudes towards own ageing:** ATOA - **Premorbid personality:** NEO-FFI - **Overall health:** EQ-5D-3L - **Care partner burden:** 11-item ZBI |
| 2 | - **Social support:** MOS-SS - **Care partner's perceived change in their well-being:** PCI - **Quality of life:** PDQ-39; WHOQOL-BREF; EQ-5D-5L; QOL-D - **Patient affect:** QOL-D - **Presence or absence of suicidal ideation in people with DLB:** BDI-II - **Caregiver anxiety and/or depression:** BDI-II; GAD-7; GDS-15 - **Subjective sleep quality:** PSQI - **Self-efficacy:** GSES - **Goals, motivations and ratings of goal attainment:** BGSI - **Phenomenology of visual hallucinations and their perceived severity and distress:** NEVHI - **HRQoL:** SF-12-v2; EQ-5D-3L - **Distress related to perceptions of bodily dysfunction:** SCL-90-R |
| 3 | - **Caregiver grief:** MM-CGI-SF - **Caregiver depression:** PHQ-9 - **Caregiver burden:** J-ZBI-8 - **Self-efficacy:** RSCSE - **Relationship satisfaction:** RSS; DRS; FCR - **Mental and physical health:** SF-12 - **Resilience:** BRS |
| 4 | - **Caregiver depression:** PHQ-2 - **Psychological well-being:** C-PWBS |
| 5 | - **Quality of life:** EQ-5D-3L |
| 6 | - **Anxiety and depression:** HADS |
| 7 | - **Care partner burden:** 12 item ZBI |
| 9 | - **Quality of life:** QoL-AD |
| 10 | - **Care partner/relative’s stress:** Rel.SS |
| 13 | - **Caregiver distress:** NPI-D |
| 34 | - **Care partner burden:** 22 item ZBI |
| **Abbreviations:** Conversation Analysis Profile for People with Cognitive Impairment (CAPPCI); Resource Utilization in Dementia - Lite Version (RUD-Lite); Brief Pain Questionnaire (BPI); Blessed Dementia Scale (BDS); The Music in Dementia Assessment Scale (MiDAS); Client Services Receipt Inventory (CSRI); Scales for Outcomes in PD-Autonomic (SCOPA-Aut); Satisfaction with Life Scale (SwLS); The WHO-5 Well-being Index (WHO-5); Dementia Attitudes Scale (DAS); Pearlin Mastery Scale (PMS); UCLA Loneliness Scale (UCLA-LS); Revised UCLA Loneliness Scale (R-UCLA-LS); The Prolonged Grief-12 (PG-12); Family Care Partner Self-Efficacy for Managing Dementia Scale (**FCP-SEMD Scale);** Geriatric Depression Scale (GDS-15); Perceived Stress Scale (PSS); Condensed version of the Psychological Well-Being Scale (C-PWBS); The Interpersonal Reactivity Index (IRI); Parkinson's Disease Questionnaire―8 (PDQ‐8); Linear Analogue Self-Assessment Scale (LASA); The Alzheimer Disease-related Quality of Life (ADRQL); Dementia Quality of Life Measure (DEMQOL); Euroqol Questionnaire‐short version (ED5D3L); The Desire to Institutionalize Scale (DIS); Insomnia Severity Index (ISI); Social Support Questionnaire (SSQ); Subjective Time Questionnaire (STQ); The Attitude Toward Own Aging questionnaire (ATOA); NEO Five Factor Inventory (NEO-FFI); The Medical Outcome Study Social Support (MOS-SS); Perceived Change Index (PCI); 39-item Parkinson's Disease Questionnaire (PDQ-39); Beck Depression Inventory-II (BDI-II); Quality of life questionnaire for dementia (QOL-D); General Anxiety Disorder-7 (GAD-7); The Pittsburgh Sleep Quality Index(PSQI); Short-Form-8 (SF-8); World Health Organization Quality of Life-Brief Version (WHOQOL-BREF); Generalized Self-Efficacy Scale (GSES); The Bangor Goal Setting Interview (BGSI); Euro-Qol 5 dimensions 5-level (EQ-5D-5L); The North-East Visual Hallucination Interview (NEVHI); Short Form Health Survey, version 2 (SF-12-v2); Symptom Checklist 90R (SCL-90-R); The Marwit-Meuser Caregiver Grief Inventory Short Form (MM-CGI-SF); Patient Health Questionnaire-9 (PHQ-9); 8-item short version of the Japanese Zarit Burden Interview (J-ZBI_8); Revised Scale for Caregiving Self-Efficacy (RSCSE); The Relationship Satisfaction Scale (RSS); The 12-item short-form health survey (SF-12); The Dyadic Relationship Scale (DRS); The Family Caregiving Role Scale (FCR); The Brief Resilience Scale (BRS); Patient Health Questionnaire-2 (PHQ-2); Hospital Anxiety and Depression Scale (HADS); 12-item Zarit Burden Interview (ZBI-12); EuroQol- 5 Dimension (EQ-5D); Quality of Life in Alzheimer’s Disease (QoL-AD); Relative’s Stress Scale (Rel.SS); Neuropsychiatric Inventory Caregiver Distress Scale (NPI-D); The Zarit Burden Interview (ZBI) | |

**Appendix 12**: Table: Overview of topic categories, aims of sources and data collection methods

| **Categories of topics for which perspectives were sought (number of sources)** | | | | |
| --- | --- | --- | --- | --- |
| **Aims** | **Method of data collection** | | | **Citation** |
| **1. Perspectives related to research (n= 3) –** Captures patient and CP research priorities and perspectives related to the use of lumbar punctures in research. | | | | |
| To identify the research priorities of individuals with DLB and CPs. | Qualitative study involving telephone interviews with individuals living with DLB and CPs, querying research needs across different categories and asking participants to select their top priorities. | | | [1] |
| To identify the research priorities of people affected by LBD. | Quantitative study involving a bespoke web-based survey of people with LBD and current and former CPs, querying research priorities through forced ranking and exploration of the burden of LBD symptoms. | | | [2] |
| To attain a better understanding of the knowledge, concerns and opinions of people with LBD and their CPs on the risks and benefits of lumbar punctures in LBD biomarker research. | Quantitative study involving a bespoke web-based survey of people with LBD and their CPs, exploring their knowledge, attitudes, concerns and opinions on research lumbar punctures. | | | [3] |
| **2. End-of-life experiences (n=4) -** Captures CP experiences and reflections related to the end-of-life period. | | | | |
| One study, reported across two sources, investigated the end-of-life experiences of informal CPs of individuals with DLB who died within the past 5 years. | Qualitative study involving telephone interviews with CPs, using a bespoke semi-structured guide to query experiences during the end-of-life period. | | | [4,5] |
| To investigate barriers to quality end-of-life care in DLB. | Qualitative study involving telephone interviews with CPs and family members of individuals who died with DLB in the last 5 years. The interviews used a semi-structured questionnaire to identify common barriers to quality end-of-life care. | | | [6] |
| Study investigated the natural history, cause of death and end-of-life experiences of individuals with DLB. | Quantitative study involving a bespoke web survey for CPs, family, and friends of individuals who died with DLB in the past 5 years. Topics included the time from symptom onset and diagnosis to death, cause of death, advance directive completion, end-of-life education, hospice use, and location of death. | | | [7] |
| **3. Resource use and costs (n=4) –** Captures the use of health, social, and informal care services by patients and CPs, along with the associated financial costs and contributing factors. | | | | |
| To characterise use and costs of paid and unpaid care over time in a cohort of PLWD in Britain and explore the relationship between cohort members’ demographic and clinical characteristics and service costs. Investigators calculated costs of health and social services, unpaid care, and out-of-pocket expenditure for PLWD participating in three waves of the IDEAL cohort. | Mixed methods longitudinal study. At Wave 1, dyads completed questions from the participant questionnaire taken from the CSRI on paid and unpaid care activities. PLWD without a participating CP completed these questions on their own. CPs also completed questions taken from the CSRI on work time lost to caring responsibilities. Structuring of resource use and cost questions were modified for subsequent waves. | | | [8] |
| To compare resource use and costs in patients with DLB and AD and assess determinants of costs of care in DLB. | Quantitative study. People with DLB were either examined in their homes or at the clinic together with their primary CP. Resource use was measured using the RUD-Lite. | | | [9] |
| To assess the feasibility of undertaking a cluster randomised study of the introduction of an evidence-based management toolkit for LBD, assessing the outcomes for patients and CPs as secondary measures. | A pragmatic, cluster-randomised mixed methods trial. Health economic data on the use of health and social care resources were collected using a questionnaire administered to care partners at baseline, 3 months, and 6 months. The bespoke questionnaire was developed based on items from the CSRI. | | | [10] |
| To evaluate the current economic costs of DLB and its related factors. | Quantitative study. Patients' per capita annual economic costs related to DLB in the year preceding the interview were evaluated, and factors related to the costs were assessed using regression analysis. Patients with DLB and their CPs were interviewed, during which statistics on the costs of the disease over a 1-year period were collected. Electronic medical records were used to gather data on specialised inpatient and outpatient healthcare costs. When information could not be obtained from the electronic medical records system, investigators conducted face-to-face interviews using a unified standard electronic questionnaire. | | | [11] |
| **4. Perspectives on clinical and assessment instruments (n=5) -** Captures views and reflections on the use, development, or acceptability of tools and measures used in clinical or research contexts. | | | | |
| To ensure that assessment toolkits for improving the diagnosis of LBD were acceptable to staff and could be integrated into current assessment procedures in busy clinical services. | Qualitative investigation within a mixed methods project (DIAMOND-Lewy). Interviews were conducted with patients and CPs for their perspectives on the appropriateness and clarity of the toolkits. There was no reference to an interview guide. | | | [12] |
| Work package 5 of DIAMOND-Lewy aimed to capture feedback on the acceptability of the toolkits. | Mixed methods study. This work package included a qualitative investigation of patient and CP views the toolkits' acceptability. Semi-structured interviews conducted. There was no reference to an interview guide. | | | [13] |
| To examine the feasibility and acceptability of a psychosocial intervention for DLB designed to build coping capability. A secondary objective was to identify the most appropriate primary outcome measure to inform a future multi-centre evaluation study. | Mixed methods trial design. As part of this process, feedback on the acceptability of potential outcome measures was gathered through dyadic interviews. | | | [14] |
| To describe the development and validity testing of the Lewy Body Disease Caregiver Activities Scale (LBD-CAS). | Mixed methods study with a methodological design. Think-aloud interviews were conducted with CPs who were asked to think aloud and comment on the clarity, relevance, appropriateness, and wording of the items. | | | [15] |
| To determine parameters influencing CPs’ quality of life and their possible link with patients’ QoL. | Quantitative study. CPs completed a scale measuring QoL developed from data from previous PIXEL studies (i.e., a novel measure). Reactions of families to the scale are reported. | | | [16] |
| **5. Experience and impact of the COVID-19 pandemic and lockdown (n=6) -** Captures the impact of the COVID-19 pandemic and related lockdown measures on patients and CPs. Sub-topics include: (1) The impact of COVID-19 and lockdown on patients and CPs (broad focus), and (2) the subjective experience of time during this period for patients. | | | | |
| **5.1. Impact of COVID-19 and lockdown on patients and care partners (n=5)** | | | | |
| To describe the lockdown’s impact amongst people affected by young onset, non-memory-led and inherited dementias and their CPs. | A mixed methods design was used. Data were collected via a bespoke web survey administered to PLWD and family CPs to explore the impact of lockdown on cognitive symptoms, psychological well-being, ability to do things, social connections, general health, and medication changes. Questions also explored CP health, care-related support, helpful strategies, and any positives found in the situation. | | | [17] |
| To investigate clinical changes in patients with AD and other dementia subtypes, and to evaluate CPs' distress during the COVID-19 quarantine. | Quantitative study. A bespoke survey was administered to family CPs over the phone. Main outcomes were patients’ changes in cognitive, behavioural, and motor symptoms. Secondary outcomes were effects on CPs’ psychological features. | | | [18] |
| To assess modifications of neuropsychiatric symptoms during quarantine in PLWD and their CPs. | Quantitative sub-study of a multicentre, nation-wide survey. A bespoke survey was administered to family CPs over the phone which queried variations in BPSD, and associations with disease type, severity, gender, and CP’s stress burden were analysed. | | | [19] |
| To examine changes in behaviour among PLWD and to look for associations between the evolution of BPSD and CPs’ mental health in the context of COVID-19. | Quantitative study. A web survey, which comprised both standardised instruments and novel questions, was completed by CPs. For DLB CPs, perspective data related to novel measures of changes or emergences of BPSD since lockdown. | | | [20] |
| To investigate the effects of the pandemic on anxiety, depression and care burden in CPs of nursing patients with AD, DLB and MCI over a one-year period, and to explore predictive factors, particularly physical activity, social contact and sleep disturbance, in relation to the worsening or improvement of these concerns. | Longitudinal, quantitative study. A one-year follow-up survey of patients and their CP was conducted to assess changes in physical activity, social contact, sleep quality, CP burden, anxiety and depression. Standardised measures included the ZBI, GAD-7, and PHQ-9. Non-standardised measures included a semi-structured questionnaire measuring social contact, and a health-related questionnaire assessing physical activity and caring time. | | | [21] |
| **5.2 Patient’s subjective experience of time (n=1)** | | | | |
| To assess the subjective experience of the passage of time for present and past time intervals during the lockdown due to coronavirus disease for people with DLB. | Quantitative study. The STQ was administered via telephone to patients and compared with CPs of a similar age. | | | [22] |
| **6. Experiences and perspectives on non-pharmacological interventions (n=12)-** Captures patient and CP experiences of, and views related to, non-pharmacological interventions such as music therapy, cognitive rehabilitation, and psychosocial interventions. | | | | |
| To explore the impact of a home-based, individualised cognitive stimulation therapy adapted for people with PD-MCI, PDD or DLB, and to evaluate the acceptability of the intervention and the feasibility of a full-scale RCT. | Mixed methods trial. Post-intervention dyadic, semi-structured interviews explored the acceptability and usability of the intervention manual, adherence (including barriers and facilitators), and the intervention's impact. | | | [23] |
| To explore how people with DLB experience daily life following a pacemaker implant to manage associated symptoms of bradyarrhythmia. | Qualitative case study design. Two men with DLB and their spouse CPs were repeatedly interviewed as a dyad within one year of receiving a dual-chamber rate-adaptive pacemaker for managing sick sinus syndrome. | | | [24] |
| To examine the feasibility and acceptability of a psychosocial intervention for DLB designed to build coping capability. A secondary objective was to identify the most appropriate primary outcome measure to inform a future multi-centre evaluation study. | Mixed methods trial. Post-intervention face-to-face dyadic interviews were conducted to explore experiences with the intervention. | | | [14] |
| To assess the feasibility of a peer mentor-led educational intervention and its impact on LBD family CPs' knowledge, dementia attitudes, and mastery. | Mixed methods trial. Focus groups with CPs guided the refinement of intervention materials. Weekly questionnaires (non-standardised measure) during the intervention included novel questions on mentoring preferences, adherence, satisfaction, and whether participants would consider future involvement or recommend the intervention. | | | [25] |
| To identify care navigator approaches used to address CP burden in dementia as part of a dementia care navigation program. | A qualitative design was used, with data collected through interviews, focus groups, observations, and qualitative case study analysis. Both formal and informal CP perspectives related to a care nagivation program were captured. | | | [26] |
| To explore the perceived effects of three non-pharmacological interventions in managing symptoms in older adults with AD or DLB from family CPs’ perspectives. | Qualitative study. Focus groups with CPs, guided by a bespoke questionnaire, explored whether changes were observed following the intervention. | | | [27] |
| To evaluate the effectiveness of a guide to music use for PLWD which was trialled with patient-CP dyads in both long-term residential care and home-based care. | To assess changes in mood and behaviour at each listening session, the MiDAS was used. This scale was completed by a CP or family member before and after each session. | | | [28] |
| To evaluate the feasibility, acceptability, and tolerability of a home-based, CP-delivered cognitive stimulation therapy for people with PDD or DLB and their CPs. | A mixed methods trial design was used. Training evaluation forms (completed by CPs), skills self-assessments, therapy diaries, and post-intervention dyadic interviews (patient/participant-CP dyads) assessed perspectives on feasibility, acceptability, and tolerability. | | | [29] |
| To examine the use of dolls in two nursing homes. | Using a mixed methods design, bespoke post-intervention questionnaires were used to query formal CPs' perceptions of doll therapy, their overall impression of doll use, and to ask them to hypothesise the mechanisms of change resulting from doll use. | | | [30] |
| To prospectively evaluate the feasibility and usability of the Partner in Balance intervention for CPs of persons with YOD, and to identify directions for further improvement. Preliminary effects were examined to evaluate if the intervention facilitated role adaptation. | A mixed methods trial design was used. Telephone interviews, using the PPQ (a semi-structured interview developed for the Partner in Balance study [31]), were conducted with participants (spouses and family members of people with young-onset dementia) to evaluate usability, feasibility, acceptability, and perceptions of intervention content. | | | [32] |
| To assess goal setting in early-PDD or DLB including the types of goals set and deemed as important, as well as the nature and themes of goal statements. The goal performance ratings from the perspectives of participants with DLB and PDD and their CPs was also compared. | Mixed methods trial. The BGSI, a semi-structured interview, was used to identify participants’ (patients) rehabilitation goals and their ratings of performance and satisfaction with these goals. | | | [33] |
| To examine the appropriateness and feasibility of cognitive rehabilitation for people with dementias associated with Parkinson's in a pilot randomised controlled study. It also assessed the usefulness of outcome measures and obtaining effect sizes to inform the development of future RCTs of cognitive rehabilitation in PDD and DLB. | Mixed methods trial. The primary outcome was participants' (patients) ratings of goal attainment and satisfaction, measured using the BGSI, a semi-structured interview. | | | [34] |
| **7. Social connection and engagement (n=17) -** Captures the social and relational experiences of patients and their CPs, including communication, connectedness, relationship quality, and expressions of engagement such as advocacy and campaigning. Sub-topics include: (1) communication difficulties and strategies, (2) the experience of campaigning, (3) Social support, isolation, and connectedness, and (4) dyadic relationship quality. | | | | |
| **7.1 Patient and care partner perspectives on communication difficulties and strategies (n=2)** | | | | |
| To examine the impact of cognitive impairment on the interaction between people with PD and their CPs. The study also compared the conversational profiles of (i) people with PD who developed DLB and (ii) those whose cognitive impairment was attributed to subcortical pathology. | Using a qualitative conversation analytic approach, CPs reported communication challenges and strategies during an interview based on the CAPPCI. | | | [35] |
| To explore resources and barriers in communication in DLB. | A mixed methods case study design involving dyadic, face-to-face interviews based on a bespoke guide, exploring perceptions of communicative function in daily life (more specifically, experiences of barriers and resources in daily life and how the couple adjust to change). | | | [36] |
| **7.2 Patient experience of campaigning (n=1)** | | | | |
| Non-structured narrative reflection detailing an interview with an individual with DLB, in which they discuss their experience of campaigning through the internet. | Non-structured narrative reflection detailing an interview with an individual with DLB, in which they discuss their experience of campaigning through the internet. | | | [37] |
| **7.3 Patient and care partner perspectives on social support, isolation, and connectedness (n=8)** | | | | |
| To explore differences in CP self-efficacy and their associated factors between DLB and AD CPs. | Quantitative study. CPs completed a series of instruments, including the SSQ to assess social support and the R-UCLA-LS to assess perceived loneliness. | | | [38] |
| To examine the differences in CP experiences of grief, burden, and QoL between DLB, PDD, and AD. | Quantitative study. Data were collected via a web survey which included a CP-reported MOS-SS to compare social support across dementia etiologies. | | | [39] |
| To identify factors associated with depressive symptoms in DLB, AD, and PDD CPs. It compared the burden, grief, and depressive symptoms of CPs. It also examined the role of CPs' self-efficacy and received social support in improving psychological well-being and investigated whether self-efficacy mediates the relationship between CP burden and psychological well-being, as well as between CP grief and psychological well-being. | Quantitative study. Data were collected via a web survey which was completed by CPs. The survey included the MOS-SS to measure level of social support. | | | [40] |
| To examine differences in the experiences of spouse versus adult child CPs of individuals with DLB. | Quantitative study. Data were collected via a web survey which included novel questions assessing the CP’s social support and social network. | | | [41] |
| To assess the feasibility of a peer mentor-led educational intervention and its impact on LBD family CPs' knowledge, dementia attitudes, and mastery. | Mixed methods trial. The UCLA-LS was included as a pre- and post-outcome measure to measure CP loneliness. | | | [25] |
| To examine the dimensions of subjective burden of CPs of patients with LBD using data from a quantitative online survey. | Quantitative survey. An online survey was conducted, including a novel measure of isolation that assessed whether CPs felt isolated due to limited awareness of LBD. | | | [42] |
| To describe the lockdown’s impact amongst people affected by young onset, non-memory-led and inherited dementias and their CPs. | A mixed methods design was used. Data were collected via a bespoke web survey administered to PLWD and family CPs. Survey captured data on the patient’s social connections during lockdown. | | | [17] |
| To investigate the effects of the pandemic on anxiety, depression and care burden in CPs of nursing patients with AD, DLB and MCI over a one-year period, and to explore predictive factors, particularly physical activity, social contact and sleep disturbance, in relation to the worsening or improvement of these concerns. | Longitudinal, quantitative study. A one-year follow-up survey of patients and their CP included an assessment of the lockdown’s impact on care partner social contact via a bespoke semi-structured questionnaire. | | | [21] |
| **7.4 Patient and care partner perspectives on dyadic relationship quality (n=6)** | | | | |
| To explore changes in long-term intimate relationships in Parkinson’s-related dementia, as perceived by spousal CPs. | Qualitative study. Participants completed semi-structured face-to-face interviews based on a bespoke guide. | | | [43] |
| To explore the impact of a home-based, individualised cognitive stimulation therapy adapted for a population of people with PD-MCI, PDD or DLB. They also evaluated the acceptability of the intervention and the feasibility of conducting a full-scale RCT. | Mixed methods trial. A range of exploratory outcomes were reported, including the RSS (for both patients and CPs) to assess satisfaction with the dyadic relationship, the DRS (for CPs) to assess positive dyadic interaction and negative strain, and the FCR (for CPs) to assess specific feelings associated to care provision. | | | [23] |
| To explore and compare levels of mental health, care burden, and relationship satisfaction among caregiving spouses of people with PD-MCI, PDD or DLB. | Quantitative study. CPs completed a battery of rating scales including the RSS (assessing relationship satisfaction), the DRS (assessing positive dyadic interaction and negative strain), and the FCR (assessing specific feelings associated to care provision in terms of satisfaction with the caring role, resentment, and anger). | | | [44] |
| To assess psychometric properties and describe clinimetric properties of PROMs completed by informant CPs of people with PD-MCI/PDD/DLB to gain insight into the suitability, reliability and usability of the scales in this population. | Data were extracted from the INVEST trial including CP scores for the the FCR (assessing feelings related to care provision), DRS (assessing positive dyadic interaction and dyadic strain) and RSS (assessing communication, conflict resultion, affection and intimacy). | | | [45] |
| To document the lived experience of spouse CPs of people with early-onset dementia. | Qualitative study. Face-to-face interviews were conducted with CPs which were based on interviews developed by Clare et al [46]. These explored experiences within the disease trajectory, including first symptoms, the quest for diagnosis, main difficulties encountered, and the evolution of relationships within the dyad and family. | | | [47] |
| To provide an analytical example of applying Murray’s levels of narrative analysis in health psychology to longitudinal narrative data from a couple living with LBD. | Qualitative study. Data were drawn from a PhD project exploring the day-to-day experiences of couples living with LBD (healthcare interactions, relationship changes, becoming a carer). Longitudinal, narrative, dyadic interview data, based on a bespoke guide, from one couple are reported to illustrate the analytical process. | | | [48] |
| **8. Self, identity, and coping (n= 19) -** Captures how people with DLB and their CPs understand, adapt to, and cope with the condition. Sub-topics include: (1) self-perception, (2) self-efficacy, resilience, and coping, and (3) attitudes towards own ageing. | | | | |
| **8.1 Patient and care partner self-perception (n=3)** | | | | |
| To explore the subjective experience of living with DLB. | A qualitative study using in-depth interviews and interpretative phenomenological analysis. Five males with DLB participated. While there was no strict interview guide, prompts and questions were used to explore topics which included the individual’s self-perception, with participants guiding the interviews toward topics of personal importance. | | | [49] |
| To understand the carer identification practices of older partners providing end-of-life care. | A qualitative, longitudinal design. Narrative interviews, based on a bespoke guide, were conducted with CPs. The study reports on LBD CPs' attitudes towards the label of ‘carer’. | | | [50] |
| To provide an analytical example of applying Murray’s levels of narrative analysis in health psychology to longitudinal narrative data from a couple living with LBD. | Qualitative study. Data were drawn from a PhD project exploring the day-to-day experiences of couples living with LBD (including becoming a carer). Longitudinal, narrative, dyadic interview data, based on a bespoke guide, from one couple are reported to illustrate the analytical process. | | | [48] |
| **8.2 Patient and care partner self-efficacy, resilience, and coping (n=16)** | | | | |
| To describe the lockdown’s impact amongst people affected by young onset, non-memory-led and inherited dementias and their CPs. | A mixed methods design was used. Data were collected via a bespoke web survey administered to PLWD and family CPs. Survey captured data on the care partners coping strategies during lockdown. | | | [17] |
| To explore the causes, consequences, and potential solutions to toilet-use and incontinence problems for PLWD and their CPs. | A qualitative study involving interviews with PLWD and CPs. A bespoke, semi-structured topic guide queried solutions to toilet-use and incontinence problems. | | | [51] |
| To explore the subjective experience of living with DLB. | A qualitative study of in-depth interviews using interpretative phenomenological analysis. Five males with DLB participated. Interviews explored the patient’s illness experience, barriers and facilitators of well-being, and coping strategies. Participants guided the interviews towards topics of personal importance. | | | [49] |
| To determine the needs, concerns, strategies, and advice of family CPs of persons with LBD. | A qualitative study involving a convenience sample of LBD caregivers interviewed by telephone regarding their caregiving needs, concerns, strategies, and advice. A pre-existing topic guide, based on the work of Bakas et al. was used [52]. | | | [53] |
| To clarify the visual hallucination-related difficulties experienced by people with DLB, as well as their coping methods. | A qualitative study involving data collected through semi-structured interviews with patients, with input also obtained from family members. A bespoke topic guide was used. | | | [54] |
| To examine the feasibility and acceptability of a psychosocial intervention for DLB designed to build coping capability. A secondary objective was to identify the most appropriate primary outcome measures to inform a future multi-centre evaluation study. | Mixed methods trial with pre- and post-outcome measures, including the GSES for people with DLB and CPs (measuring self-efficacy), the FCP-SEMD scale for CPs (measuring certainty in coping with 10 caregiving situations), the RSCSE for CPs (measuring self-efficacy), and a novel Likert-scale measure completed by CPs, assessing perceived changes in their capability to cope. | | | [14] |
| To examine the appropriateness and feasibility of cognitive rehabilitation for people with dementias associated with Parkinson's in a pilot randomised controlled study. Additional aims included assessing the usefulness of outcome measures and obtaining effect sizes to inform the development of future RCTs of cognitive rehabilitation in PDD and DLB. | A mixed methods trial with secondary outcomes completed by both patients and CPs, including the GSES to measure self-efficacy. | | | [34] |
| To identify care navigator (formal CPs) approaches used to address CP burden in dementia as part of a dementia care navigation program. | Qualitative methods (interviews, focus groups, and observations) and case study analysis were used to identify care navigator approaches to addressing caregiver burden in dementia as part of a dementia care navigation programme. | | | [26] |
| To describe the different experiences of DLB and AD CPs and to explore how coping strategies may be applied to support these CPs. | Qualitative study within a PhD thesis, where data were collected through two focus groups with DLB CPs, based on a bespoke, semi-structured topic guide. | | | [55] |
| To examine differences in DLB, AD and PDD CP experiences of grief, burden, and QoL. | Quantitative study using a web survey which included novel questions assessing confidence in ability to provide care (adapted from the Dementia Care Confidence scale), mastery, and self-efficacy. | | | [39] |
| To identify factors associated with depressive symptoms in family CPs of persons with DLB, AD, and PDD. It compared the burden, grief, and depressive symptoms of CPs across these conditions. It examined the role of CPs' self-efficacy and received social support in improving psychological well-being and investigated whether self-efficacy mediates the relationship between CP burden and psychological well-being, as well as between CP grief and psychological well-being. | Quantitative study involving a web survey completed by CPs. The survey included the RSCSE which measured CP self-efficacy. | | | [40] |
| To explore the impact of a home-based, individualised cognitive stimulation therapy adapted for a population of people with PD-MCI, PDD or DLB. They also evaluated the acceptability of the intervention and the feasibility of conducting a full-scale RCT. | Mixed methods trial design. The BRS was used to measure resilience as an outcome for individuals with PD-MCI, PDD, and DLB, as well as for their caregivers. | | | [23] |
| To explore and compare levels of mental health, care burden, and relationship satisfaction among PD-MCI, PDD or DLB. | Quantitative study. CPs completed a battery of rating scales, including the BRS to assess resilience. | | | [44] |
| Aimed to assess psychometric properties and describe clinimetric properties of the PROMs completed by informal CPs of people with PD-MCI/PDD/DLB to gain insight into the suitability, reliability, and usability of the scales in this population and to help future researchers and clinicians with decision-making regarding appropriate instruments. | Quantitative data from CP-reported measures were extracted from the INVEST trial, including CP resilience scores derived from the BRS. | | | [45] |
| To assess the feasibility of a peer mentor-led educational intervention and its impact on LBD family CPs' knowledge, dementia attitudes, and mastery. | Mixed methods trial. The PMS was used as a pre- and post-outcome measure to assess the extent that CPs believe they control their own lives. The DAS was included to assess feelings towards and confidence caring for PLWD. | | | [25] |
| To examine the differences in CP self-efficacy and their associated factors between DLB and AD CPs. | A quantitative approach was used. As part of a comprehensive questionnaire of CPs, the RSCSE was completed to assess caregiving self-efficacy. | | | [38] |
| **8.3 Patient attitudes towards own ageing (n=1)** | | | | |
| To investigate whether PLWD have more negatives attitudes toward own ageing (ATOA) than those without dementia and whether cognition and dementia subtype are associated with ATOA in PLWD. | Quantitative, cross-sectional data from the IDEAL and PROTECT studies were used to compare ATOA between PLwD and individuals without dementia. The ATOA questionnaire assessed ATOA in both studies from which data is drawn. In the IDEAL cohort, this questionnaire was administered as part of a questionnaire administered during interviews. In the PROTECT cohort, the measure was self-completed through the PROTECT online platform. | | | [56] |
| **9. Experiences and needs related to care (n=23) -** Captures the care-related experiences, support needs, and treatment preferences of people with DLB and their CPs from the pre-diagnostic stage through to advanced stages of dementia (before end-of-life). The topic includes helpful or challenging aspects of care, unmet needs, access to services, information needs, care planning, treatment experiences and preferences, reflections on care satisfaction, and formal caregiving experiences. It also encompasses the diagnostic process and decisions about future care, such as institutionalisation. Sub-topics include: (1) care, support, and treatment needs and experiences from pre-diagnosis to advanced stages, (2) perspectives and experiences of formal care staff on managing LBD, and (3) desire to institutionalise. | | | | |
| **9.1** **Patient and care partner needs and experiences of care, support, and treatment from pre-diagnosis to advanced stages (n=21)** | | | | |
| To investigate the information behaviours of PLWD. It also aimed to identify their motivations for changing information behaviours over time. | Qualitative study. Data were collected through contextual inquiry sessions with PLWD, consisting of interviews and direct observation. Findings were reported on the nature, content, and evolution of information behaviours, as well as motivations for changes over time. | | | [57] |
| To investigate aspects of care that are helpful and unmet needs. | Qualitative design. Investigators conducted telephone interviews with individuals living with DLB and their CPs, using a bespoke, semi-structured questionnaire to explore helpful aspects of care and unmet needs. | | | [58] |
| To identify the needs, concerns, strategies, and advice of family CPs of persons with LBD. | Qualitative design. A convenience sample of LBD caregivers was interviewed by telephone about their caregiving needs, concerns, strategies, and advice, using a pre-existing topic guide based on the work of Bakas et al [52]. | | | [53] |
| To identify care navigator approaches used to address CP burden in dementia as part of a dementia care navigation program. | A qualitative design was used, involving a combination of interviews, focus groups, observations, and qualitative case study analysis to collect perspectives and experiences related to a care programme. | | | [26] |
| To ascertain the unmet needs of LBD CPs and collect data to inform educational programming and enhance CP support. | Quantitative study. A web survey of CPs was conducted, including novel Likert scale questions to capture descriptive assessments of the LBD CP experience and requested services. | | | [59] |
| To evaluate the diagnostic concordance between video-based vs self-reported diagnoses of MSA, PSP, DLB, and CBS. | Quantitative study. Patient satisfaction with video-based visits was assessed using a bespoke survey. | | | [60] |
| Explore family CPs’ experiences of the LBD Admiral Nurse service. | Qualitative study. Telephone interviews were conducted with CPs using a bespoke, semi-structured questionnaire to explore helpful aspects of care and unmet needs. | | | [61] |
| To identify the components of post-diagnostic dementia support. | Qualitative study. A qualitative design was used, involving telephone or face-to-face interviews with PLWD and CPs, as well as observations of direct service delivery. Perspectives on post-diagnostic support content were explored. | | | [62] |
| To examine the dimensions of subjective burden of CPs of patients with LBD using data from a quantitative online survey. | Quantitative study. Data were obtained from a web survey survey of 611 respondents currently involved in the care of a relative with LBD. The survey included novel questions on difficulty finding a clinician, coordinating medical care, and overall evaluation of the help received. | | | [42] |
| Non-structured narrative reflection offering a CP’s perspective on DLB progression, highlighting challenges and delays in diagnosis, complexities of arranging care, and personal recommendations. | Non-structured narrative reflection presenting a CP’s perspective on DLB progression, focusing on challenges and delays in diagnosis, complexities in arranging suitable care, and personal recommendations. | | | [63] |
| To provide an analytical example of applying Murray’s levels of narrative analysis in health psychology to longitudinal narrative data from a couple living with LBD. | Qualitative study. Data were drawn from a PhD project exploring the day-to-day experiences of couples living with LBD, including healthcare interactions. Longitudinal, narrative, dyadic interview data, based on a bespoke guide, from one couple are reported to illustrate the analytical process. | | | [48] |
| To document the lived experience of spouse CPs of people with early-onset dementia. | Qualitative study. Face-to-face interviews were conducted with CPs, based on interviews developed by Clare et al. [46]. These explored experiences within the disease trajectory, including first symptoms, the quest for diagnosis, main difficulties encountered, and the evolution of relationships within the dyad and family. | | | [47] |
| To identify support and information needs following a diagnosis of DLB. | Quantitative design. People with DLB and their family members completed a web survey focusing on past experiences of information and support received, as well as information and support needs that would have been beneficial at the time of diagnosis (novel questions). | | | [64] |
| To ascertain the experiences of LBD CPs and collect data to improve diagnosis and management of LBD. | Quantitative study. A web survey of CPs was conducted to explore the challenges, burdens, and frustrations faced by LBD CPs in obtaining a diagnosis and care for the patient (novel questions). | | | [65] |
| Non-structured narrative reflection written from the perspective of a child reflecting on their experience of their parent living with DLB. | Reflection includes a discussion on obtaining a diagnosis. | | | [66] |
| To assess the satisfaction of DLB patients, CPs, and their attending physicians with medication for five DLB symptom domains. | This was a sub-analysis of a cross-sectional, questionnaire-based survey study. A bespoke survey was completed by patients, CPs, and clinicians. | | | [67] |
| Report on lumateperone for treating psychotic symptoms in LBD. | Quantitative case report. Family CPs reported on the patient’s response to treatment. | | | [68] |
| Report on response to donepezil treatment in individuals with DLB. | Quantitative case series. Family members’ comments on the patients’ treatment experiences were reported. | | | [69] |
| A commentary article presenting a clinician's experiences with treatment strategies for DLB. Insights into the treatment experience from the perspective of the patient and spouse also reported. | A commentary article presenting a clinician's experiences with treatment strategies for DLB. Insights into the treatment experience from the perspective of the patient and spouse also reported. | | | [70] |
| To examine the treatment needs of patients with DLB and their CPs, and the extent to which attending physicians understand these needs. | Quantitative study. Data were collected through a bespoke survey for people with DLB and CPs. Fifty-two DLB symptoms were pre-selected and classified into seven domains. Treatment needs were defined as the 'symptom causing most distress,' and the frequency of each response was tabulated. | | | [71] |
| To describe the lockdown’s impact amongst people affected by young onset, non-memory-led and inherited dementias and their CPs. | A mixed methods design was used. Data were collected via a bespoke web survey administered to PLWD and family CPs. Survey captured data on care-related support during lockdown, as well as medication changes due to changing symptoms. | | | [17] |
| **9.2 Perspectives and experiences of formal care staff on managing LBD (n=2)** | | | | |
| To identify care navigator approaches used to address CP burden in dementia as part of a dementia care navigation program. | Qualitative study. A combination of interviews, focus groups, observations, and qualitative case study analysis were used to identify strategies employed by formal CPs. | | | [26] |
| To estimate the prevalence of DLB and PDD in care home settings as well as staff’s level of knowledge about DLB and PDD. | Mixed methods study. Data were collected in two ways: (1) Bespoke survey comprising novel questions on the prevalence of LBD or LBD symptoms among residents, the challenges of caring for people with LBD, whether care home staff have the knowledge and skills to care for residents with LBD, and available sources of advice or training for specialist support, and (2) A combination of individual interviews, paired interviews and focus groups with formal CPs to explore experiences of caring, information and advice about the care of residents with LBD, and staff training in relation to LBD. | | | [72] |
| **9.3 Care partner desire to institutionalise (n=1)** | | | | |
| To examine CP burden using the ZBI and explore factors influencing the decision to place a dementia care recipient in a structured living facility. | Desire to institutionalise was assessed using the DIS. Although no scores were reported, outcomes from the assessment were discussed. | | | [73] |
| **10. Life satisfaction, QoL and HRQoL (n=25) -** Captures QoL, HRQoL, and life satisfaction in patients and their CPs. Sub-topics include: (1) patient QoL and HRQoL, (2) CP QoL and HRQoL, and (3) life satisfaction of both patients and CPs. | | | | |
| **10.1 Patient’s QoL and HRQoL (n=23)** | | | | |
| To examine the treatment needs of patients with DLB and their CPs and the extent to which the attending physicians understand these needs. | | Quantitative study. Participating patients and CPs underwent several screening tests including the SF-8 to measure quality of life (could be proxy reported). | | [71] |
| To determine parameters influencing CP’s QoL and its possible link with patients’ QoL. | | Quantitative study. Patients’ QoL was assessed by the clinician/physician with the CP, according to Rabin’s ADRQL scale. | | [16] |
| To investigate QoL and the effect of memantine in patients with LBD. | | Secondary analysis of a randomised controlled study. Both patients and CPs reported on patient QoL which was measured using the QoL-AD scale. | | [74] |
| To assess the efficacy, safety, and tolerability of armodafinil for hypersomnia associated with DLB. | | A quantitative trial. Patient QoL (proxy or self-reported) was a secondary efficacy measure and assessed using the LASA. | | [75] |
| To assess the feasibility of undertaking a cluster randomised study of the introduction of an evidence-based management toolkit for LBD, assessing the outcomes for patients and CPs as secondary measures. | | A pragmatic cluster RCT (part of a mixed methods project). Patient QoL was assessed using self- and proxy-reported DEMQOL and EQ-5D-5L as part of the outcomes. | | [10] |
| To compare the QoL of individuals with different dementias, especially focusing on positive and negative affect, using an objective QoL scale for dementia. | | Quantitative study. The QOL-D was completed by family CPs to provide information on positive affect, negative affect and action, communication, restlessness, attachment to others, spontaneity, and activity. | | [76] |
| To compare QoL in patients with DLB and AD and investigate determinants of QoL in DLB. | | Quantitative study. The EQ-5D-3L and QoL-AD instruments were administered to both patients and CPs (as proxies) to assess QoL. | | [77] |
| To investigate the associations between core and suggestive DLB symptoms and different aspects of disease burden, i.e., instrumental activities of daily living, QoL and CP burden. | | Quantitative, longitudinal study. QoL data were collected using the QoL-AD. Patients’ QoL was assessed twice: once as self-rated and once as CP-rated. The weighted QoL-AD composite score was calculated by combining these scores, with the patients’ self-rated score weighing twice as much as the CP-rated QoL score. | | [78] |
| To identify the frequency of suicidal ideation in LBD and the factors associated with it. | | Quantitative study. A retrospective review of a prospectively collected longitudinal database was conducted. QoL was assessed using the PDQ-39. The database did not capture whether intake forms were completed by the patient or CP. | | [79] |
| To explore the subjective experience of living with DLB. | | Qualitative study. Following interviews, participants completed the QoL-AD. | | [49] |
| To assess the safety and tolerability of low frequency deep brain stimulation of the nucleus basalis of Meynert in DLB patients and exploring its potential effects on both clinical symptoms and functional connectivity in underlying cognitive networks. | | Trial design. Trial endpoints included patient QoL, assessed using the QoL-AD through both self- and proxy-reports. | | [80] |
| To compare adjunct zonisamide versus an increased levodopa dose. | | Quantitative trial. Patient QoL was included as a secondary trial endpoint and assessed using the EQ-5D-5L. CPs were available to provide additional patient information. | | [81] |
| To explore the impact of a home-based, individualised cognitive stimulation therapy adapted for a population of people with PD-MCI, PDD or DLB. They also evaluated the acceptability of the intervention and the feasibility of conducting a full-scale RCT. | | A mixed methods trial. Exploratory outcomes included an assessment of QoL in patients using the PDQ-39 and EQ-5D-3L. | | [23] |
| To investigate the potential impact of dementia subtypes on the capability to live well for both PLWD and their CPs. | | A sub-study of a mixed-methods, five-year longitudinal cohort study. The analysis focused on 1283 dyads of community-dwelling PLWD and CPs in the Improving the experience of Dementia and Enhancing Active Life (IDEAL) project. ‘Capability to live well’ was defined using three measures, one of which was QoL, assessed in patients using the QoL-AD. | | [82] |
| To examine the appropriateness and feasibility of cognitive rehabilitation for people with dementias associated with Parkinson's in a pilot randomised controlled study. It also assessed the usefulness of outcome measures and obtaining effect sizes to inform the development of future RCTs of cognitive rehabilitation in PDD and DLB. | | Mixed methods trial. Patient QoL was assessed as a secondary outcome using the PDQ-8 and WHOQOL-BREF. | | [34] |
| To investigate determinants and trajectories of QoL in DLB compared to AD and controls. | | Quantitative longitudinal cohort study. Patients were invited for annual follow-up for at least two years, during which they completed the EQ-5D-3L and VAS. | | [83] |
| To identify associations with rate of change in cognition, everyday functioning and QoL in the DEvELOP cohort. | | Quantitative longitudinal cohort study. Patient QoL was assessed using the QoL-AD. | | [84] |
| To examine the differences in CP experiences of grief, burden, and QoL between DLB, PDD, and AD. | | Quantitative study. Data were collected via a web survey for CPs which included the QoL-AD to assess the patient’s QoL. | | [39] |
| To examine differences in the experiences of spouse versus adult child CPs of individuals with DLB. | | Quantitative study. Data were collected via a web survey for CPs which involved CPs rating their own and the patient’s QoL using the QoL-AD scale. | | [41] |
| To investigate tau in people with DLB using a second-generation tau positron emission tomography tracer as well as plasma p-tau181. | | Quantitative study. Clinical assessments included the EQ-5D-3L measuring QoL. Care/study partners were available to provide information. | | [85] |
| To investigate the clinical and imaging correlates of amyloid-beta deposition in DLB, particularly its relationship with cortical thickness in AD–prone regions and hippocampal volume. | | Quantitative study. The EQ-5D-3L was administered to capture QoL. Care/study partners were available to provide information. | | [86] |
| To measure HRQoL in individuals with different types of dementia, MCI, and healthy controls and assess agreement levels between participants and proxies. | | Quantitative study. HRQoL was measured using the SF-12-v2, separately for participants and proxies. | | [87] |
| To measure HRQoL in people with different primary degenerative dementias and compare them with 50+ years old individuals with MCI and healthy controls. | | Quantitative study. HRQoL was measured using the SF-12-v2 (self- and proxy-rated). | | [88] |
| **10.2 Care partner QoL and HRQoL (n=11)** | | | | |
| To examine the treatment needs of patients with DLB and their CPs and the extent to which the attending physicians understand these needs. | | Quantitative study. Participating patients and CPs underwent several screening tests including the SF-8 to measure QoL. | | [71] |
| To assess the efficacy, safety, and tolerability of armodafinil for hypersomnia associated with DLB. | | A quantitative trial. CP QoL was a secondary efficacy measure and assessed using the LASA. | | [75] |
| To assess the feasibility of undertaking a cluster randomised study of the introduction of an evidence-based management toolkit for LBD, assessing the outcomes for patients and carers as secondary measures. | | A pragmatic cluster RCT trial. Among the outcomes, CP QoL was assessed using the EQ-5D-5L. | | [10] |
| To explore the impact of a home-based, individualised cognitive stimulation therapy adapted for people with PD-MCI, PDD or DLB. They also evaluated the acceptability of the intervention and the feasibility of conducting a full-scale RCT. | | A mixed methods trial design was used. Exploratory outcomes included an assessment of CP QoL using the EQ-5D-3L. | | [23] |
| To investigate the potential impact of dementia subtypes on the capability of PLWD and CPs to live well. | | A sub-study of a mixed-methods, five-year longitudinal cohort study. The analysis was based on 1283 dyads of community-dwelling people with dementia and CPs in the Improving the experience of Dementia and Enhancing Active Life (IDEAL) project. ‘Capability to live well’ was defined using three measures, one of which was QoL, assessed for CPs using the WHOQOL-BREF. | | [82] |
| To examine the appropriateness and feasibility of cognitive rehabilitation for people with dementias associated with Parkinson's in a pilot randomised controlled study. It also assessed the usefulness of outcome measures and obtaining effect sizes to inform the development of future RCTs of cognitive rehabilitation in PDD and DLB. | | Mixed methods trial. CP QoL was assessed as a secondary outcome using the WHOQOL-BREF. | | [34] |
| To examine the differences in CP experiences of grief, burden, and QoL between DLB, PDD, and AD. | | Quantitative study. Data were collected using a web survey for CPs which included the QoL-AD to capture data on CP QoL. | | [39] |
| To examine differences in the experiences of spouse versus adult child CPs of individuals with DLB. | | Quantitative study. Data were collected using a web survey for CPs which involved CPs rating their own and the patient’s QoL using the QoL-AD scale. | | [41] |
| To determine parameters influencing CP’s QoL and its possible link with patients’ QoL. | | Quantitative study. CP QoL was assessed using a scale developed from data from previous PIXEL studies (i.e. non-standardised measure). | | [16] |
| To assess psychometric properties and describe clinimetric properties of PROMs completed by informant CPs of people with PD-MCI, PDD, or DLB to gain insight into the suitability, reliability, and usability of the scales in this population. | | Quantitative study. Data were extracted from the INVEST trial, including CP-reported EQ-5D-3L scores (assessing CP HRQoL). | | [45] |
| To explore and compare levels of mental health, care burden, and relationship satisfaction among caregiving spouses of people with PD-MCI, PDD or DLB. | | Quantitative study. CPs completed a battery of rating scales including the EQ-5D-3L which assessed HRQoL. | | [44] |
| **10.3. Patient and care partner life satisfaction (n=1)** | | | |  |
| To investigate the potential impact of dementia subtypes on the capability to live well for both PLWD and their CPs. | | A sub-study of a mixed-methods, five-year longitudinal cohort study. The analysis was based on 1283 dyads of community-dwelling people with dementia and CPs in the Improving the experience of Dementia and Enhancing Active Life (IDEAL) project. ‘Capability to live well’ was defined using three measures, one of which was satisfaction with life, self-rated by patients and CPs using the SwLS. | | [82] |
| **11. Perspectives related to the symptom and illness experience (n=34)-** Captures patient and CP perspectives on the lived experience of DLB symptoms and illness across multiple domains. Sub-topics include: (1) patient and care partner perspectives related to core DLB symptoms, (2) patient and care partner perspectives related to supportive or additional symptoms, (3) patient and CP perspectives on suicidality, (4) patient experience of pain, (5) patient perspectives related to disability, (6) patient general physical and mental health status, (7) patient and CP narrative comments on the symptom and illness experience, and (8) patient and CP perspectives on symptom changes during lockdown. | | | | |
| **11.1 Patient and care partner perspectives related to core DLB symptoms (n=8)** | | | | |
| Study aimed to clarify the visual hallucination-related difficulties experienced by people with DLB as well as their coping methods. | | A qualitative design involving interviews with people with DLB, based on a bespoke guide. Input was also obtained from family members. | | [54] |
| Describe an individual with DLB who presented long-lasting preclinical complex polymodal hallucinations. | | Mixed methods case report involving a semi-structured, open-ended interview with the patient to investigate, among other points, the experience of hallucinations and their emotional impact. | | [89] |
| Compare the phenomenology and characteristics of visual hallucinations in eye disease, PD, and LBD using the NEVHI interview based on patient information. | | Quantitative study involving secondary analysis. Data from five controlled cross-sectional visual hallucination studies were combined and analysed. The NEVHI interview screened the phenomenology of visual hallucinations and assessed their emotional and behavioural impact. Responses reflected patients’ perspectives. | | [90] |
| Investigate relationships between cognition, hallucination-specific insight, temporal, emotional and severity aspects of hallucinations and what aspects of the relationships might be the same or different when comparing neurodegenerative disease and eye disease. | | Quantitative study involving secondary analysis, using a combination of samples from extant studies on visual hallucinations across different neurological disorders and eye diseases. The NEVHI interview screened the phenomenology of visual hallucinations and assessed their emotional and behavioural impact. Responses reflected patients’ perspectives, as no CP data were available. | | [91] |
| Study aimed to (1) investigate the phenomenology of cognitive fluctuations in DLB by understanding CP experiences, (2) examine whether CPs considered sleep, or sleep disturbances, to influence the nature of cognitive fluctuations, and (3) understand the nature of habitual sleep patterns, sleep changes and sleep disturbances, and their impact on people with DLB and their CPs. | | Qualitative study involving telephone interviews with CPs, based on a bespoke guide, asking them to describe the nature, frequency, duration, and potential triggers of cognitive fluctuations experienced by the individual with DLB. Also queried the subjective sleep experience of the individual with DLB. | | [92] |
| To correlate neuropsychiatric and motor assessments with language and visual organization tests in LBD. | | Quantitative design. Clinical assessments included patient sleep satisfaction. Although no specific measures were referenced, the percentage of patients reporting sleep satisfaction was reported. | | [93] |
| To assess risk factors for age at dementia onset, as well as which neuropsychiatric features are associated with pharmacotherapy and signs and symptoms of LBD. | | Quantitative design. Clinical assessments included patient sleep satisfaction. Although no specific measures were referenced, the percentage of patients reporting sleep satisfaction was reported. | | [94] |
| To compare and distinguish demographic and neuropsychiatric features between LBD and *APOE*- **ε**3/**ε**3 late-onset AD. | | Quantitative design. Clinical assessments included patient sleep satisfaction. Although no specific measures were referenced, the percentage of patients reporting sleep satisfaction was reported. | | [95] |
| **11.2 Patient and care partner perspectives related to supportive or additional symptoms (n=13)** | | | | |
| To investigate the clinical features of delusional jealousy and possible mechanisms whereby delusional jealousy arises in patients with dementia. | | Quantitative design. Perspective data captured during a clinical assessment/clinical interview with the patient and primary CP (no assessment instruments or quantitative scoring). | | [96] |
| To update a previous report [97] and further detail the clinical phenomenology of SFMD in PD and DLB. | | Quantitative study. Assessments of SFMD were based on multiple methods: a semi-structured interview based on DSM-IV-TR manual. This interview investigated somatic complaints, with examples and a checklist presented to patients and CPs, focusing on SFMD traits, along with neuropsychological scale ratings, including the Somatisation Subscale of the SCL-90-R. | | [98] |
| To assess SFMD prevalence and impact in PD and DLB. | | Quantitative study. Categorisation of patients into SFMD and non-SFMD was based on multiple methods, one of which was a semi-structured interview based on the DSM-IV-TR manual. This interview investigated somatic complaints, with examples and a checklist presented to patients and CPs, focusing on SFMD traits. Two comparison studies were performed between patients with PD and patients with DLB, with and without SFMD. In the first, patients were evaluated with multiple standardised scales, including the SCL-90R Somatisation Subscale. | | [97] |
| To clarify the neuropsychological features of the patients with amyotrophic lateral sclerosis and parkinsonism-dementia complex of the Kii peninsula. | | A quantitative approach was used, and clinical interviews were conducted. Patients and their CPs were asked to report any history of personality change and a variety of other symptoms. No assessment instruments or quantitative scoring were used. | | [99] |
| The aim was to explore potential changes in personal tastes in a cohort of patients with early stages of DLB and test a potential relationship with insular atrophy using volumetric analysis. From a clinical perspective, the aim was to better understand behavioural changes in patients with DLB and develop a potential clinical tool that could reflect insular damage. | | Quantitative study. A bespoke questionnaire explored patients' perspectives on changes in personal tastes. It contained Likert-scale questions, and patients had the opportunity to exemplify their changes in taste. | | [100] |
| To identify personality traits that distinguish DLB from AD. | | Quantitative study. Participants enrolled in a longitudinal study were examined and followed up to autopsy. Annual interviews with a collateral source queried specific changes in personality, interests, and drives based on items from the BDS. | | [101] |
| To elucidate the relation between premorbid personality traits and BPSD in DLB and AD patients. | | Quantitative study. Each patient’s midlife personality traits were rated by a family member using the NEO-FFI questionnaire. | | [102] |
| To examine parkinsonian disorder patients' subjective experience of presence phenomena, and to retrospectively analyse their cognitive correlates to elucidate the emergence of information processing deficits in parkinsonian disorders. | | Mixed methods study. Patients endorsing presence phenomena completed a semi-structured interview about their subjective experience of the phenomena, focusing on the content and impact of the presence, the patient’s sense of control over their experiences, and the meaning patients attribute to their experiences. The interview was based on a bespoke guide. | | [103] |
| To evaluate the time of onset of autonomic symptoms in patients with DLB using the SCOPA-Aut questionnaire. | | Quantitative study. Patients completed the SCOPA-Aut questionnaire with CP confirmation. A sexual score was obtained from the SCOPA-Aut questionnaire, which assessed the domain of sexual dysfunction. The occurrence of each symptom was scored at 0 (never), 1 (sometimes), 2 (regularly), or 3 (often), allowing for a severity rating. Men’s erectile and ejaculation problems, as well as women’s vaginal lubrication and orgasm problems, were combined and analysed as a single item of sexual dysfunction. The time of onset of autonomic symptoms was also explored. | | [104] |
| Aimed to better understand the associations between sexual disinhibition in dementia and family CP burden, as well as the desire to place the care recipient in a structured living facility. | | Quantitative study. CPs completed a cross-sectional, online survey. Data for LBD participants were only available for a novel measure of sexual disinhibition. Participants were asked whether their care recipient displayed “lewd comments” or “lewd behaviour” in the past 2 weeks, using a 1 to 7 scale. They were also asked to report any behaviour they found “socially inappropriate and/or embarrassing” in a free-text response format. | | [105] |
| To clarify the frequency and characteristics of eating problems in people with DLB. | | A quantitative study examining the characteristics of eating and swallowing in patients, using a revised version of a questionnaire originally designed for FTD/AD. The questionnaire comprised 40 items investigating the following five domains: swallowing problems, appetite change, food preferences, eating habits, and other oral behaviours. Four additional questions relevant to DLB were added, and seven questions relating to FTD were deleted. Information was gathered from caregivers familiar with the patients’ eating problems. | | [106] |
| Explore the impact of caring for an individual with neurogenic orthostatic hypotension. | | Qualitative study involving telephone interviews with CPs, using a bespoke guide to query the impact of caring for the individual. | | [107] |
| To establish the range of causes, consequences and potential solutions for toilet-use and incontinence problems for PLWD and their CPs. | | Qualitative study involving face-to-face interviews with patients and CPs, using a bespoke guide to query causes and consequences of difficulties related to toilet-use. | | [51] |
| **11.3** **Patient and care partner perspectives on suicidality (n=2)** | | | | |
| To compare the frequencies, severity, and symptoms of depression between DLB and AD. | | Quantitative study. All patients and their main CP were interviewed by a neurologist or geriatric psychiatrist, and each depression symptom in the DSM-IV major depression criteria was compared between groups. This provided an indication of experiences of suicidality. | | [108] |
| To identify the frequency of suicidal ideation in LBD and the factors associated with it. | | Quantitative study. A retrospective review of a prospectively collected longitudinal database was conducted. BDI-II scores were extracted to analyse suicidal ideation. The database did not capture whether intake forms were patient- or CP-completed. | | [79] |
| **11.4 Patient experience of pain (n=2)** | | | | |
| To examine the prevalence and characteristics of pain in PD and other Parkinson plus syndromes, as well as patients’ use and response to pain medications. | | Quantitative study. A clinical interview involved the administration of a structured bespoke questionnaire requiring patients to report whether they experienced low-back pain and whether they experienced pain other than low-back pain. | | [109] |
| To assess the pain prevalence, pain intensity, and pain medication use in older patients with a diagnosed subtype of dementia, MCI, or SCI. | | Quantitative study. Patients were recruited from three outpatient memory clinics. In two clinics, pain was assessed using the BPI. In the third clinic, pain was assessed in two ways: (1) during a general physical examination as part of the clinical diagnostic procedure, where all patients were questioned about their physical complaints, including pain, and (2) using an 11-point numerical rating scale to assess pain intensity, identical to that used in the other two clinics. | | [110] |
| **11.5 Patient perspectives related to** **disability (n=1)** | | | | |
| To illustrate the use of time-budget methodology as a means of measuring disability within the framework of the World Health Organisation (WHO) International Classification of Functioning, Disability and Health in patients with neurodegenerative disease. | | Mixed methods study. The 'Yesterday Interview' was used to reconstruct the preceding 24-hour period in terms of activity, social and environmental context, and subjective enjoyment. The patient was the primary informant, although the CP was invited to supplement, clarify, or correct the information as appropriate. | | [111] |
| **11.6 Patient general physical and mental health (n=1)** | | | | |
| To examine the appropriateness and feasibility of cognitive rehabilitation for people with dementias associated with Parkinson's in a pilot randomised controlled study. It also assessed the usefulness of outcome measures and obtaining effect sizes to inform the development of future RCTs of cognitive rehabilitation in PDD and DLB. | | | Mixed methods trial. Secondary trial outcome measures completed by patients included the EQ-5D-3L assessing general health. | [34] |
| **11.7 Patient and care partner narrative comments on the symptom and illness experience (n=3)** | | | | |
| Non-structured narrative reflection from the perspective of a child on their experience of having a parent with DLB. | | | The author discusses difficulty in obtaining a diagnosis and experiences of symptoms. | [66] |
| To explore the subjective experience of living with DLB. | | | A qualitative study of in-depth interviews. Five males with DLB participated. There was no strict interview guide; however, prompts and questions were used to explore the patient’s illness experience. | [49] |
| To document the lived experience of spouse CPs of people with early-onset dementia. | | | Qualitative study. Face-to-face interviews were conducted with CPs, based on interviews developed by Clare et al. [46]. These explored experiences within the disease trajectory, including first symptoms, the quest for diagnosis, main difficulties encountered, and the evolution of relationships within the dyad and family. | [47] |
| **11.8 Patient and care partner perspectives on symptom changes during lockdown (n=4)** | | | | |
| To describe the lockdown’s impact amongst people affected by young onset, non-memory-led and inherited dementias and their CPs. | | | A mixed methods design was used. Data were collected via a bespoke web survey administered to PLWD and family CPs. The survey captured the impact of lockdown on cognitive symptoms and functioning. | [17] |
| To assess modifications of neuropsychiatric symptoms during quarantine in PLWD and their CPs. | | | Quantitative sub-study of a multicentre, nation-wide survey. A bespoke survey was administered to family CPs over the phone which queried variations in BPSD during the COVID-19 lockdown. | [19] |
| To examine changes in behaviour among PLWD and to look for associations between the evolution of BPSD and CPs’ mental health in the context of COVID-19. | | | Quantitative study. A web survey, which comprised both standardised instruments and novel questions, was completed by CPs. For DLB CPs, perspective data related to novel measures of changes or emergences of BPSD since lockdown. | [20] |
| To investigate clinical changes in patients with AD and other dementia subtypes, and to evaluate CPs' distress during the COVID-19 quarantine. | | | Quantitative study. A bespoke survey was administered to family CPs over the phone. Main outcomes were patients’ changes in cognitive, behavioural, and motor symptoms. | [18] |
| **12. Care partner physical and mental health (n=18) -** Captures the mental and physical health of CPs. This includes experiences of anxiety, depression, sleep disturbance, stress, and general health, as well as how these outcomes are influenced by caregiving demands, interventions, and broader contextual factors such as the COVID-19 pandemic. | | | | |
| Study aimed to (1) investigate the phenomenology of cognitive fluctuations in DLB by understanding CP experiences, (2) examine whether CPs considered sleep, or sleep disturbances, to influence the nature of cognitive fluctuations, and (3) understand the nature of habitual sleep patterns, sleep changes and sleep disturbances, and their impact on people with DLB and their CPs. | | | Qualitative study involving telephone interviews with CPs, based on a bespoke guide. Among other items, the interview queried the CP’s own sleep experiences. | [92] |
| To investigate the effects of the pandemic on anxiety, depression and care burden in CPs of nursing patients with AD, DLB and MCI over a one-year period, and to explore predictive factors, particularly physical activity, social contact and sleep disturbance, in relation to the worsening or improvement of these concerns. | | | Longitudinal, quantitative study. A one-year follow-up survey of patients and their CP was conducted to assess changes in CP anxiety and depression (GAD-7 and PHQ-9), as well as sleep quality (PSQI). Novel questions assessed physical activity. | [21] |
| To investigate clinical changes in patients with AD and other dementia subtypes, and to evaluate CPs' distress during the COVID-19 quarantine. | | | Quantitative study. A bespoke survey was administered to family CPs over the phone. Questions addressed CPs’ anxiety and depression. | [18] |
| To describe the lockdown’s impact amongst people affected by young onset, non-memory-led and inherited dementias and their CPs. | | | A mixed methods design was used. Data were collected via a bespoke web survey administered to PLWD and family CPs. Survey captured data on CP health during lockdown. | [17] |
| To explore and compare levels of mental health, care burden, and relationship satisfaction among caregiving spouses of people with PD-MCI, PDD or DLB. | | | Quantitative study. Results reported from a battery of rating scales completed by CPs including the SF-12 which assessed mental and physical health, and the HADS (assessing anxiety and depression). | [44] |
| To assess psychometric properties and describe clinimetric properties of PROMs completed by informant CPs of people with PD-MCI, PDD, or DLB to gain insight into the suitability, reliability, and usability of the scales in this population | | | Quantitative study. Data were extracted from the INVEST trial, including CP-reported HADS (assessing anxiety and depression) as well as assessments of CP physical and mental health (SF-12). | [45] |
| To investigate CP burden, sleep quality, and stress in DLB, FTLD and AD CPs. | | | Quantitative study. CPs were compared according to the PHQ-9 and GAD-9 assessing depression and anxiety, and with the PSQI assessing sleep quality. | [112] |
| To review clinical characteristics of DLB with Capgras syndrome compared to those without and its potential impact on CPs. | | | Quantitative study. CPs were assessed for depression using the PHQ-9. | [113] |
| To explore differences in CP self-efficacy and their associated factors between DLB and AD CPs. | | | Quantitative study. CPs completed a series of instruments including the BDI-II which assessed CP depression and the ISI to assess insomnia. | [38] |
| To explore the impact of a home-based, individualised cognitive stimulation therapy adapted for a population of people with PD-MCI, PDD or DLB. They also evaluated the acceptability of the intervention and the feasibility of conducting a full-scale RCT. | | | Mixed methods trial design. Exploratory trial outcomes for CPs included the HADS which assessed CP self-rated anxiety and depression, and the SF-12 assessing general physical and mental health. | [23] |
| To examine the appropriateness and feasibility of cognitive rehabilitation for people with dementias associated with Parkinson's in a pilot randomised controlled study. It also assessed the usefulness of outcome measures and obtaining effect sizes to inform the development of future RCTs of cognitive rehabilitation in PDD and DLB. | | | Mixed methods trial. Secondary trial outcome measures completed by CPs included the HADS to assess CP self-reported anxiety and depression, and the EQ-5D-3L to assess general health. | [34] |
| To assess the feasibility of undertaking a cluster randomised study of the introduction of an evidence-based management toolkit for LBD, assessing the outcomes for patients and CPs as secondary measures. | | | Pragmatic cluster RCT trial. Among the outcomes, the HADS assessed CP anxiety and depression | [10] |
| To assess the feasibility of a peer mentor-led educational intervention and its impact on LBD family CPs' knowledge, dementia attitudes, and mastery. | | | Mixed methods trial. The HADS (assessing CP anxiety and depression) and GDS-15 (assessing depression) were included as a pre- and post measures | [25] |
| To examine the feasibility and acceptability of a psychosocial intervention for DLB designed to build coping capability. A secondary objective was to identify the most appropriate primary outcome measure to inform a future multi-centre evaluation study. | | | Mixed methods trial. Pre- and post-outcome measures for CPs included the GDS-15 which measured depression. | [14] |
| To examine the differences in CP experiences of grief, burden, and QoL between DLB, PDD, and AD. | | | Quantitative study. Data were collected using a web survey which included the PHQ-2 to measure CP depression. | [39] |
| To identify factors associated with depressive symptoms in family CPs of people with DLB, AD, and PDD. It compared the burden, grief, and depressive symptoms of CPs across conditions. | | | Quantitative study. Data were collected using a web survey which was completed by CPs. The survey included the PHQ-2 to measure CP depression. | [40] |
| To examine differences in the experiences of spouse versus adult child CPs of individuals with DLB. | | | Quantitative study. Data were collected using a web survey that included the PHQ-2 to measure CP depression. | [41] |
| To examine factors associated with pre-loss grief in CPs of older adults with DLB to determine whether overall CP experiences differ based on the stages of DLB in care recipients. The study also compared pre-loss grief in DLB CPs with that in AD CPs and other dementias. | | | Quantitative study. Data were collected using a web survey which included the PHQ-2 to measure CP depression. | [114] |
| **13. Emotional and psychological well-being (n=64) -** Captures the emotional and psychological well-being of both people with DLB and their CPs. Sub-topics include: (1) CP burden, stress, and/or distress, (2) CP experience of grief, (3) patient and CP psychological well-being, (4) patient positive affect, and (5) patient empathy. | | | | |
| **13.1 Care partner burden, stress, and/or distress (n=61)** | | | | |
| To investigate the effects of the pandemic on anxiety, depression and care burden in CPs of nursing patients with AD, DLB and MCI over a one-year period, and to explore predictive factors, particularly physical activity, social contact and sleep disturbance, in relation to the worsening or improvement of these concerns. | | Longitudinal, quantitative study. A one-year follow-up survey of patients and their CP included an assessment of CP burden using the ZBI. | | [21] |
| To assess modifications of neuropsychiatric symptoms during quarantine in PLWD and their CPs. | | Quantitative sub-study of a multicentre, nation-wide survey. A bespoke survey was administered to family CPs over the phone which queried variations in BPSD. Associations with disease type, severity, gender, and CP’s stress burden were analysed. | | [19] |
| To assess the satisfaction of DLB patients, CPs (and their attending physicians) with medication for five DLB symptom domains. | | This was a sub-analysis of a cross-sectional, bespoke questionnaire-based survey study. CPs were screened using the ZBI. | | [67] |
| To correlate neuropsychiatric and motor assessments with language and visual organization tests in LBD. | | A quantitative approach was used. CPs were assessed using the ZBI to explore characterise differences in CP burden between DLB and PDD. | | [93] |
| To assess risk factors for age at dementia onset, as well as neuropsychiatric features that are associated with pharmacotherapy and signs and symptoms of LBD. | | Quantitative study. CPs were interviewed using the ZBI (assessing burden) and NPI-D (assessing distress). | | [94] |
| To compare and distinguish demographic and neuropsychiatric features between LBD and APOE-ε3/ε3 late-onset AD. | | Quantitative study. CPs were interviewed using the ZBI to assess CP burden. | | [95] |
| To investigate CP burden, sleep quality, and stress in DLB, FTLD and AD CPs. | | Quantitative study. CPs were compared according to the ZBI. | | [112] |
| To examine the treatment needs of patients with DLB and their CPs and the extent to which the attending physicians understand these needs. | | Quantitative study. CPs completed screening tests including the ZBI. | | [71] |
| To explore the factor structure of the ZBI in life partners of people with Parkinson-related dementia and to examine the relationships among emerging factors and the demographic and clinical features. | | Quantitative study. Life partners of people with Parkinson-related dementia participated in home-based quantitative assessments and self-completed postal questionnaires. The assessment battery included the ZBI. | | [115] |
| To evaluate the current economic costs of DLB and its related factors. | | Quantitative study. Patients' per capita annual economic costs related to DLB in the year preceding the interview were evaluated, and factors related to the costs were assessed using regression analysis. CPs completed the ZBI to explore burden as a factor related to DLB. | | [11] |
| To investigate tau in people with DLB using a second-generation tau positron emission tomography tracer as well as plasma p-tau181. | | Quantitative study. CP burden was assessed using the ZBI during clinical assessments. | | [85] |
| To investigate the clinical and imaging correlates of amyloid-beta deposition in DLB. | | Quantitative study. Study partners completed the ZBI during clinical assessments. | | [86] |
| Quantitative study aiming to classify BPSD and to reveal what type of BPSD affected CP burden in patients with DLB. | | CPs completed the ZBI. | | [116] |
| To elucidate the influence of dementia etiologies on the degree of CP burden and determine which factors predict a high CP burden. | | A longitudinal (18-month), quantitative, retrospective cohort study was conducted. The ZBI was completed to assess CP burden. | | [117] |
| To explore associations of cerebrospinal fluid biomarkers of neurodegeneration and amyloidosis with CP burden, cognition, and functionality in DLB paired with late-onset AD and healthy older people. | | Quantitative study. The ZBI assessed CP burden. | | [118] |
| To examine clinical characteristics, cognitive decline, and predictors for time to dementia in MCI-LB compared to MCI-AD. | | A quantitative, longitudinal cohort study was conducted. The ZBI was used to compare CP burden between groups. | | [119] |
| To review clinical characteristics of DLB with Capgras syndrome compared to those without and its potential impact on CPs. | | Quantitative study. CPs completed the ZBI to measure CP burden. | | [113] |
| To investigate the prevalence and types of neuropsychiatric symptoms in dementia patients presenting to a Tertiary Care Hospital. It also explored the association between neuropsychiatric symptoms and the type of dementia, as well as assessing the relationship between these symptoms and CP stress in this setting. | | Quantitative study. CPs completed the ZBI. | | [120] |
| To compare CP burden of AD, DLB and behavioural variant FTD, paying attention to positive affect of patients. | | Quantitative study. CPs completed the ZBI. | | [121] |
| To compare levels of CP burden and the possible influential factors on the CP burden between DLB and AD. | | Quantitative study. CPs completed the ZBI. | | [122] |
| To identify factors that may be associated with burden among male CPs of PLWD. | | Quantitative study. CPs complete the ZBI. | | [123] |
| To evaluate the effects of BPSD on CP burden in these three types of dementia. | | Quantitative study. Burden was measured using the ZBI | | [124] |
| To investigate the associations between core and suggestive DLB symptoms and different aspects of disease burden, i.e., instrumental activities of daily living, QoL and CP burden. | | Quantitative, longitudinal study. CP burden was assessed using the ZBI. | | [78] |
| To identify associations with rate of change in cognition, everyday functioning, and QoL. | | Quantitative, longitudinal cohort study. Annual assessments included administration of the ZBI. | | [84] |
| To explore differences in CP self-efficacy and their associated factors between DLB and AD CPs. | | Quantitative study. CPs completed a series of instruments including the NPI-D (assessing CP distress) and the ZBI (assessing CP burden). | | [38] |
| To examine the specific impacts of dementia subtype diagnosis and neuropsychiatric symptoms on the stress of AD, VaD, DLB and PDD CPs. | | Quantitative study. CP were interviewed using the NPI-D to assess distress. | | [125] |
| To assess the frequency of CP- and patient-reported anxiety and associations between anxiety and socio-demographic and clinical variables in individuals with first-time diagnosed mild dementia. | | Quantitative study. Symptoms of anxiety were rated by a CP on the NPI, which included an assessment of CP distress related to the symptoms. The Rel.SS was used to measure CP stress. | | [126] |
| To assessing neuropsychiatric disorders, functional abilities, and CP distress in DLB and in AD subjects. | | Quantitative study. CPs completed the NPI-D. | | [127] |
| To investigate associated factors of delusions in patients with DLB. | | A quantitative retrospective study of outpatients with DLB registered in a regional hospital’s database was performed. All patients and their main CPs were interviewed using the NPI, and all 12 NPI domains were rated for CP burden. | | [128] |
| To explore and compare levels of mental health, care burden, and relationship satisfaction among caregiving spouses of people with PD-MCI, PDD or DLB. | | Quantitative study. CPs completed a battery of rating scales, including the Rel.SS (assessing care-related stress), the ZBI (assessing CP burden), and the NPI assessing CP distress. | | [44] |
| To assess psychometric properties and describe clinimetric properties of PROMs completed by informant CPs of people with PD-MCI, PDD, or DLB to gain insight into the suitability, reliability, and usability of the scales in this population and to help future researchers and clinicians with decision-making regarding appropriate instruments. | | Quantitative data were extracted from the INVEST trial. CP scores for the the ZBI and Rel.SS were reported. | | [45] |
| To investigate multivariate associations between individual neuropsychiatric symptoms in people with mild dementia living at home and CP distress. It also aimed to explore whether there are differences in the way individual neuropsychiatric symptoms are associated with CP distress in relation to diagnosis (AD vs DLB). | | Quantitative study. The Rel.SS was used to assess the level of reported distress in CPs. | | [129] |
| To characterise the differences in CP distress between DLB and AD CPs, with a view of differentiating and improving support for CPs. | | Quantitative study. CP distress was measured using the Rel.SS. | | [130] |
| To assess the level of CP reported distress in mild dementia over a three-year period. | | Longitudinal quantitative study. The Rel.SS was used to track patterns of CP distress. | | [131] |
| To examine time to nursing home admission in mild dementia and predictors for nursing home admission in people with DLB and how it compares to AD. | | A longitudinal quantitative study. CP distress, investigated as a predictor of time until nursing home admission, was measured using the Rel.SS. | | [132] |
| To examine the differences in CP experiences of grief, burden, and QoL between DLB, PDD, and AD. | | Quantitative study. Data were collected via a web survey which included the ZBI to measure CP burden. | | [39] |
| To identify factors associated with depressive symptoms in family CPs of people with DLB, AD, and PDD. The study compared the burden, grief, and depressive symptoms of CPs across these conditions. It also examined the role of CPs' self-efficacy and received social support in improving psychological well-being and investigated whether self-efficacy mediates the relationship between CP burden and psychological well-being, as well as between CP grief and psychological well-being. | | Quantitative study. Data were collected via a web survey completed by CPs. The survey included the ZBI to measure CP burden. | | [40] |
| To examine differences in the CP experiences of spousal versus adult child CPs in individuals with DLB. | | Quantitative study. Data collected via a web survey which included the ZBI to measure burden across CP groups. | | [41] |
| To ascertain the unmet needs of LBD CPs and collect data to inform educational programming and enhance CP support. | | Quantitative study. Data were collected via a web survey which included the ZBI to measure CP burden. | | [59] |
| To examine the dimensions of subjective burden of CPs of patients with LBD using data from a quantitative online survey. | | Data collected via a quantitative web survey. Respondents were 611 current LBD CPs. The survey included the ZBI as the dependent measure. | | [42] |
| To examine factors associated with pre-loss grief in CPs of older adults with DLB and determine whether overall CP experiences differ based on the stages of DLB in care recipients. The study also compared pre-loss grief in DLB CPs with that in AD CPs and other dementias. | | Quantitative study. Using a cross-sectional design, 714 CPs of older adults with dementia completed a web survey on pre-loss grief. Multivariate linear regression identified risk factors associated with pre-loss grief and analysis of variance examined whether pre-loss grief in CPs differed significantly based on type of dementia or stage of DLB. The ZBI was used to measure CP burden. | | [114] |
| To identify care navigator approaches used to address CP burden in dementia as part of a dementia care navigation programme. A qualitative design is used. | | Methods of data collection were interviews, focus groups, observations, and qualitative case study analysis which captured perspectives of formal and informal CPs. | | [26] |
| To describe pharmacological treatment outcomes in patients with DLB or LBD. | | Quantitative case reports. Outcomes included the NPI-D (assessing CP distress) [68,133] and the ZBI (assessing CP burden) [134–137]. | | [68,133–137] |
| Trials of pharmacological treatment in patients with DLB included measures capturing CP perspectives as outcomes. | | Trials. Measures included the NPI-D (assessing CP distress) [138] and ZBI (assessing CP burden) [81,139–143]. In two sources, no formal primary endpoint was predefined [139,143]. In three sources, the instruments assessed primary outcomes [138,141,142], and in two, they assessed secondary outcomes [81,140]. | | [81,138–143] |
| To explore the impact of a home-based, individualised cognitive stimulation therapy adapted for a population of people with PD-MCI, PDD or DLB. They also evaluated the acceptability of the intervention and the feasibility of conducting a full-scale RCT. | | A mixed methods trial design was used. Exploratory outcomes for CPs included the ZBI (assessing CP burden), the NPI-D (assessing CP distress), and the Rel.SS (assessing stress related to care provision). | | [23] |
| To examine the appropriateness and feasibility of cognitive rehabilitation for people with dementias associated with Parkinson's in a pilot randomised controlled study. Additional aims included assessing the usefulness of outcome measures and obtaining effect sizes to inform the development of future RCTs of cognitive rehabilitation in PDD and DLB. | | Mixed methods trial. Secondary trial outcome measures completed by CPs included the NPI-D (assessing CP distress) and the Rel.SS (assessing stress related to care provision). | | [34] |
| To evaluate the general safety and tolerability of a nucleus basalis of Meynert (NBM) deep brain stimulation (DBS) procedure in DLB patients, and to compare scores on a battery of standardised cognitive, psychiatric, and motor tests pre-operatively and repeated after six weeks of bilateral NBM DBS and six weeks of sham deep brain stimulation. | | Trial endpoints included CP distress measured using the NPI-D. | | [80] |
| To assess the feasibility of a peer mentor-led educational intervention and its impact on LBD family CPs' knowledge, dementia attitudes, and mastery. | | Mixed methods trial design. The ZBI was included as a pre- and post outcome measure. | | [25] |
| To assess the feasibility of undertaking a cluster randomised study of the introduction of an evidence-based management toolkit for LBD, assessing the outcomes for patients and carers as secondary measures. | | A pragmatic cluster RCT trial. Among the outcomes, the ZBI was included to assess CP burden. | | [10] |
| To examine the feasibility and acceptability of a psychosocial intervention for DLB designed to build coping capability. A secondary objective was to identify the most appropriate primary outcome measure to inform a future multi-centre evaluation study. | | Mixed methods trial design. CPs completed the PSS (assessing perceived stress) and the Rel.SS (assessing stress) as pre- and post-outcome measures. | | [14] |
| **13.2 Care partner grief (n=4)** | | | | |
| To examine the differences in CP experiences of grief, burden, and QoL between DLB, PDD, and AD. | | Quantitative study. CPs completed a 230-question web survey which included the MM-CGI-SF to measure CP grief. | | [39] |
| To identify factors associated with depressive symptoms in family CPs of people with DLB, AD, and PDD. It compared the burden, grief, and depressive symptoms of CPs across these conditions. Additionally, it examined the role of CPs' self-efficacy and received social support in improving psychological well-being and investigated whether self-efficacy mediates the relationship between CP burden and psychological well-being, as well as between CP grief and psychological well-being. | | Quantitative study. Data were collected through a web survey for family CPs of individuals with DLB. The survey included the MM-CGI-SF to assess CP grief. | | [40] |
| To examine differences in the caregiving experiences of spouses versus adult child CPs in individuals with DLB. | | Quantitative study. Spouses and adult children CPs of individuals with DLB completed a web survey which included the MM-CGI-SF to measure CP grief. | | [41] |
| To examine factors associated with pre-loss grief in CPs of older adults with DLB to determine whether overall CP experiences differ based on the stages of DLB in care recipients. The study also compared pre-loss grief in DLB CPs with that in AD CPs and other dementias. | | Quantitative study. CPs of older adults with dementia completed a web survey on pre-loss grief. Multivariate linear regression identified risk factors associated with pre-loss grief and analysis of variance examined whether pre-loss grief in CPs differed significantly based on type of dementia or stage of DLB. The survey included the PG-12 together with novel questions to characterise pre-loss grief. | | [114] |
| **13.3 Patient and care partner psychological well-being (n=7)** | | | | |
| To describe the lockdown’s impact amongst people affected by young onset, non-memory-led and inherited dementias and their CPs. | | A mixed methods design was used. Data were collected via a bespoke web survey administered to PLWD and family CPs. Survey captured data on the impact of lockdown on the patient’s psychological well-being. | | [17] |
| To explore the subjective experience of living with DLB. | | Qualitative study. In-depth interviews were conducted and analysed using interpretative phenomenological analysis. Prompts and questions explored the patient’s illness experience and factors affecting well-being, with participants guiding the interviews toward personally significant topics. | | [49] |
| To investigate the potential impact of dementia subtypes on the capability to live well for both PLWD and their CPs. | | A sub-study of a mixed-methods, five-year longitudinal cohort study. The analysis was based on 1283 dyads of community-dwelling people with dementia and CPs in the Improving the experience of Dementia and Enhancing Active Life (IDEAL) project. ‘Capability to live well’ was defined using three measures, one of which was well-being, assessed for CPs and PLWD using the WHO-5. | | [82] |
| To examine the differences in CP experiences of grief, burden, and QoL between DLB, PDD, and AD. | | Quantitative study. Data were collected via a web survey for CPs which included the C-PWBS (assessing general psychological well-being) and the PCI (measuring appraisals of self-improvement or decline in distinct areas of well-being related to caregiving). | | [39] |
| To identify factors associated with depressive symptoms in family CPs of people with DLB, AD, and PDD. It compared the burden, grief, and depressive symptoms of CPs across these conditions. It also examined the role of CPs' self-efficacy and received social support in improving psychological well-being and investigated whether self-efficacy mediates the relationship between CP burden and psychological well-being, as well as between CP grief and psychological well-being. | | Quantitative study. Data were collected using a web survey which was completed by CPs. The survey included the C-PWBS to measure psychological well-being. | | [40] |
| To examine factors associated with pre-loss grief in CPs of older adults with DLB to determine whether overall CP experiences differ based on the stages of DLB in care recipients. Also compared pre-loss grief in DLB CPs with that in AD CPs and other dementias. | | Quantitative study. Data were collected using a web survey which included the C-PWBS and PCI to measure CP psychological well-being and improvement or decline in well-being related to being a caregiver. | | [114] |
| To examine differences in the experiences of spouse versus adult child CPs of individuals with DLB. | | Quantitative study. Data were collected using a web survey for CPs. The survey included the C-PWBS to assess psychological well-being. | | [41] |
| **13.4 Patient positive affect (n=1)** | | | | |
| To compare CP burden in AD, DLB and bvFTD, paying attention to differences in the positive affect of patients. | | Quantitative study of CPs. Standardised assessments were administered during clinical visits, including the QOL-D which measured CP-reported patient affect. | | [121] |
| **13.5 Patient empathy (n=1)** | | | | |
| To explore the impact of a home-based, individualised cognitive stimulation therapy adapted for a population of people with PD-MCI, PDD, or DLB. They also evaluated the acceptability of the intervention and the feasibility of conducting a full-scale RCT. | | Mixed methods trial design. Exploratory trial outcomes for people with PD-MCI/PDD/DLB included the IRI which assessed empathy. | | [23] |
| *Note:* Sources could be charted across multiple topics. Bespoke survey is a survey containing only novel, investigator-generated questions (i.e., non-standardised measures)*.* ZBI and NPI included all versions.  **Abbreviations:**   A. **Standardised measures:** Conversation Analysis Profile for People with Cognitive Impairment (CAPPCI); Resource Utilization in Dementia - Lite Version (RUD-Lite); Brief Pain Questionnaire (BPI); Blessed Dementia Scale (BDS); The Music in Dementia Assessment Scale (MiDAS); Client Services Receipt Inventory (CSRI); Scales for Outcomes in PD-Autonomic (SCOPA-Aut); Satisfaction with Life Scale (SwLS); The WHO-5 Well-being Index (WHO-5); Dementia Attitudes Scale (DAS); Pearlin Mastery Scale (PMS); UCLA Loneliness Scale (UCLA-LS); Revised UCLA Loneliness Scale (R-UCLA-LS); The Prolonged Grief-12 (PG-12); Family Care Partner Self-Efficacy for Managing Dementia Scale (FCP-SEMD Scale); Geriatric Depression Scale (GDS-15); Perceived Stress Scale (PSS); Condensed version of the Psychological Well-Being Scale (C-PWBS); The Interpersonal Reactivity Index (IRI); Parkinson's Disease Questionnaire―8 (PDQ‐8); Linear Analogue Self-Assessment Scale (LASA); The Alzheimer Disease-related Quality of Life (ADRQL); Dementia Quality of Life Measure (DEMQOL); Euroqol Questionnaire‐short version (ED5D3L); The Desire to Institutionalize Scale (DIS); Insomnia Severity Index (ISI); Social Support Questionnaire (SSQ); Subjective Time Questionnaire (STQ); The Attitude Toward Own Aging questionnaire (ATOA); NEO Five Factor Inventory (NEO-FFI); The Medical Outcome Study Social Support (MOS-SS); Perceived Change Index (PCI); 39-item Parkinson's Disease Questionnaire (PDQ-39); Beck Depression Inventory-II (BDI-II); Quality of life questionnaire for dementia (QOL-D); General Anxiety Disorder-7 (GAD-7); The Pittsburgh Sleep Quality Index (PSQI); Short-Form-8 (SF-8); World Health Organization Quality of Life-Brief Version (WHOQOL-BREF); Generalized Self-Efficacy Scale (GSES); The Bangor Goal Setting Interview (BGSI); Euro-Qol 5 dimensions 5-level (EQ-5D-5L); The North-East Visual Hallucination Interview (NEVHI); Short Form Health Survey, version 2 (SF-12-v2); Symptom Checklist 90R (SCL-90-R); The Marwit-Meuser Caregiver Grief Inventory Short Form (MM-CGI-SF); Patient Health Questionnaire-9 (PHQ-9); 8-item short version of the Japanese Zarit Burden Interview (J-ZBI_8); Revised Scale for Caregiving Self-Efficacy (RSCSE); The Relationship Satisfaction Scale (RSS); The 12-item short-form health survey (SF-12); The Dyadic Relationship Scale (DRS); The Family Caregiving Role Scale (FCR); The Brief Resilience Scale (BRS); Patient Health Questionnaire-2 (PHQ-2); Hospital Anxiety and Depression Scale (HADS); 12-item Zarit Burden Interview (ZBI-12); EuroQol- 5 Dimension (EQ-5D); Quality of Life in Alzheimer’s Disease (QoL-AD); Relative’s Stress Scale (Rel.SS); Neuropsychiatric Inventory carer distress scale (NPI-D); The Zarit Burden Interview (ZBI) B. **Other:** Care partner (CP); Dementia with Lewy bodies (DLB); Lewy body dementia (LBD); Frontotemporal dementia (FTD); behavioural-variant frontotemporal dementia (bvFTD); Alzheimer’s disease (AD); Parkinson’s disease (PD); Frontotemporal lobar degeneration (FTLD); Mild cognitive impairment in Parkinson’s (PD-MCI); Parkinson’s disease dementia (PDD); Mild cognitive impairment (MCI); Multiple system atrophy (MSA); Progressive supranuclear palsy (PSP); Corticobasal syndrome (CBS): Vascular dementia (VaD); Subjective cognitive impairment (SCI); People living with dementia (PLWD); Behavioural and psychological symptoms of dementia (BPSD); Somatoform disorders (SFMD); QoL (Quality of life); Health-related quality of life (HRQoL); Randomised controlled trial (RCT); The Program Participation Questionnaire (PPQ) | | | | |

**References**

1. Armstrong MJ, Gamez N, Alliance S, Majid T, Taylor A, Kurasz AM, et al. Research priorities of caregivers and individuals with dementia with Lewy bodies: an interview study. PLoS ONE. 2020;15:e0239279. https://doi.org/10.1371/journal.pone.0239279

2. Holden SK, Bedenfield N, Taylor AS, Bayram E, Schwilk C, Fleisher J, et al. Research priorities of individuals and caregivers with Lewy body dementia: a web-based survey. Alzheimer Dis Assoc Disord. 2023;37:50–8. https://doi.org/10.1097/WAD.0000000000000545

3. Taylor A., Corsentino P., Peterson B., Richard I., Long A., Leverenz J.B. P4-067: The importance of educating the lewy body dementia community on risks and benefits of lumbar punctures in LBD biomarker research. Alzheimers Dement. 2018;14:1458. https://doi.org/10.1016/j.jalz.2018.06.2470

4. Armstrong MJ, Alliance S, Corsentino P, Lunde A, Taylor A. Informal caregiver experiences at the end-of-life of individuals living with dementia with Lewy bodies: an interview study. Dementia (London). 2022;21:287–303. https://doi.org/10.1177/14713012211038428

5. Armstrong MJ, Alliance S, Taylor A, Corsentino P, Galvin JE. End-of-life experiences in dementia with Lewy bodies: Qualitative interviews with former caregivers. PLoS One. 2019;14:e0217039. https://doi.org/10.1371/journal.pone.0217039

6. Armstrong MJ, Alliance S, Corsentino P, Maixner SM, Paulson HL, Taylor A. Caregiver-reported barriers to quality end-of-life care in dementia with Lewy bodies: a qualitative analysis. Am J Hosp Palliat Care. 2020;37:728–37. https://doi.org/10.1177/1049909119897241

7. Armstrong MJ, Alliance S, Corsentino P, DeKosky ST, Taylor A. Cause of death and end-of-life experiences in individuals with dementia with Lewy bodies. J Am Geriatr Soc. 2019;67:67–73. https://doi.org/10.1111/jgs.15608

8. Henderson C., Knapp M., Martyr A., Gamble L.D., Nelis S.M., Quinn C., et al. The use and costs of paid and unpaid care for people with dementia: longitudinal findings from the IDEAL cohort. J Alzheimer’s Dis. 2022;86:135–53. https://doi.org/10.3233/JAD-215117

9. Boström F, Jönsson L, Minthon L, Londos E. Patients with Lewy body dementia use more resources than those with Alzheimer’s disease. Int J Geriatr Psychiatry. 2007;22:713–9. https://doi.org/10.1002/gps.1738

10. O’Brien JT, McKeith IG, Thomas AJ, Bamford C, Vale L, Hill S, et al. Introduction of a management toolkit for Lewy body dementia: a pilot cluster‐randomized trial. Mov Disord. 2021;36:143–51. https://doi.org/10.1002/mds.28282

11. Guo X., Meng Q., Zuo M., Su Z., Gan J., Li X.-D., et al. Costs of dementia with lewy bodies: A Chinese multicenter cross-sectional study. Int J Geriatr Psychiatry. 2023;38:e5848. https://doi.org/10.1002/gps.5848

12. Thomas AJ, Taylor JP, McKeith I, Bamford C, Burn D, Allan L, et al. Development of assessment toolkits for improving the diagnosis of the Lewy body dementias: feasibility study within the DIAMOND Lewy study. Int J Geriatr Psychiatry. 2017;32:1280–304. https://doi.org/10.1002/gps.4609

13. O’Brien JT, Taylor J-P, Thomas A, Bamford C, Vale L, Hill S, et al. Improving the diagnosis and management of Lewy body dementia: the DIAMOND-Lewy research programme including pilot cluster RCT. Programme Grants Appl Res. 2021;9:1–120. https://doi.org/10.3310/pgfar09070

14. Killen A., Flynn D., O’Brien N., Taylor J.-P. The feasibility and acceptability of a psychosocial intervention to support people with dementia with Lewy bodies and family care partners. Dementia (London). 2022;21:77–93. https://doi.org/10.1177/14713012211028501

15. Stacy KE, Lambert J, Shatz R, Bakas T. Development and validation of the Lewy body disease caregiver activities scale. J Nurs Meas. 2023;31:606–14. https://doi.org/10.1891/JNM-2021-0100

16. Thomas P, Lalloué F, Preux P, Hazif‐Thomas C, Pariel S, Inscale R, et al. Dementia patients caregivers quality of life: the PIXEL study. Int J Geriatr Psychiatry. 2006;21:50–6. https://doi.org/10.1002/gps.1422

17. Harding E, Rossi-Harries S, Gerritzen EV, Zimmerman N, Hoare Z, Proctor D, et al. ‘I felt like I had been put on the shelf and forgotten about’ - lasting lessons about the impact of COVID-19 on people affected by rarer dementias. BMC Geriatr. 2023;23:392. https://doi.org/10.1186/s12877-023-03992-1

18. Rainero I, Bruni AC, Marra C, Cagnin A, Bonanni L, Cupidi C, et al. The impact of COVID-19 quarantine on patients with dementia and family caregivers: a nation-wide survey. Front Aging Neurosci. 2021;12:625781. https://doi.org/10.3389/fnagi.2020.625781

19. Cagnin A, Di Lorenzo R, Marra C, Bonanni L, Cupidi C, Laganà V, et al. Behavioral and psychological effects of coronavirus disease-19 quarantine in patients with dementia. Front Psychiatry. 2020;11:578015. https://doi.org/10.3389/fpsyt.2020.578015

20. Pongan E., Dorey J.-M., Borg C., Getenet J.C., Bachelet R., Lourioux C., et al. COVID-19: Association between increase of behavioral and psychological symptoms of dementia during lockdown and caregivers’ poor mental health. J Alzheimer’s Dis. 2021;80:1713–21. https://doi.org/10.3233/JAD-201396

21. Bao X., Xu J., Meng Q., Gan J., Wang X.-D., Wu H., et al. Impact of the COVID-19 pandemic and lockdown on anxiety, depression and nursing burden of caregivers in Alzheimer’s disease, dementia with Lewy bodies and mild cognitive impairment in China: a 1-year follow-up study. Front Psychiatry. 2022;13:921535. https://doi.org/10.3389/fpsyt.2022.921535

22. Torboli D, Mioni G, Bussé C, Cagnin A, Vallesi A. Subjective experience of time in dementia with Lewy bodies during COVID-19 lockdown. Curr Psychol. 2023;42:4653–62. https://doi.org/10.1007/s12144-021-01811-7

23. Leroi I, Vatter S, Carter L-A, Smith SJ, Orgeta V, Poliakoff E, et al. Parkinson’s-adapted cognitive stimulation therapy: a pilot randomized controlled clinical trial. Ther Adv Neurol Disord. 2019;12:1756286419852217. https://doi.org/10.1177/1756286419852217

24. Heyman I, Brorsson A, Persson T, Londos E. Pacemaker implants and their influence on the daily life of patients with dementia with Lewy bodies: A qualitative case study. Neurol Ther. 2023;12:1359–73. https://doi.org/10.1007/s40120-023-00513-5

25. Fleisher J, Suresh M, Levin M, Hess S, Akram F, Dodson D, et al. Learning to PERSEVERE: a pilot study of peer mentor support and caregiver education in Lewy body dementia. Parkinsonism Relat Disord. 2023;113:105492. https://doi.org/10.1016/j.parkreldis.2023.105492

26. Bernstein A., Merrilees J., Dulaney S., Harrison K.L., Chiong W., Ong P., et al. Using care navigation to address caregiver burden in dementia: a qualitative case study analysis. Alzheimers Dement (N Y). 2020;6:e12010. https://doi.org/10.1002/trc2.12010

27. Park J, Howard H, Tolea MI, Galvin JE. Perceived benefits of using nonpharmacological interventions in older adults with Alzheimer’s disease or dementia with Lewy bodies. J Gerontol Nurs. 2020;46:37–46. https://doi.org/10.3928/00989134-20191217-01

28. Garrido S., Dunne L., Stevens C.J., Chang E., Clements-Cortes A. Music playlists for people with dementia: trialing a guide for caregivers. J Alzheimer’s Dis. 2020;77:219–26. https://doi.org/10.3233/JAD-200457

29. McCormick SA, Vatter S, Carter L-A, Smith SJ, Orgeta V, Poliakoff E, et al. Parkinson’s-adapted cognitive stimulation therapy: feasibility and acceptability in Lewy body spectrum disorders. J Neurol. 2019;266:1756–70. https://doi.org/10.1007/s00415-019-09329-6

30. Mackenzie L., James I.A., Morse R., Mukaetova-Ladinska E., Reichelt F.K. A pilot study on the use of dolls for people with dementia. Age Ageing. 2006;35:441–4. https://doi.org/10.1093/ageing/afl007

31. Boots LM, De Vugt ME, Withagen HE, Kempen GI, Verhey FR. Development and Initial Evaluation of the Web-Based Self-Management Program “Partner in Balance” for Family Caregivers of People With Early Stage Dementia: An Exploratory Mixed-Methods Study. JMIR Res Protoc. 2016;5:e33. https://doi.org/10.2196/resprot.5142

32. Bruinsma J., Peetoom K., Bakker C., Boots L., Millenaar J., Verhey F., et al. Tailoring and evaluating the web-based ‘Partner in Balance’ intervention for family caregivers of persons with young-onset dementia. Internet Interv. 2021;25:100390. https://doi.org/10.1016/j.invent.2021.100390

33. Watermeyer T, Hindle J, Roberts J, Lawrence C, Martyr A, Lloyd-Williams H, et al. Goal setting for cognitive rehabilitation in mild to moderate Parkinson’s disease dementia and dementia with Lewy bodies. Parkinsons Dis. 2016;2016:8285041. https://doi.org/10.1155/2016/8285041

34. Hindle JV, Watermeyer TJ, Roberts J, Brand A, Hoare Z, Martyr A, et al. Goal‐orientated cognitive rehabilitation for dementias associated with Parkinson’s disease―a pilot randomised controlled trial. Int J Geriatr Psychiatry. 2018;33:718–28. https://doi.org/10.1002/gps.4845

35. Whitworth A, Lesser R, McKeith I. Profiling conversation in Parkinson’s disease with cognitive impairment. Aphasiology. 1999;13:407–25. https://doi.org/10.1080/026870399402154

36. Lindeberg S., Muller N., Samuelsson C. Conversations in dementia with Lewy bodies: Resources and barriers in communication. Int J Lang Commun Disord. 2023;58:419–32. https://doi.org/10.1111/1460-6984.12799

37. Clasper K, Savitch N. Raising awareness and mutual support: using the internet. J Dement Care. 2011;19:30–2.

38. Sato H, Nakaaki S, Sato J, Shikimoto R, Furukawa TA, Mimura M, et al. Caregiver self-efficacy and associated factors among caregivers of patients with dementia with lewy bodies and caregivers of patients with alzheimer’s disease. Psychogeriatrics. 2021;21:783–94. https://doi.org/10.1111/psyg.12740

39. Rigby T, Johnson DK, Taylor A, Galvin JE. Comparison of the caregiving experience of grief, burden, and quality of life in dementia with Lewy bodies, Alzheimer’s disease, and Parkinson’s disease dementia. J Alzheimer’s Dis. 2021;80:421–32. https://doi.org/10.3233/JAD-201326

40. Park J, Tolea MI, Arcay V, Lopes Y, Galvin JE. Self-efficacy and social support for psychological well-being of family caregivers of care recipients with dementia with Lewy bodies, Parkinson’s disease dementia, or Alzheimer’s disease. Soc Work Ment Health. 2019;17:253–78. https://doi.org/10.1080/15332985.2018.1526756

41. Rigby T, Ashwill RT, Johnson DK, Galvin JE. Differences in the experience of caregiving between spouse and adult child caregivers in dementia with Lewy bodies. Innov Aging. 2019;3:igz027. https://doi.org/10.1093/geroni/igz027

42. Leggett AN, Zarit S, Taylor A, Galvin JE. Stress and burden among caregivers of patients with Lewy body dementia. Gerontologist. 2011;51:76–85. https://doi.org/10.1093/geront/gnq055

43. Vatter S, McDonald KR, Stanmore E, Clare L, McCormick SA, Leroi I. A qualitative study of female caregiving spouses’ experiences of intimate relationships as cognition declines in Parkinson’s disease. Age Ageing. 2018;47:604–10. https://doi.org/10.1093/ageing/afy049

44. Vatter S., Stanmore E., Clare L., McDonald K.R., McCormick S.A., Leroi I. Care burden and mental ill health in spouses of people with Parkinson disease dementia and Lewy body dementia. J Geriatr Psychiatry Neurol. 2020;33:3–14. https://doi.org/10.1177/0891988719853043

45. Vatter S., McDonald K.R., Stanmore E., McCormick S.A., Clare L., Leroi I. A brief psychometric and clinimetric evaluation of self-report burden and mental health measures completed by care partners of people with Parkinson’s-related dementia. Int Psychogeriatr. 2020;32:875–80. https://doi.org/10.1017/S1041610220000605

46. Clare L, Rowlands J, Bruce E, Surr C, Downs M. ‘I don’t do like I used to do’: A grounded theory approach to conceptualising awareness in people with moderate to severe dementia living in long-term care. Soc sci med. 2008;66:2366–77. https://doi.org/10.1016/j.socscimed.2008.01.045

47. Ducharme F, Kergoat M-J, Antoine P, Pasquier F, Coulombe R. The unique experience of spouses in early-onset dementia. Am J Alzheimers Dis Other Demen. 2013;28:634–41. https://doi.org/10.1177/1533317513494443

48. Bentley A, Salifu Y, Walshe C. Applying an analytical process to longitudinal narrative interviews with couples living and dying with Lewy body dementia. Int J Qual Methods. 2021;20:16094069211060653. https://doi.org/10.1177/16094069211060653

49. Larsson V, Holmbom-Larsen A, Torisson G, Strandberg EL, Londos E. Living with dementia with Lewy bodies: an interpretative phenomenological analysis. BMJ Open. 2019;9:e024983. https://doi.org/10.1136/bmjopen-2018-024983

50. Morgan T., Duschinsky R., Gott M., Barclay S. Problematising carer identification: a narrative study with older partner’s providing end-of-life care. SSM- Qual Res Health. 2021;1:100015. https://doi.org/10.1016/j.ssmqr.2021.100015

51. Murphy C., Laine C.D., Macaulay M., Lennie K.H., Fader M. Problems faced by people living at home with dementia and incontinence: causes, consequences and potential solutions. Age Ageing. 2021;50:944–54. https://doi.org/10.1093/ageing/afaa262

52. Bakas T, Austin JK, Okonkwo KF, Lewis RR, Chadwick L. Needs, Concerns, Strategies, and Advice of Stroke Caregivers the First 6 Months After Discharge: J Neurosci Nurs. 2002;34:242–51. https://doi.org/10.1097/01376517-200210000-00004

53. Stacy KE, Perazzo J, Shatz R, Bakas T. Needs and concerns of Lewy body disease family caregivers: A qualitative study. West J Nurs Res. 2022;44:227–38. https://doi.org/10.1177/01939459211050957

54. Yumoto A, Suwa S. Difficulties and associated coping methods regarding visual hallucinations caused by dementia with Lewy bodies. Dementia (London). 2021;20:291–307. https://doi.org/10.1177/1471301219879541

55. Svendsboe EJ. Carers to people with Lewy body dementia and Alzheimer’s disease: Experiences and coping strategies. PhD. Thesis. [Sweden]: Karolinska Institutet; 2018.

56. Sabatini S, Martyr A, Ukoumunne OC, Ballard C, Collins R, Pentecost C, et al. Attitudes toward own aging and cognition among individuals living with and without dementia: Findings from the IDEAL programme and the PROTECT study. BMC Geriatr. 2022;22:641. https://doi.org/10.1186/s12877-022-03336-5

57. Dixon E., Anderson J., Blackwelder D.C., Radnofsky M.L., Lazar A. The human need for equilibrium: qualitative study on the ingenuity, technical competency, and changing strategies of people with dementia seeking health information. J Med Internet Res. 2022;24:e35072. https://doi.org/10.2196/35072

58. Armstrong M.J., Gamez N., Alliance S., Majid T., Taylor A.S., Kurasz A.M., et al. Clinical care and unmet needs of individuals with dementia with Lewy bodies and caregivers: an interview study. Alzheimer Dis Assoc Disord. 2021;35:327–34. https://doi.org/10.1097/WAD.0000000000000459

59. Galvin JE, Duda JE, Kaufer DI, Lippa CF, Taylor A, Zarit SH. Lewy body dementia: caregiver burden and unmet needs. Alzheimer Dis Assoc Disord. 2010;24:177–81. https://doi.org/10.1097/WAD.0b013e3181c72b5d

60. Tarolli CG, Zimmerman GA, Goldenthal S, Feldman B, Berk S, Siddiqi B, et al. Video research visits for atypical parkinsonian syndromes among Fox Trial Finder participants. Neurol Clin Pract. 2020;10:7–14. https://doi.org/10.1212/CPJ.0000000000000680

61. Brown LJE, Aldridge Z, Pepper A, Leroi I, Dening KH. ‘It’s just incredible the difference it has made’: Family carers’ experiences of a specialist Lewy body dementia Admiral Nurse service. Age Ageing. 2022;51:afac207. https://doi.org/10.1093/ageing/afac207

62. Bamford C, Wheatley A, Brunskill G, Booi L, Allan L, Banerjee S, et al. Key components of post-diagnostic support for people with dementia and their carers: a qualitative study. PLoS ONE. 2021;16:e0260506. https://doi.org/10.1371/journal.pone.0260506

63. Stuart J. Parkinson’s/Lewy body dementia: a carer’s perspective. J Dement Care. 2010;18:18–22.

64. Killen A, Flynn D, De Brún A, O’Brien N, O’Brien J, Thomas AJ, et al. Support and information needs following a diagnosis of dementia with Lewy bodies. Int Psychogeriatr. 2016;28:495–501. https://doi.org/10.1017/S1041610215001362

65. Galvin JE, Duda JE, Kaufer DI, Lippa CF, Taylor A, Zarit SH. Lewy body dementia: the caregiver experience of clinical care. Parkinsonism Relat Disord. 2010;16:388–92. https://doi.org/10.1016/j.parkreldis.2010.03.007

66. Miller C. My Friend Lewy. J Am Geriatr Soc. 2021;69:1399–400. https://doi.org/10.1111/jgs.17084

67. Toya S, Manabe Y, Hashimoto M, Yamakage H, Ikeda M. Questionnaire survey of satisfaction with medication for five symptom domains of dementia with Lewy bodies among patients, their caregivers, and their attending physicians. Psychogeriatrics. 2023;23:752–62. https://doi.org/10.1111/psyg.12993

68. Bied AM, Njuguna SW, Satodiya RM. Lumateperone for treatment of psychotic symptoms in Lewy body disease: a case report. Exp Clin Psychopharmacol. 2024;32:136–9. https://doi.org/10.1037/pha0000663

69. Shea C, MacKnight C, Rockwood K. Donepezil for treatment of dementia with Lewy bodies: A case series of nine patients. Int Psychogeriatr. 1998;10:229–38. https://doi.org/10.1017/S1041610298005341

70. Londos E. Practical treatment of Lewy body disease in the clinic: patient and physician perspectives. Neurol Ther. 2018;7:13–22. https://doi.org/10.1007/s40120-017-0090-8

71. Hashimoto M, Manabe Y, Yamaguchi T, Toya S, Ikeda M. Treatment needs of dementia with Lewy bodies according to patients, caregivers, and physicians: a cross-sectional, observational, questionnaire-based study in Japan. Alzheimers Res Ther. 2022;14:188. https://doi.org/10.1186/s13195-022-01130-4

72. Jackson G.A., Newbronner L., Chamberlain R., Borthwick R., Yardley C., Boyle K. Caring for people with dementia with Lewy bodies and Parkinson’s dementia in UK care homes - a mixed methods study. Eur Geriatr Med. 2017;8:146–52. https://doi.org/10.1016/j.eurger.2017.01.003

73. Martin J.T., Chapman K.R., Was C., Spitznagel M.B. Factors of dementia caregiver burden differentially contribute to desire to institutionalize. J Geriatr Psychiatry Neurol. 2022;35:594–600. https://doi.org/10.1177/08919887211036183

74. Larsson V, Engedal K, Aarsland D, Wattmo C, Minthon L, Londos E. Quality of life and the effect of memantine in dementia with Lewy bodies and Parkinson’s disease dementia. Dement Geriatr Cogn Disord. 2011;32:227–34. https://doi.org/10.1159/000334523

75. Lapid MI, Kuntz KM, Mason SS, Aakre JA, Lundt ES, Kremers W, et al. Efficacy, safety, and tolerability of armodafinil therapy for hypersomnia associated with dementia with Lewy bodies: a pilot study. Dement Geriatr Cogn Disord. 2017;43:269–80. https://doi.org/10.1159/000471507

76. Kurisu K, Terada S, Oshima E, Horiuchi M, Imai N, Yabe M, et al. Comparison of QOL between patients with different degenerative dementias, focusing especially on positive and negative affect. Int Psychogeriatr. 2016;28:1355–61. https://doi.org/10.1017/S1041610216000491

77. Boström F, Jönsson L, Minthon L, Londos E. Patients with dementia with Lewy bodies have more impaired quality of life than patients with Alzheimer disease. Alzheimer Dis Assoc Disord. 2007;21:150–4. https://doi.org/10.1097/WAD.0b013e318065c4a9

78. van de Beek M, van Steenoven I, van der Zande JJ, Porcelijn I, Barkhof F, Stam CJ, et al. Characterization of symptoms and determinants of disease burden in dementia with Lewy bodies: DEvELOP design and baseline results. Alzheimer’s Res Ther. 2021;13:53. https://doi.org/10.1186/s13195-021-00792-w

79. Armstrong M.J., Moore K., Jacobson C.E., Bedenfield N., Patel B., Sullivan J.L. Frequency of suicidal ideation and associated clinical features in Lewy body dementia. Parkinsonism Relat Disord. 2021;90:33–7. https://doi.org/10.1016/j.parkreldis.2021.07.029

80. Gratwicke J, Zrinzo L, Kahan J, Peters A, Brechany U, McNichol A, et al. Bilateral nucleus basalis of Meynert deep brain stimulation for dementia with Lewy bodies: a randomised clinical trial. Brain Stimul. 2020;13:1031–9. https://doi.org/10.1016/j.brs.2020.04.010

81. Ikeda M, Mori E, Orimo S, Yamada T, Konishi O. Efficacy of adjunctive therapy with zonisamide versus increased dose of levodopa for motor symptoms in patients with dementia with Lewy bodies: the randomized, controlled, non-inferiority DUEL study. J Alzheimer’s Dis. 2023;95:251–64. https://doi.org/10.3233/JAD-230335

82. Wu Y-T, Clare L, Hindle JV, Nelis SM, Martyr A, Matthews FE, et al. Dementia subtype and living well: results from the Improving the experience of Dementia and Enhancing Active Life (IDEAL) study. BMC Med. 2018;16:140. https://doi.org/10.1186/s12916-018-1135-2

83. van de Beek M, van Steenoven I, Ramakers IHGB, Aalten P, Koek HL, Olde Rikkert MGM, et al. Trajectories and determinants of quality of life in dementia with Lewy bodies and Alzheimer’s disease. J Alzheimer’s Dis. 2019;70:389–97. https://doi.org/10.3233/JAD-190041

84. van De Beek M, Van Unnik A, Van Steenoven I, Van Der Zande J, Barkhof F, Teunissen CE, et al. Disease progression in dementia with Lewy bodies: a longitudinal study on clinical symptoms, quality of life and functional impairment. Int J Geriatr Psychiatry. 2022;37:1–9. https://doi.org/10.1002/gps.5839

85. Chin KS, Churilov L, Doré V, Villemagne VL, Rowe CC, Yassi N, et al. Tau in dementia with Lewy bodies. Aust N Z J Psychiatry. 2024;58:175–82. https://doi.org/10.1177/00048674231177219

86. Chin KS, Gajamange S, Desmond PM, Villemagne VL, Rowe CC, Churilov L, et al. Association between amyloid-beta deposition and cortical thickness in dementia with Lewy bodies. Aust N Z J Psychiatry. 2023;57:594–602. https://doi.org/10.1177/00048674221081773

87. Onandia-Hinchado I, Diaz-Orueta U. Health related quality of life in individuals with cognitive decline and discrepancies between patients and their proxies. Arch Gerontol Geriatr. 2019;85:103914. https://doi.org/10.1016/j.archger.2019.103914

88. Onandia-Hinchado I, Diaz-Orueta U. Health related quality of life and cognitive decline in older populations: preliminary results from NeuroDemeNPsia study. J Appl Gerontol. 2020;39:618–26. https://doi.org/10.1177/0733464819866587

89. Abbate C, Trimarchi PD, Inglese S, Viti N, Cantatore A, De Agostini L, et al. Preclinical polymodal hallucinations for 13 years before dementia with Lewy bodies. Behav Neurol. 2014;2014:694296. https://doi.org/10.1155/2014/694296

90. Urwyler P, Nef T, Müri R, Archibald N, Makin SM, Collerton D, et al. Visual hallucinations in eye disease and Lewy body disease. Am J Geriatr Psychiatry. 2016;24:350–8. https://doi.org/10.1016/j.jagp.2015.10.007

91. Montagnese M, Vignando M, Collerton D, Ffytche D, Mosimann UP, Taylor J-P, et al. Cognition, hallucination severity and hallucination-specific insight in neurodegenerative disorders and eye disease. Cogn Neuropsychiatry. 2022;27:105–21. https://doi.org/10.1080/13546805.2021.1960812

92. Matterson E, Wilson-Menzfeld G, Olsen K, Taylor J-P, Elder GJ. Understanding the nature and impact of cognitive fluctuations and sleep disturbances in dementia with Lewy bodies: a qualitative caregiver study. SAGE Open Med. 2024;12:20503121241271827. https://doi.org/10.1177/20503121241271827

93. Machado FC, Oliveira FFD, Marin SDMC, Sampaio G, Bertolucci PHF. Correlates of neuropsychiatric and motor tests with language assessment in patients with Lewy body dementia. Arch Clin Psychiatry. 2020;47:75–81. https://doi.org/10.1590/0101-60830000000236

94. de Oliveira FF, Machado FC, Sampaio G, Marin S de MC, Naffah-Mazzacoratti M da G, Bertolucci PHF. Neuropsychiatric feature profiles of patients with Lewy body dementia. Clin Neurol Neurosurg. 2020;194:105832. https://doi.org/10.1016/j.clineuro.2020.105832

95. Oliveira FF, Machado FC, Sampaio G, Marin SM, Chen ES, Smith MC, et al. Contrasts between patients with Lewy body dementia syndromes and APOE-ε3/ε3 patients with late-onset Alzheimer disease dementia. Neurologist. 2015;20:35–41. https://doi.org/https://doi.org/10.1097/nrl.0000000000000045

96. Hashimoto M, Sakamoto S, Ikeda M. Clinical features of delusional jealousy in elderly patients with dementia. J Clin Psychiatry. 2015;76:691–5. https://doi.org/10.4088/JCP.14m09018

97. Onofrj M., Bonanni L., Manzoli L., Thomas A. Cohort study on somatoform disorders in Parkinson disease and dementia with Lewy bodies. Neurology. 2010;74:1598–606. https://doi.org/10.1212/WNL.0b013e3181df09dd

98. Onofrj M, Thomas A, Tiraboschi P, Wenning G, Gambi F, Sepede G, et al. Updates on somatoform disorders (SFMD) in Parkinson’s disease and dementia with Lewy bodies and discussion of phenomenology. J Neurol Sci. 2011;310:166–71. https://doi.org/10.1016/j.jns.2011.07.010

99. Shindo A, Ueda Y, Kuzuhara S, Kokubo Y. Neuropsychological study of amyotrophic lateral sclerosis and parkinsonism-dementia complex in Kii peninsula, Japan. BMC Neurol. 2014;14:151. https://doi.org/10.1186/1471-2377-14-151

100. Philippi N., Noblet V., Hamdaoui M., Soulier D., Botzung A., Ehrhard E., et al. The insula, a grey matter of tastes: A volumetric MRI study in dementia with Lewy bodies. Alzheimer’s Res Ther. 2020;12:79. https://doi.org/10.1186/s13195-020-00645-y

101. Galvin JE, Malcom H, Johnson D, Morris JC. Personality traits distinguishing dementia with Lewy bodies from Alzheimer disease. Neurology. 2007;68:1895–901. https://doi.org/10.1212/01.wnl.0000263131.80945.ad

102. Tabata K, Saijo Y, Morikawa F, Naoe J, Yoshioka E, Kawanishi Y, et al. Association of premorbid personality with behavioral and psychological symptoms in dementia with Lewy bodies: Comparison with Alzheimer’s disease patients. Psychiatry Clin Neurosci. 2017;71:409–16. https://doi.org/10.1111/pcn.12511

103. Reckner E., Cipolotti L., Foley J.A. Presence phenomena in parkinsonian disorders: Phenomenology and neuropsychological correlates. Int J Geriatr Psychiatry. 2020;35:785–93. https://doi.org/10.1002/gps.5303

104. Hu W., Liu S., Wang F., Zhu H., Du X., Ma L., et al. Autonomic symptoms are predictive of dementia with Lewy bodies. Parkinsonism Relat Disord. 2022;95:1–4. https://doi.org/10.1016/j.parkreldis.2021.11.023

105. Chapman K.R., Tremont G., Malloy P., Spitznagel M.B. The role of sexual disinhibition to predict caregiver burden and desire to institutionalize among family dementia caregivers. J Geriatr Psychiatry Neurol. 2020;33:42–51. https://doi.org/10.1177/0891988719856688

106. Shinagawa S, Adachi H, Toyota Y, Mori T, Matsumoto I, Fukuhara R, et al. Characteristics of eating and swallowing problems in patients who have dementia with Lewy bodies. Int Psychogeriatr. 2009;21:520–5. https://doi.org/10.1017/S1041610209008631

107. Gallop K, Pham N, Maclaine G, Saunders E, Black B, Acaster S. Exploring the impact of caring for an individual with neurogenic orthostatic hypotension: a qualitative study. Neurodegener Dis Manag. 2023;13:191–201. https://doi.org/10.2217/nmt-2022-0016

108. Chiu P-Y, Wang C-W, Tsai C-T, Li S-H, Lin C-L, Lai T-J. Depression in dementia with Lewy bodies: A comparison with Alzheimer’s disease. PLoS ONE. 2017;12:e0179399. https://doi.org/10.1371/journal.pone.0179399

109. Yust-Katz S., Hershkovitz R., Gurevich T., Djaldetti R. Pain in extrapyramidal neurodegenerative diseases. Clin J Pain. 2017;33:635–9. https://doi.org/10.1097/AJP.0000000000000437

110. Binnekade TT, Scherder EJA, Maier AB, Lobbezoo F, Overdorp EJ, Rhebergen D, et al. Pain in patients with different dementia subtypes, mild cognitive impairment, and subjective cognitive impairment. Pain Med. 2018;19:920–7. https://doi.org/10.1093/pm/pnx162

111. Lomax CL, Brown RG, Howard RJ. Measuring disability in patients with neurodegenerative disease using the ‘Yesterday Interview’. Int J Geriatr Psychiatry. 2004;19:1058–64. https://doi.org/10.1002/gps.1210

112. Liu S, Liu J, Wang X-D, Shi Z, Zhou Y, Li J, et al. Caregiver burden, sleep quality, depression, and anxiety in dementia caregivers: a comparison of frontotemporal lobar degeneration, dementia with Lewy bodies, and Alzheimer’s disease. Int Psychogeriatr. 2018;30:1131–8. https://doi.org/10.1017/S1041610217002630

113. Thaipisuttikul P, Lobach I, Zweig Y, Gurnani A, Galvin JE. Capgras syndrome in dementia with Lewy bodies. Int Psychogeriatr. 2013;25:843–9. https://doi.org/10.1017/S1041610212002189

114. Park J, Galvin JE. Pre-loss grief in caregivers of older adults with dementia with Lewy bodies. J Alzheimer’s Dis. 2021;82:1847–59. https://doi.org/10.3233/JAD-210616

115. Vatter S, McDonald KR, Stanmore E, Clare L, Leroi I. Multidimensional care burden in Parkinson-related dementia. J Geriatr Psychiatry Neurol. 2018;31:319–28. https://doi.org/10.1177/0891988718802104

116. Kanemoto H., Sato S., Satake Y., Koizumi F., Taomoto D., Kanda A., et al. Impact of behavioral and psychological symptoms on caregiver burden in patients with dementia with Lewy bodies. Front Psychiatry. 2021;12:753864. https://doi.org/10.3389/fpsyt.2021.753864

117. Huang W-C, Chang M-C, Wang W-F, Jhang K-M. A comparison of caregiver burden for different types of dementia: an 18-month retrospective cohort study. Front Psychol. 2022;12:798315. https://doi.org/10.3389/fpsyg.2021.798315

118. De Oliveira FF, Miraldo MC, De Castro-Neto EF, De Almeida SS, Matas SLDA, Bertolucci PHF, et al. Differential associations of clinical features with cerebrospinal fluid biomarkers in dementia with Lewy bodies and Alzheimer’s disease. Aging Clin Exp Res. 2023;35:1741–52. https://doi.org/10.1007/s40520-023-02452-5

119. van de Beek M, van Steenoven I, van der Zande JJ, Barkhof F, Teunissen CE, van der Flier WM, et al. Prodromal dementia with Lewy bodies: Clinical characterization and predictors of progression. Mov Disord. 2020;35:859–67. https://doi.org/10.1002/mds.27997

120. Mahale M., Behal P., Bewal N.M., Aggarwal V., Singhal A., Yadav A.K., et al. Study of prevalence of neuropsychiatric symptoms in elderly dementia patients. J Clin Diagnostic Res. 2021;15:14–7. https://doi.org/10.7860/JCDR/2021/44440.14510

121. Kawano Y, Terada S, Takenoshita S, Hayashi S, Oshima Y, Miki T, et al. Patient affect and caregiver burden in dementia. Psychogeriatrics. 2020;20:189–95. https://doi.org/10.1111/psyg.12487

122. Yuuki S, Hashimoto M, Koyama A, Matsushita M, Ishikawa T, Fukuhara R, et al. Comparison of caregiver burden between dementia with Lewy bodies and Alzheimer’s disease. Psychogeriatrics. 2023;23:682–9. https://doi.org/10.1111/psyg.12978

123. Tsai C.-F., Huang M.-H., Lee J.-J., Jhang K.-M., Huang L.-C., Huang L.-K., et al. Factors associated with burden among male caregivers for people with dementia. J Chin Med Assoc. 2022;85:462–8. https://doi.org/10.1097/JCMA.0000000000000704

124. Liu S, Jin Y, Shi Z, Huo YR, Guan Y, Liu M, et al. The effects of behavioral and psychological symptoms on caregiver burden in frontotemporal dementia, Lewy body dementia, and Alzheimer’s disease: Clinical experience in China. Aging Ment Health. 2017;21:651–7. https://doi.org/10.1080/13607863.2016.1146871

125. Lee DR, McKeith I, Mosimann U, Ghosh-Nodyal A, Thomas AJ. Examining carer stress in dementia: the role of subtype diagnosis and neuropsychiatric symptoms. Int J Geriatr Psychiatry. 2013;28:135–41. https://doi.org/10.1002/gps.3799

126. Hynninen M.J., Breitve M.H., Rongve A., Aarsland D., Nordhus I.H. The frequency and correlates of anxiety in patients with first-time diagnosed mild dementia. Int Psychogeriatr. 2012;24:1771–8. https://doi.org/10.1017/S1041610212001020

127. Ricci M, Guidoni SV, Sepe-Monti M, Bomboi G, Antonini G, Blundo C, et al. Clinical findings, functional abilities and caregiver distress in the early stage of dementia with Lewy bodies (DLB) and Alzheimer’s disease (AD). Arch Gerontol Geriatr. 2009;49:e101–4. https://doi.org/10.1016/j.archger.2008.10.001

128. Tzeng R-C, Tsai C-F, Wang C-T, Wang T-Y, Chiu P-Y. Delusions in patients with dementia with Lewy bodies and the associated factors. Behav Neurol. 2018;2018:6707291. https://doi.org/10.1155/2018/6707291

129. Terum TM, Testad I, Rongve A, Aarsland D, Svendsboe E, Andersen JR. The association between specific neuropsychiatric disturbances in people with Alzheimer’s disease and dementia with Lewy bodies and carer distress. Int J Geriatr Psychiatry. 2019;34:1421–8. https://doi.org/10.1002/gps.5134

130. Svendsboe E, Terum T, Testad I, Aarsland D, Ulstein I, Corbett A, et al. Caregiver burden in family carers of people with dementia with Lewy bodies and Alzheimer’s disease. Int J Geriatr Psychiatry. 2016;31:1075–83. https://doi.org/10.1002/gps.4433

131. Svendsboe EJ, Testad I, Terum T, Jörg A, Corbett A, Aarsland D, et al. Patterns of carer distress over time in mild dementia. Int J Geriatr Psychiatry. 2018;33:987–93. https://doi.org/10.1002/gps.4882

132. Rongve A, Vossius C, Nore S, Testad I, Aarsland D. Time until nursing home admission in people with mild dementia: Comparison of dementia with Lewy bodies and Alzheimer’s dementia. Int J Geriatr Psychiatry. 2014;29:392–8. https://doi.org/10.1002/gps.4015

133. Rice T, Dobry Y, Wang E, Novakovic V, Sher L. Cognitive effects of quetiapine in a patient with dementia with Lewy bodies. Psychogeriatrics. 2013;13:52–7. https://doi.org/10.1111/j.1479-8301.2012.00414.x

134. Iwasaki K, Kosaka K, Mori H, Okitsu R, Furukawa K, Manabe Y, et al. Improvement in delusions and hallucinations in patients with dementia with Lewy bodies upon administration of yokukansan, a traditional Japanese medicine. Psychogeriatrics. 2012;12:235–41. https://doi.org/10.1111/j.1479-8301.2012.00413.x

135. Iwasaki K, Kosaka K, Mori H, Okitsu R, Furukawa K, Manabe Y, et al. Open label trial to evaluate the efficacy and safety of yokukansan, a traditional Asian medicine, in dementia with Lewy bodies. J Am Geriatr Soc. 2011;59:936–8. https://doi.org/10.1111/j.1532-5415.2011.03373.x

136. Kasanuki K, Iseki E, Nishida Y, Fujishiro H, Chiba Y, Sato K, et al. Effectiveness of ramelteon for treatment of visual hallucinations in dementia with Lewy bodies: a report of 4 cases. J Clin Psychopharmacol. 2013;33:581–3. https://doi.org/10.1097/JCP.0b013e318295fdf4

137. Odawara T, Shiozaki K, Togo T, Hirayasu Y. Administration of zonisamide in three cases of dementia with Lewy bodies. Psychiatry Clin Neurosci. 2010;64:327–9. https://doi.org/10.1111/j.1440-1819.2010.02075.x

138. Thomas AJ, Burn DJ, Rowan EN, Littlewood E, Newby J, Cousins D, et al. A comparison of the efficacy of donepezil in Parkinson’s disease with dementia and dementia with Lewy bodies. Int J Geriatr Psychiatry. 2005;20:938–44. https://doi.org/10.1002/gps.1381

139. Emre M, Tsolaki M, Bonuccelli U, Destée A, Tolosa E, Kutzelnigg A, et al. Memantine for patients with Parkinson’s disease dementia or dementia with Lewy bodies: a randomised, double-blind, placebo-controlled trial. Lancet Neurol. 2010;9:969–77. https://doi.org/10.1016/S1474-4422(10)70194-0

140. Murata M, Odawara T, Hasegawa K, Iiyama S, Nakamura M, Tagawa M, et al. Adjunct zonisamide to levodopa for DLB parkinsonism: a randomized double-blind phase 2 study. Neurology. 2018;90:e664–72. https://doi.org/10.1212/WNL.0000000000005010

141. Mori E, Ikeda M, Kosaka K, Donepezil-DLB Study Investigators. Donepezil for dementia with Lewy bodies: A randomized, placebo-controlled trial. Ann Neurol. 2012;72:41–52. https://doi.org/10.1002/ana.23557

142. Mori E, Ikeda M, Nagai R, Matsuo K, Nakagawa M, Kosaka K. Long-term donepezil use for dementia with Lewy bodies: Results from an open-label extension of Phase III trial. Alzheimer’s Res Ther. 2015;7:5. https://doi.org/10.1186/s13195-014-0081-2

143. Ikeda M, Mori E, Kosaka K, Iseki E, Hashimoto M, Matsukawa N, et al. Long-term safety and efficacy of donepezil in patients with dementia with Lewy bodies: results from a 52-week, open-label, multicenter extension study. Dement Geriatr Cogn Disord. 2013;36:229–41. https://doi.org/10.1159/000351672
